# Mechanistic Characterisation and Engineering of Sesterviolene Synthase from *Streptomyces violens*

**DOI:** 10.1002/anie.202215688

**Published:** 2022-12-01

**Authors:** Binbin Gu, Bernd Goldfuss, Jeroen S. Dickschat

**Affiliations:** ^1^ Kekulé-Institute for Organic Chemistry and Biochemistry University of Bonn Gerhard-Domagk-Straße 1 53121 Bonn Germany; ^2^ Department for Chemistry University of Cologne Greinstraße 4 50939 Cologne Germany

**Keywords:** Biosynthesis, Enzyme Engineering, Enzyme Mechanisms, Isotopes, Terpenoids

## Abstract

The sesterviolene synthase from *Streptomyces violens* was identified and represents the second known sesterterpene synthase from bacteria. Isotopic labelling experiments in conjunction with DFT calculations were performed that provided detailed insight into its complex cyclisation mechanism. Enzyme engineering through site‐directed mutagenesis gave access to a high‐yielding enzyme variant that provided six additional minor products and the main product in sufficient quantities to study its chemistry.

## Graphical abstract

Sesterviolene synthase from *Streptomyces violarus* is the second bacterial sesterterpene synthase to be described. The enzyme generates one main and several side products whose mechanisms of formation were studied in isotopic labelling experiments and through DFT calculations. Enzyme engineering allowed isolation and structural characterisation of the side products and gave access to the main product in sufficient quantity to study its chemistry.

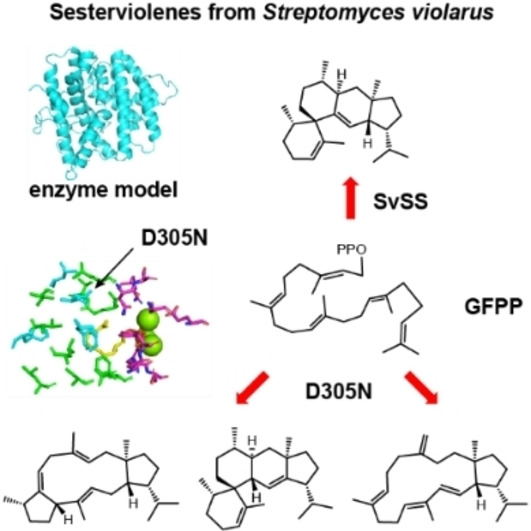

The biosynthesis of terpenes is a fascinating process in which acyclic and achiral oligoprenyl diphosphate precursors are converted into usually polycyclic and complex frameworks with multiple stereogenic centers. These reactions are multistep cascades via cationic intermediates that are generated in the case of class I terpene synthases (TS) by the abstraction of diphosphate,^[1]^ or in the case of class II TS by protonation of the substrate.^[2]^ Occasionally, the cascade can also be initiated through transfer of an electrophile such as Br^+^ or CH_3_^+^.^[3]^ The oligoprenyl diphosphates are constructed from only two building blocks, the electrophile dimethylallyl diphosphate (DMAPP) and the nucleophile isopentenyl diphosphate (IPP), which are fused by prenyltransferases (PT) to successively yield geranyl (GPP, monoterpenes), farnesyl (FPP, sesquiterpenes), geranylgeranyl (GGPP, diterpenes), and geranylfarnesyl diphosphate (GFPP, sesterterpenes).^[4]^ Only recently it was shown that also farnesylfarnesyl diphosphate (FFPP) can be converted by fungal bifunctional PT‐TS enzymes into triterpenes, a class of compounds that was previously believed to originate solely from squalene.^[5]^ Also diterpene synthases (DTS) and sesterterpene synthases (StTS) are often bifunctional in fungi.^[6]^ In contrast, two discrete enzymes are found in plants.^[7]^ This is paralleled in bacteria, in which class I diterpenes and sesterterpenes are generated from a discrete GGPP synthase (GGPPS) or GFPP synthase (GFPPS) and a TS, with the sestermobaraene synthase from *Streptomyces mobaraensis* (SmTS1) being the only known bacterial class I StTS.^[8]^ Herein we report on the discovery of the StTS for sesterviolenes from *S. violens*, its mechanistic characterisation and engineering to a high‐yielding enzyme that enabled intensive study of the chemistry of its main product.

A phylogenetic tree constructed from 3278 amino acid sequences of bacterial TS homologues (Figure S1) identified a few candidate enzymes that are closely related to SmTS1. The TS homologue from *S. violens* showing 43 % sequence identity to SmTS1 was selected for further study through gene cloning and expression in *Escherichia coli*. The purified enzyme (Figure S2) converted GFPP into one major sesterterpene hydrocarbon beside a few trace compounds (Figure S3), while GPP, FPP and GGPP were not accepted. The main product was isolated (3 % yield from GFPP) and structurally characterised by NMR spectroscopy (Figures S4–S11, Table S2), resulting in the structure of sesterviolene A (**1**), which represents a novel skeleton (Scheme 1). The TS for **1** was thus identified as *Streptomyces violens* Sesterviolene Synthase (SvSS).

**Scheme 1:**
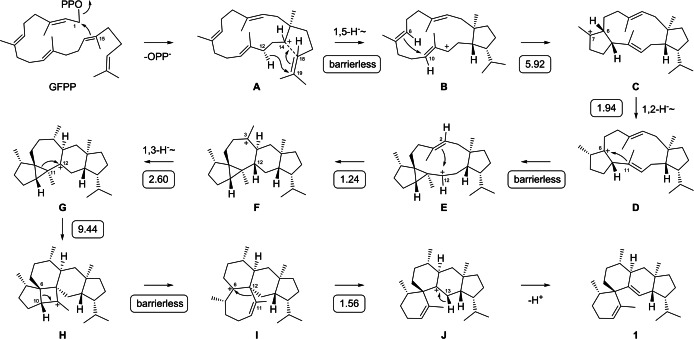
Cyclisation mechanism from GFPP to **1**. Numbers in boxes are computed reaction barriers (mPW1PW91/6‐311+G(d,p)//B97D3/6‐31G(d,p), Gibbs energies at 298 K in kcal mol^−1^).

The proposed cyclisation mechanism from GFPP to **1** proceeds through a 1,15‐ and 14,18‐cyclisation with a 1,5‐hydride shift from C12 to C19 towards **B** (the reasoning for cation **A** is discussed below). A subsequent 6,10‐cyclisation to **C**, 1,2‐hydride shift to **D** and 6,11‐cyclisation lead to **E**, which further reacts through 2,12‐cyclisation to **F** and 1,3‐hydride shift to **G**. Such 1,3‐hydride shifts are possible in terpene biosynthesis, but only for *trans*‐fused ring systems.^[9]^ Ring expansion to **H** and opening of the cyclobutane ring result in **I**, which can undergo ring contraction to **J** and deprotonation to **1**.

The cyclisation mechanism to **1** was experimentally investigated through isotopic labelling experiments (Table S3). For this purpose, all 25 isotopomers of (^13^C_1_)GFPP, generated enzymatically with GFPPS from *S. mobaraensis* ^[8b]^ from correspondingly labelled shorter terpene precursors, were converted with SvSS into **1** with incorporation of ^13^C‐labelling at the expected positions in all cases (Figure S12). Specifically, the obtained data were in agreement with the skeletal rearrangement from **I** to **J**, disconnecting C11 and C12, and revealed high face selectivity for the 1,5‐hydride shift from C12 to C19 in the conversion of **A** into **B**, as indicated by the fact that the labelling from C20 and C21 was not distributed over the two geminal Me groups in the product. The 1,5‐hydride shift from **A** to **B** was investigated using (7‐^13^C)GPP^[10]^ and (*E*)‐ or (*Z*)‐(4‐^13^C,4‐^2^H)IPP^[11]^ in conjunction with GFPPS and SvSS. ^13^C NMR analysis showed a triplet for C19 (^13^C−^2^H spin coupling) in the experiment with (*E*)‐(4‐^13^C,4‐^2^H)IPP, revealing specific migration of the hydrogen atom derived from 4H_*E*_ of IPP (Scheme S1, Figure S13). The 1,2‐hydride migration from **C** to **D** was explored using (3‐^13^C,2‐^2^H)GGPP^[12]^ and IPP with GFPPS and SvSS, resulting in a triplet for C7 of **1** in the ^13^C NMR spectrum (Scheme S2, Figure S14). Due to peak broadening of the signal for this carbon atom at 298 K, the triplet was only observable at higher temperature (343 K). The 1,3‐hydride shift from C12 to C3 in the step from **F** to **G** was investigated through conversion of GPP with (4,4‐^2^H_2_)IPP and (3‐^13^C)IPP with GFPPS and SvSS. This combination of substrates yields mixtures of isotopomers, but only the four isotopomers in which the fifth isoprene unit of GFPP is ^13^C‐labelled from (3‐^13^C)IPP are relevant for the detection of C3 by ^13^C NMR spectroscopy (Scheme S3), while four more isotopomers in which the fifth isoprene unit is deuterated from (4,4‐^2^H_2_)IPP do not result in a strong signal for C3 (not shown). Only if the third unit of GFPP is derived from (4,4‐^2^H_2_)IPP, C12 is deuterated and the 1,3‐hydride shift becomes visible by a triplet signal for C3 (Schemes S3A and S3B, Figure S15), but not if the third unit arises from (3‐^13^C)IPP (this results in a singlet for C3, Schemes S3C and S3D, Figure S15). Finally, the terminal deprotonation step to **1** was investigated with (*R*)‐ and (*S*)‐(1‐^2^H)‐GPP^[13]^ and IPP, demonstrating the specific loss of deuterium from (*S*)‐(1‐^2^H)‐GPP (Figure S16). Because of the inversion of configuration at C1 of oligoprenyl diphosphates in the elongation with IPP,^[14]^ this is the 13‐*pro*‐*S* hydrogen atom in **J** (top face in Scheme 1).

The absolute configuration of **1** was determined through a stereoselective deuteration approach using DMAPP and (*R*)‐ or (*S*)‐(1‐^13^C,1‐^2^H)IPP (Figure S17), and (*E*)‐ or (*Z*)‐(4‐^13^C,4‐^2^H)IPP (Figure S18). With these substrates, stereochemical anchors of known configuration are introduced into **1** that allow determination of its absolute configuration by resolving the relative configuration of the naturally present stereogenic centers in **1** with respect to these anchors. The additional ^13^C label serves as a highly sensitive probe for analysis through HSQC spectroscopy. The results revealed the absolute configuration of **1** as shown in Scheme 1.

Site‐directed mutagenesis of terpene synthases^[15]^ is a promising approach to study their mechanisms and to obtain new enzyme variants with improved production yields or altered product profiles towards new compounds. The residues contouring the active site of SvSS were identified through sequence alignment of SvSS with selinadiene synthase (SdS), for which the active‐site residues are known from the crystal structure in the fully closed conformation^[16]^ (Figures S19). Although for polytrichastrene synthase (CpPS) this approach had recently resulted in the highly interesting I66F variant, which showed higher activity and the formation of several new diterpenes in comparison to the wildtype,^[17]^ for SvSS the analogous mutant (L59F) only showed strongly reduced activity (7.5±0.3 % of wildtype level, Figure 1, Table S5). Also, the exchange of other active‐site residues (shown by entries 2–12 in Figure 1, green bars, and Figure 2C, green residues) only resulted in diminished enzyme activities.

**Figure 1:**
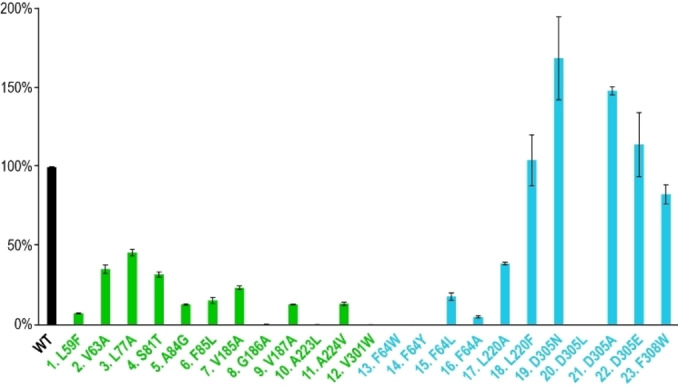
Relative activity of wildtype SvSS (WT) and enzyme variants obtained by site‐directed mutagenesis for the production of **1**. Error bars indicate standard deviations from triplicates. The colour code refers to the colouration of residues as in Figure 2C.

**Figure 2:**
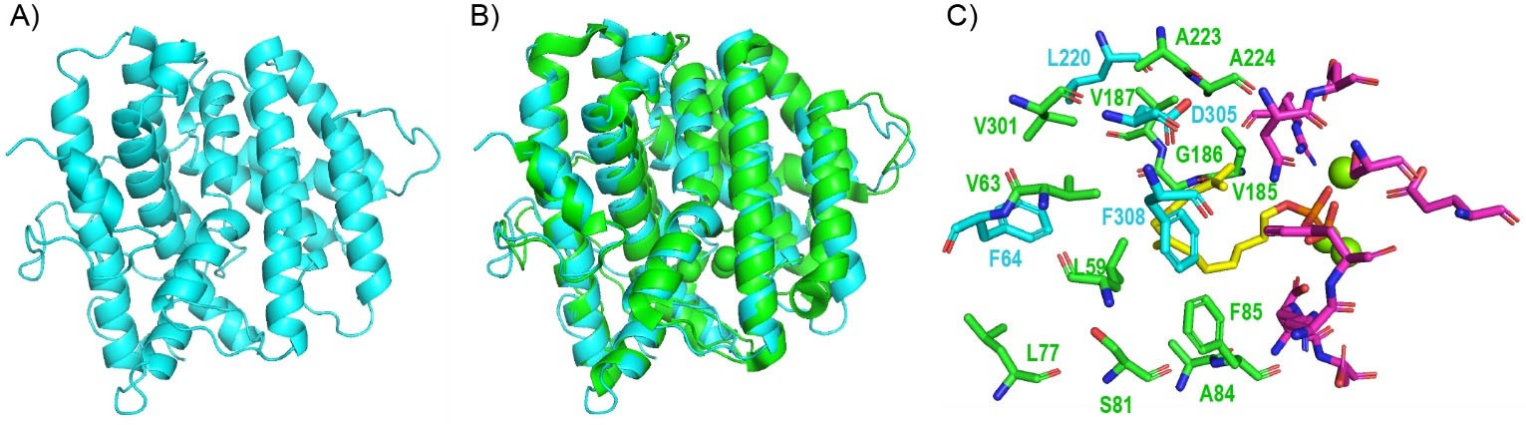
Enzyme models of SvSS. A) Enzyme model generated with AlphaFold2. B) Alignment of the AlphaFold2 model (cyan) with the crystal structure of SdS (green, PDB code: 4OKZ). C) Active‐site model combining active‐site residues of SvSS with Mg^2+^ cations (green spheres) and the co‐crystallised substrate analogue DHFPP (yellow) from the SdS crystal structure. Active‐site residues important for substrate and Mg^2+^ binding are shown in purple, those selected for mutation based on the sequence alignment between SvSS and SdS are shown in green, and those selected for mutation based on the AlphaFold2 model are shown in cyan.

To gain further insight into the active‐site architecture of SvSS, an enzyme model was created using AlphaFold2 (Figure 2A).^[18]^ The alignment of this model to the SdS crystal structure showed a highly similar α‐helical fold, but also some pronounced conformational differences with a root mean square deviation (rmsd) of 1.449 Å (Figure 2B). Based on this alignment, four additional active‐site residues of SvSS were selected for mutation (F64, L220, D305 and F308, Figure 1, cyan bars, and Figure 2C, cyan residues). The exchange of F64 for other aromatic (F64W, F64Y) or aliphatic residues (F64L, F64A) resulted in strongly reduced catalytic activity in all cases (Figure 1, entries 13–16). Also, the exchange of L220 for a smaller (L220A) residue gave reduced product yields, while the L220F variant showed similar activity to that of the wildtype. Interestingly, the D305N variant showed increased activity (169±26 %), while this effect was less pronounced for the D305A (148±3 %) and D305E variants (114±20 %), and the D305L variant was inactive. Finally, the F308W variant had similar activity to the wildtype.

The D305N variant not only showed highest activity, but also gave significantly improved enzyme yields in heterologous expressions (wildtype: 8 mg L^−1^, D305N: 29 mg mL^−1^). Under improved incubation conditions with the addition of β‐cyclodextrin, 150 mg of GFPP were converted with the D305N variant to yield 20 mg of **1** (22 % yield). Moreover, the production of the minor compounds already observed with wildtype SvSS was also increased (Figure S21), allowing their isolation and structure elucidation (Figures S22–S69, Tables S6–S11). The newly obtained compounds **2**–**7** were named sesterviolenes B–G (Figure 3).

**Figure 3:**
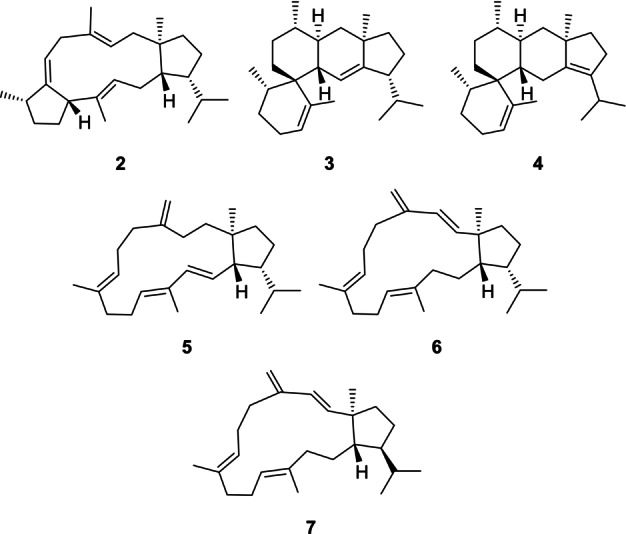
Structures of sesterviolenes B–G (**2**–**7**).

Biosynthetically, **2** can directly arise from **D** by deprotonation, while **3** requires a 1,2‐hydride shift from **J** and deprotonation, and **4** can be formed from **J** by two sequential 1,2‐ or a single 1,3‐hydride shift and deprotonation (Scheme S4). The formation of **5** and **6** must proceed with transannular proton or hydride shifts, similar to the reactions observed previously with the sestermobaraene synthase SmTS1.^[8b]^ Compound **7** is the C18 epimer of **6**, pointing to a slightly changed conformational fold of GFPP in the initial cyclisations.

Because of the low production of **5**–**7** in conjunction with a deuterium kinetic isotope effect that further lowered their yields, trials to follow the transannular hydrogen migrations in their biosynthesis by isotopic labelling failed. Therefore, DFT calculations were performed to further investigate the cyclisation mechanism of SvSS (Table S12, Figure S70). Along the main pathway to **1** (Scheme 1), instead of a concerted 1,15–14,18‐cyclisation of GFPP, the secondary cation **A** could be localised as a minimum that reacts in a barrierless manner to give **B** with simultaneous ring closure and hydride shift. All other transformations proceeded with low transition‐state (TS) barriers or even barrierless, with the highest TS barrier found for the rearrangement from **G** to **H**. Notably, the cyclopropanation from **D** to **E** was found to be barrierless, and the ring contraction from **I** to **J** was associated with a very low TS barrier (1.56 kcal mol^−1^). Low TS barriers were also computed for the transannular hydrogen shifts in the biosynthesis of **5**–**7**, showing the feasibility of the proposed steps (Scheme S4). The calculations could not resolve the question whether **4** is formed from **J** through two sequential 1,2‐hydride shifts or a single 1,3‐hydride migration, as the TS barriers for these pathways were, at 10.78 kcal mol^−1^ and 10.96 kcal mol^−1^, very similar. An isotopic labelling experiment using (2‐^2^H)GPP and (4‐^13^C)IPP with GFPPS and SvSS allowed us to distinguish between these alternatives, indicating a sequence of two 1,2‐hydride shifts by a moderate deshielding (Δδ=−0.08 ppm) for C12 caused by a deuterium at the neighbouring carbon atom C13 (Scheme S5, Figure S71). Also, the loss of deuterium in the formation of **3** was in line with this model (Scheme S5, Figure S72).

The high yields of **1** obtained with the D305N variant allowed us to study the chemistry of this sesterterpene (Scheme 2A). Interestingly, simple exposure of the main product **1** to air for 24 h resulted in the accumulation of the autoxidation product 14,18‐dehydrosesterviolene A (**8**, Figures S73–S80, Table S13), which was slowly further converted into sesterviolene epoxide A (**9**) under these conditions (Figures S81–S88, Table S14). Potentially, **4** could form the same autoxidation products, but for this compound no changes were observed after prolonged standing in air. The different behaviour of these sesterterpenes may be explained by a release of strain from **1** resulting from a steric interaction of the *i*Pr group with Me‐23 that is less pronounced in **8** (Scheme 2B).

**Scheme 2:**
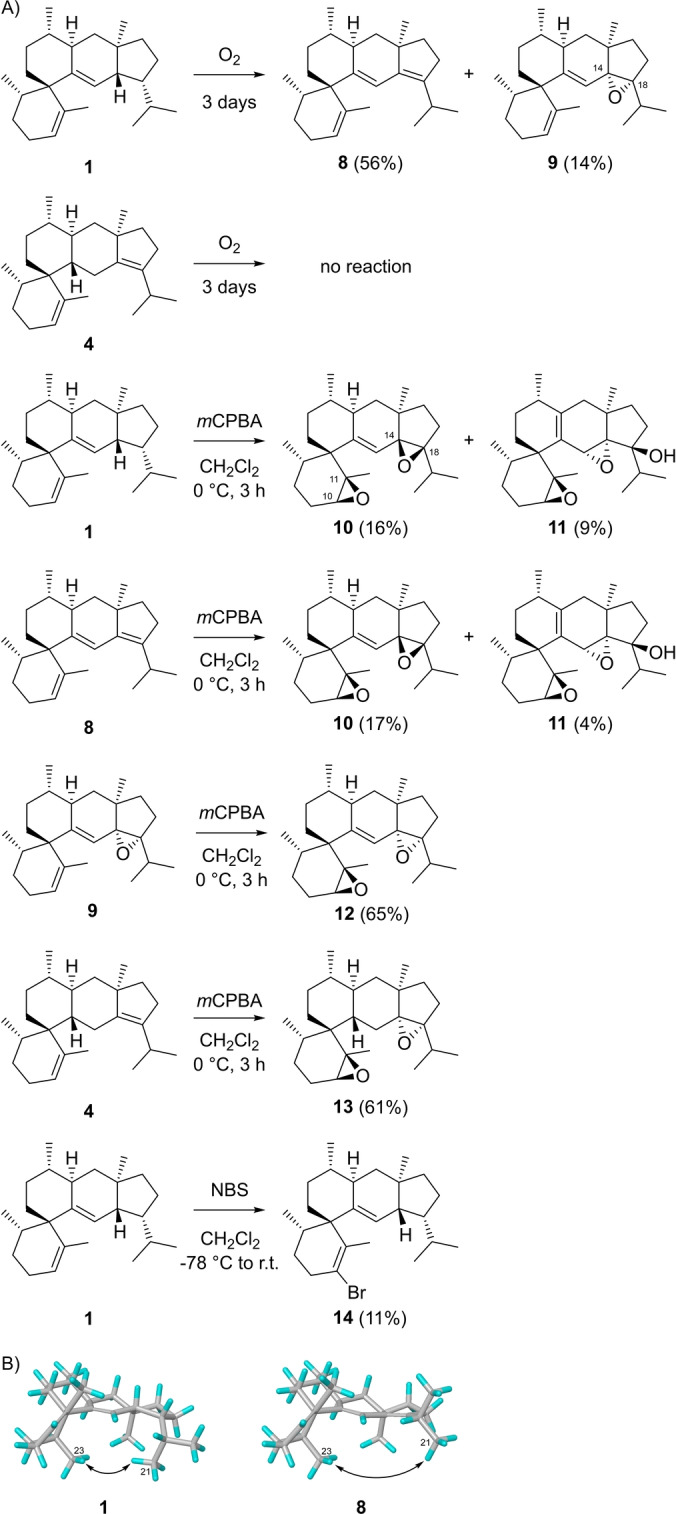
A) Synthetic transformations of **1**. B) Strain release in the conversion of **1** into **8**.

The treatment of **1** with *meta*‐chloroperbenzoic acid (*m*CPBA) also proceeded with formation of a conjugated diene system and likely subsequent epoxidation. From the complex product mixture, the two bis‐epoxides sesterviolene epoxide B (**10**) and C (**11**) were isolated (Figures S89–S104, Tables S15 and S16), in which not only were epoxide functionalities introduced, but the carbon skeleton was oxidised. In **10**, the 10,11‐ and the 14,18‐epoxide functionalities were installed from the sterically less hindered sides, with the 14,18‐epoxide showing opposite configurations to those in **9**. The small oxidant O_2_ may attack **1** from the less accessible side, leading to the thermodynamic product **9**. The *m*CPBA‐mediated formation of **10** and **11** from **1** may proceed via intermediate **8**. This hypothesis was tested by the oxidation of **8** with *m*CPBA, which resulted in the same mixture of **10** and **11** as obtained from **1** (Figures S105 and S106). Furthermore, the epoxidation of **9** yielded sesterviolene epoxide D (**12**, Figures S107–S114, Table S17), a stereoisomer of **10**. Interestingly, the reaction of **4** with *m*CPBA gave the expected bis‐epoxide sesterviolene epoxide E (**13**, Figures S115–S122, Table S18), showing that in **4** with a saturated C12/C13 bond, the lower side of the C14/C18 double bond is more easily accessible. Besides these oxidations, **1** was treated with *N*‐bromosuccinimide (NBS), which resulted in a low yield of 10‐bromosesterviolene A (**14**, Figures S123–S130, Table S19).

In summary, we have identified and mechanistically characterised the second bacterial sesterterpene synthase SvSS for sesterviolenes from *Streptomyces violarus*. The enzyme produces one main compound besides several other sesterterpene hydrocarbons, but their production was too low for compound isolation. Therefore, enzyme engineering through site‐directed mutagenesis based on an AlphaFold2 enzyme model was used to gain access to an enzyme variant (D305N) that showed much higher expression levels and better catalytic performance. The deeper reason for this intriguing effect and a possible catalytic function of the D305 residue are currently unclear and will require future structural work on SvSS. Not ony did the D305N enzyme variant enable isolation of the minor side products for structure elucidation, showing the high potential of structure‐model‐based enzyme engineering, but the main compound was made accessible in good yields, thus enabling its chemistry to be studied. Sesterviolene A is notoriously unstable and readily oxidised in air, with installation of a conjugated double bond that presumably releases strain from **1**, followed by spontaneous epoxidation. Treatment with *m*CPBA caused similar reactions, but epoxidation at C14/C18 proceeded from the opposite site, and resulted in further oxidations. Similar to the sestermobaraene synthase from *S. mobaraensis*, SvSS is genetically clustered with a prenyltransferase likely for GFPP biosynthesis that shows 49 % identity to the characterised GFPPS from *S. mobaraensis*.^[8b]^ However, no cytochrome P450 is encoded near the gene for SvSS that could account for the biosynthetic oxidation of **1**. It is also interesting to note that the gene for SvSS does not seem to be expressed under laboratory culture conditions, as the analysis of volatiles released by *S. violarus* did not show the presence of **1** or its autoxidation products **8** and **9**. Many terpenoids have been isolated from bacteria to date,^[19]^ but the work presented herein demonstrates that genome mining continues to be an interesting approach to tap bacterial sources.

## Conflict of interest

The authors declare no conflict of interest.

## Supporting information

As a service to our authors and readers, this journal provides supporting information supplied by the authors. Such materials are peer reviewed and may be re‐organized for online delivery, but are not copy‐edited or typeset. Technical support issues arising from supporting information (other than missing files) should be addressed to the authors.

Supporting Information

## Data Availability

The data that support the findings of this study are available in the supplementary material of this article.
