# Supplementary material for: Mechanistic Characterisation and Engineering of Sesterviolene Synthase from Streptomyces violens
Source: Angew Chem Int Ed Engl. 2022 Dec 1;62(1):e202215688. doi: 10.1002/anie.202215688 (PMC10107272; doi:10.1002/anie.202215688)
Supplement: Supplementary file 1 — Supporting Information [file ANIE-62-0-s001.pdf]

Angewandte Chemie
Eine Zeitschrift der Gesellschaft Deutscher Chemiker

Supporting Information

# Mechanistic Characterisation and Engineering of Sesterviolene Synthase from *Streptomyces violens*

*B. Gu, B. Goldfuss, J. S. Dickschat**

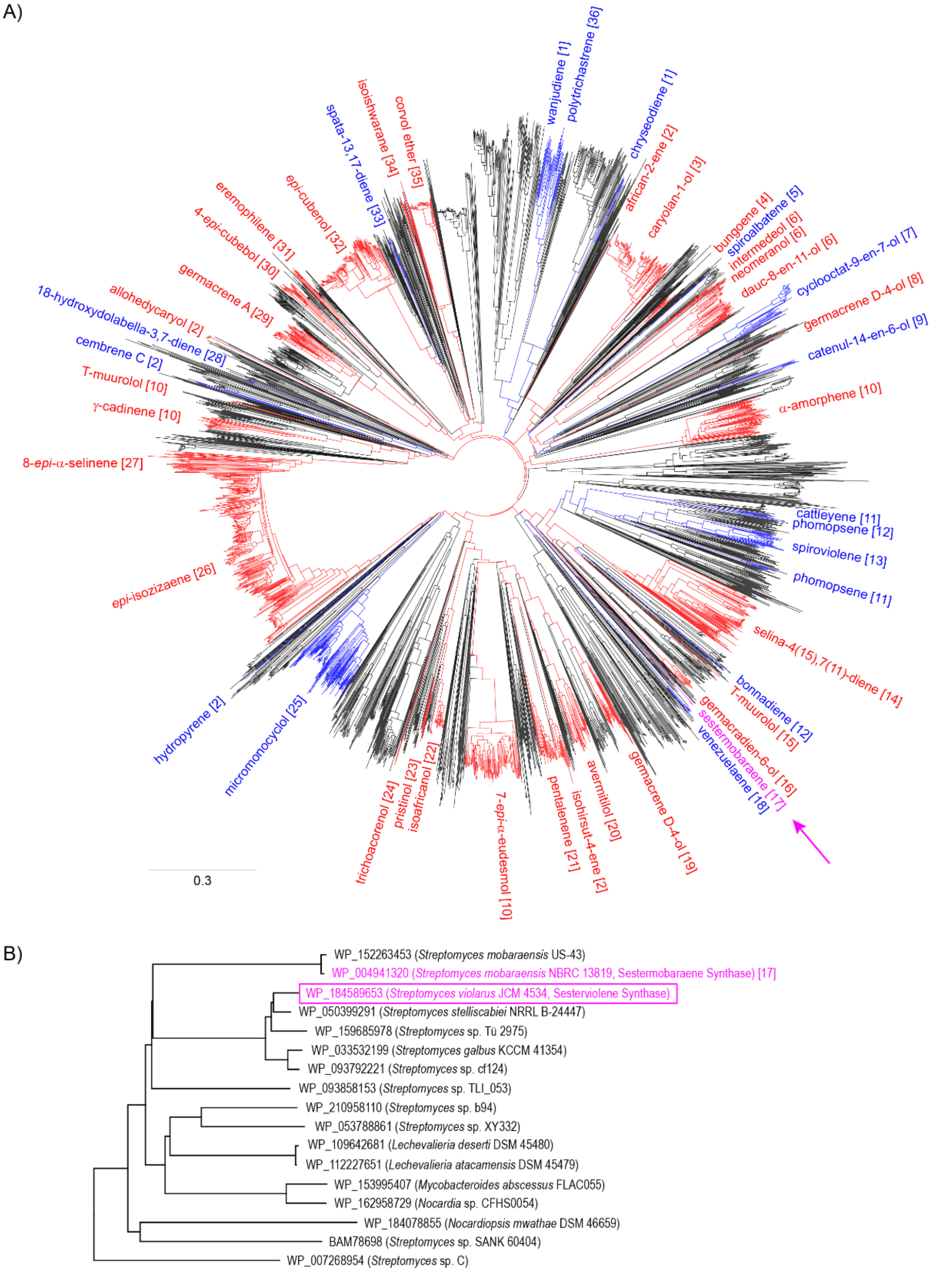


**Figure S1.** A) Phylogenetic tree constructed from 3278 amino acid sequences of bacterial terpene synthase homologs. The tree was built using the tree builder function of Geneious (alignment type: global alignment with free end gaps, cost matrix: Blosum45, genetic distance model: Jukes-Cantor, tree build method: neighbor-joining, gap open panelty: 8, gap extension penalty: 2; the bar indicates substitutions per site). Functionally characterized enzymes[1-36] (references are also indicated in the tree) and their closest relatives with likely the same function are shown in red (sesquiterpene synthases), blue (diterpene synthases) and purple (sesterterpene synthases). The arrow indicates the location of the sestermobaraene synthase from *S. mobaraensis*. B) Enlargement showing the region of sestermobaraene synthase and its closest homologs. The enzyme investigated in this study is shown in the box.

**General**

Chemicals were purchased from Sigma Aldrich Chemie GmbH (Steinheim, Germany), Carbolution Chemicals GmbH (St. Ingbert, Germany), or Carl Roth (Karlsruhe, Germany) and used without purification. Solvents for column chromatography were purchased in p.a. grade and purified by distillation. Thin-layer chromatography was performed with 0.2 mm precoated plastic sheets Polygram Sil G/UV254 purchased from Machery-Nagel (Düren, Germany). Column chromatography was performed using silica gel 60 purchased from Merck (Darmstadt, Germany).

**GC/MS and GC/MS-QTOF analyses**

GC/MS analyses were carried out on a 7890B/5977A series gas chromatography/mass selective detector (Agilent, Santa Clara, CA, USA). The GC was equipped with an HP5-MS fused silica capillary column (30 m, 0.25 mm i. d., 0.50 µm film; Agilent) and operated using the settings: 1) inlet pressure: 77.1 kPa, He at 23.3 mL $min^{-1}$, 2) injection volume: 1 µL, 3) temperature program: 5 min at 50 °C then increasing 10 °C $min^{-1}$ to 320 °C, 4) splitless or split ratio 50:1, 60 s valve time, and 5) carrier gas: He at 1 mL $min^{-1}$. The MS was operated with settings: 1) source: 230 °C, 2) transfer line: 250 °C, 3) quadrupole: 150 °C and 4) electron energy: 70 eV. Retention indices (*I*) were determined from a homologous series of *n*-alkanes ($C_7$-$C_{40}$).

GC/MS-QTOF analyses were performed on a 7890B GC equipped with a HP5-MS fused silica capillary column (30 m, 0.25 mm i. d., 0.50 µm film) connected to a 7200 accurate-mass QTOF detector (Agilent). GC parameters were 1) inlet pressure: 83.2 kPa, He at 24.6 mL $min^{-1}$, 2) injection volume: 1 µL, 3) split ratio: 50:1, 60 s valve time, 4) temperature program: 5 min at 50 °C increasing at 5 °C $min^{-1}$ to 320 °C, 5) carrier gas: He at 1 mL $min^{-1}$. MS parameters were 1) transfer line: 250 °C, 2) electron energy 70 eV.

**HRMS**

High resolution mass spectra were recorded with LTQ Orbitrap XL (Thermo Fisher Scientific, Waltham. Massachusetts, USA).

**NMR spectroscopy**

NMR spectra were recorded on a Bruker Avance III HD 700 MHz Cryo spectrometer. Chemical shifts were referenced to the residual proton signal of $C_6D_6$ ($\delta$ = 7.16 ppm) for $^1$H NMR and the $^{13}$C signal of $C_6D_6$ ($\delta$ = 128.06 ppm) for $^{13}$C NMR.[37]

**IR spectroscopy**

IR spectra were recorded on a Bruker $\alpha$ infrared spectrometer with a diamond ATR probehead. Peak intensities are given as s (strong), m (medium), w (weak) and br (broad).

**Optical rotations**

Optical rotations were recorded on a Modular Compact Polarimeter MCP 100 (Anton Paar, Graz, Austria). The temperature setting was 25 °C; the wavelength of the light used was 589 nm (sodium D line); the path-length was 10 cm; the compound concentrations *c* are given in g 100 $mL^{-1}$.

**Strains and culture conditions**

The strain *Streptomyces violarus* DSM 40205 was obtained from Deutsche Sammlung von Mikroorganismen und Zellkulturen (Braunschweig, Germany). This strain was cultivated in 65. GYM medium (4.0 g glucose, 4.0 g yeast extract, 10.0 g malt extract, dissolved in 1 L distilled water, pH 7.2) at 28 °C.

**Isolation of genomic DNA**

*S. violarus* DSM 40205 cells from a 65. GYM liquid culture (100 mL) were harvested by centrifugation, resuspended in SET buffer (5 mL, 75 mM NaCl, 25 mM EDTA, 20 mM Tris/HCl, pH 8.0) and incubated with lysozyme solution (1 mg/mL) for 30 min at 37 °C. Proteinase K solution (100 µL, 1 mg/mL) was added and the solution was mixed. It was incubated for 1 h at 55 °C after addition of 10% SDS (600 µL) and mixing by inversion. Phenol/chloroform (5 mL) was added and the solution was mixed by inversion before centrifugation for 5 min at 14000 g. The aqueous layer was transferred to a fresh tube and ice-cold ethanol (60%-70% vol.) was added for precipitation of DNA. After centrifugation and washing with 70% ethanol the DNA was redissolved in TE buffer (100 µL, 10 mM Tris/HCl, 1 mM EDTA, pH 8.0).

**Gene cloning**

The target gene of SvSS from *S. violarus* DSM 40205 (WP_184589653) was amplified from gDNA by PCR using Q5 High-fidelity DNA polymerase (New England Biolabs, Ipswich, MA, USA) and the primer pair in Table S1 named after the accession number. Yeast homologous recombination of the PCR product with the linearised pYE-Express shuttle vector[30] was carried out through the standard protocol using LiOAc, polyethylene glycol and salmon sperm DNA.[38] After yeast transformation culture was grown on SM-URA agar (425 mg yeast nitrogen base, 1.25 g ammonium sulphate, 5 g glucose, 192.5 mg nutritional supplement minus uracil, 5 g agar, 250 mL water) at 28 °C for 3 days. The recombinant plasmid was isolated from grown yeast colonies using the Zymoprep Yeast Plasmid Miniprep II kit (Zymo Research, Irvine, CA, USA) and subsequently used for transformation of *E. coli* BL21 (DE3) electrocompetent cells. Cells were plated on LB agar plates with Kanamycin (50 µg $mL^{-1}$) followed by incubation at 37 °C overnight. Single colonies were selected and used to inoculate LB medium (6 mL) liquid cultures with kanamycin (6 µL; 50 mg $mL^{-1}$). After 24 h growth plasmid DNA was isolated and checked for correct insertion of the desired gene by PCR amplifying the DNA sequence, containing the target gene, using T7 primer pair and by sequencing. The obtained plasmid was named pYE-WP_184589653.

**Table S1.** Primers used in this study.

| **Primer** | **Sequence**[a] |
|---|---|
| WP_184589653_F | GGCAGCCATATGGCTAGCATGACTGGTGGAatgcaccctgccgacctccc |
| WP_184589653_R | TCTCAGTGGTGGTGGTGGTGGTGCTCGAGTGttatgatgcgatgtgatcccaccaccaggc |

[a] The sequences of capital letters are homology arms for recombination in yeast which match the terminal sequences of linearised pYE-Express (HindIII and EcoRI digestion).

**Gene expression and enzyme purification**

A preculture of *E. coli* BL21(DE3) transformed with pYE-WP_184589653 (SvSS) was grown in LB medium with kanamycin (50 µg $mL^{-1}$) overnight with shaking at 37 °C. The gene expression culture was inoculated with the preculture (2/100) and grown in LB medium containing kanamycin (50 µg $mL^{-1}$) with shacking at 37 °C until $OD_{600}$ = 0.4 – 0.6 was reached. After cooling the culture to 18 °C, enzyme expression was induced by the addition of aqueous IPTG solution (400 mM, 1/1000). The culture was shaken at 18 °C overnight. The cells were harvested via centrifugation (10000 g, 5 min, 4 °C), resuspended in binding buffer (10 mL $L^{-1}$ culture; 20 mM $Na_2HPO_4$, 500 mM NaCl, 20 mM imidazole, 1 mM $MgCl_2$, pH 7.4, 4 °C) and lysed by ultrasonication (8 x 1 min) on ice. The cell debris was removed by centrifugation (14600 g, 10 min, 4 °C) and the soluble protein fraction was loaded onto $Ni^{2+}$-NTA superflow affinity chromatography columns (Qiagen, Venlo, Netherlands) equilibrated with binding buffer.

The column was washed with washing buffer (2 x 10 mL $L^{-1}$ culture; 20 mM $Na_2HPO_4$, 500 mM NaCl, 50 mM imidazole, 1 mM $MgCl_2$, pH 7.4, 4 °C) and the desired protein was eluted with elution buffer (2 x 6.25 mL $L^{-1}$ culture; 20 mM $Na_2HPO_4$, 500 mM NaCl, 500 mM imidazole, 1 mM $MgCl_2$, pH 7.4, 4 °C). The GFPPS protein was acquired in the same way as reported before.[17] The protein concentrations were determined by Bradford assay.[39]

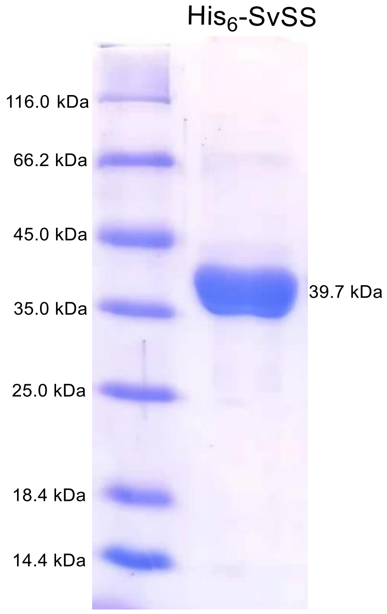


**Figure S2.** SDS-PAGE analysis of recombinant SvSS.

**Enzyme incubation of GFPP with wildtype SvSS and compound purification**

For incubations with GFPP, purified SvSS (0.2 mL; ca. 2.6 mg/mL), a solution of GFPP (0.2 mL; 1.0 mg/mL in 25 mM $NH_4HCO_3$) and incubation buffer (0.5 mL; 50 mM TRIS, 10 mM $MgCl_2$, 10% glycerol, pH 8.2) were mixed and incubated at 28 °C overnight. The enzyme products were extracted with hexane (0.2 mL), and the obtained extract was dried with $MgSO_4$ and analysed by GC/MS.

For preparative scale incubation, GFPP (50 mg, 87.8 µmol) in $NH_4HCO_3$ (25 mM, 25 mL) and an enzyme preparation of SvSS (25 mL; from 8 L expression culture, 2.6 mg $mL^{-1}$) were added to incubation buffer (150 mL). A solution of (2-hydroxypropyl)-β-cyclodextrin (β-CD; 13.3 mL, 160 mM β-CD, 0.2% SDS, 10% glycerol, 10 mM $MgCl_2$, pH = 8.2) was added. The reaction mixture was stirred overnight at 28 °C and then extracted *n*-hexane (3 x 200 mL). The combined extracts were dried with $MgSO_4$ and concentrated in vacuo.

The crude product was purified via silica gel chromatography. The first fractions with elution of *n*-hexane afforded impure **1**, chromatographed again on silica gel with *n*-hexane to obtain pure **1** (1.0 mg, 2.9 µmol, 3.3%).

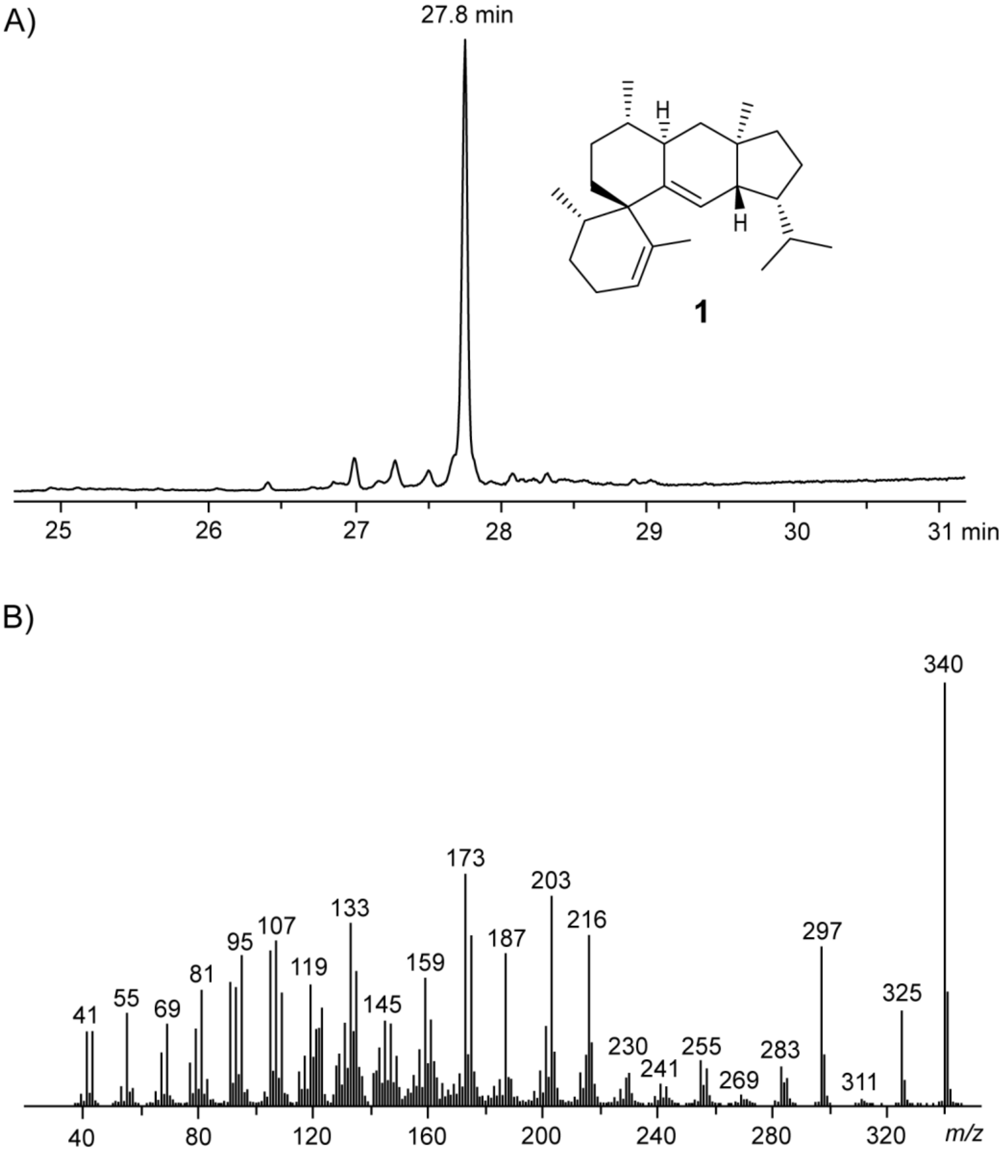


**Figure S3.** Sesterviolene synthase (SvSS). A) Total ion chromatogram of an extract from an incubation experiment of GFPP with SvSS. B) EI mass spectrum of the main compound sesterviolene A (**1**) eluting at 27.8 min.

**Sesterviolene A (1).** Yield: 1.0 mg (2.9 µmol, 3.3%), from 50 mg (87.8 µmol) GFPP triammonium salt. TLC (100% *n*-hexane): $R_f$ = 0.8. GC (HP5-MS): *I* = 2442. MS (EI, 70 eV): *m*/*z* (%) = 340 (100), 325 (31), 297 (40), 283 (27), 257 (14), 216 (56), 203 (66), 187 (54), 175 (68), 173 (75), 159 (40), 157 (41), 145 (39), 143 (41), 133 (60), 135 (46), 119 (50), 107 (67), 105 (83), 95 (73), 91 (73), 81 (46), 67 (31), 55 (51), 43 (69), 41 (61); conf. Figure S3B. IR (diamond ATR): ṽ = 2924 (s), 2855 (s), 1659 (w), 1633 (w), 1463 (m), 1375 (w), 1261 (w), 1099 (m), 1019 (m), 801 (m), 696 (w), 543 (w) $cm^{-1}$. HR-MS (Q-TOF, 70 eV): calc. $[C_{25}H_{40}]^{+\bullet}$ *m*/*z* = 340.3125; found: *m*/*z* = 340.3123. Optical rotary power: $[\alpha]_D^{20}$ = +18.0 (*c* 0.10, $CH_2Cl_2$). NMR data are given in Table S2.

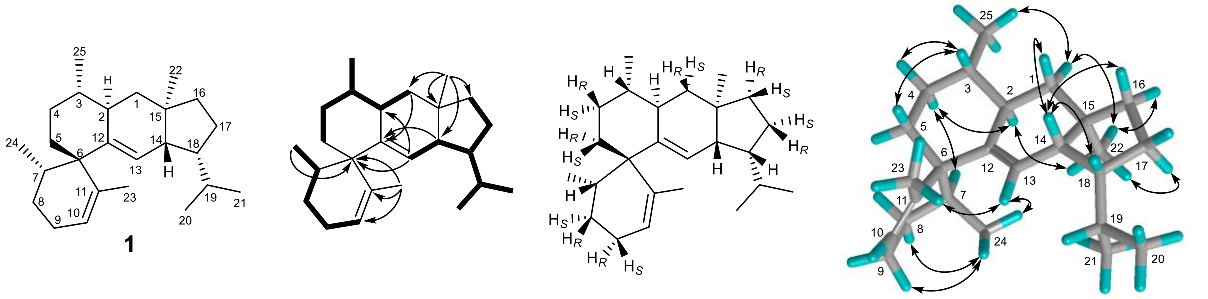

**Figure S4.** Structure elucidation of **1**. Bold: $^{1}H,^{1}H$-COSY, single headed arrows: key HMBC, and double headed arrows: NOESY correlations. Carbon numbering follows GFPP numbering to indicate the origin of each carbon. Diastereotopic hydrogens are labelled $H_R$ (*pro-R*) and $H_S$ (*pro-S*).

**Table S2.** NMR data of sesterviolene A (**1**) in $C_6D_6$ recorded at 298 K.

| C[a] | type | $^{1}H$[b] | $^{13}C$[b] |
|---|---|---|---|
| 1 | $CH_2$ | 2.00 (dd, *J* = 12.1, 6.1, $H_S$)<br>1.01 (m, $H_R$) | 45.74 |
| 2 | CH | 1.73 (m) | 41.22 |
| 3 | CH | 1.13 (m) | 39.36 |
| 4 | $CH_2$ | 1.44 (m, $H_R$)<br>1.34 (m, $H_S$) | 31.98 |
| 5 | $CH_2$ | 1.75 (m, $H_S$)<br>1.70 (m, $H_R$) | 35.02 |
| 6 | $C_q$ | – | 49.46 |
| 7 | CH | 2.06 (m) | 31.81 |
| 8 | $CH_2$ | 1.74 (m, $H_R$)<br>1.39 (m, $H_S$) | 26.28 |
| 9 | $CH_2$ | 2.14 (m, $H_R$)<br>1.82 (m, $H_S$) | 21.85 |
| 10 | CH | 5.60 (ddt, *J* = 5.6, 2.4, 1.1) | 125.33 |
| 11 | $C_q$ | – | 139.12 |
| 12 | $C_q$ | – | 137.42 |
| 13 | CH | 5.82 (t, *J* = 2.2) | 126.50 |
| 14 | CH | 2.34 (ddd, *J* = 9.4, 4.1, 2.2) | 51.17 |
| 15 | $C_q$ | – | 41.18 |
| 16 | $CH_2$ | 1.51 (dd, *J* = 11.3, 8.0, $H_R$)<br>1.13 (m, $H_S$) | 40.04 |
| 17 | $CH_2$ | 1.86 (m, $H_R$)<br>1.62 (m, $H_S$) | 28.50 |
| 18 | CH | 1.79 (m) | 46.61 |
| 19 | CH | 1.67 (m) | 32.37 |
| 20 | $CH_3$ | 1.07 (d, *J* = 6.5) | 24.25 |
| 21 | $CH_3$ | 0.85 (d, *J* = 6.6) | 22.48 |
| 22 | $CH_3$ | 0.93 (s) | 20.73 |
| 23 | $CH_3$ | 1.84 (dt, *J* = 2.3, 1.3) | 21.03 |
| 24 | $CH_3$ | 1.03 (d, *J* = 7.0) | 17.75 |
| 25 | $CH_3$ | 0.90 (d, *J* = 6.4) | 20.47 |

[a] Carbon numbering as shown in Figure S4. [b] Chemical shifts δ in ppm, multiplicity: s = singlet, d = doublet, t = triplet, m = multiplet, coupling constants *J* are given in Hertz.

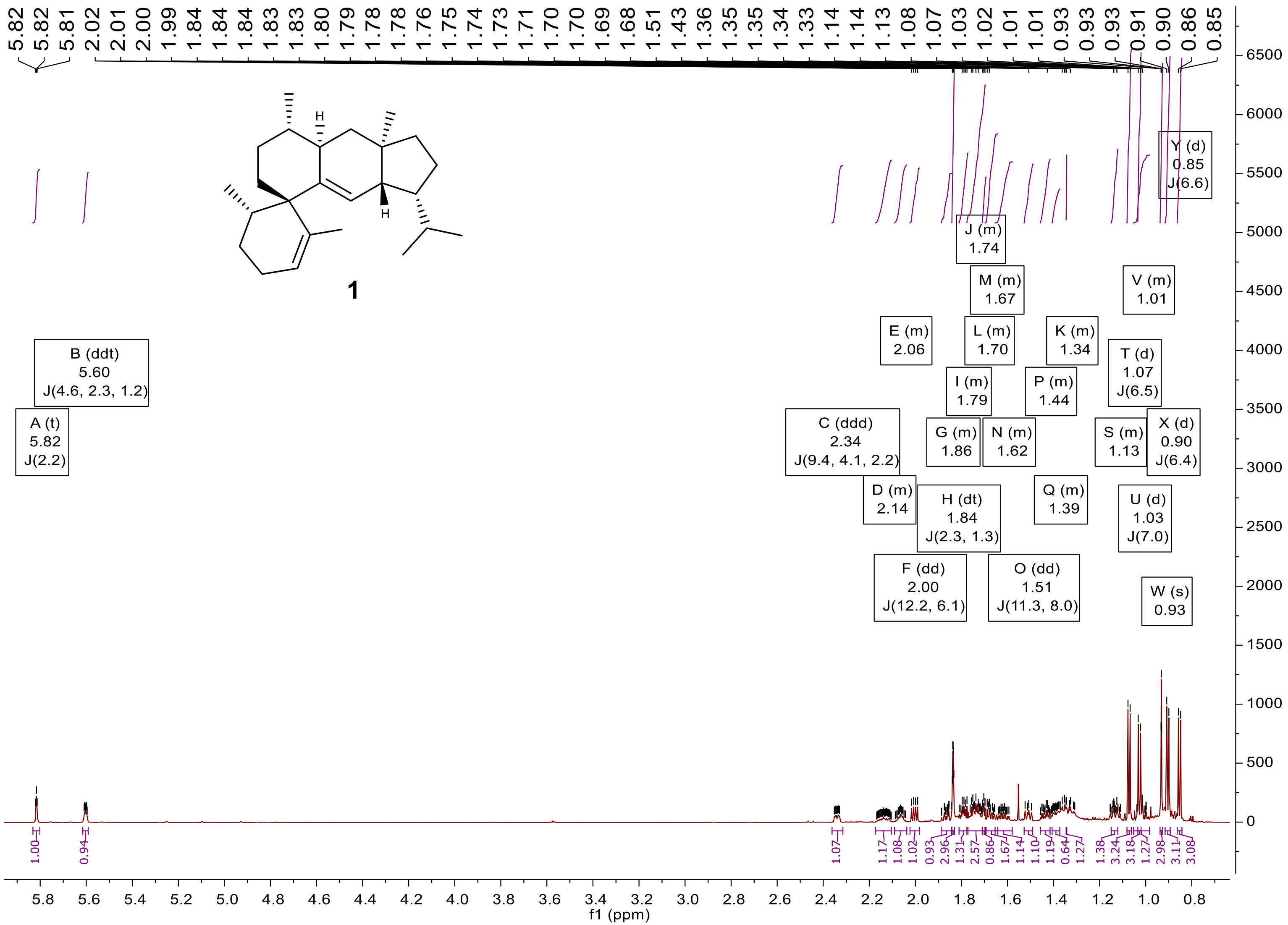

**Figure S5.** $^{1}$H-NMR spectrum of **1** (700 MHz, $C_6D_6$).

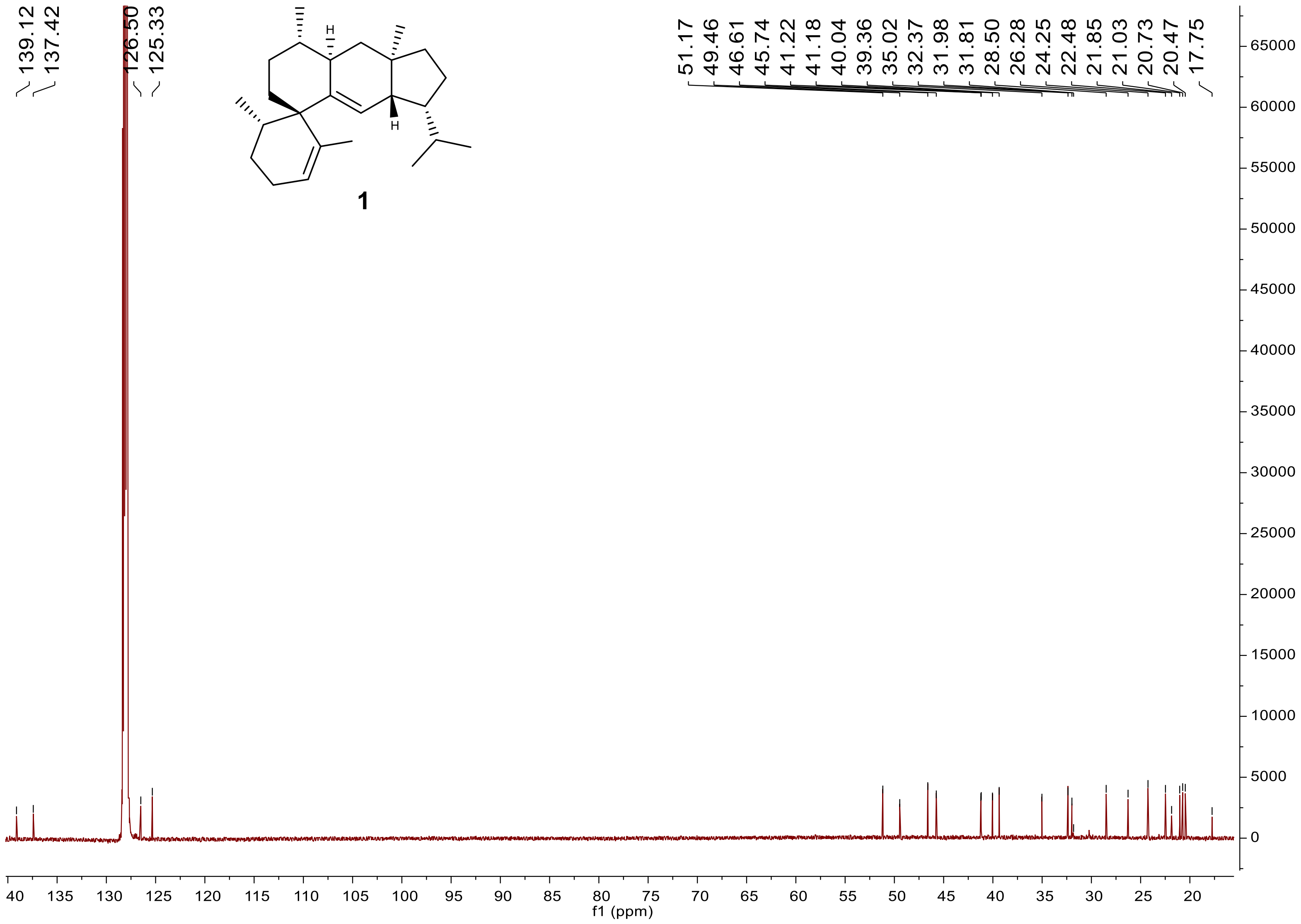


**Figure S6.** $^{13}$C-NMR spectrum of **1** (176 MHz, $C_6D_6$).

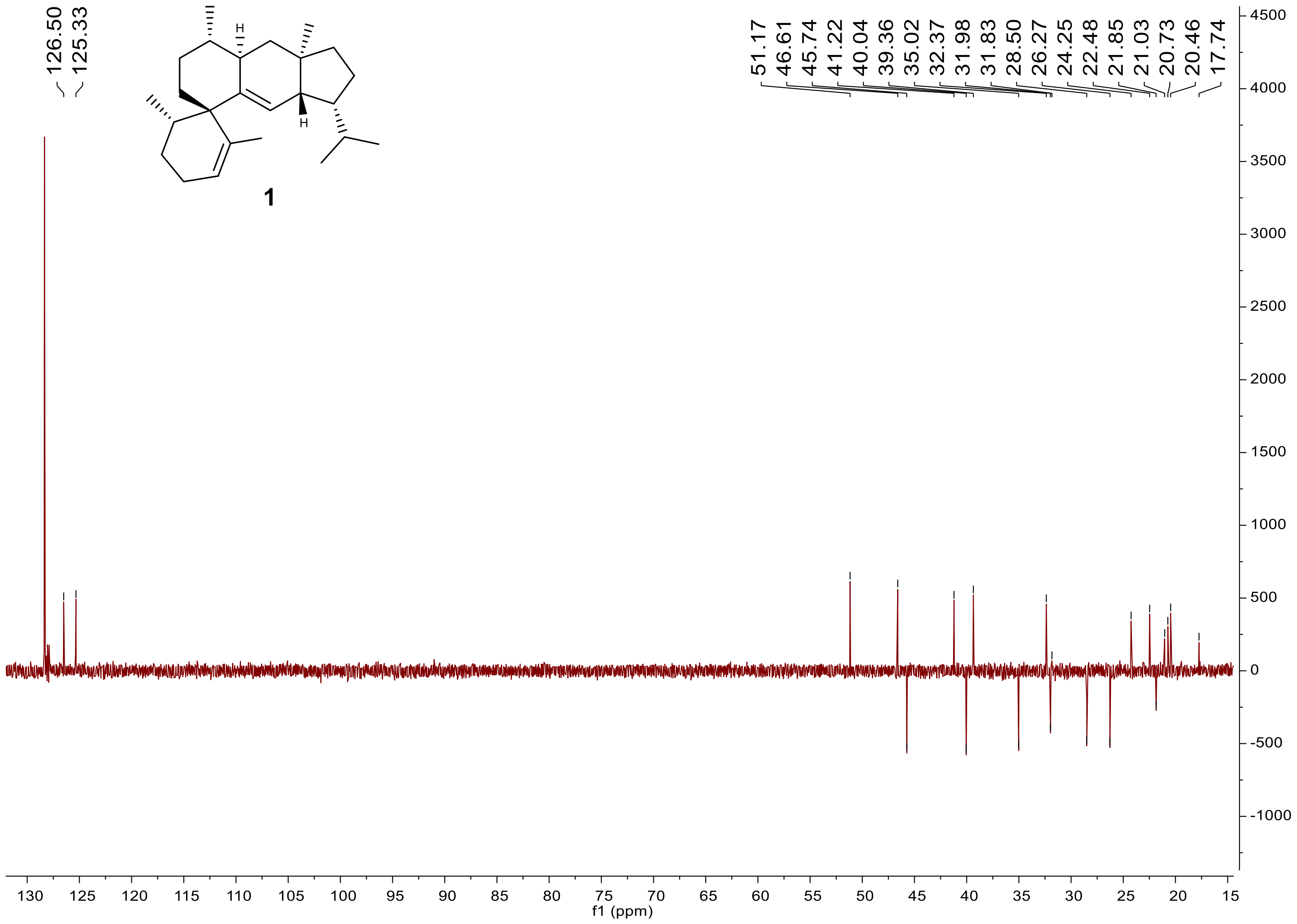


**Figure S7.** $^{13}$C-DEPT135 spectrum of **1** (176 MHz, $C_6D_6$).

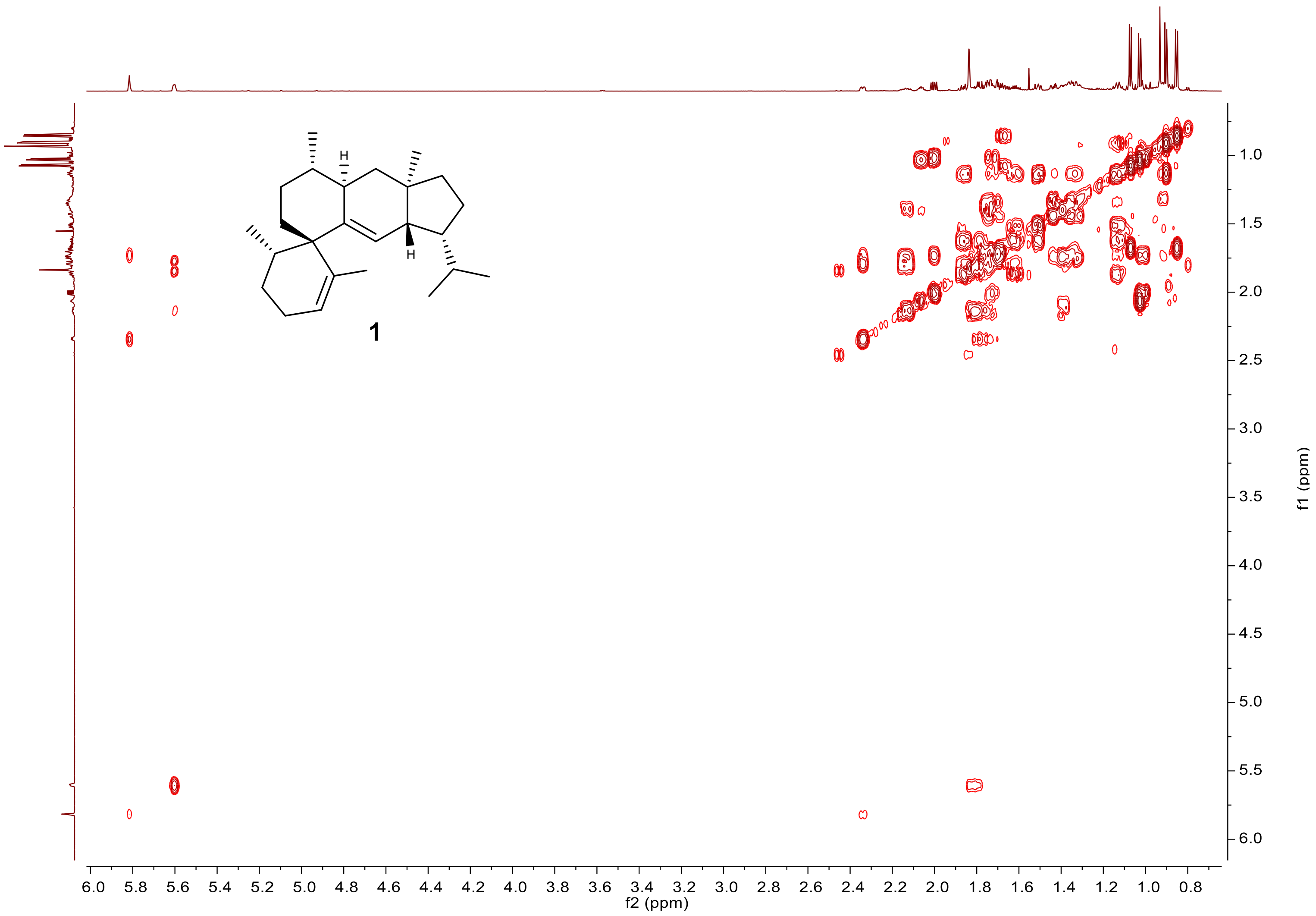


**Figure S8.** $^{1}H$-$^{1}H$-COSY spectrum ($C_6D_6$) of **1**.

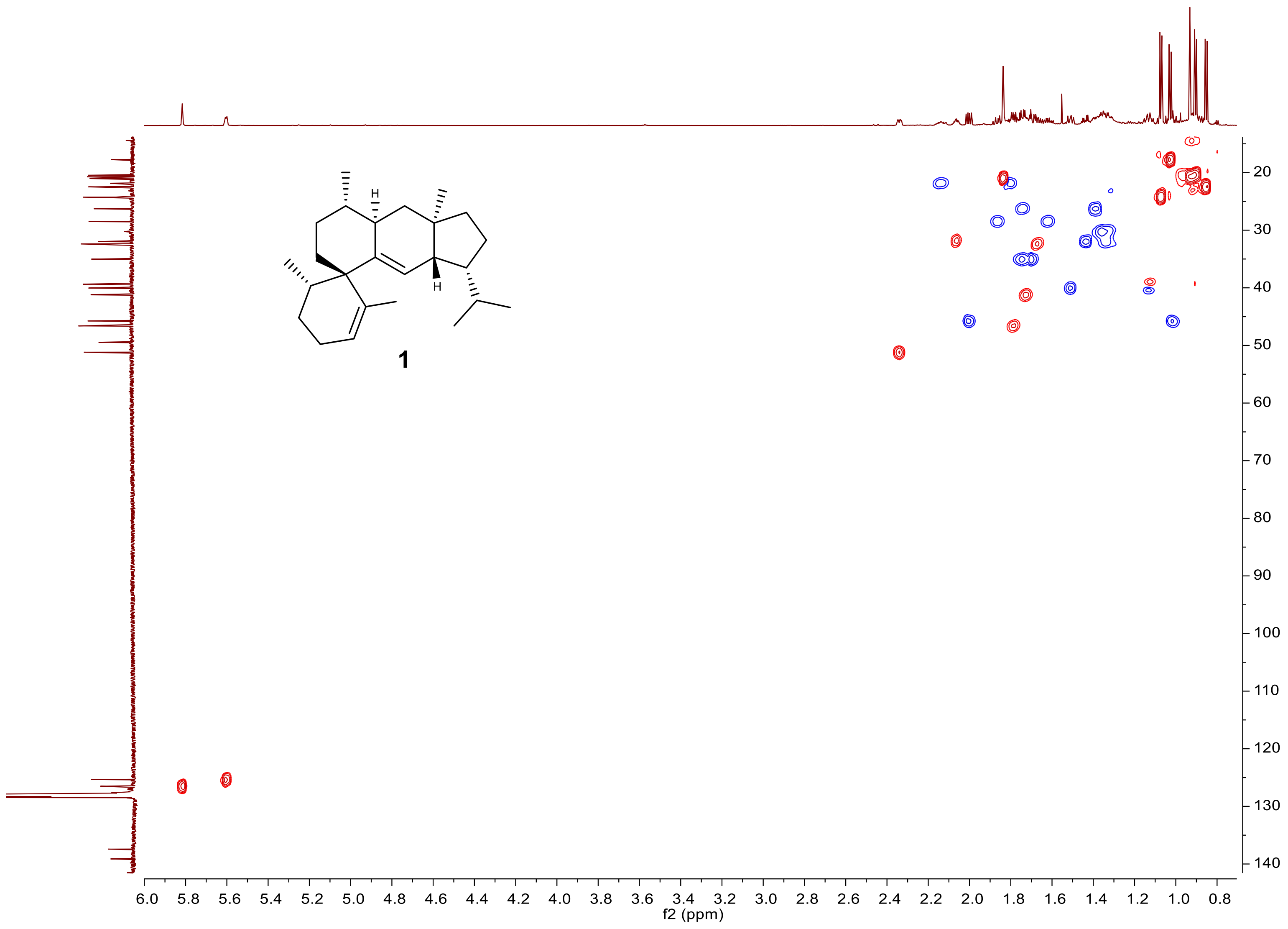


**Figure S9.** HSQC spectrum ($C_6D_6$) of **1**.

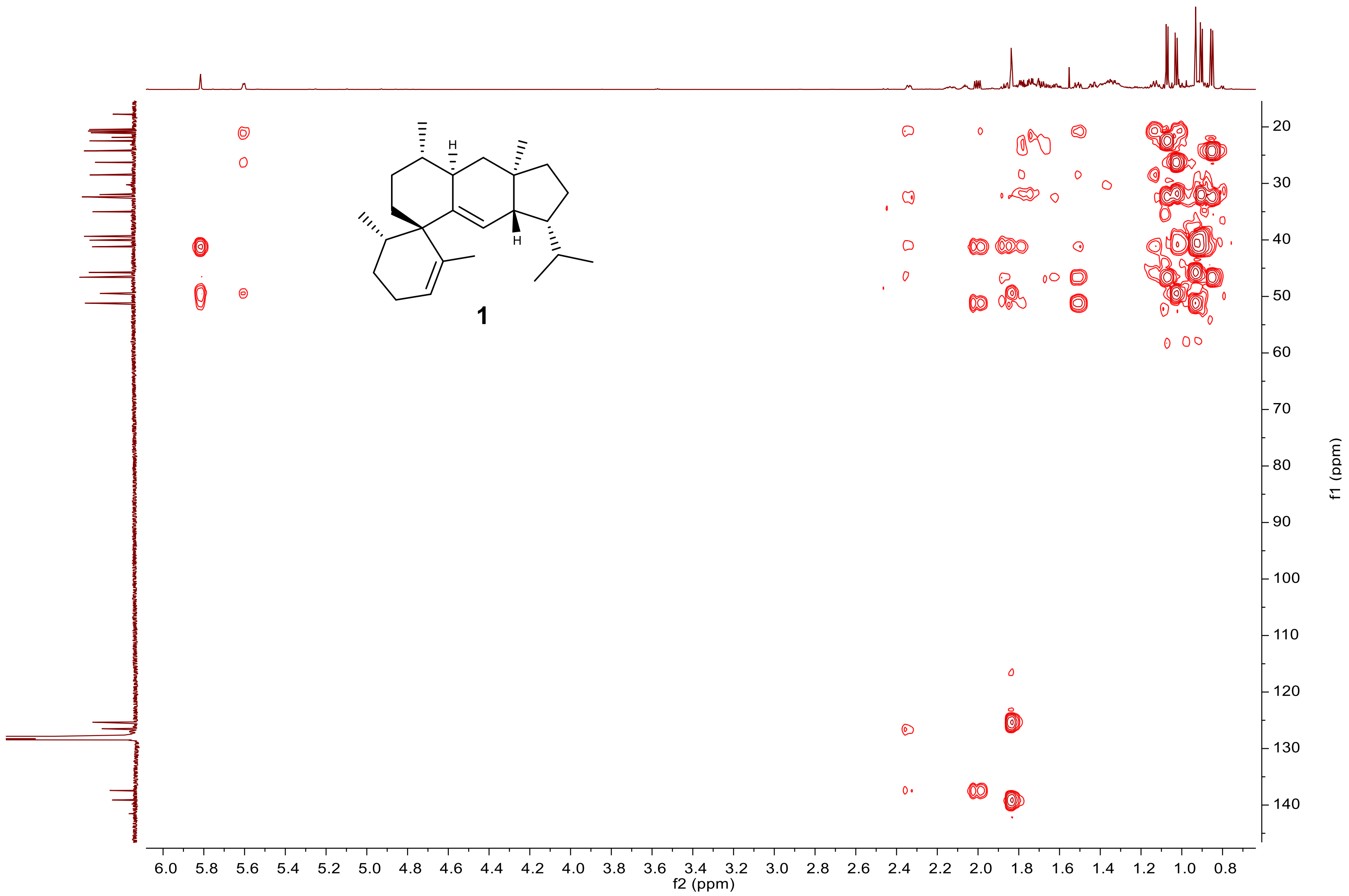


**Figure S10.** HMBC spectrum ($C_6D_6$) of **1**.

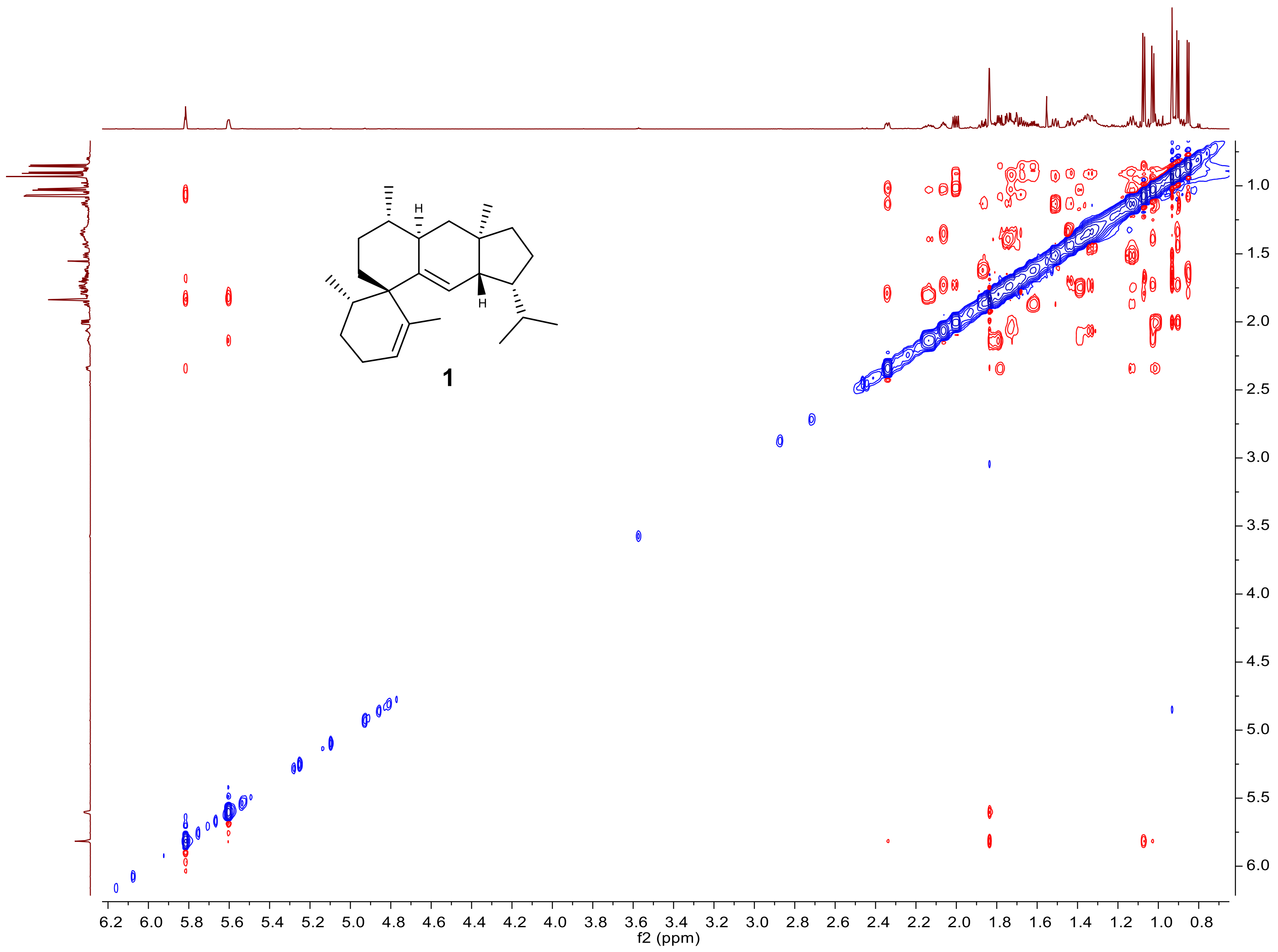


**Figure S11.** NOESY spectrum ($C_6D_6$) of **1**.

**Incubation experiments with labelled substrates**

Isotopic labelling experiments were performed with the precursors of GFPP (ca. 1.5 mg, in 1 mL 25 mM $NH_4HCO_3$), incubation buffer (5 mL) and preparations of purified enzymes (each 1 mL) as listed in Table S3. After incubation at 28 °C overnight, the products were extracted with $C_6D_6$ (600 µL + 300 µL) or *n*-hexane (500 µL), then the extracts were dried with $MgSO_4$ and analysed by NMR and/or GC/MS.

**Table S3.** Labelling experiments with SvSS.

| entry | substrates | enzymes | results shown in |
|---|---|---|---|
| 1 | GGPP + (1-$^{13}$C)IPP[13] | GFPPS,[17] SvSS | Figure S12 |
| 2 | GGPP + (2-$^{13}$C)IPP[40] | GFPPS, SvSS | Figure S12 |
| 3 | GGPP + (3-$^{13}$C)IPP[13] | GFPPS, SvSS | Figure S12 |
| 4 | GGPP + (4-$^{13}$C)IPP[13] | GFPPS, SvSS | Figure S12 |
| 5 | (1-$^{13}$C)GGPP[41] + IPP | GFPPS, SvSS | Figure S12 |
| 6 | (2-$^{13}$C)GGPP[13] + IPP | GFPPS, SvSS | Figure S12 |
| 7 | (3-$^{13}$C)GGPP[41] + IPP | GFPPS, SvSS | Figure S12 |
| 8 | (4-$^{13}$C)GGPP[41] + IPP | GFPPS, SvSS | Figure S12 |
| 9 | (1-$^{13}$C)FPP[16] + IPP | GFPPS, SvSS | Figure S12 |
| 10 | (2-$^{13}$C)FPP[16] + IPP | GFPPS, SvSS | Figure S12 |
| 11 | (3-$^{13}$C)FPP[16] + IPP | GFPPS, SvSS | Figure S12 |
| 12 | (4-$^{13}$C)FPP[16] + IPP | GFPPS, SvSS | Figure S12 |
| 13 | (1-$^{13}$C)GPP[42] + IPP | GFPPS, SvSS | Figure S12 |
| 14 | (6-$^{13}$C)FPP[16] + IPP | GFPPS, SvSS | Figure S12 |
| 15 | (7-$^{13}$C)FPP[16] + IPP | GFPPS, SvSS | Figure S12 |
| 16 | (8-$^{13}$C)FPP[16] + IPP | GFPPS, SvSS | Figure S12 |
| 17 | (9-$^{13}$C)FPP[16] + IPP | GFPPS, SvSS | Figure S12 |
| 18 | (10-$^{13}$C)FPP[16] + IPP | GFPPS, SvSS | Figure S12 |
| 19 | (11-$^{13}$C)FPP[16] + IPP | GFPPS, SvSS | Figure S12 |
| 20 | (12-$^{13}$C)FPP[16] + IPP | GFPPS, SvSS | Figure S12 |
| 21 | (9-$^{13}$C)GPP[43] + IPP | GFPPS, SvSS | Figure S12 |
| 22 | (10-$^{13}$C)GPP[44] + IPP | GFPPS, SvSS | Figure S12 |
| 23 | (15-$^{13}$C)FPP[16] + IPP | GFPPS, SvSS | Figure S12 |
| 24 | (20-$^{13}$C)GGPP[13] + IPP | GFPPS, SvSS | Figure S12 |
| 25 | GGPP + (5-$^{13}$C)IPP[17] | GFPPS, SvSS | Figure S12 |
| 26 | (7-$^{13}$C)GPP[44] + (*E*)-(4-$^{13}$C,4-$^{2}$H)IPP[12] | GFPPS, SvSS | Figure S13 |
| 27 | (7-$^{13}$C)GPP[44] + (*Z*)-(4-$^{13}$C,4-$^{2}$H)IPP[12] | GFPPS, SvSS | Figure S13 |
| 28 | (3-$^{13}$C,2-$^{2}$H)GGPP[13] + IPP | GFPPS, SvSS | Figure S14 |
| 29 | GPP + (4,4-$^{2}H_2$)IPP[44] + (3-$^{13}$C)IPP[13] | GFPPS, SvSS | Figure S15 |
| 30 | (*S*)-(1-$^{2}$H)GPP + IPP[45] | GFPPS, SvSS | Figure S16 |
| 31 | (*R*)-(1-$^{2}$H)GPP + IPP[45] | GFPPS, SvSS | Figure S16 |
| 32 | DMAPP + (*S*)-(1-$^{13}$C,1-$^{2}$H)IPP[29] | GFPPS, SvSS | Figure S17 |
| 33 | DMAPP + (*R*)-(1-$^{13}$C,1-$^{2}$H)IPP[29] | GFPPS, SvSS | Figure S17 |
| 34 | DMAPP + (*E*)-(4-$^{13}$C,4-$^{2}$H)IPP[12] | GFPPS, SvSS | Figure S18 |
| 35 | DMAPP + (*Z*)-(4-$^{13}$C,4-$^{2}$H)IPP[12] | GFPPS, SvSS | Figure S18 |
| 36 | (2-2H)GPP[43] + (4-$^{13}$C)IPP[13] | GFPPS, SvSS | Figures S71, S72 |

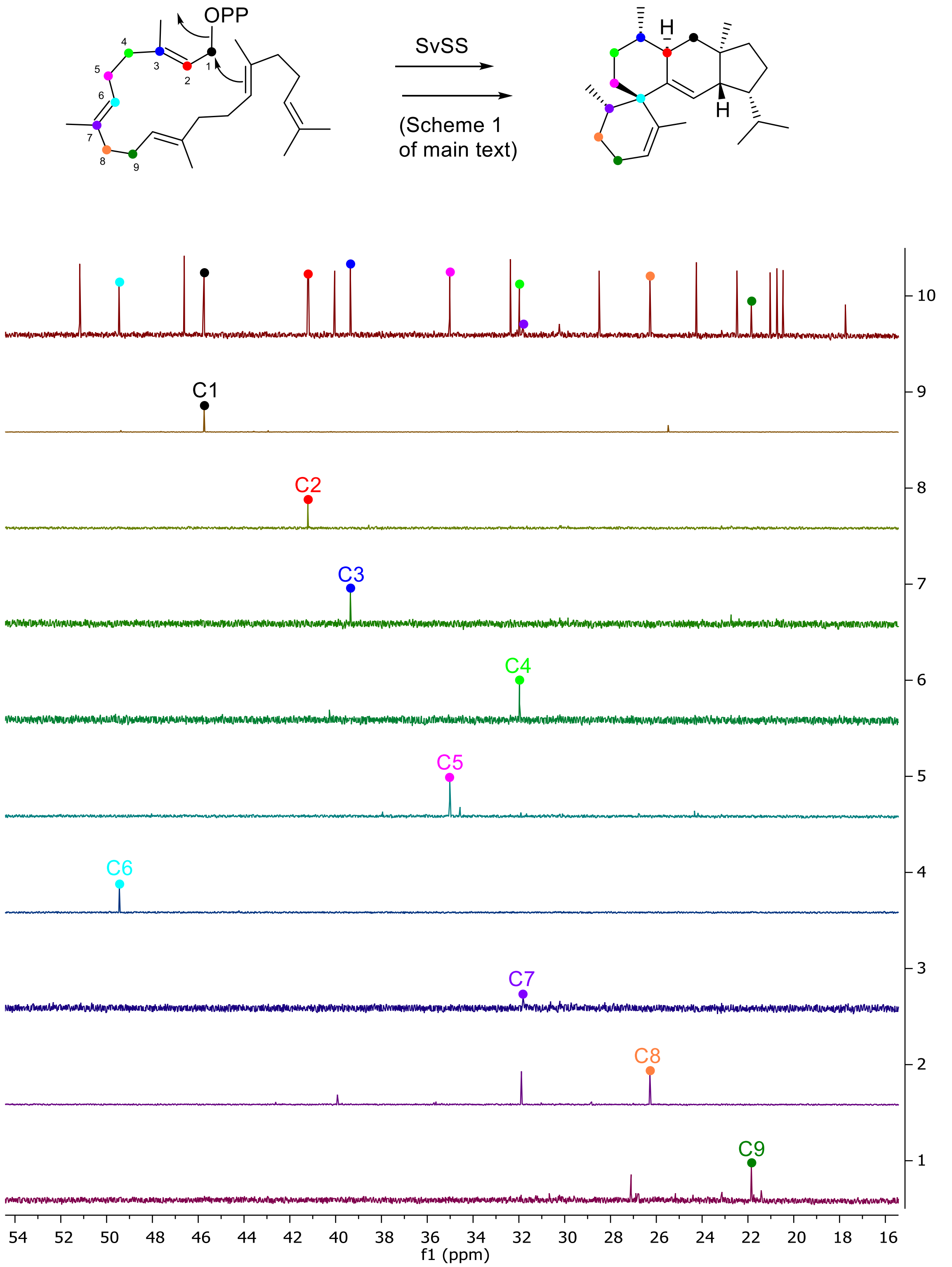


**Figure S12.** $^{13}$C-NMR spectra of the products obtained from all 25 isotopomers of ($^{13}C_1$)GFPP obtained by chemical and enzymatic synthesis. The coloured dots show the site of incorporation into **1** and indicate the corresponding signal in the $^{13}$C-NMR spectra.

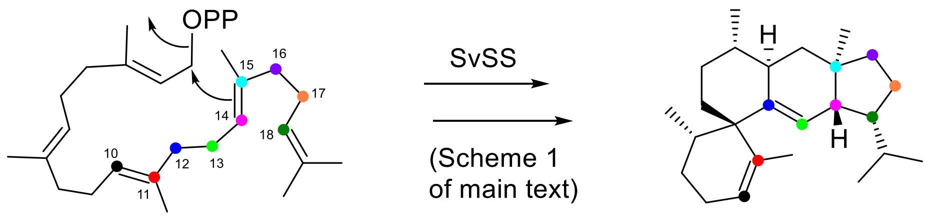


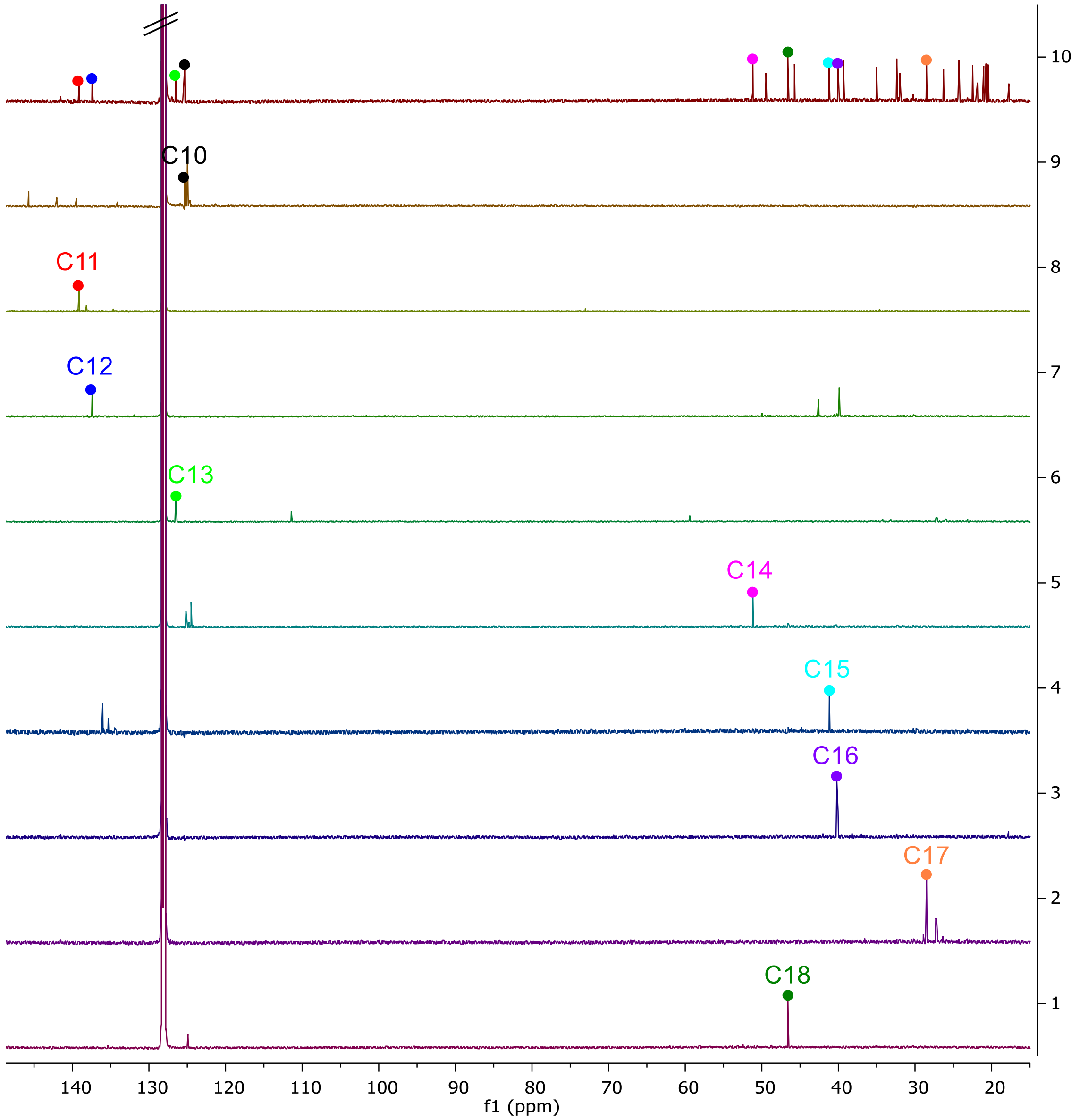


**Figure S12 (continued).** $^{13}C$-NMR spectra of the products obtained from all 25 isotopomers of ($^{13}C_1$)GFPP obtained by chemical and enzymatic synthesis. The coloured dots show the site of incorporation into **1** and indicate the corresponding signal in the $^{13}C$-NMR spectra.

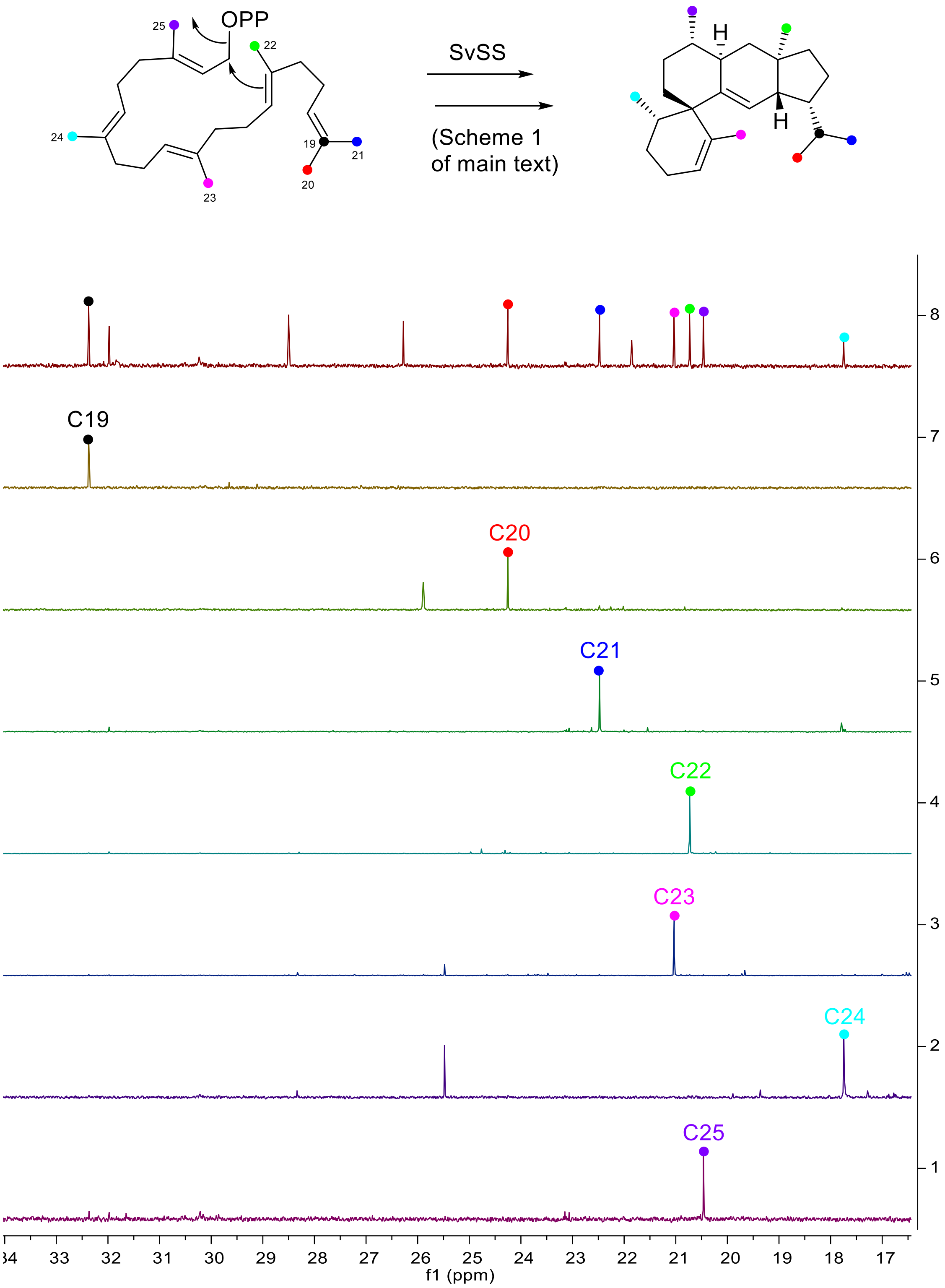


**Figure S12 (continued).** $^{13}$C-NMR spectra of the products obtained from all 25 isotopomers of ($^{13}C_1$)GFPP obtained by chemical and enzymatic synthesis. The coloured dots show the site of incorporation into **1** and indicate the corresponding signal in the $^{13}$C-NMR spectra.

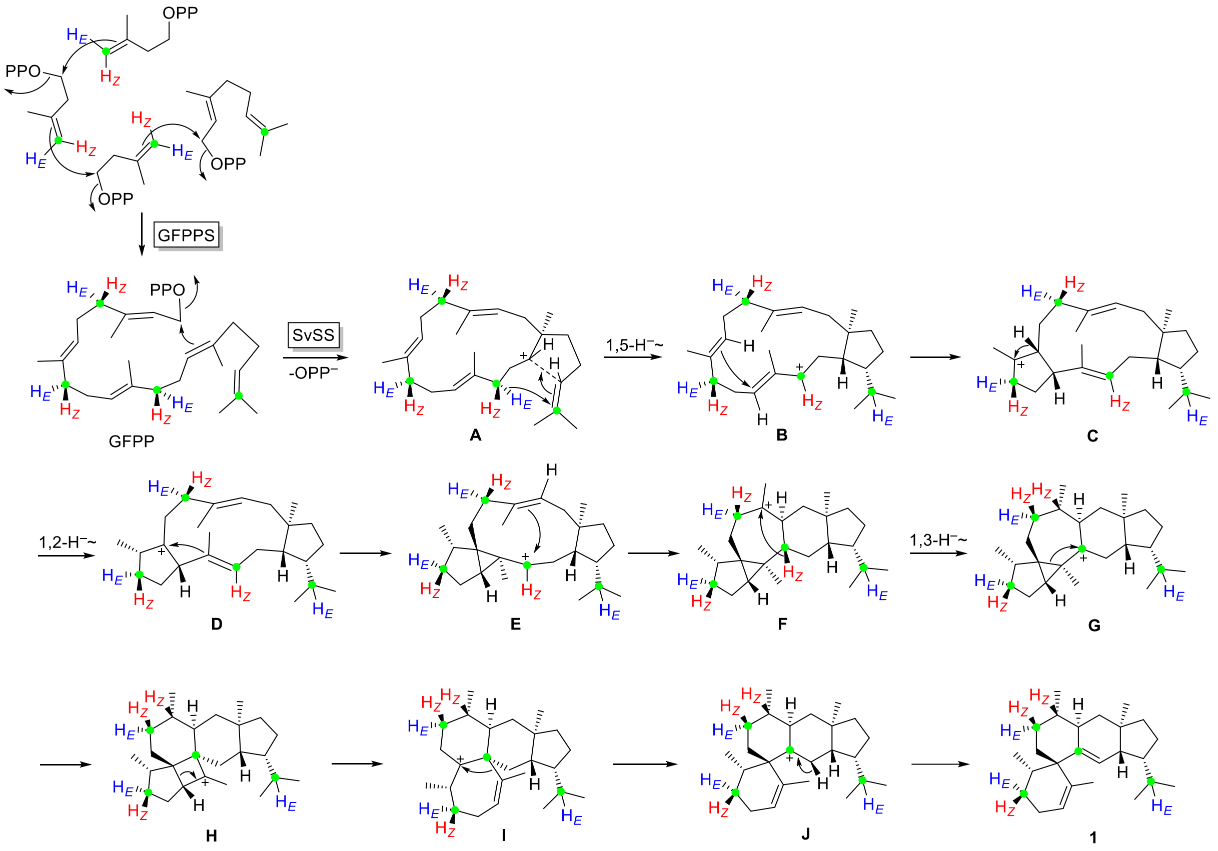


**Scheme S1.** Biosynthesis of labelled **1** from (7-$^{13}$C)GPP and (*E*)- or (*Z*)-(4-$^{13}$C,4-$^{2}$H)IPP with GFPPS and SvSS.

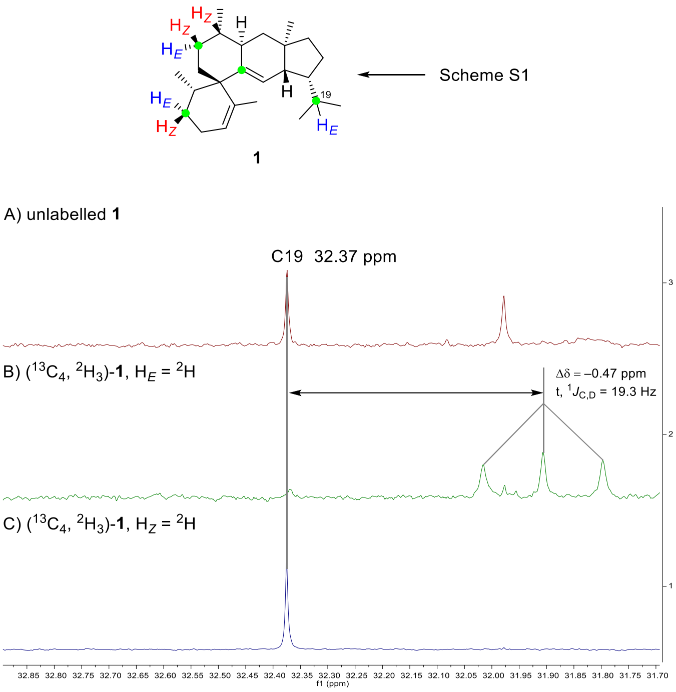


**Figure S13.** 1,5-Hydride shift from **A** to **B** in the formation of compound **1**. A) $^{13}$C-NMR signal for C19 of unlabelled **1**, B) $^{13}$C-NMR signal for deuterated C19 of labelled **1** obtained from (7-$^{13}$C)GPP and (*E*)-(4-$^{13}$C,4-$^{2}$H)IPP with GFPPS and SvSS, C) $^{13}$C-NMR signal for non-deuterated C19 of labelled **1** obtained from (7-$^{13}$C)GPP and (*Z*)-(4-$^{13}$C,4-$^{2}$H)IPP with GFPPS and SvSS. The slightly upfield shifted triplet in B) is indicative for a direct $^{13}$C-$^{2}$H bond and supports the proposed 1,5-hydride shift.

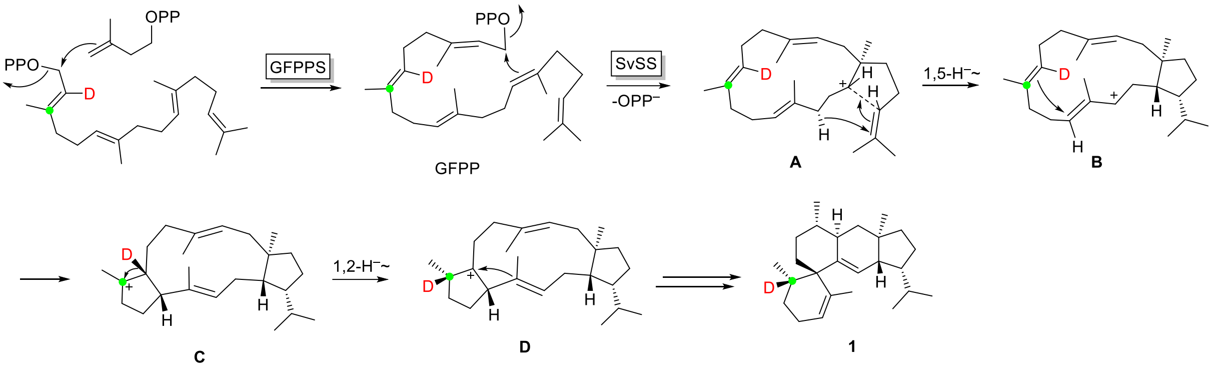


**Scheme S2.** Biosynthesis of labelled **1** from (3-$^{13}$C,2-$^{2}$H)GGPP and IPP with GFPPS and SvSS.

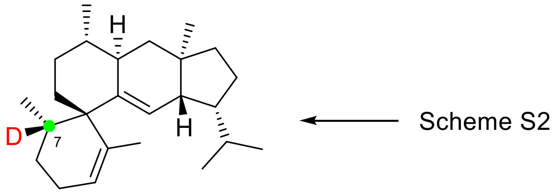


**1**

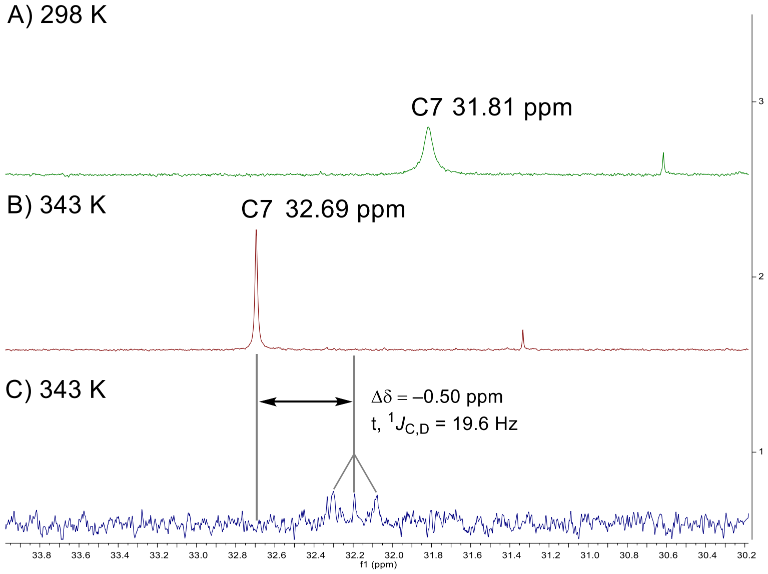


**Figure S14.** 1,2-Hydride shift from **C** to **D** in the formation of compound **1**. A) $^{13}$C-NMR signal for (7-$^{13}$C)-**1** at 298 K. B) $^{13}$C-NMR signal for (7-$^{13}$C)-**1** at 343 K. C) $^{13}$C-NMR signal for deuterated C7 of labelled **1** at 343 K obtained from (3-$^{13}$C,2-$^{2}$H)GGPP and IPP with GFPPS and SvSS. The slightly upfield shifted triplet in C) is indicative for a direct $^{13}$C-$^{2}$H bond and supports the proposed 1,2-hydride shift from **C** to **D** (Scheme S2). This triplet cannot be observed at 298 K due to peak broadening.

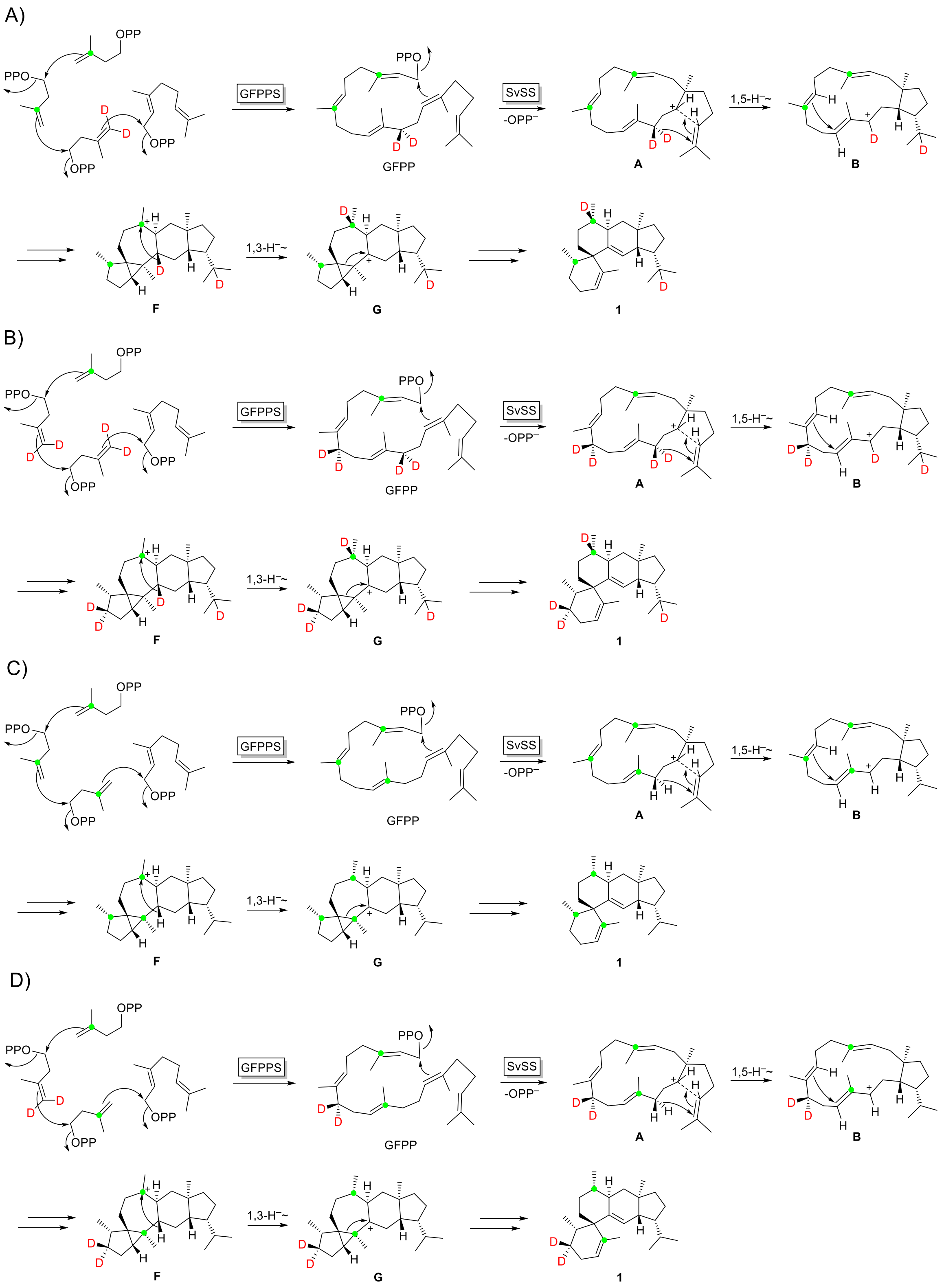


**Scheme S3.** Biosynthesis of labelled **1** from GPP, (4,4-$^2H_2$)IPP, and (3-$^{13}C$)IPP with GFPPS and SvSS. This reaction produces a mixture of eight isotopomers, four of which are shown, in which the last unit of GFPP is derived from (3-$^{13}C$)IPP. Specifically, these isotopomers are: A) (3-$^{13}C$,7-$^{13}C$,12,12-$^2H_2$)GFPP, B) (3-$^{13}C$,8,8-$^2H_2$,12,12-$^2H_2$)GFPP, C) (3-$^{13}C$,7-$^{13}C$,11-$^{13}C$)GFPP, and D) (3-$^{13}C$, 8,8-$^2H_2$,11-$^{13}C$)GFPP. The 1,3-hydride shift from **F** to **G** becomes visible if the third isoprene unit in GFPP is derived from (4,4-$^2H_2$)IPP and the last unit is derived from (3-$^{13}C$)IPP (Scheme S3A and S3B).

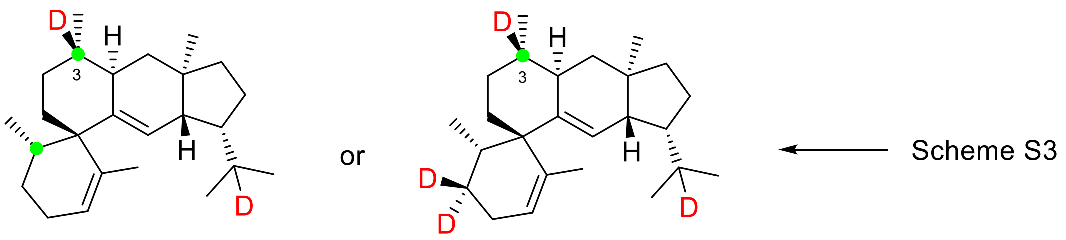


**1** **1**

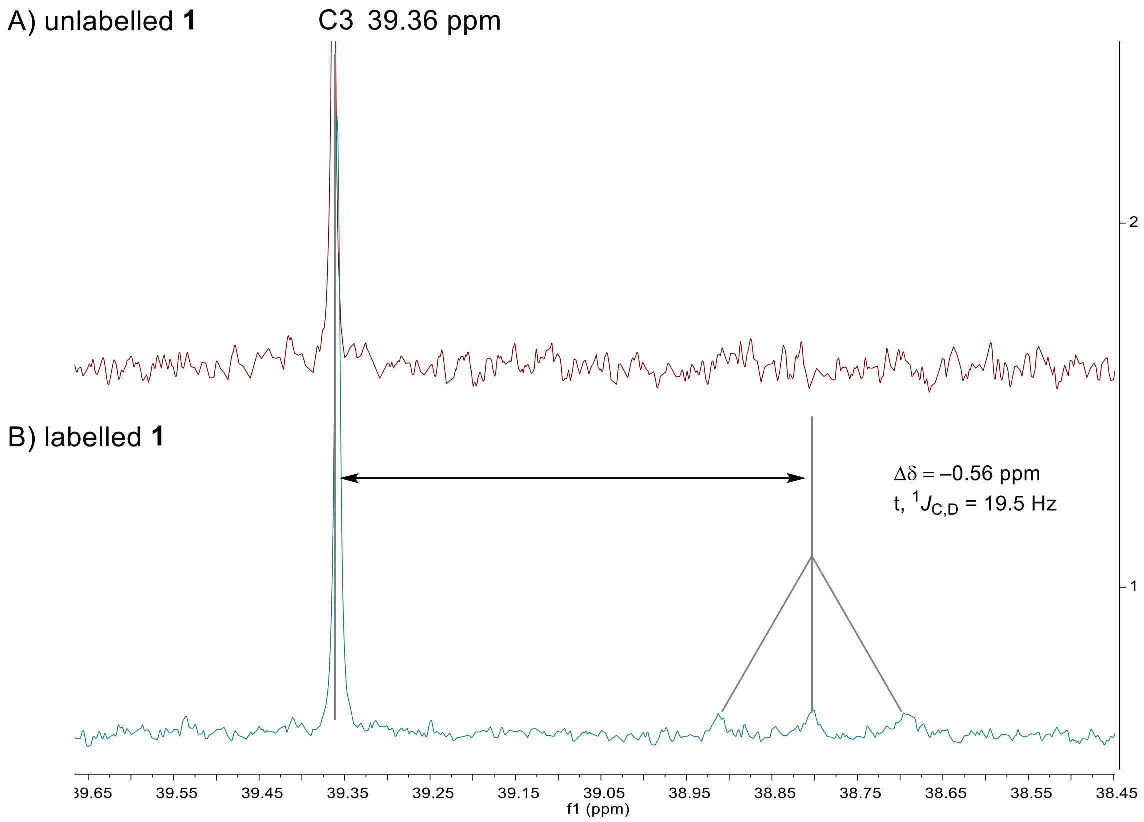


**Figure S15.** 1,3-Hydride shift from **F** to **G** in the formation of compound **1**. A) $^{13}$C-NMR signal for C3 of unlabelled **1**, B) $^{13}$C-NMR signal for deuterated C3 of labelled **1** obtained from GPP, (4,4-$^{2}H_{2}$)IPP, and (3-$^{13}$C)IPP with GFPPS and SvSS. The slightly upfield shifted triplet in B) is indicative for a direct $^{13}$C-$^{2}$H bond and supports the proposed 1,3-hydride shift.

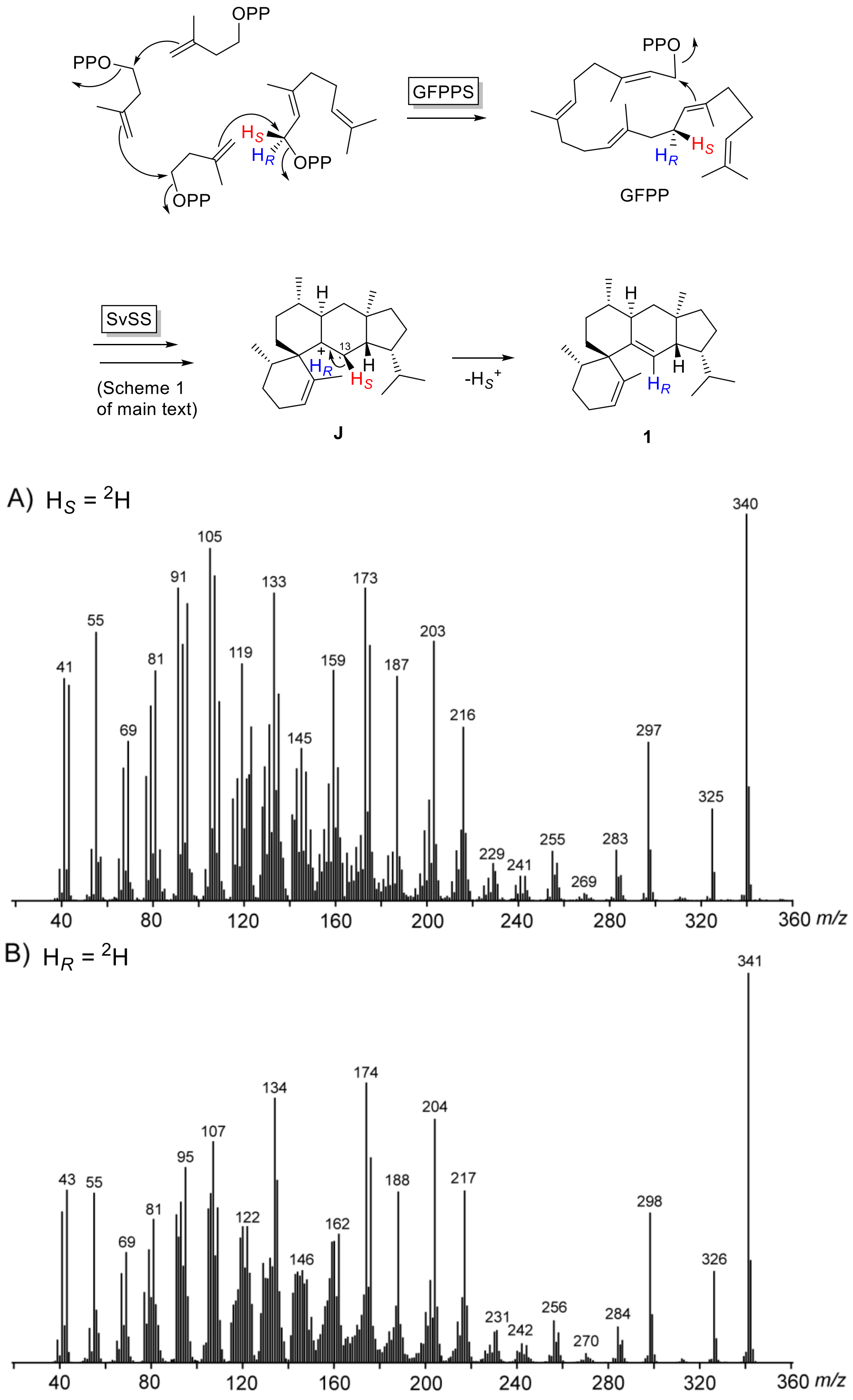


**Figure S16.** Stereochemical course of the terminal deprotonation step to **1**. A) (*S*)-(1-$^{2}$H)GPP or B) (*R*)-(1-$^{2}$H)GPP is enzymatically elongated with three units of IPP. Subsequent cyclisation of both resulting enantiomers of (*S*)-(13-$^{2}$H)GFPP and (*R*)-(13-$^{2}$H)GFPP shows specific loss of $H_S$ from GPP by product analysis through GC/MS.

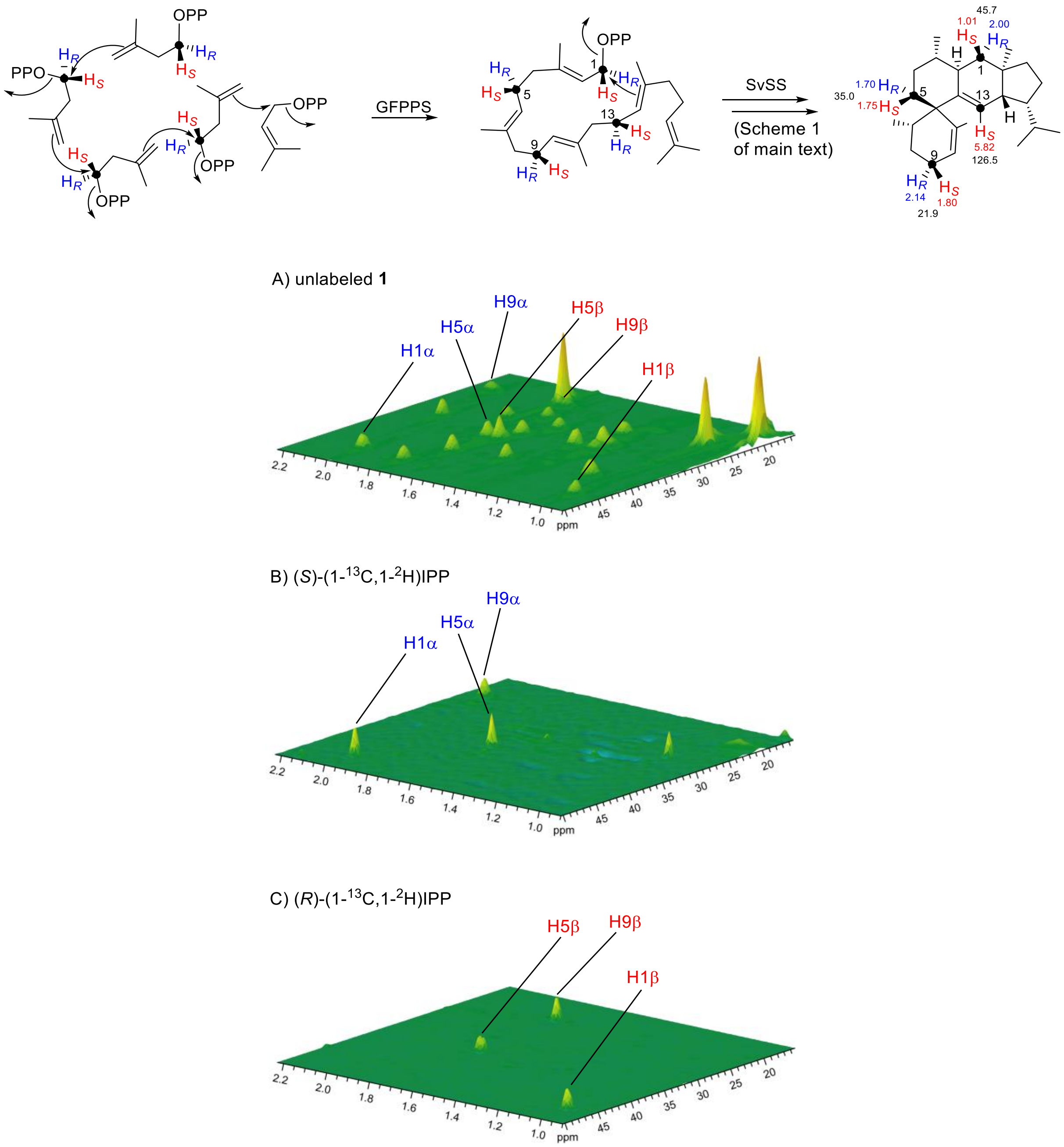


**Figure S17.** The absolute configuration of **1**. Partial HSQC spectra of A) unlabelled **1**, B) labelled **1** obtained from DMAPP and (*S*)-(1-$^{13}$C, 1-$^{2}$H)IPP (red H = $^{2}$H), and C) labelled **1** from DMAPP and (*R*)-(1-$^{13}$C, 1-$^{2}$H)IPP (blue H = $^{2}$H). The specific incorporation at C1, C5, and C9 with known configuration at these carbons in experiments B) and C) together with the NOESY based assignments of relative orientations of H1α, H1β, H5α, H5β, H9α, and H9β (Figure S4) with respect to the naturally present stereogenic centers in **1** allows to assign the shown absolute configuration for **1**. Black dots represent $^{13}$C-labelled carbons.

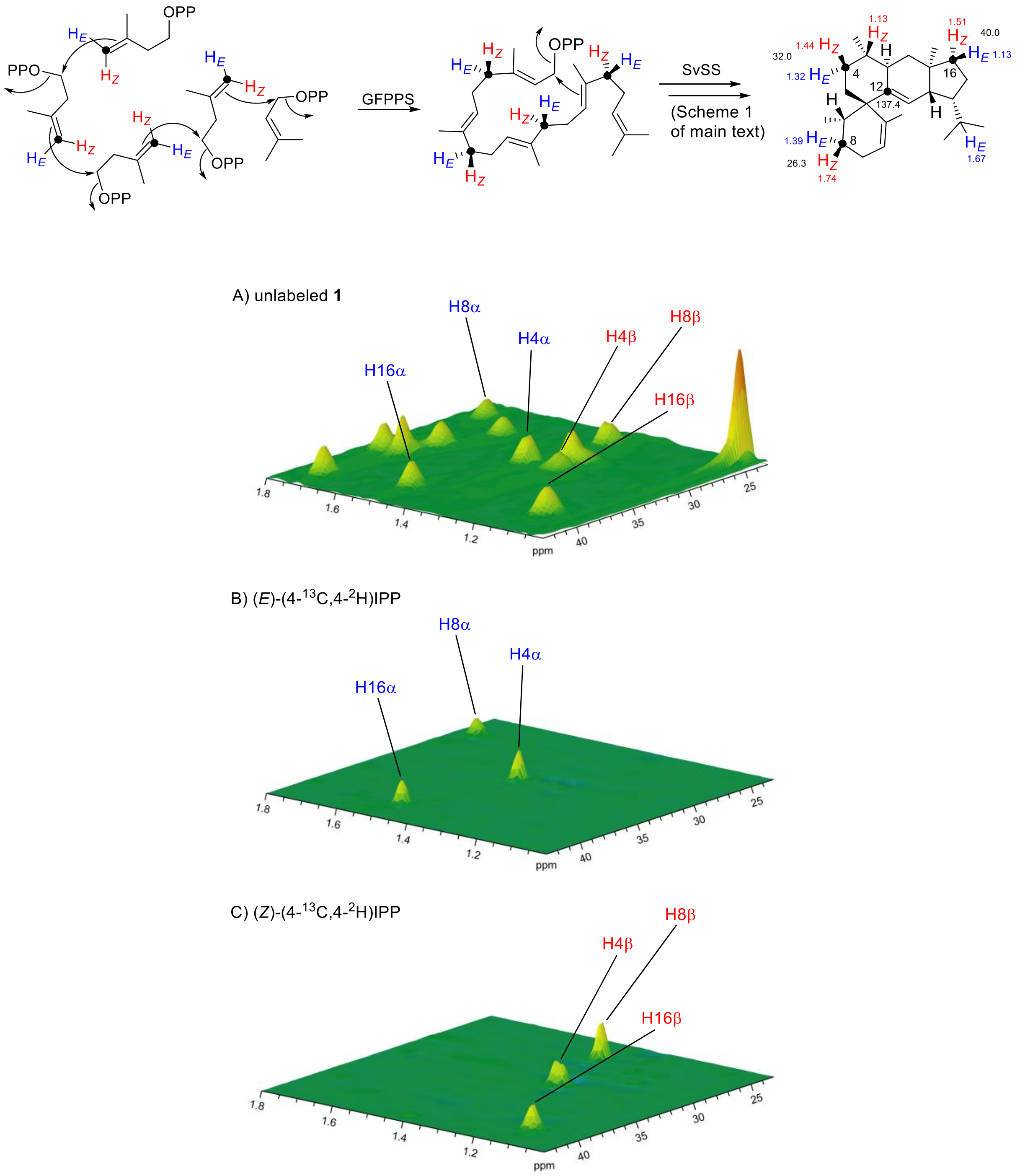


**Figure S18.** The absolute configuration of **1**. Partial HSQC spectra of A) unlabelled **1**, B) labelled **1** obtained from DMAPP and (*E*)-(4-$^{13}$C, 4-$^{2}$H)IPP (blue H = $^{2}$H), and C) labelled **1** from DMAPP and (*Z*)-(4-$^{13}$C, 4-$^{2}$H)IPP (red H = $^{2}$H). The specific incorporation at C4, C8, and C16 with known configuration at these carbons in experiments B) and C) together with the NOESY based assignments of relative orientations of H4α, H4β, H8α, H8β, H16α, and H16β (Figure S4) with respect to the naturally present stereogenic centers in **1** allows to assign the shown absolute configuration for **1**. Black dots represent $^{13}$C-labelled carbons.

```
Consensus MXPXXXXPXXFXXXXXPJXZXXXXXXXXJXXXXAAWAZXFXJGXXXZXXXXLXXXXXXTXXXXXXPXXXXXXXXXXXLADXXXW
SdS       MEPELTVPPLF----SPIRQAIHPKHADIDVQTAAWAETFRIG-SEELRGKLVTQDIGTFSARILPE--GREEVVSLLADFILW
SvSS      MHP-ADLPEGFWTFYCPLEEETGADAERLSANSAAWAQKFDLGRGDQDLSALYGVGGATLITHVFPHATANPDLAQALADYSGW
                                                                     *   **            *   *

Consensus XFXXDDXXXXXXXXXXRXXDXXXXXXRXXRXXXXPXXXXXQXXXLXXXLRBXXXRXXXXXXXXQXXRXXXXXXXXXXXXXXWEXA
SdS       LFGVDDGHCEEGELGHRPGDLAGLLHRLIRVAQNPEAPMMQDDPLAAGLRDLRMRVDRFGTAGQTARWVDALREYFFSVVWEAA
SvSS      AFMADDFIVPDPDA--R-ADVLHAVYRWARIMLCPRSWENQGTHLDDALRNALERLRACMSDVQYERFTTTQADWLHAMLWERA
          **

Consensus XXXXGTXXXXNDYXXXRLXXGAXXVXXXPXXLXXXXGXELXXXEXXXXXVXAXAEXXXFXXXXDNDXXSXXKERXXXXXXXXNXX
SdS       HRRAGTVPDLNDYTLMRLYDGATSV-VLP-MLEMGHGYELQPYERDRTAVRAVAEMASFIITWDNDIFSYHKERRGSGYYLNAL
SvSS      LSERGTALTVNDYLAVRL--GAVGVHATPGYLDAVEGTELTAQEWSSPLVKAAAEAGLFAAALDNDRYSFCKERDLAQINYNLF
                          *     ***                                *  **

Consensus XXLZXEXXXXTXXQAXXXXIXXRDXXXXXXXXXXXQJXXXXSPXLRXYLXXXXXXIXGXXXXGXXXXRYXXPXXXXXXXXXXTX
SdS       RVLEQER-GLTPAQALDAAISQRDRVMCLFTTVSEQLAEQGSPQLRQYLHSLRCFIRGAQDWGISSVRYTTPDDPANMPSVFTD
SvSS      GALQYEHPDWTLEQAMLEGITIRDTMLACYLRLRDQILPTASPDLRKYLTGVERVISGDITFGTTCMRYFAPEATPEVRRTRTP
                                                                *   *  *

Consensus XPXXXXXEPLXXPXVXWWWDXJAXDARSVRRQVPAQRSA
SdS       VPTDDSTEPLDIPAVSWWWDLLAEDARSVRRQVPAQRSA
SvSS      -PAHLTDEPLPYPTVAWWWDHIAS---------------
```

**Figure S19.** Amino acid sequence alignment of *Streptomyces pristinaespiralis* selinadiene synthase (SdS) and *Streptomyces violarus* sesterviolene synthase (SvSS). Highly conserved motifs that are important for binding of the substrate and the $Mg^{2+}$ cofactor (Asp-rich motif, pyrophosphate sensor, NSE triad and RY pair) are shown in purple. Hydrophobic residues contouring the active site in SdS and the corresponding residues in SvSS are shown in cyan. Active site residues of SvSS identified by protein homology modelling are shown in green. Asterisks indicate positions targeted by site-directed mutagenesis.

## Site-directed mutagenesis

The single mutations were performed by the overlap extension PCR (OE-PCR) method.[46] The expression plasmid that contained the wildtype gene was used as a template, and the Q5® High-Fidelity DNA polymerase was purchased from NEB (Ipswich, Massachusetts, USA). The primers used for PCR were designed to carry suitable mutations to obtain the desired amino acid exchanges (Table S4) and were used to amplify the SvSS gene in two overlapping fragments. These first-round PCRs were performed using the following temperature program: 1) 98 °C for 30 s; 2) 98 °C for 10 s, 68 °C for 30 s, 72 °C for 40 s; repeated 35 times; 3) 72 °C for 2 min. In the second-round PCR the two fragments were mixed. Amplification was achieved through the following process. Step 1: 1) 98 °C for 30 s; 2) 98 °C for 10 s, 70 °C for 30 s, 72 °C for 40 s; repeated 5 times; 3) 72 °C for 2 min. Step 2: addition of primers 35G3 and 35G4 for amplification of the whole *SvSS* gene carrying additional homology arms for cloning into the expression vector pYE-Express by homologous recombination. The PCR was then continued: 1) 98 °C for 30 s; 2) 98 °C for 10 s, 68 °C for 30 s, 72 °C for 40 s; repeated 35 times; 3) 72 °C for 2 min. The mutated gene was then analysed by gel electrophoresis and purified by the Wizard SV Gel and PCR Clean-Up System (Promega, Madison, Wisconsin, USA).
The mutated genes were incorporated into the pET28 based expression vector pYE-Express by homologous recombination in yeast using the standard PEG/LiOAc/salmon sperm protocol.[30,38] *Saccharomyces cerevisiae* cultures containing the plasmids were grown on SM-URA plates for 3 days. The colonies were collected to isolate the plasmid mixture by using the Zymoprep Yeast Plasmid Miniprep II kit (Zymo Research, Irvine, CA, USA). The isolated plasmids were introduced into *E. coli* BL21(DE3) electrocompetent cells by electroporation. Cells were plated on LB medium and grown over night. Single colonies were picked and used to inoculate 3 mL LB cultures (kanamycin). After incubation at 37 °C for 12 h, the plasmids

were extracted by the PureYield Plasmid Miniprep System (Promega) and checked by sequencing. The transformants containing the correct mutations were used for protein expression.

**Table S4.** Primers used for site-directed mutagenesis of SvSS.

| Enzyme variant | Primer | Nucleotide sequence (5‘ → 3‘)[a] |
|---|---|---|
| SvSS wildtype | 35G3 | **GGCAGCCATATGGCTAGCATGACTGGTGGA**atgcaccctgccgacctccc |
| | 35G4 | **TCTCAGTGGTGGTGGTGGTGGTGCTCGAGTG**ttatgatgcgatgtgatcccaccaccaggc |
| L59F | | |
| Fragment 1 | 35G5 | atgcaccctgccgacctccc |
| | 38I3 | ggaagacatgggtgatAAAggtggcccctcccacc |
| Fragment 2 | 38I2 | agtgggaggggccaccTTTatcacccatgtcttccc |
| | 36G6 | ttatgatgcgatgtgatcccaccaccaggc |
| V63A | | |
| Fragment 1 | 35G5 | atgcaccctgccgacctccc |
| | 38I5 | ggtggcgtgggggaaCGCatgggtgatcagggtg |
| Fragment 2 | 38I4 | ccctgatcacccatGCGttcccccacgccaccg |
| | 36G6 | ttatgatgcgatgtgatcccaccaccaggc |
| L77A | | |
| Fragment 1 | 35G5 | atgcaccctgccgacctccc |
| | 38I7 | gctgtagtcggcCGCcgcctgggccaggtc |
| Fragment 2 | 38I6 | cctggcccaggcgGCGgccgactacagcg |
| | 36G6 | ttatgatgcgatgtgatcccaccaccaggc |
| S81T | | |
| Fragment 1 | 35G5 | atgcaccctgccgacctccc |
| | 38I9 | gaacgcccagccGGTgtagtcggc |
| Fragment 2 | 38I8 | gctggccgactacACCggctgggcgttcatg |
| | 36G6 | ttatgatgcgatgtgatcccaccaccaggc |
| A84G | | |
| Fragment 1 | 35G5 | atgcaccctgccgacctccc |
| | 39A2 | gtcgtcggccatgaaGCCccagccgctgtagtcg |
| Fragment 2 | 39A1 | ccgactacagcggctggGGCttcatggccgacgac |
| | 36G6 | ttatgatgcgatgtgatcccaccaccaggc |
| F85L | | |
| Fragment 1 | 35G5 | atgcaccctgccgacctccc |
| | 39A4 | gtcgtcggccatCAGcgcccagccgc |
| Fragment 2 | 39A3 | ctacagcggctgggcgCTGatggccgacgacttc |
| | 36G6 | ttatgatgcgatgtgatcccaccaccaggc |
| V185A | | |
| Fragment 1 | 35G5 | atgcaccctgccgacctccc |
| | 39A6 | ggcgtggacgccCGCggcgccgagg |
| Fragment 2 | 39A5 | cctcggcgccGCGggcgtccacgc |
| | 36G6 | ttatgatgcgatgtgatcccaccaccaggc |
| G186A | | |
| Fragment 1 | 35G5 | atgcaccctgccgacctccc |
| | 39A8 | cgtggcgtggacCGCgacggcgccgag |
| Fragment 2 | 39A7 | ctcggcgccgtcGCGgtccacgccacg |
| | 36G6 | ttatgatgcgatgtgatcccaccaccaggc |
| V187A | | |
| Fragment 1 | 35G5 | atgcaccctgccgacctccc |
| | 39B1 | gcgtggcgtgCGCgccgacggcgc |
| Fragment 2 | 39A9 | cgccgtcggcGCGcacgccacgcc |
| | 36G6 | ttatgatgcgatgtgatcccaccaccaggc |

| Enzyme variant | Primer | Nucleotide sequence (5‘ → 3‘)[a] |
|---|---|---|
| A223L | | |
| Fragment 1 | 35G5 | atgcaccctgccgacctccc |
| | 39B3 | cggtcgttgtccagagcCAGggcgaagagacccgc |
| Fragment 2 | 39B2 | gcgggtctcttcgccCTGgctctggacaacgaccgc |
| | 36G6 | ttatgatgcgatgtgatcccaccaccaggc |
| A224V | | |
| Fragment 1 | 35G5 | atgcaccctgccgacctccc |
| | 39B5 | gcggtcgttgtccagCACggcggcgaagagac |
| Fragment 2 | 39B4 | ggtctcttcgccgccGTGctggacaacgaccg |
| | 36G6 | ttatgatgcgatgtgatcccaccaccaggc |
| V301W | | |
| Fragment 1 | 35G5 | atgcaccctgccgacctccc |
| | 39B7 | gtcgccgctgatCCAccgctccacgccg |
| Fragment 2 | 39B6 | cggcgtggagcggTGGatcagcggcgacatc |
| | 36G6 | ttatgatgcgatgtgatcccaccaccaggc |
| F64W | | |
| Fragment 1 | 35G5 | atgcaccctgccgacctccc |
| | 40A2 | gcggtggcgtggggCCAgacatgggtgatcagg |
| Fragment 2 | 40A1 | ctgatcacccatgtcTGGccccacgccaccgcg |
| | 36G6 | ttatgatgcgatgtgatcccaccaccaggc |
| F64Y | | |
| Fragment 1 | 35G5 | atgcaccctgccgacctccc |
| | 40A4 | gcggtggcgtggggATAgacatgggtgatcagg |
| Fragment 2 | 40A3 | ctgatcacccatgtcTATccccacgccaccgcg |
| | 36G6 | ttatgatgcgatgtgatcccaccaccaggc |
| F64L | | |
| Fragment 1 | 35G5 | atgcaccctgccgacctccc |
| | 40B1 | cggtggcgtggggCAGgacatgggtgatcagg |
| Fragment 2 | 40A9 | ctgatcacccatgtcCTGccccacgccaccgc |
| | 36G6 | ttatgatgcgatgtgatcccaccaccaggc |
| F64A | | |
| Fragment 1 | 35G5 | atgcaccctgccgacctccc |
| | 40B3 | gcggtggcgtggggCGCgacatgggtgatcaggg |
| Fragment 2 | 40B2 | ccctgatcacccatgtcGCGccccacgccaccgcg |
| | 36G6 | ttatgatgcgatgtgatcccaccaccaggc |
| L220A | | |
| Fragment 1 | 35G5 | atgcaccctgccgacctccc |
| | 40B7 | cagagcggcggcgaaCGCacccgcttcggctg |
| Fragment 2 | 40B6 | cagccgaagcgggtGCGttcgccgccgctctg |
| | 36G6 | ttatgatgcgatgtgatcccaccaccaggc |
| L220F | | |
| Fragment 1 | 35G5 | atgcaccctgccgacctccc |
| | 40B9 | gagcggcggcgaaAAAacccgcttcggctg |
| Fragment 2 | 40B8 | gccgaagcgggtTTTttcgccgccgctc |
| | 36G6 | ttatgatgcgatgtgatcccaccaccaggc |
| D305N | | |
| Fragment 1 | 35G5 | atgcaccctgccgacctccc |
| | 40C2 | ggtaccgaaggtgatGTTgccgctgatcacc |
| Fragment 2 | 40C1 | gggtgatcagcggcAACatcaccttcggtaccac |
| | 36G6 | ttatgatgcgatgtgatcccaccaccaggc |
| D305L | | |
| Fragment 1 | 35G5 | atgcaccctgccgacctccc |
| | 40C4 | ggtggtaccgaaggtgatCAGgccgctgatcacccgc |
| Fragment 2 | 40C3 | gcgggtgatcagcggcCTGatcaccttcggtaccacctg |
| | 36G6 | ttatgatgcgatgtgatcccaccaccaggc |

| Enzyme variant | Primer | Nucleotide sequence (5‘ → 3‘)[a] |
|---|---|---|
| D305A | | |
| Fragment 1 | 35G5 | atgcaccctgccgacctccc |
| | 40C6 | gtggtaccgaaggtgatCGCgccgctgatcacccg |
| Fragment 2 | 40C5 | cgggtgatcagcggcGCGatcaccttcggtaccacc |
| | 36G6 | ttatgatgcgatgtgatcccaccaccaggc |
| D305E | | |
| Fragment 1 | 35G5 | atgcaccctgccgacctccc |
| | 40C8 | ggtaccgaaggtgatTTCgccgctgatcacc |
| Fragment 2 | 40C7 | gggtgatcagcggcGAAatcaccttcggtacc |
| | 36G6 | ttatgatgcgatgtgatcccaccaccaggc |
| F308W | | |
| Fragment 1 | 35G5 | atgcaccctgccgacctccc |
| | 40D1 | gcatgcaggtggtaccCCAggtgatgtcgccgc |
| Fragment 2 | 40C9 | gcggcgacatcaccTGGggtaccacctgcatgcg |
| | 36G6 | ttatgatgcgatgtgatcccaccaccaggc |

[a] Homology arms for gene cloning are shown in bold. Triplet codon exchanges introduced for mutations are underlined.

## Expression and purification of enzyme variants

The transformants harboring the mutated gene were inoculated in 20 mL LB medium with kanamycin 50 µg mL$^{-1}$). The cultures were incubated at 37 °C overnight to form the precultures. Gene expression cultures were inoculated with the 20 mL preculture (2/100) and grown in 500 mL LB medium containing kanamycin (50 µg mL$^{-1}$) with shacking at 37 °C until $OD_{600}$ = 0.4 – 0.6 was reached. After cooling the cultures to 18 °C enzyme expressions were induced by the addition of aqueous IPTG solution (400 mM, 1/1000). The cultures were shaken at 18 °C for 16 h. The cells were harvested via centrifugation (10000 g, 5 min, 4 °C), resuspended in binding buffer (10 mL L$^{-1}$ culture; 20 mM $Na_2HPO_4$, 500 mM NaCl, 20 mM imidazole, 1 mM $MgCl_2$, pH 7.4, 4 °C) and lysed by ultrasonication (8x 1 min) on ice. The cell debris was removed by centrifugation (14600 g, 10 min, 4 °C) and the soluble protein fractions were loaded onto $Ni^{2+}$-NTA superflow affinity chromatography columns (Qiagen, Venlo, Netherlands) equilibrated with binding buffer. The columns were washed with wash buffer (2 x 10 mL L$^{-1}$ culture; 20 mM $Na_2HPO_4$, 500 mM NaCl, 50 mM imidazole, 1 mM $MgCl_2$, pH 7.4, 4 °C) and the desired proteins were eluted with elution buffer (2 x 6.25 mL L$^{-1}$ culture; 20 mM $Na_2HPO_4$, 500 mM NaCl, 500 mM imidazole, 1 mM $MgCl_2$, pH 7.4, 4 °C).

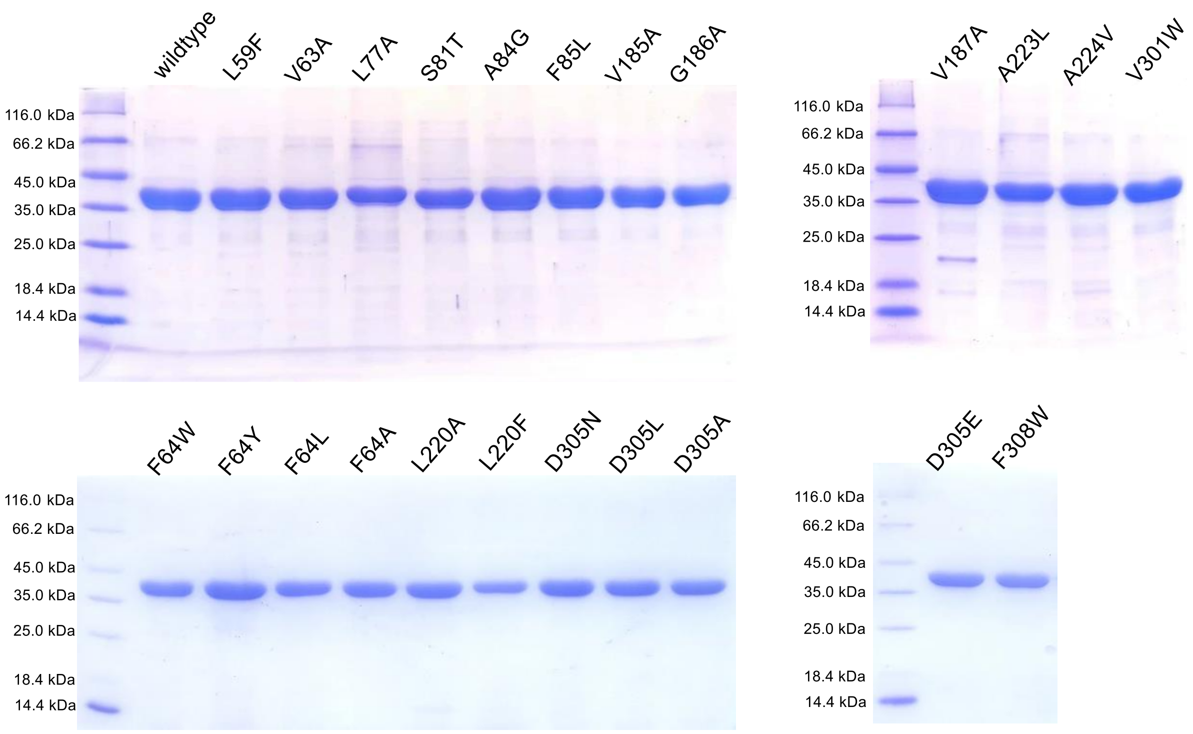


**Figure S20.** SDS-PAGE analysis of the purified SvSS variants.

**Activity of enzyme variants**

The concentration of the purified proteins was determined by the Bradford assay,[39] and then adjusted to 0.8 mg mL$^{-1}$ for all enzyme variants for activity testings. The protein preparation (0.1 mL), incubation buffer (0.8 mL; 50 mM TRIS, 10 mM $MgCl_2$, 10% glycerol, pH 8.2), and GFPP (0.1 mL; 1 mg mL$^{-1}$ in 25 mM aqueous $NH_4HCO_3$) were mixed and incubated at 28 °C overnight. The reaction was extracted with hexane (0.5 mL; containing 0.08 mg mL$^{-1}$ tetradecane as internal standard). The layers were separated by centrifugation (16000 rpm, 5 min). The organic layer was pipetted off and analysed by GC/MS. Triplicates were performed for every enzyme variant.

**Table S5.** Production of compound **1** by wildtype SvSS and its enzyme variants, and relative enzyme activities.

| **Enzyme variant** | **Activity**[a] |
|---|---|
| SvSS (wildtype) | 100±0.3% |
| SvSS (L59F) | 7.5±0.3% |
| SvSS (V63A) | 35±3% |
| SvSS (L77A) | 46±2% |
| SvSS (S81T) | 32±2% |
| SvSS (A84G) | 13±0.4% |
| SvSS (F85L) | 16±2% |
| SvSS (V185A) | 24±1% |
| SvSS (G186A) | inactive |
| SvSS (V187A) | 13±0.2% |
| SvSS (A223L) | inactive |
| SvSS (A224V) | 13±1% |
| SvSS (V301W) | inactive |
| SvSS (F64W) | inactive |
| SvSS (F64Y) | inactive |
| SvSS (F64L) | 18±2% |
| SvSS (F64A) | 5.1±0.6% |
| SvSS (L220A) | 39±1% |
| SvSS (L220F) | 104±16% |
| SvSS (D305N) | 169±26% |
| SvSS (D305L) | inactive |
| SvSS (D305A) | 148±3% |
| SvSS (D305E) | 114±20% |
| SvSS (F308W) | 83±6% |

[a] Production of compound **1** by peak integration of total ion chromatograms from triplicates. Production of **1** by wildtype SvSS is set to 100%. Since **1** is the only major product of wildtype SvSS and all its variants, the enzyme activities were calculated from the production of **1**. Data represent mean and standard deviations from triplicates.

**Enzyme incubation of GFPP with SvSS D305N and compound isolation**

The SvSS D305N variant showed the highest increase in enzyme activity (169±26%) and enzyme yield (SvSS D305N: 29 mg from 1 L expression culture, SvSS wildtype: 8 mg from 1 L expression culture) and was thus selected for preparative scale enzymatic conversions of GFPP to accumulate **1** and isolate the observed SvSS side products. The enzyme variant SvSS D305N was expressed and purified following the same procedure as described above for wildtype SvSS. GFPP (150 mg, 263.4 µmol) in $NH_4HCO_3$ (25 mM, 25 mL) and an enzyme preparation of SvSS (D305N) (25 mL; from 8 L expression culture, 9.4 mg $mL^{-1}$) were added to incubation buffer (150 mL). A solution of (2-hydroxypropyl)-β-cyclodextrin (β-CD; 13.3 mL, 160 mM β-CD, 0.2% SDS, 10% glycerol, 10 mM $MgCl_2$, pH = 8.2) was added. The reaction mixture was stirred overnight at 28 °C and then extracted with *n*-hexane (3 x 200 mL). The combined extracts were dried with $MgSO_4$ and concentrated in vacuo. The crude product mixture was purified via silica gel chromatography. The first fractions with elution of *n*-hexane afforded impure **1**, **3**, and **4**, which were further purified via silica gel with *n*-hexane to obtain pure compounds **1** (20 mg, 58.8 µmol, 22%), **3** (0.7 mg, 2.1 µmol, 0.8%), and **4** (0.6 mg, 1.8 µmol, 0.7%). The next fractions through elution with *n*-hexane afforded pure compound **2** (0.7 mg, 2.1 µmol, 0.8%). Compounds **5** (0.9 mg, 2.6 µmol, 1.0%), **6** (0.8 mg, 2.4 µmol, 0.9%), and **7** (0.3 mg, 0.9 µmol, 0.3%) were obtained via HPLC purification. HPLC purification was performed on a Smartline series HPLC system (Knauer, Berlin, Germany), equipped with a UV/Vis-Detector S-2550 (190–1000 nm) and a Knauer Eurospher II 100-5 C18 column (5 µm; 8 x 250 mm). Elution was performed with 95% MeOH/$H_2O$ at 5 mL/min (155 bar). The UV/Vis absorption was monitored at 235 nm.

A)

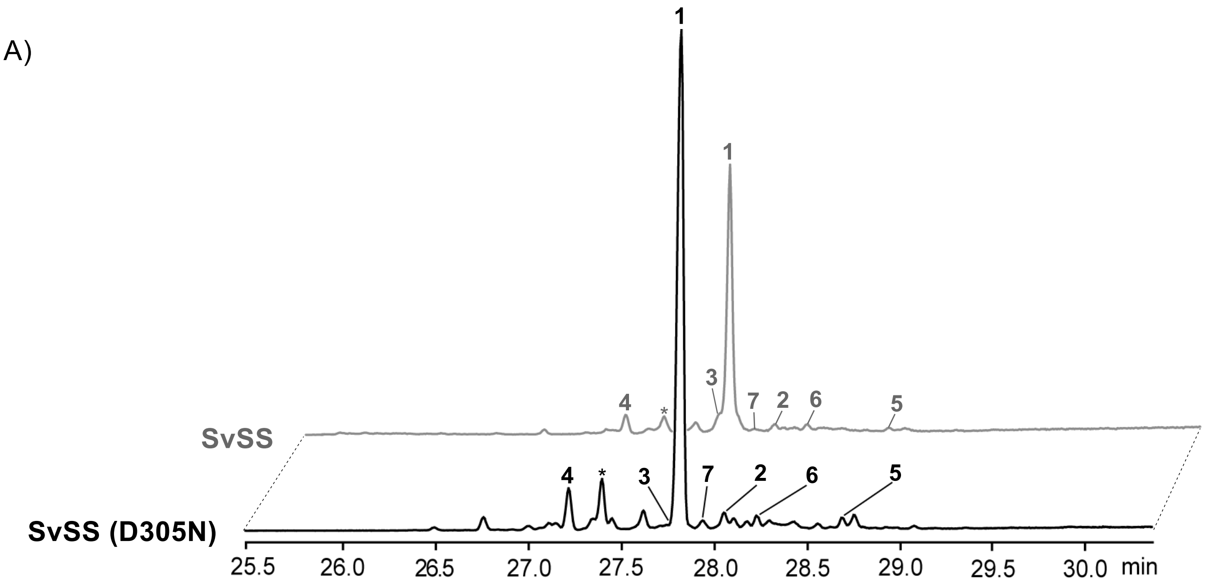


B)

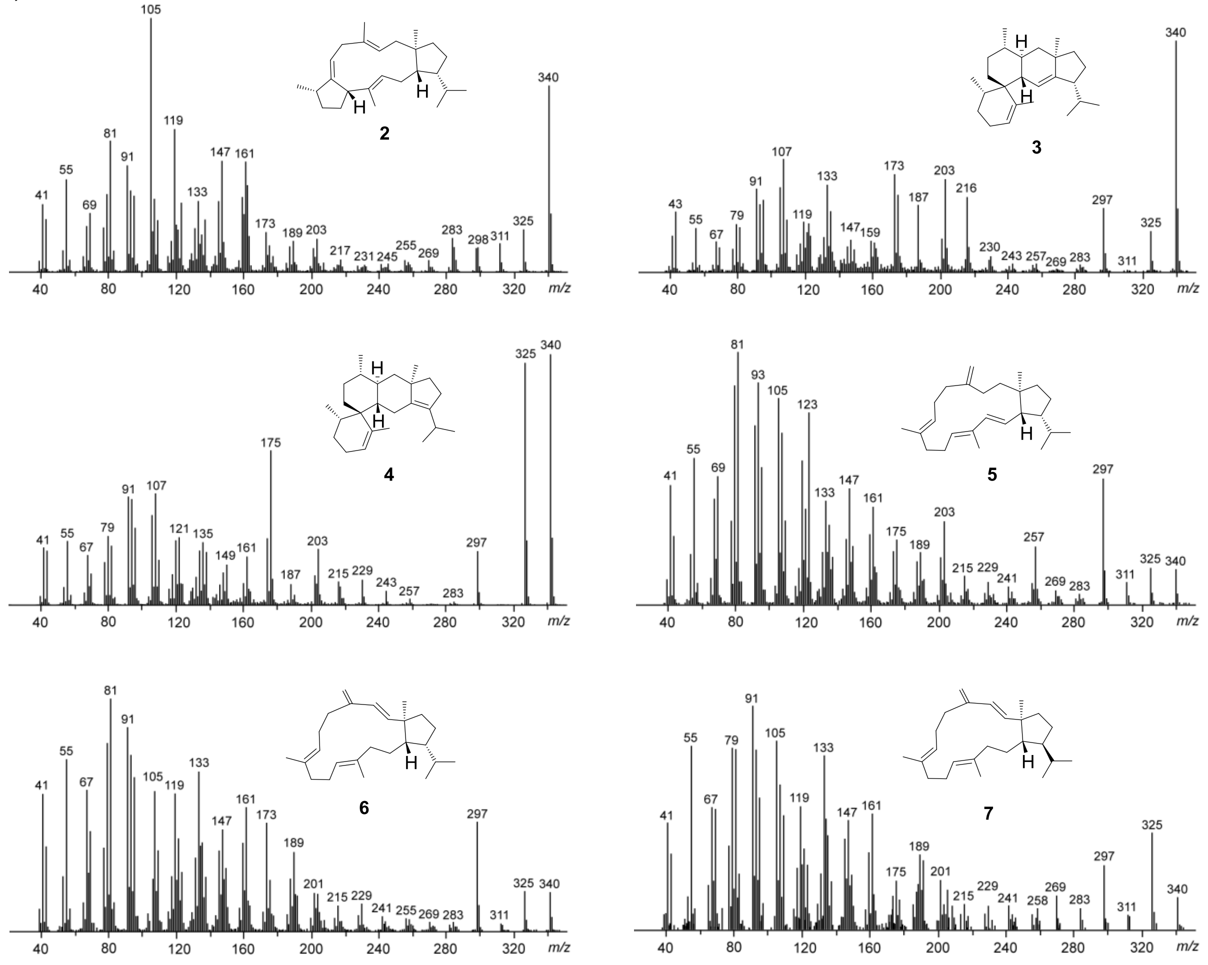


**Figure S21.** A) Total ion chromatogram of the products obtained from GFPP with SvSS (D305N). The total ion chromatogram of the products obtained from GFPP with SvSS (wild type) is shown in grey and asterisks indicate hydrolysis products of GFPP. B) Mass spectra of compounds **2** to **7**.

**Sesterviolene B (2).** Yield: 0.7 mg, 2.1 µmol, 0.8%, from 150 mg (263.4 µmol) GFPP triammonium salt. TLC (100% *n*-hexane): $R_f$ = 0.6. GC (HP5-MS): *I* = 2467. MS (EI, 70 eV): *m*/*z* (%) = 340 (70), 325 (15), 311 (10), 297 (9), 283 (13), 269 (4), 255 (5), 241 (3), 227 (2), 217 (6), 203 (11), 189 (14), 173 (15), 161 (42), 147 (40), 133 (29), 119 (58), 105 (100), 91 (41), 81 (50), 69 (22), 55 (32), 41 (25); conf. Figure S21B. IR (diamond ATR): ṽ = 2951 (s), 2922 (s), 2859 (s), 1731 (w), 1659 (w), 1632 (w), 1451 (m), 1384 (w), 1260 (w), 1093 (m), 1018 (m), 856 (w), 800 (s), 703 (w), 579 (w) $cm^{-1}$. HR-MS (Q-TOF, 70 eV): calc. $[C_{25}H_{40}]^{+\circ}$ *m*/*z* = 340.3125; found: *m*/*z* = 340.3123. Optical rotary power: $[\alpha]_D^{20}$ = –40.6 (*c* 0.07, $CH_2Cl_2$). NMR data are given in Table S6.

**Sesterviolene C (3).** Yield: 0.7 mg, 2.1 µmol, 0.8%, from 150 mg (263.4 µmol) GFPP triammonium salt. TLC (100% *n*-hexane): $R_f$ = 0.7. GC (HP5-MS): *I* = 2433. MS (EI, 70 eV): *m*/*z* (%) = 340 (100%), 325 (17), 297 (27), 283 (3), 257 (3), 243 (3), 230 (6), 216 (32), 203 (40), 187 (29), 173 (42), 159 (13), 147 (13), 133 (37), 119 (21), 107 (49), 91 (36), 79 (20), 67 (13), 55 (19), 43 (26); conf. Figure S21B. IR (diamond ATR): ṽ = 2947 (s), 2924 (s), 2867 (m), 2853 (m), 1658 (w), 1632 (w), 1466 (m), 1459 (m), 1376 (w), 1260 (w), 1103 (w), 1092 (w), 1064 (w), 1019 (m), 998 (w), 798 (m), 707 (w), 578 (w) $cm^{-1}$. HR-MS (Q-TOF, 70 eV): calc. $[C_{25}H_{40}]^{+\circ}$ *m*/*z* = 340.3125; found: *m*/*z* = 340.3122. Optical rotary power: $[\alpha]_D^{20}$ = +61.6 (*c* 0.07, $CH_2Cl_2$). NMR data are given in Table S7.

**Sesterviolene D (4).** Yield: 0.6 mg, 1.8 µmol, 0.7%, from 150 mg (263.4 µmol) GFPP triammonium salt. TLC (100% *n*-hexane): $R_f$ = 0.8. GC (HP5-MS): *I* = 2365. MS (EI, 70 eV): *m*/*z* (%) = 340 (100), 325 (97), 297 (21), 283 (1), 257 (2), 243 (5), 229 (10), 215 (9), 203 (22), 187 (8), 175 (62), 161 (19), 149 (16), 135 (25), 121 (27), 107 (44), 91 (43), 79 (27), 67 (20), 55 (25), 41 (23); conf. Figure S21B. IR (diamond ATR): ṽ = 2954 (s), 2924 (s), 2866 (s), 1657 (w), 1632 (w), 1459 (m), 1375 (m), 1360 (w), 1348 (w), 1304 (w), 1260 (m), 1196 (w), 1096 (m), 1018 (m), 973 (w), 883 (w), 803 (s), 724 (w), 695 (w), 663 (w), 577 (w), 516 (w) $cm^{-1}$. HR-MS (Q-TOF, 70 eV): calc. $[C_{25}H_{40}]^{+\circ}$ *m*/*z* = 340.3125; found: *m*/*z* = 340.3127. Optical rotary power: $[\alpha]_D^{20}$ = +55.0 (*c* 0.06, $CH_2Cl_2$). NMR data are given in Table S8.

**Sesterviolene E (5).** Yield: 0.9 mg, 2.6 µmol, 1.0%, from 150 mg (263.4 µmol) GFPP triammonium salt. TLC (100% *n*-hexane): $R_f$ = 0.5. GC (HP5-MS): *I* = 2539. MS (EI, 70 eV): *m*/*z* (%) = 340 (16), 325 (14), 311 (10), 297 (56), 283 (4), 269 (5), 257 (24), 241 (6), 229 (9), 215 (10), 203 (33), 189 (20), 175 (26), 161 (37), 147 (46), 133 (40), 123 (74), 119 (58), 105 (78), 93 (84), 81 (100), 69 (50), 55 (57), 41 (47); conf. Figure S21B. IR (diamond ATR): ṽ = 2922 (s), 2870 (m), 2853 (m), 2278 (w), 1663 (w), 1643 (w), 1447 (m), 1384 (w), 1345 (w), 1260 (w), 1098 (w), 1029 (w), 975 (m), 884 (m), 843 (w), 816 (m), 804 (w), 543 (m), 466 (w), 439 (w) $cm^{-1}$. HR-MS (Q-TOF, 70 eV): calc. $[C_{25}H_{40}]^{+\circ}$ *m*/*z* = 340.3125; found: *m*/*z* = 340.3132. Optical rotary power: $[\alpha]_D^{20}$ = –43.5 (*c* 0.09, $CH_2Cl_2$). NMR data are given in Table S9.

**Sesterviolene F (6).** Yield: 0.8 mg, 2.4 µmol, 0.9%, from 150 mg (263.4 µmol) GFPP triammonium salt. TLC (100% *n*-hexane): $R_f$ = 0.4. GC (HP5-MS): *I* = 2483. MS (EI, 70 eV): *m*/*z* (%) = 340 (18), 325 (18), 311 (4), 297 (47), 283 (4), 269 (5), 257 (7), 241 (6), 229 (13), 215 (11), 203 (19), 189 (36), 173 (46), 161 (55), 147 (48), 133 (71), 119 (60), 105 (84), 91 (93), 81 (100), 67 (63), 55 (74), 41 (55); conf. Figure S21B. IR (diamond ATR): ṽ = 2951 (s), 2925 (s), 2869 (s), 2277 (w), 1660 (w), 1642 (w), 1602 (w), 1566 (w), 1466 (m), 1446 (m), 1382 (w), 1345 (w), 1311 (w), 1261 (w), 1093 (m), 1020 (m), 969 (m), 880 (m), 843 (w), 815 (m), 804 (m), 627 (w), 587 (w), 543 (s), 495 (w), 466 (w), 438 (w) $cm^{-1}$. HR-MS (Q-TOF, 70 eV): calc. $[C_{25}H_{40}]^{+\circ}$ *m*/*z* = 340.3125; found: *m*/*z* = 340.3131. Optical rotary power: $[\alpha]_D^{20}$ = +12.5 (*c* 0.08, $CH_2Cl_2$). NMR data are given in Table S10.

**Sesterviolene G (7).** Yield: 0.3 mg, 0.9 µmol, 0.3%, from 150 mg (263.4 µmol) GFPP triammonium salt. TLC (100% *n*-hexane): $R_f$ = 0.4. GC (HP5-MS): *I* = 2448. MS (EI, 70 eV): *m*/*z* (%) = 340 (20), 325 (47), 311 (6), 297 (39), 283 (10), 269 (13), 255 (11), 241 (7), 229 (11), 215 (7), 201 (25), 189 (36), 173 (19), 161 (39), 147 (48), 133 (71), 119 (60), 105 (86), 91 (100), 81 (90), 69 (58), 55 (71), 41 (52); conf. Figure S21B. IR (diamond ATR): $\tilde{\nu}$ = 2955 (s), 2924 (s), 2871 (s), 1665 (w), 1640 (w), 1600 (s), 1449 (m), 1383 (w), 1260 (m), 1092 (s), 1020 (s), 968 (m), 880 (m), 801 (s), 726 (w), 697 (w) $cm^{-1}$. HR-MS (Q-TOF, 70 eV): calc. $[C_{25}H_{40}]^{+\cdot}$ *m*/*z* = 340.3125; found: *m*/*z* = 340.3132. Optical rotary power: $[\alpha]_D^{20}$ = –56.7 (*c* 0.03, $CH_2Cl_2$). NMR data are given in Table S11.

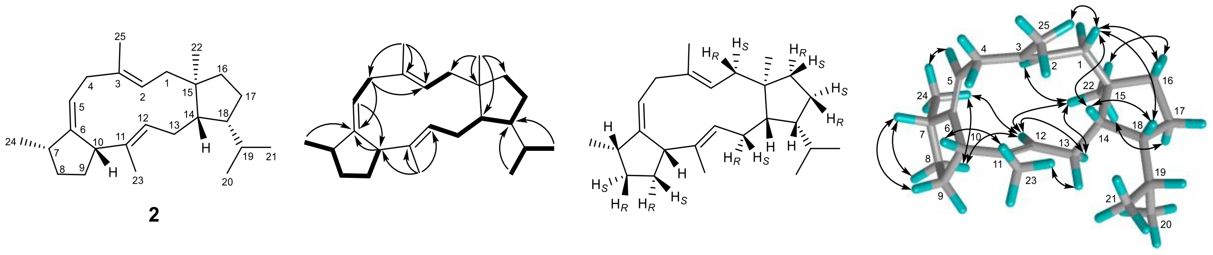

**Figure S22.** Structure elucidation of **2**. Bold: $^{1}H$,$^{1}H$-COSY, single headed arrows: key HMBC, and double headed arrows: NOESY correlations. Carbon numbering follows GFPP numbering to indicate the origin of each carbon. Diastereotopic hydrogens are labelled $H_R$ (*pro-R*) and $H_S$ (*pro-S*).

**Table S6.** NMR data of sesterviolene B (**2**) in $C_6D_6$ recorded at 298 K.

| C[a] | type | $^{1}H$[b] | $^{13}C$[b] |
|---|---|---|---|
| 1 | $CH_2$ | 2.11 (dd, *J* = 12.9, 7.9, $H_S$) | 43.55 |
| | | 1.95 (dd, *J* = 13.0, 8.4, $H_R$) | |
| 2 | CH | 5.29 (tq, *J* = 8.1, 1.5) | 124.32 |
| 3 | $C_q$ | – | 133.79 |
| 4 | $CH_2$ | 2.67 (m, 2H) | 41.35 |
| 5 | CH | 5.66 (ddt, *J* = 9.2, 6.9, 2.2) | 119.90 |
| 6 | $C_q$ | – | 151.43 |
| 7 | CH | 2.46 (m) | 39.54 |
| 8 | $CH_2$ | 1.71 (m, $H_R$) | 32.99 |
| | | 1.44 (m, $H_S$) | |
| 9 | $CH_2$ | 1.63 (m, $H_S$) | 31.48 |
| | | 1.60 (m, $H_R$) | |
| 10 | CH | 3.21 (br d, *J* = 7.9) | 51.05 |
| 11 | $C_q$ | – | 136.15 |
| 12 | CH | 5.16 (ddt, *J* = 10.3, 6.0, 1.4) | 127.39 |
| 13 | $CH_2$ | 2.31 (ddd, *J* = 13.2, 10.2, 2.6, $H_S$) | 26.10 |
| | | 1.56 (m, $H_R$) | |
| 14 | CH | 1.80 (ddd, *J* = 12.4, 9.3, 2.4) | 45.83 |
| 15 | $C_q$ | – | 46.65 |
| 16 | $CH_2$ | 1.48 (m, $H_R$) | 45.31 |
| | | 1.35 (td, *J* = 11.9, 4.9, $H_S$) | |
| 17 | $CH_2$ | 1.52 (m, $H_R$) | 28.49 |
| | | 1.20 (m, $H_S$) | |
| 18 | CH | 1.51 (m) | 53.84 |
| 19 | CH | 1.61 (m) | 28.06 |
| 20/21 | $CH_3$ | 1.01 (d, *J* = 6.3) | 23.44 |
| 2120 | $CH_3$ | 0.89 (d, *J* = 6.6) | 22.49 |
| 22 | $CH_3$ | 1.07 (s) | 24.36 |
| 23 | $CH_3$ | 1.67 (br s) | 17.01 |
| 24 | $CH_3$ | 1.21 (d, *J* = 6.8) | 19.89 |
| 25 | $CH_3$ | 1.64 (br s) | 17.16 |

[a] Carbon numbering as shown in Figure S22. [b] Chemical shifts $\delta$ in ppm, multiplicity: s = singlet, d = doublet, t = triplet, q = quartet, m = multiplet, br = broad, coupling constants *J* are given in Hertz.

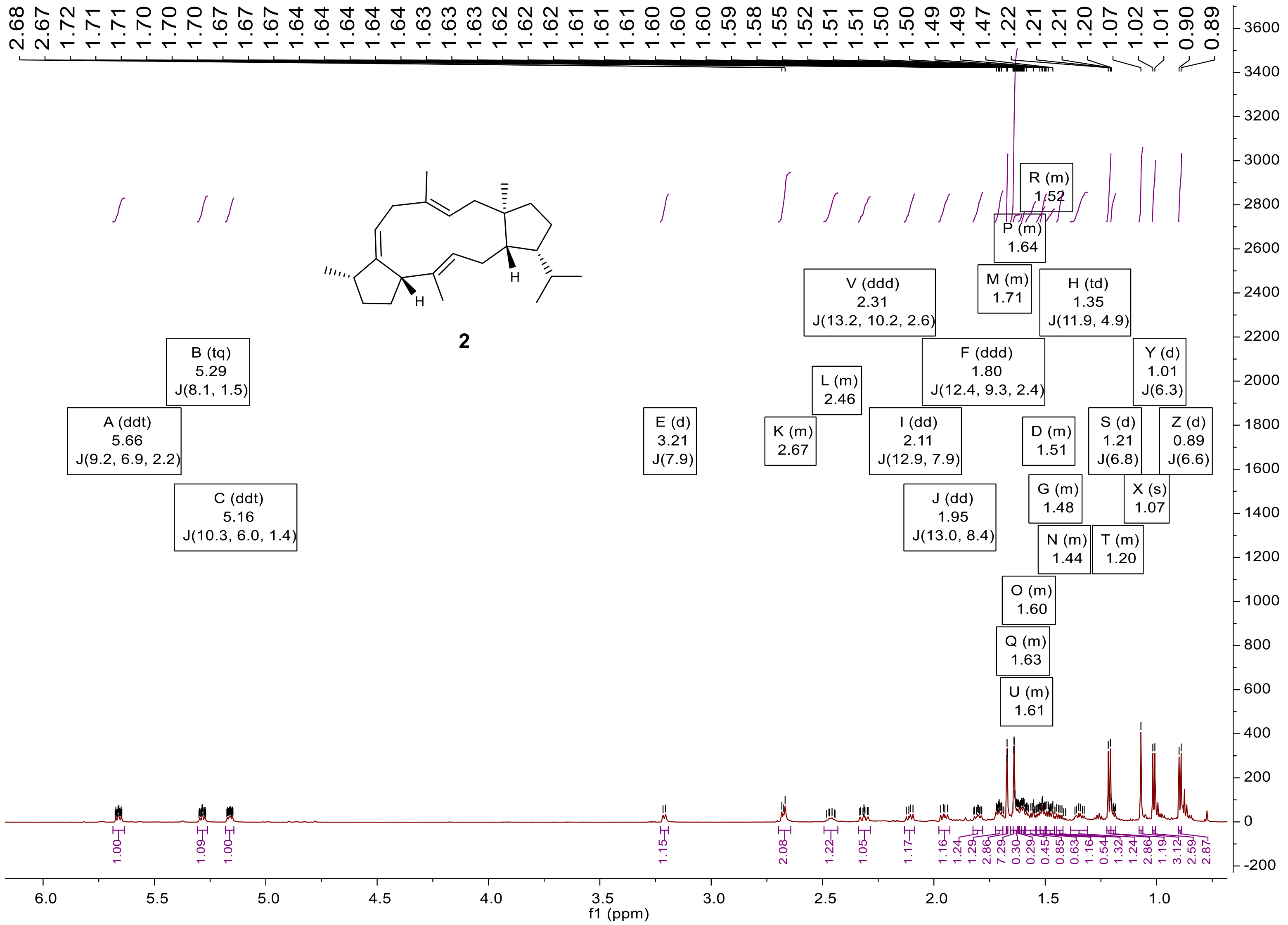


**Figure S23.** $^1$H-NMR spectrum of **2** (700 MHz, $C_6D_6$).

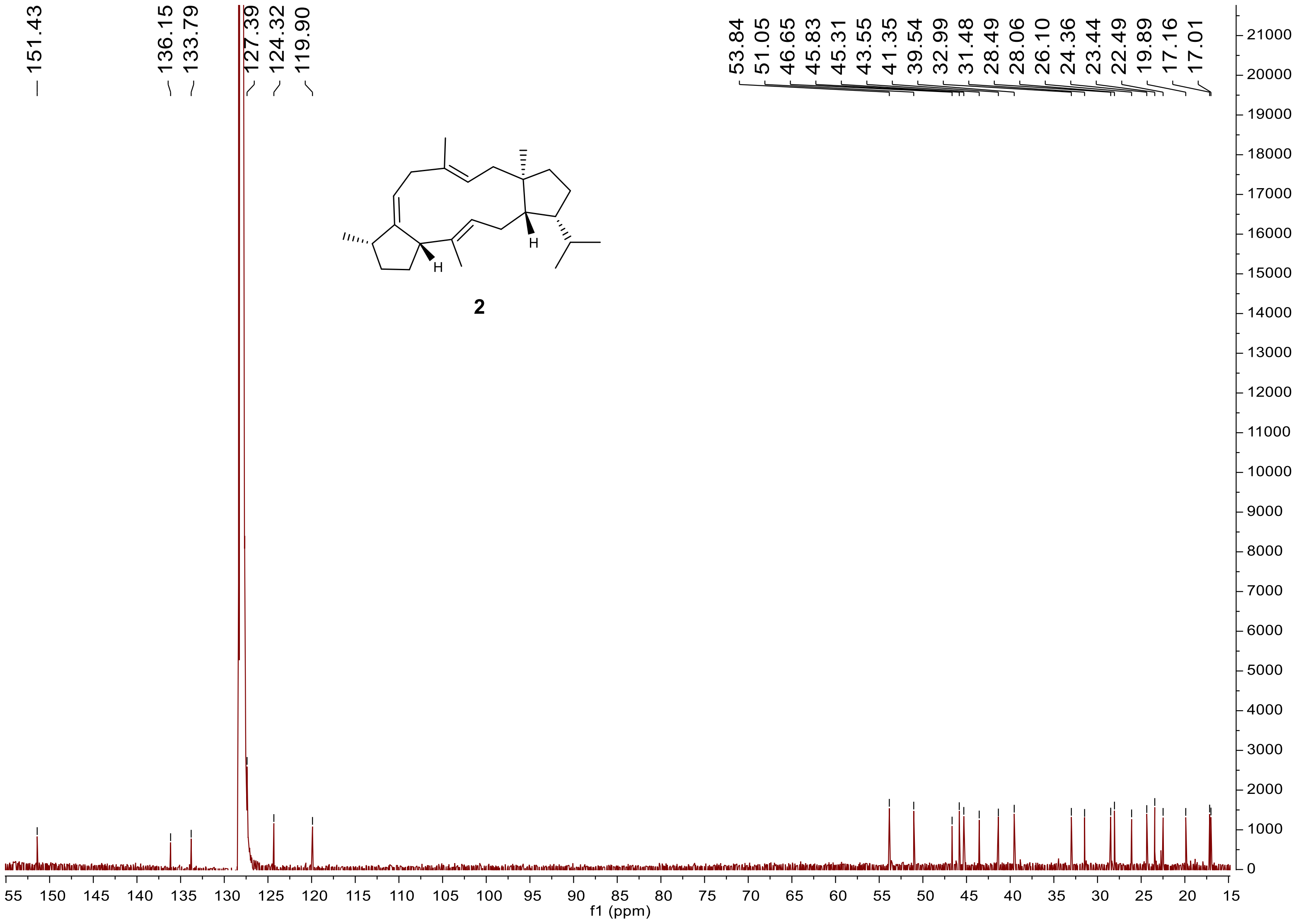


**Figure S24.** $^{13}$C-NMR spectrum of **2** (176 MHz, $C_6D_6$).

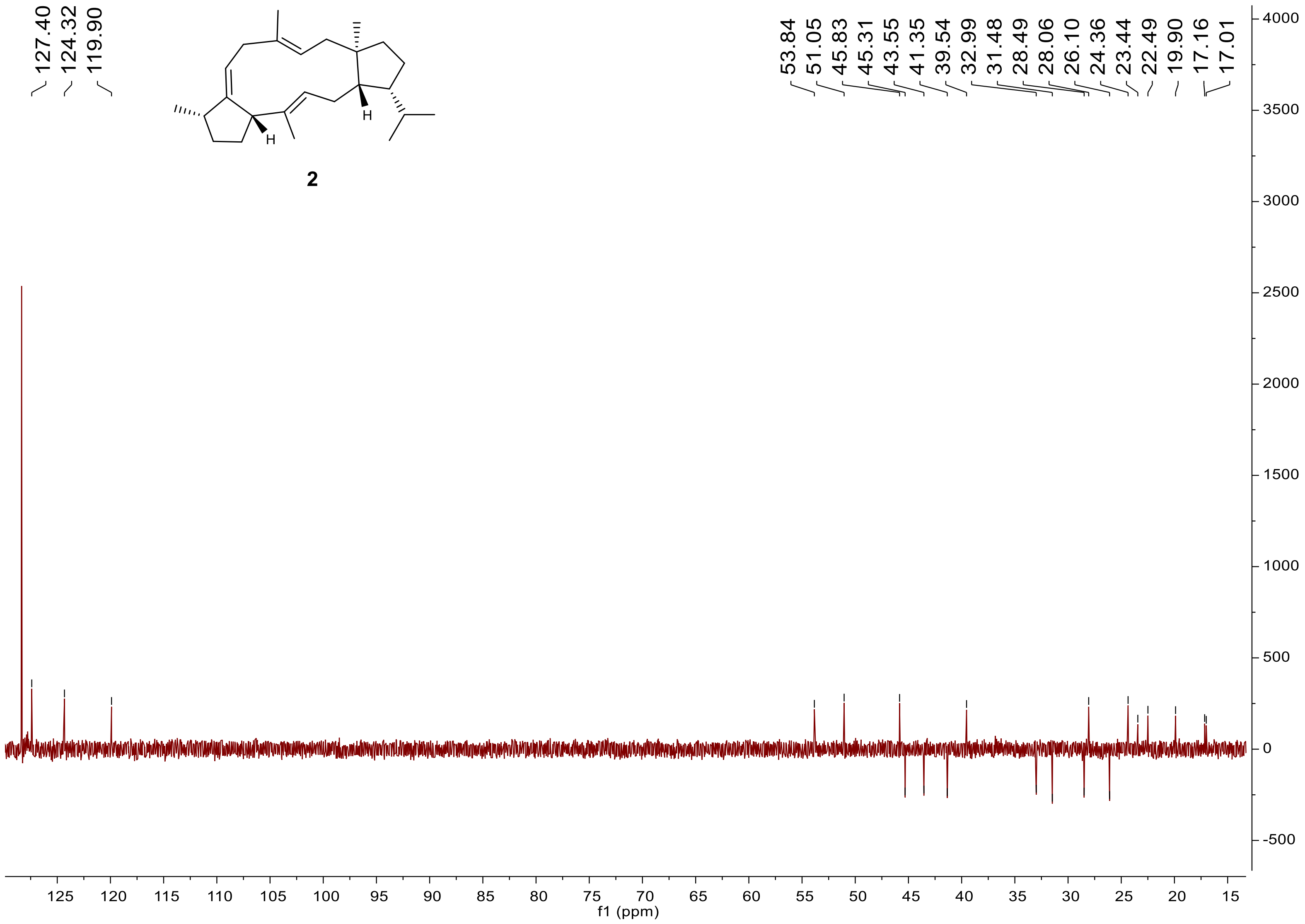


**Figure S25.** $^{13}$C-DEPT135 spectrum of **2** (176 MHz, $C_6D_6$).

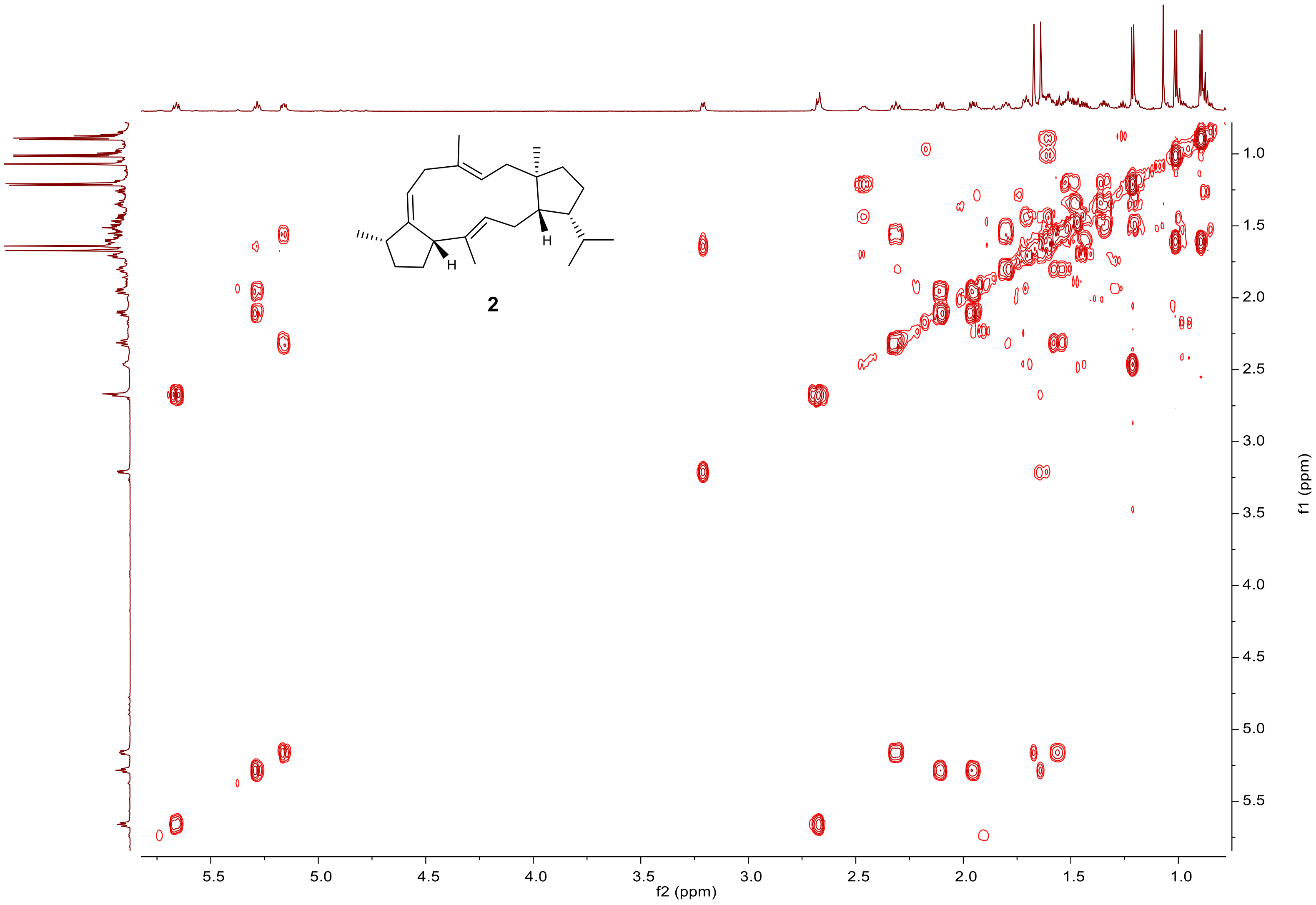


**Figure S26.** $^{1}$H-$^{1}$H-COSY spectrum ($C_6D_6$) of **2**.

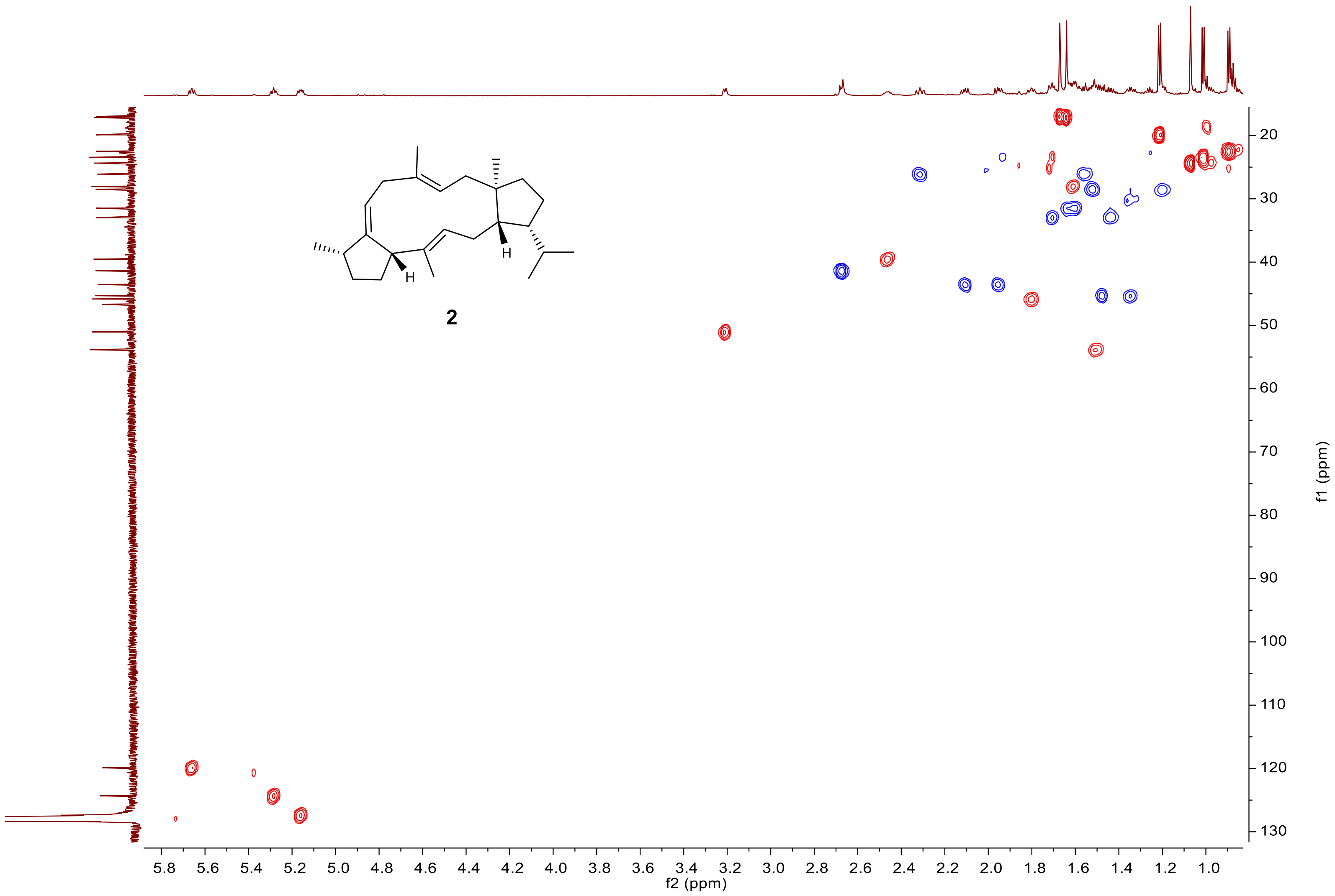


**Figure S27.** HSQC spectrum ($C_6D_6$) of **2**.

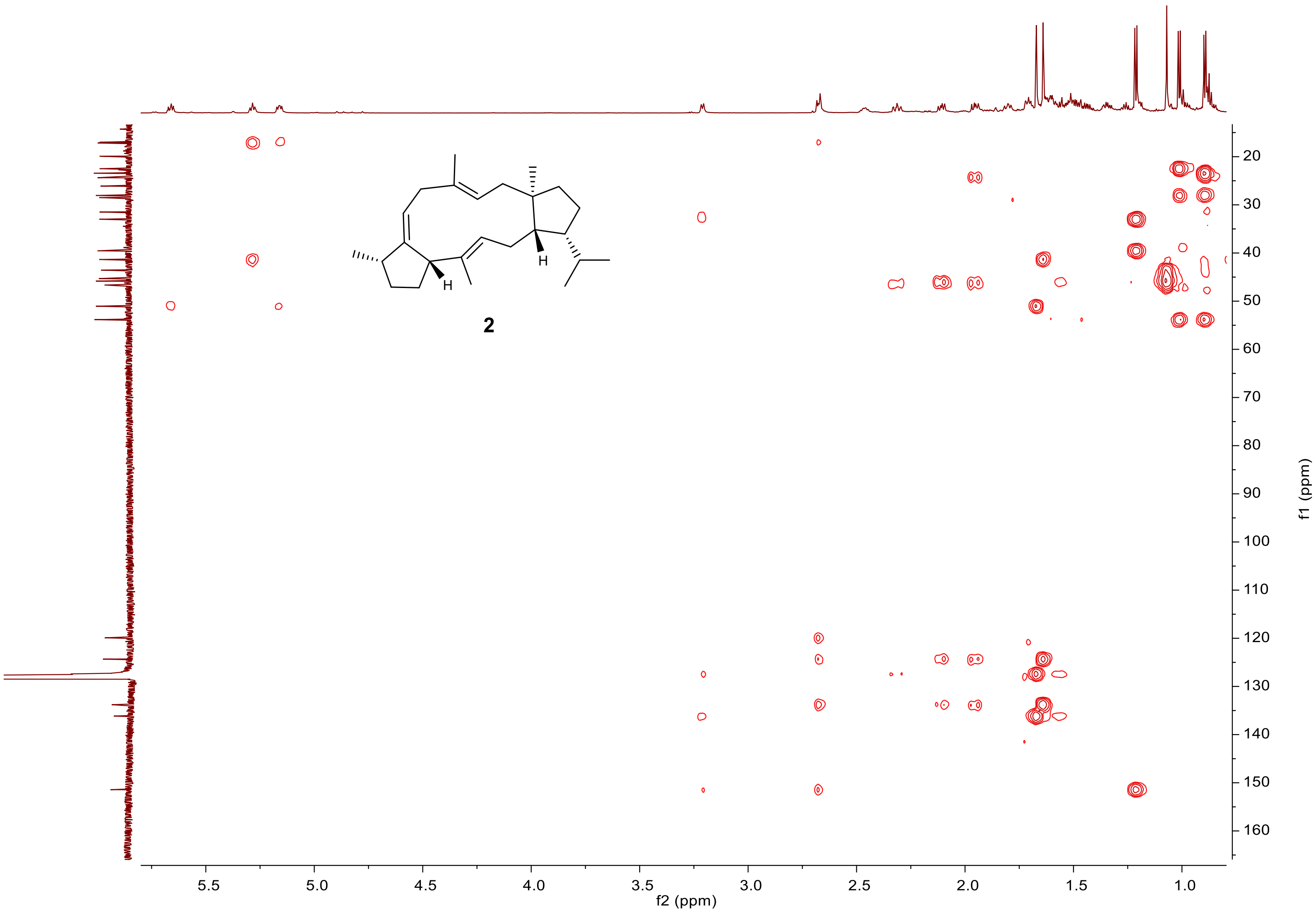


**Figure S28.** HMBC spectrum ($C_6D_6$) of **2**.

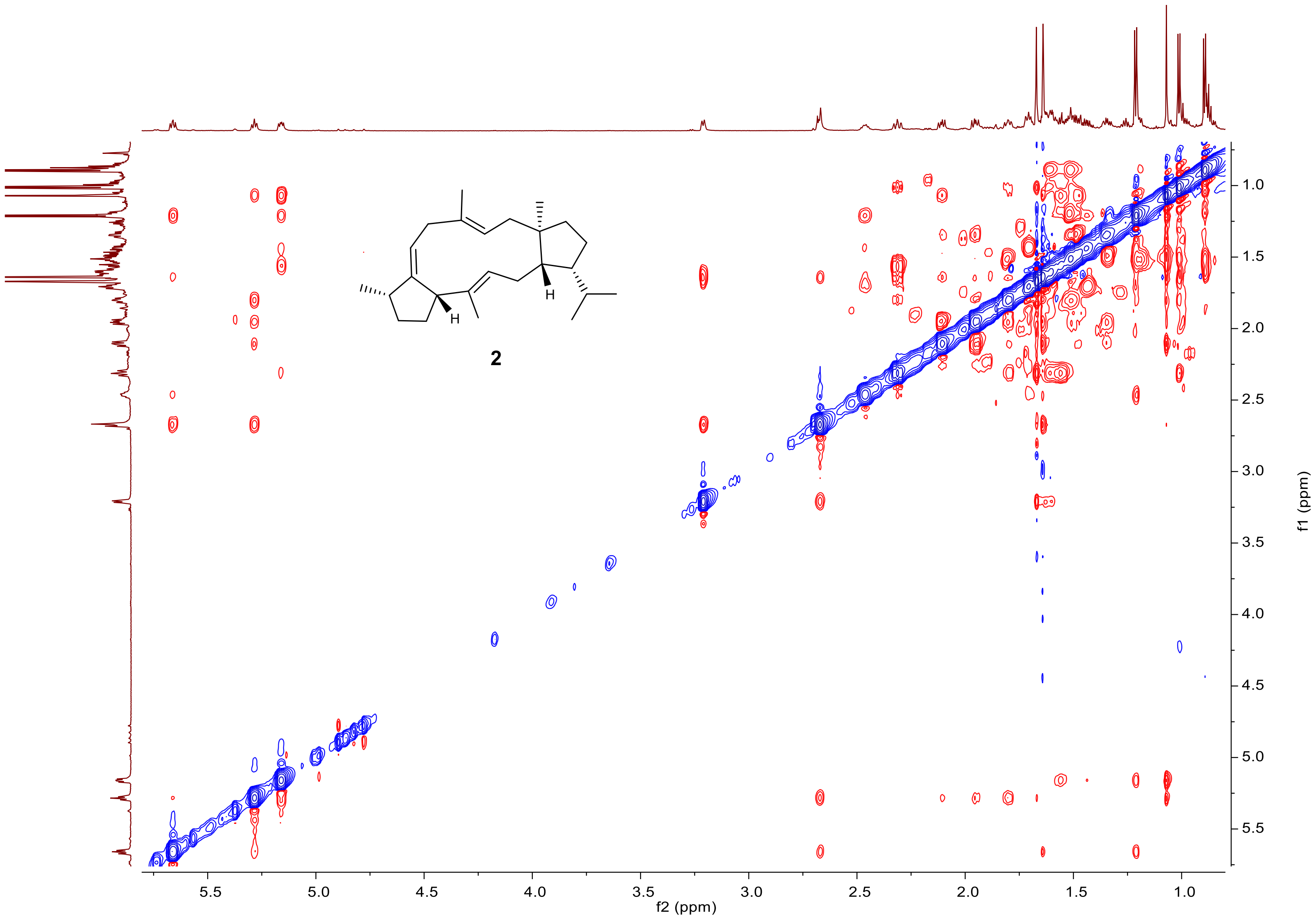


**Figure S29.** NOESY spectrum ($C_6D_6$) of **2**.

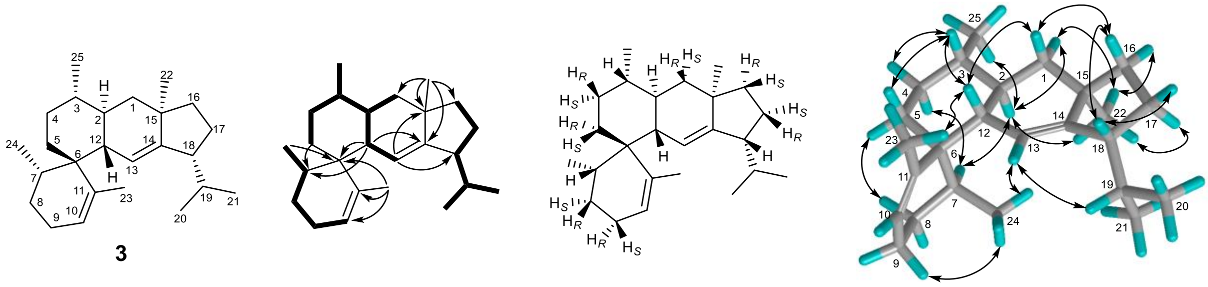

**Figure S30.** Structure elucidation of **3**. Bold: $^1H,^1H$-COSY, single headed arrows: key HMBC, and double headed arrows: NOESY correlations. Carbon numbering follows GFPP numbering to indicate the origin of each carbon. Diastereotopic hydrogens are labelled $H_R$ (*pro-R*) and $H_S$ (*pro-S*).

**Table S7.** NMR data of sesterviolene C (**3**) in $C_6D_6$ recorded at 298 K.

| C[a] | type | $^1H$[b] | $^{13}C$[b] |
|---|---|---|---|
| 1 | $CH_2$ | 2.07 (dd, *J* = 12.2, 2.7, $H_S$) | 43.59 |
| | | 0.96 (t, *J* = 12.2, $H_R$) | |
| 2 | CH | 1.52 (m) | 36.53 |
| 3 | CH | 1.05 (m) | 38.17 |
| 4 | $CH_2$ | 1.42 (m, $H_R$) | 31.32 |
| | | 1.36 (m, $H_S$) | |
| 5 | $CH_2$ | 1.68 (m, $H_R$) | 36.61 |
| | | 1.56 (td, *J* = 13.7, 4.3, $H_S$) | |
| 6 | $C_q$ | – | 43.86 |
| 7 | CH | 2.23 (m) | 29.72 |
| 8 | $CH_2$ | 1.86 (m, $H_R$) | 27.00 |
| | | 1.20 (ddd, *J* = 12.0, 5.8, 3.4, $H_S$) | |
| 9 | $CH_2$ | 2.14 (m, $H_R$) | 21.45 |
| | | 1.81 (m, $H_S$) | |
| 10 | CH | 5.52 (m) | 124.51 |
| 11 | $C_q$ | – | 138.63 |
| 12 | CH | 2.25 (m) | 48.89 |
| 13 | CH | 5.52 (m) | 125.17 |
| 14 | $C_q$ | – | 145.88 |
| 15 | $C_q$ | – | 41.73 |
| 16 | $CH_2$ | 1.61 (dd, *J* = 12.0, 6.8, $H_R$) | 42.03 |
| | | 1.25 (td, *J* = 12.0, 7.5, $H_S$) | |
| 17 | $CH_2$ | 1.71 (m, $H_R$) | 26.49 |
| | | 1.47 (m, $H_S$) | |
| 18 | CH | 2.25 (m) | 50.58 |
| 19 | CH | 1.66 (m) | 34.13 |
| 20/21 | $CH_3$ | 1.02 (d, *J* = 6.7) | 22.11 |
| 21/20 | $CH_3$ | 0.88 (d, *J* = 6.7) | 20.81 |
| 22 | $CH_3$ | 1.07 (s) | 24.98 |
| 23 | $CH_3$ | 1.78 (dt, *J* = 2.7, 1.4) | 19.73 |
| 24 | $CH_3$ | 1.15 (d, *J* = 7.0) | 16.88 |
| 25 | $CH_3$ | 0.98 (d, *J* = 6.4) | 20.19 |

[a] Carbon numbering as shown in Figure S30. [b] Chemical shifts δ in ppm, multiplicity: s = singlet, d = doublet, t = triplet, m = multiplet, coupling constants *J* are given in Hertz.

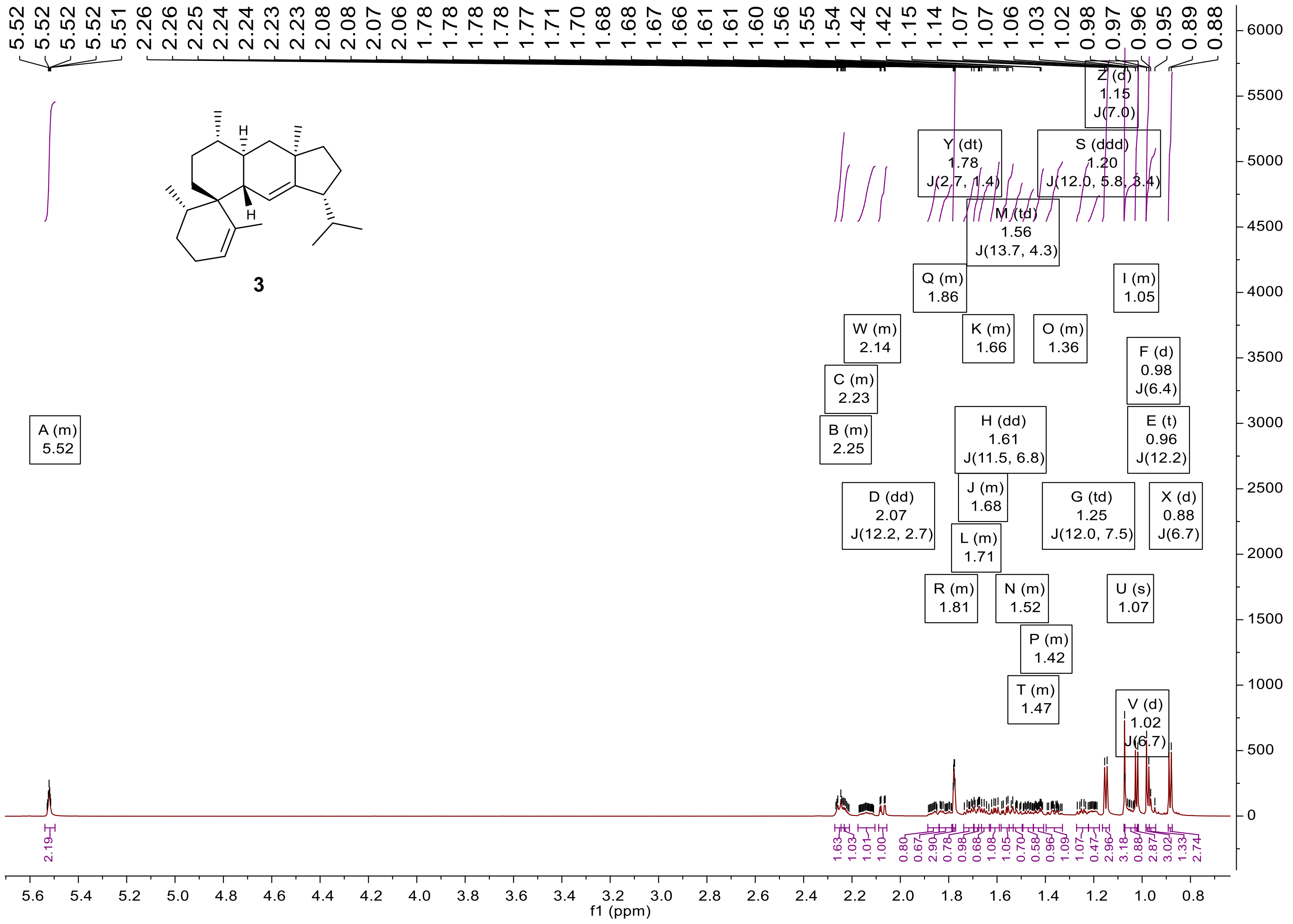


**Figure S31.** $^1$H-NMR spectrum of **3** (700 MHz, $C_6D_6$).

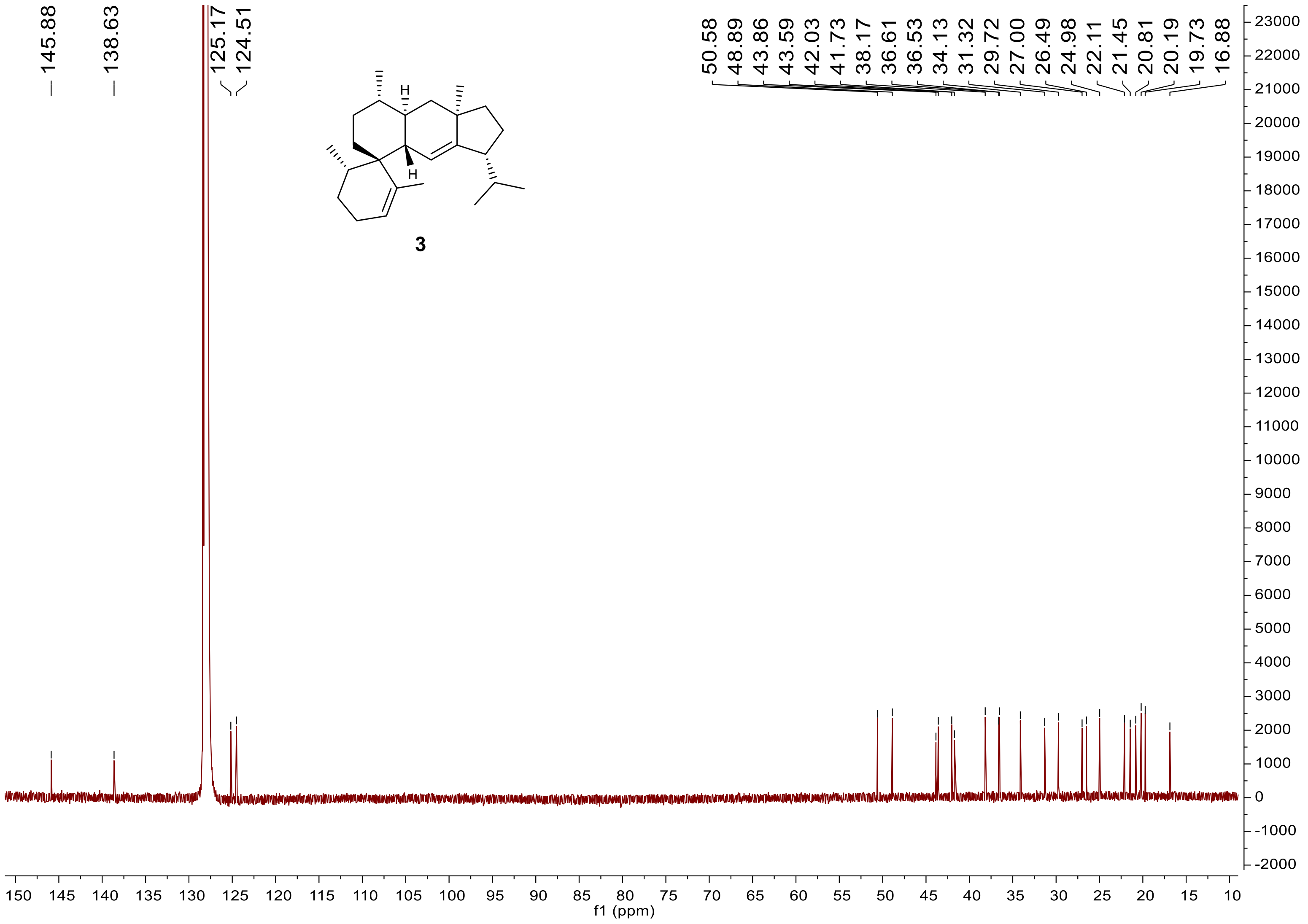


**Figure S32.** $^{13}$C-NMR spectrum of **3** (176 MHz, $C_6D_6$).

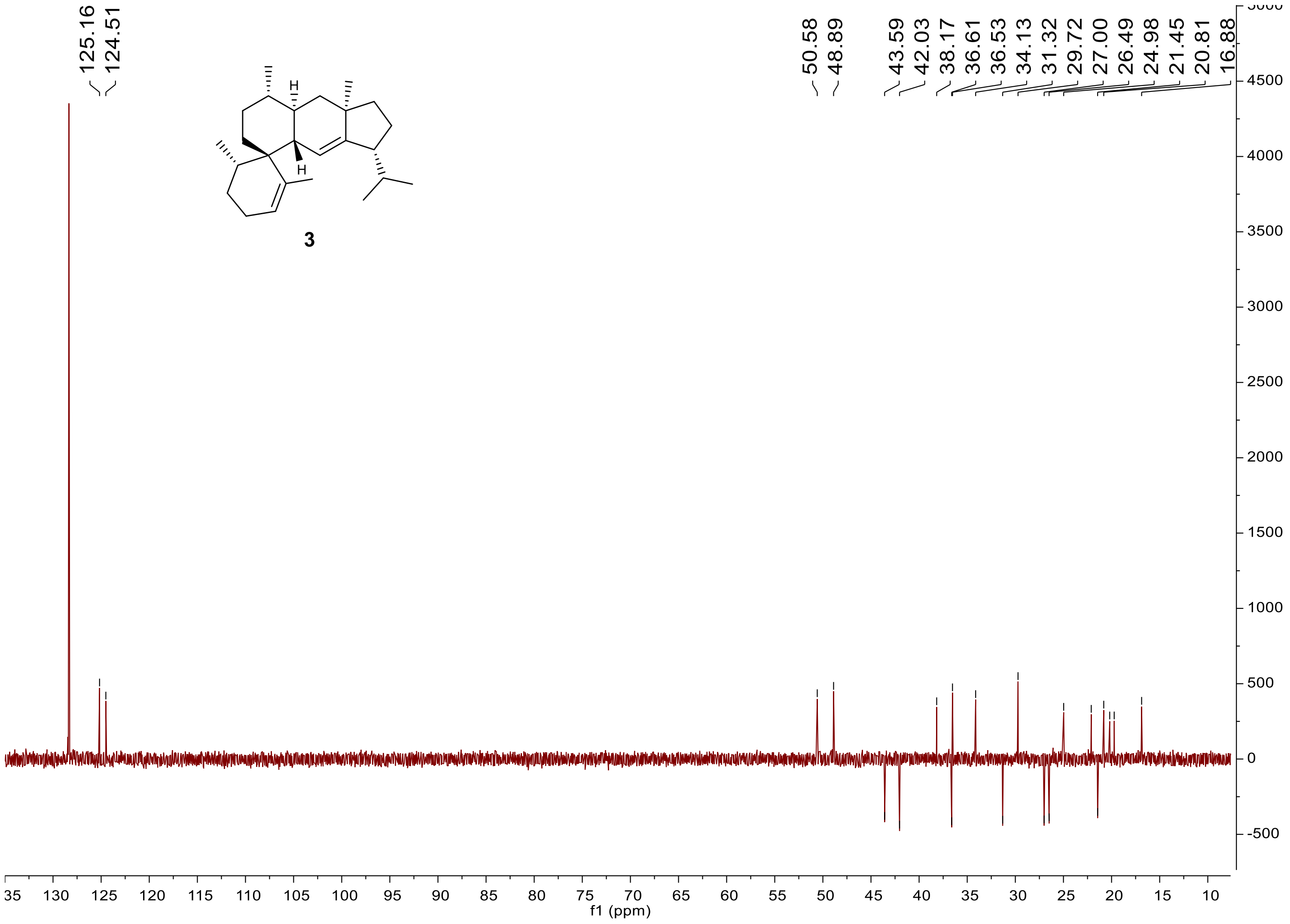


**Figure S33.** $^{13}$C-DEPT135 spectrum of **3** (176 MHz, $C_6D_6$).

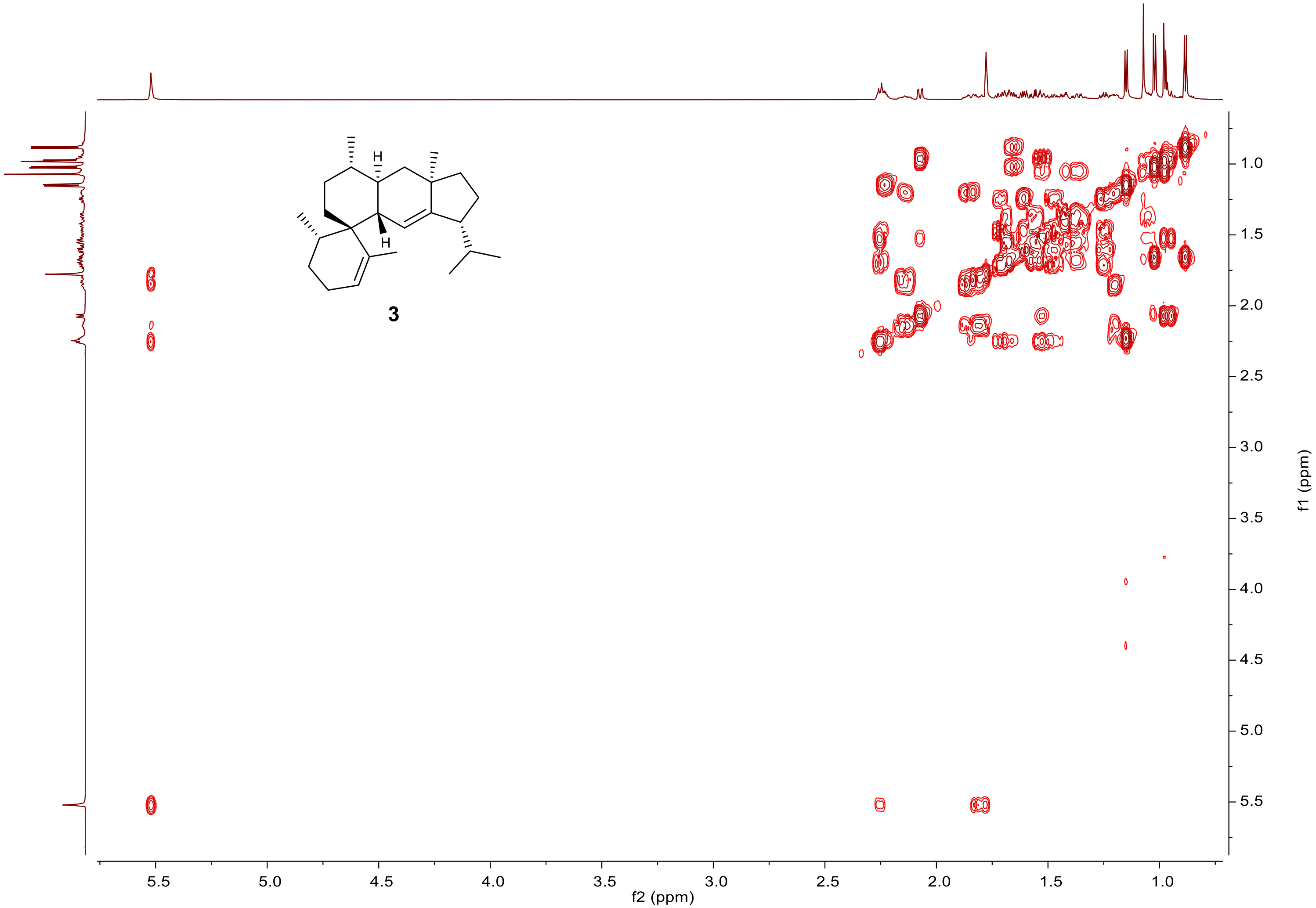


**Figure S34.** $^1H$-$^1H$-COSY spectrum ($C_6D_6$) of **3**.

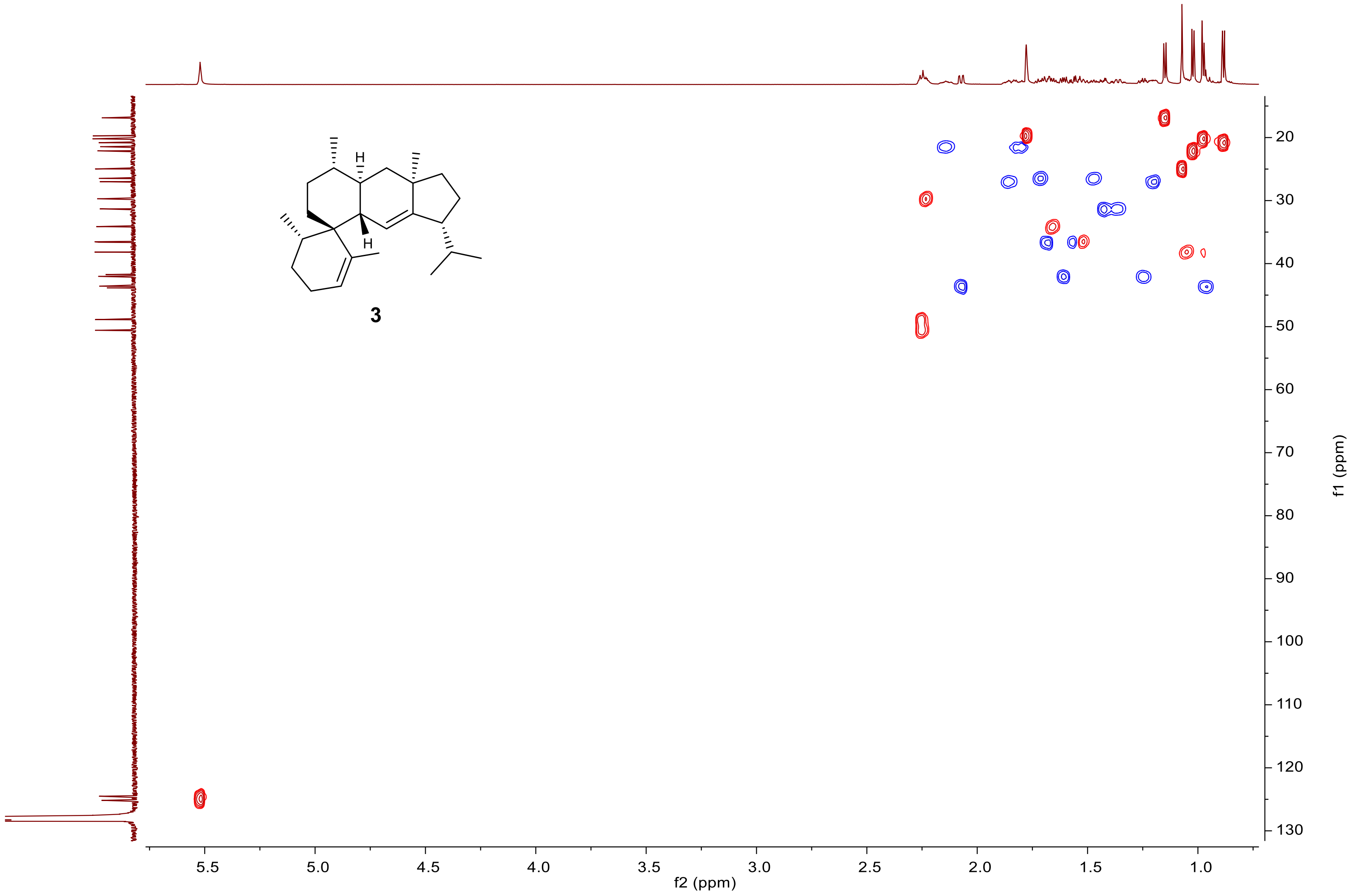


**Figure S35.** HSQC spectrum ($C_6D_6$) of **3**.

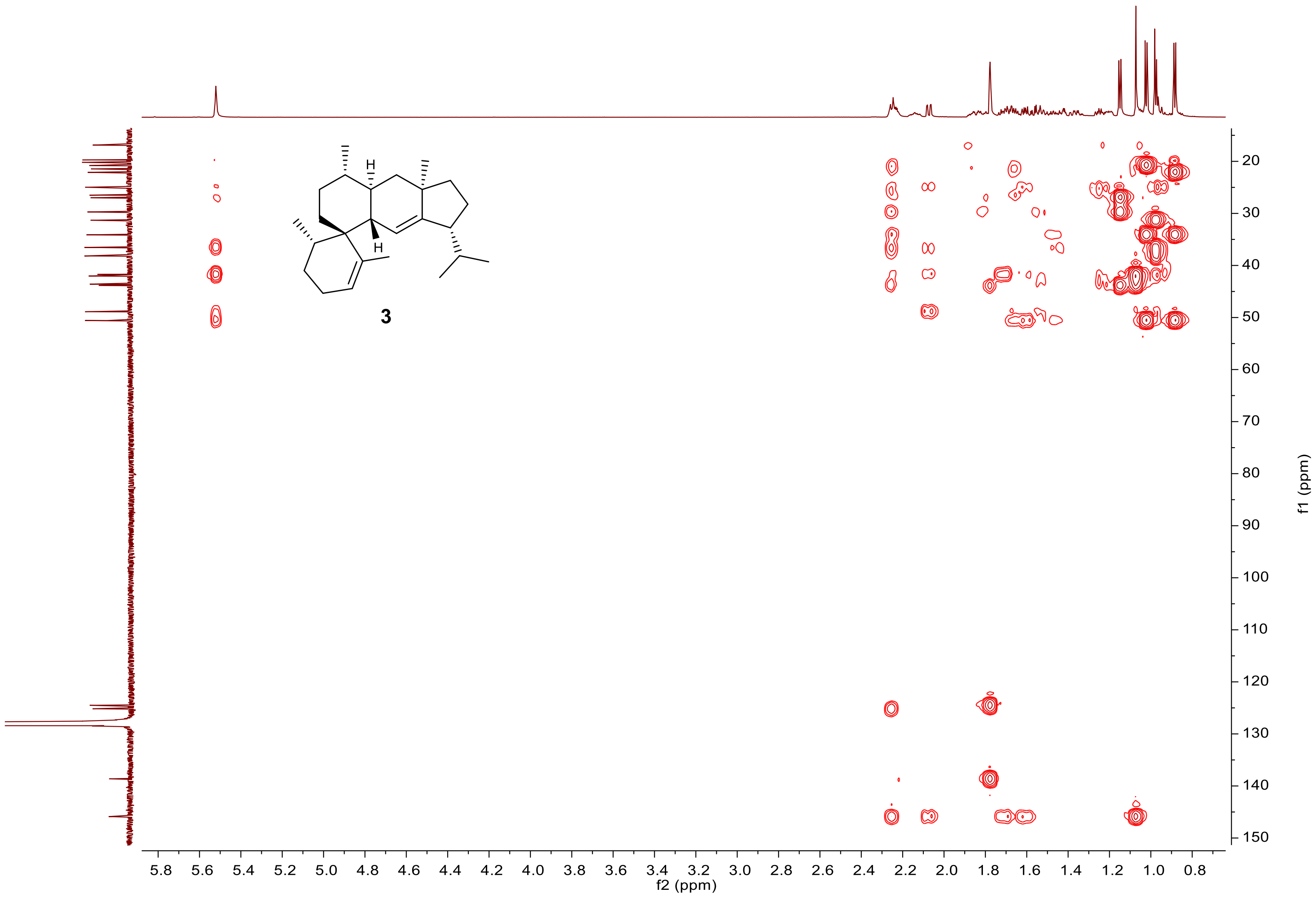


**Figure S36.** HMBC spectrum ($C_6D_6$) of **3**.

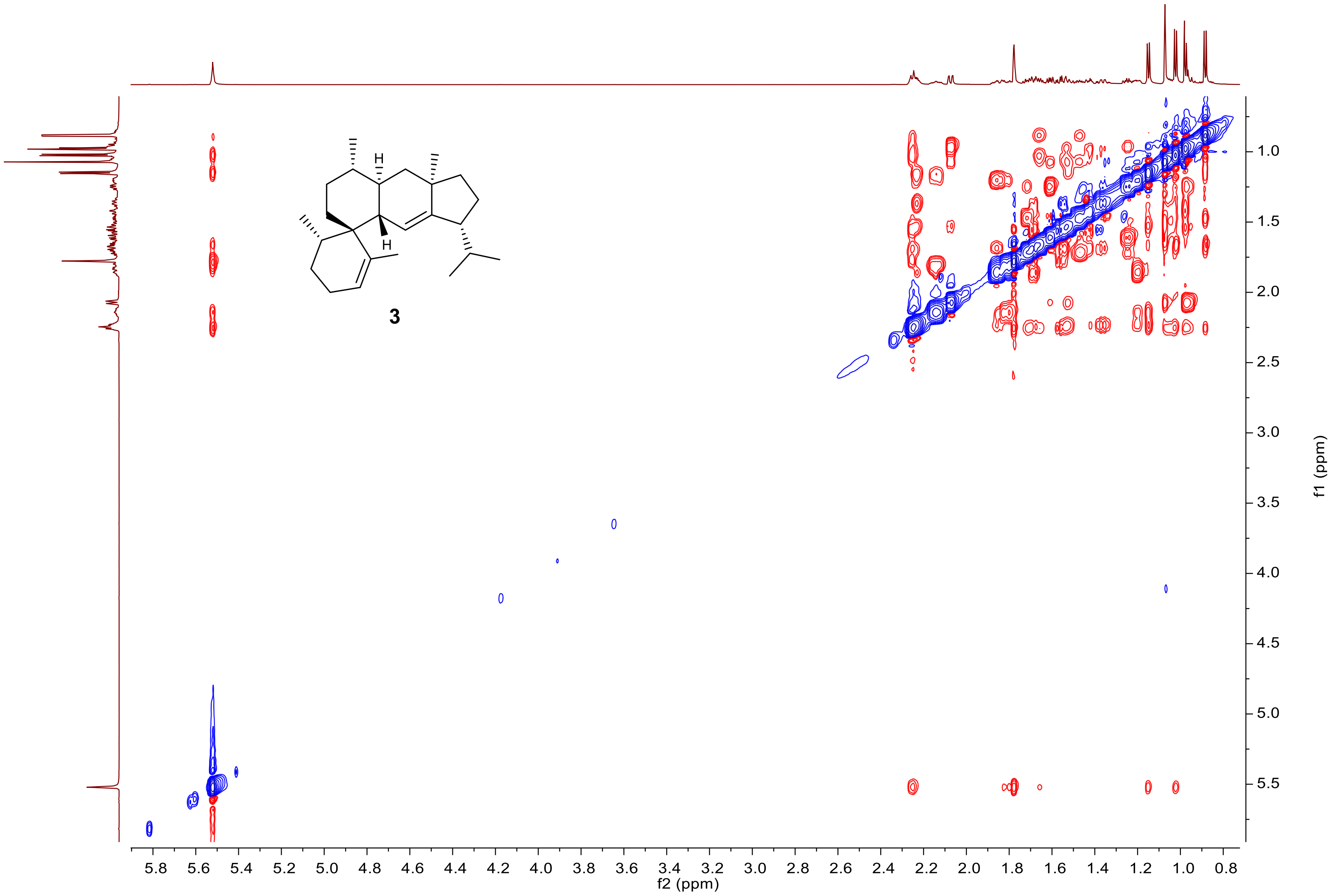


**Figure S37.** NOESY spectrum ($C_6D_6$) of **3**.

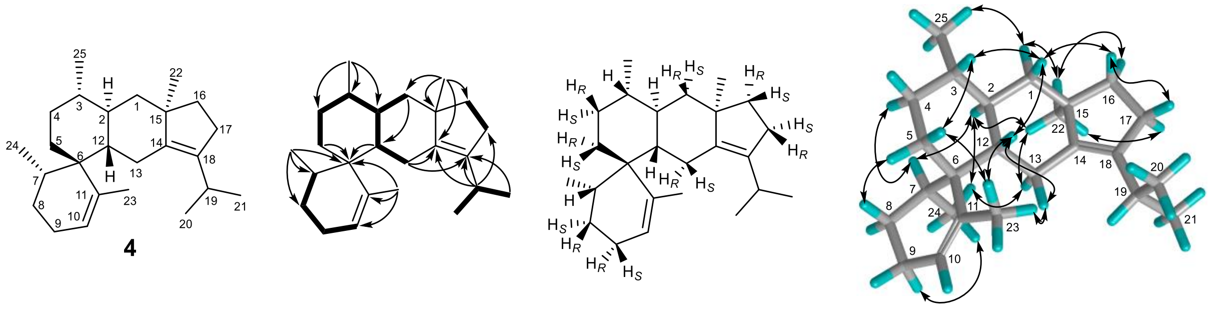

**Figure S38.** Structure elucidation of **4**. Bold: $^{1}H,^{1}H$-COSY, single headed arrows: key HMBC, and double headed arrows: NOESY correlations. Carbon numbering follows GFPP numbering to indicate the origin of each carbon. Diastereotopic hydrogens are labelled $H_R$ (*pro-R*) and $H_S$ (*pro-S*).

**Table S8.** NMR data of sesterviolene D (**4**) in $C_6D_6$ recorded at 298 K.

| C[a] | type | $^{1}H$[b] | $^{13}C$[b] |
|---|---|---|---|
| 1 | $CH_2$ | 2.14 (dd, $J$ = 12.7, 3.9, $H_S$) | 49.38 |
| | | 0.87 (dd, $J$ = 12.7, 11.1, $H_R$) | |
| 2 | CH | 1.47 (m) | 38.56 |
| 3 | CH | 0.93 (m) | 39.89 |
| 4 | $CH_2$ | 1.37 (m, 2H) | 30.77 |
| 5 | $CH_2$ | 1.64 (m, $H_S$) | 37.95 |
| | | 1.54 (m, $H_R$) | |
| 6 | $C_q$ | – | 44.24 |
| 7 | CH | 2.25 (m) | 30.61 |
| 8 | $CH_2$ | 1.85 (m, $H_R$) | 28.83 |
| | | 1.21 (m, $H_S$) | |
| 9 | $CH_2$ | 2.12 (m, $H_R$) | 21.75 |
| | | 1.81 (m, $H_S$) | |
| 10 | CH | 5.53 (m) | 124.64 |
| 11 | $C_q$ | – | 139.28 |
| 12 | CH | 1.39 (m) | 49.98 |
| 13 | $CH_2$ | 2.40 (dd, $J$ = 14.1, 3.6, $H_S$) | 25.93 |
| | | 2.27 (m, $H_R$) | |
| 14 | $C_q$ | – | 139.76 |
| 15 | $C_q$ | – | 47.69 |
| 16 | $CH_2$ | 1.78 (m, $H_R$) | 39.54 |
| | | 1.61 (m, $H_S$) | |
| 17 | $CH_2$ | 2.38 (m, $H_S$) | 28.43 |
| | | 2.31 (m, $H_R$) | |
| 18 | $C_q$ | – | 135.36 |
| 19 | CH | 2.78 (m) | 27.10 |
| 20/21 | $CH_3$ | 1.07 (d, $J$ = 6.9) | 22.26 |
| 21/20 | $CH_3$ | 0.98 (d, $J$ = 6.8) | 21.55 |
| 22 | $CH_3$ | 1.11 (s) | 24.77 |
| 23 | $CH_3$ | 1.80 (m) | 19.67 |
| 24 | $CH_3$ | 1.21 (d, $J$ = 7.0) | 19.36 |
| 25 | $CH_3$ | 0.94 (d, $J$ = 2.3) | 20.52 |

[a] Carbon numbering as shown in Figure S38. [b] Chemical shifts δ in ppm, multiplicity: s = singlet, d = doublet, m = multiplet, coupling constants $J$ are given in Hertz.

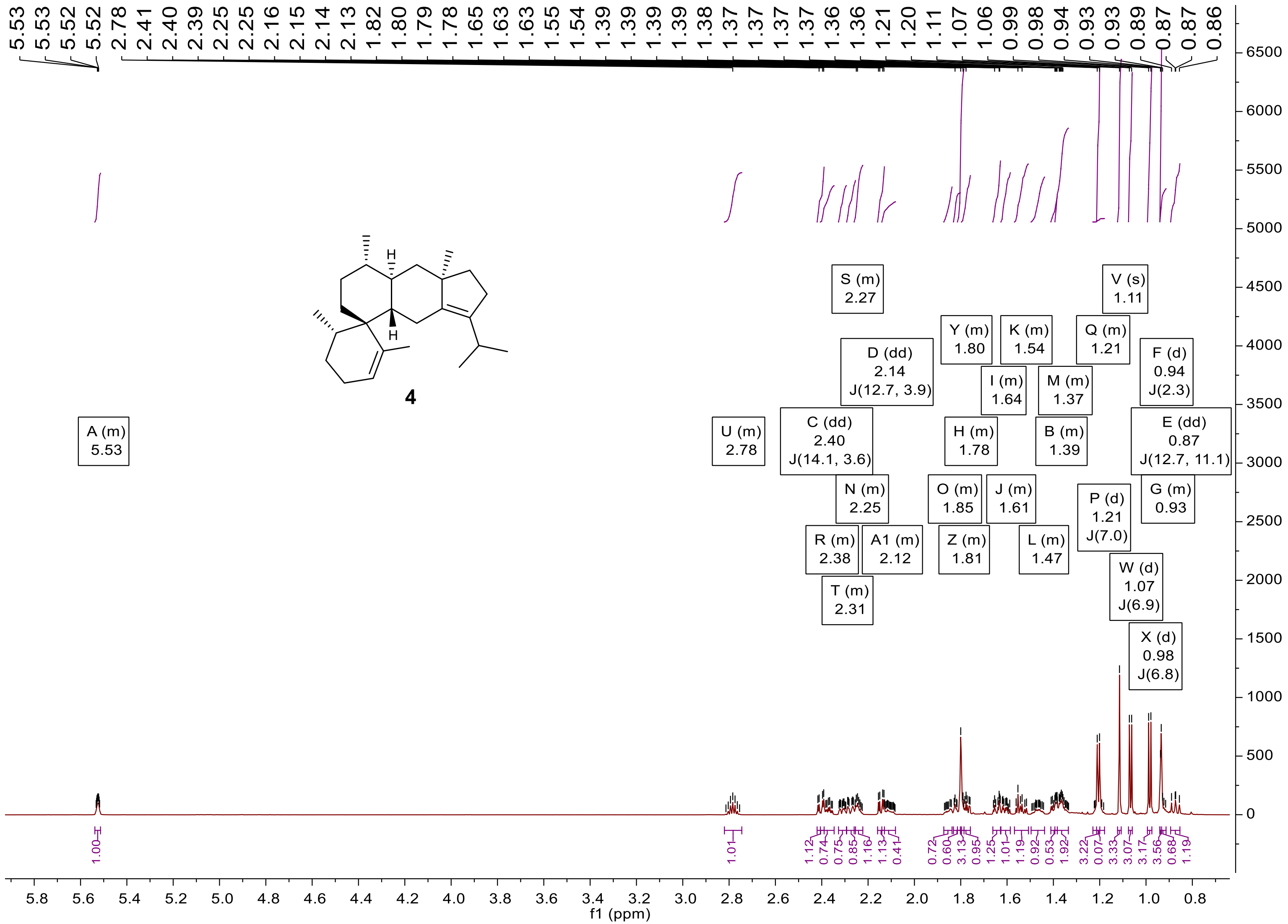


**Figure S39.** $^1$H-NMR spectrum of **4** (700 MHz, $C_6D_6$).

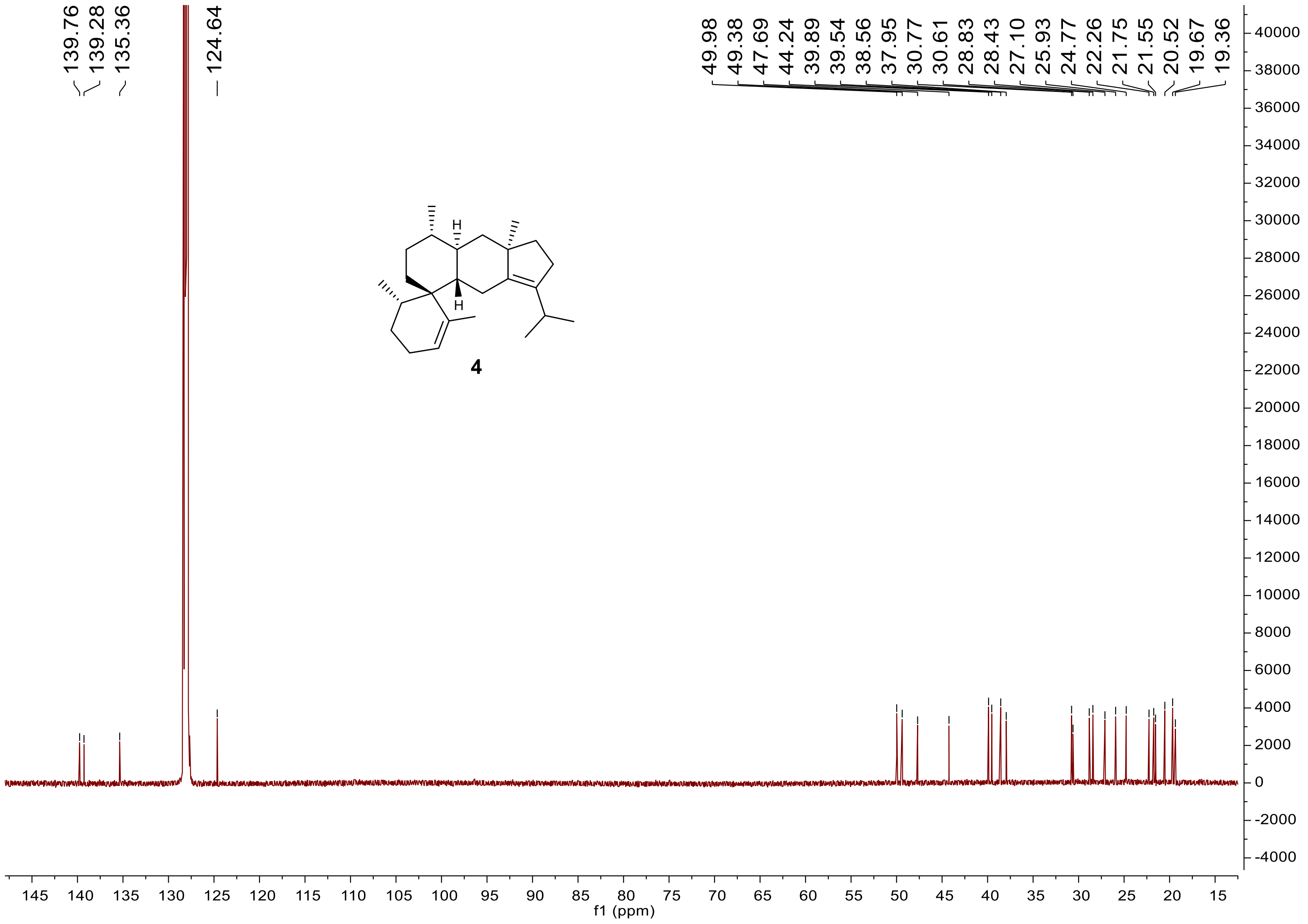


**Figure S40.** $^{13}$C-NMR spectrum of **4** (176 MHz, $C_6D_6$).

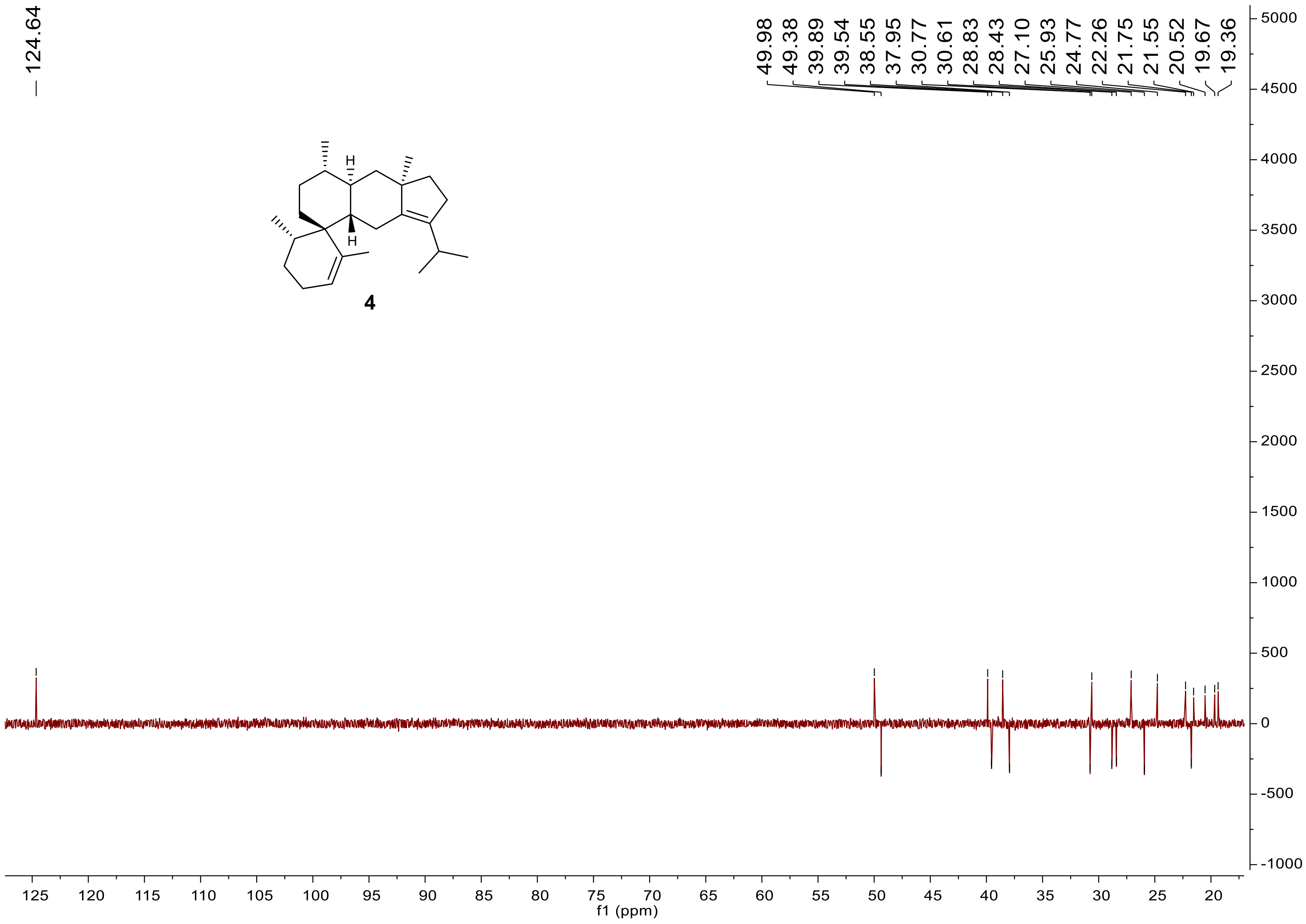


**Figure S41.** $^{13}$C-DEPT135 spectrum of **4** (176 MHz, $C_6D_6$).

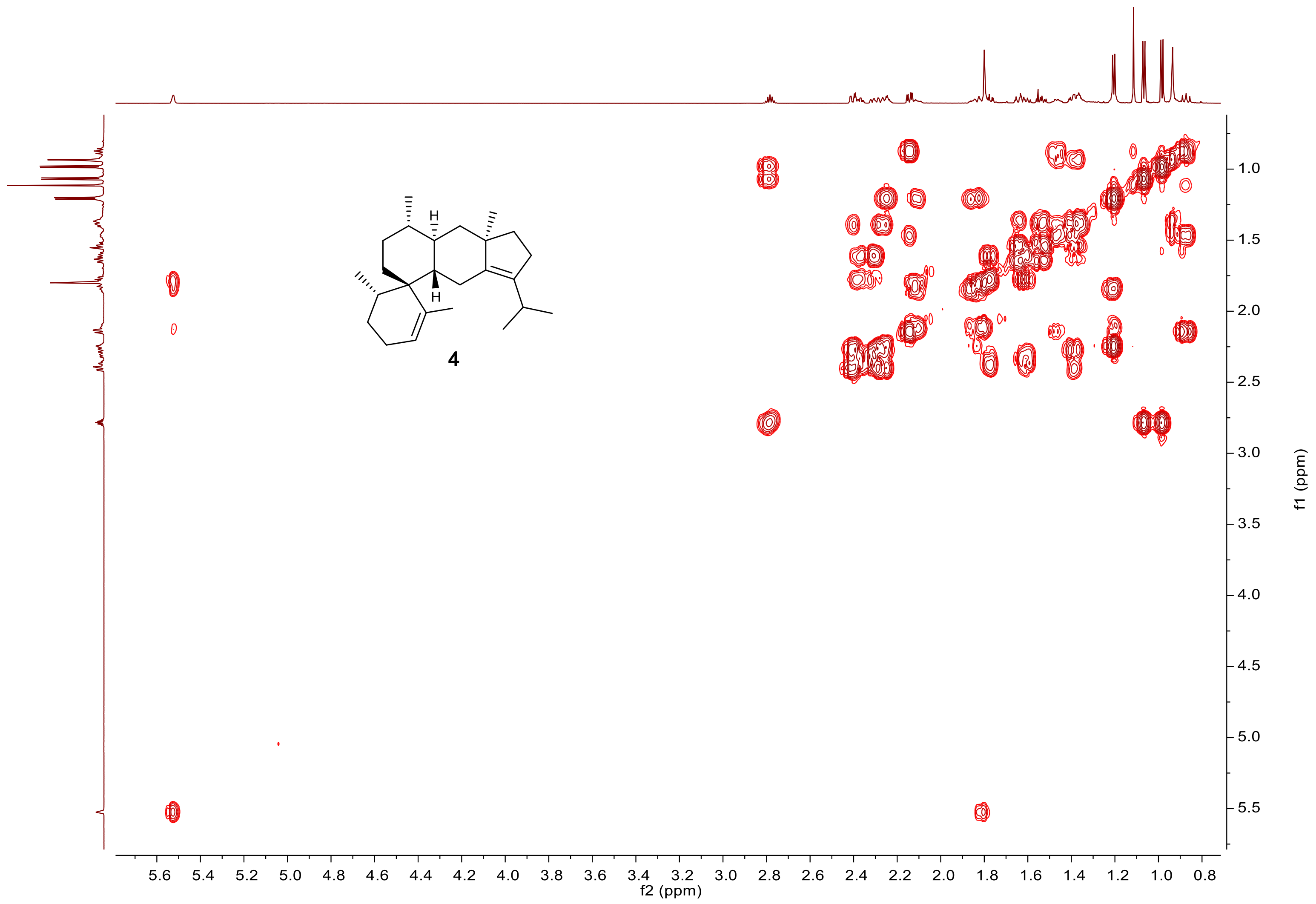


**Figure S42.** $^1H$-$^1H$-COSY spectrum ($C_6D_6$) of **4**.

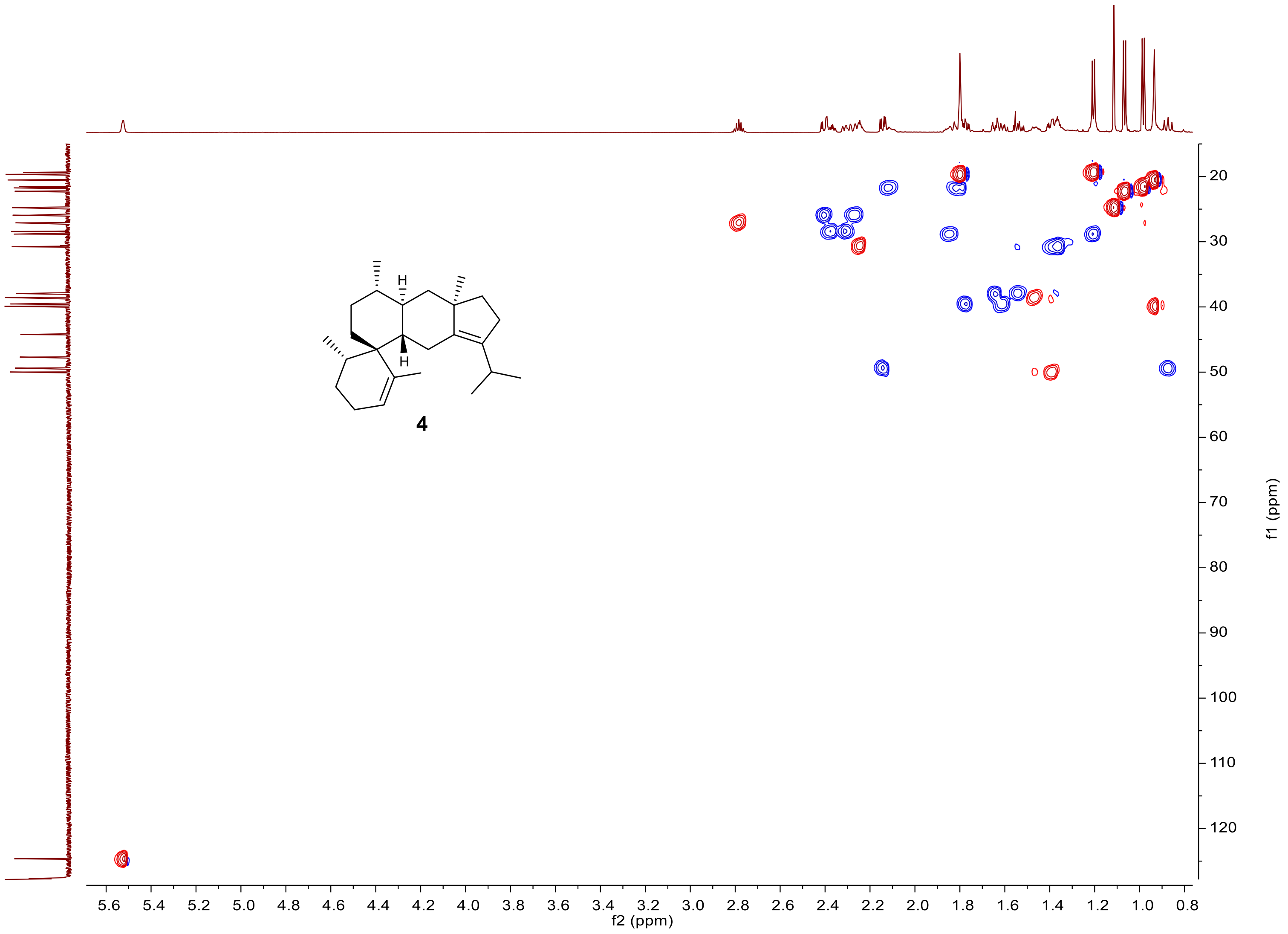


**Figure S43.** HSQC spectrum ($C_6D_6$) of **4**.

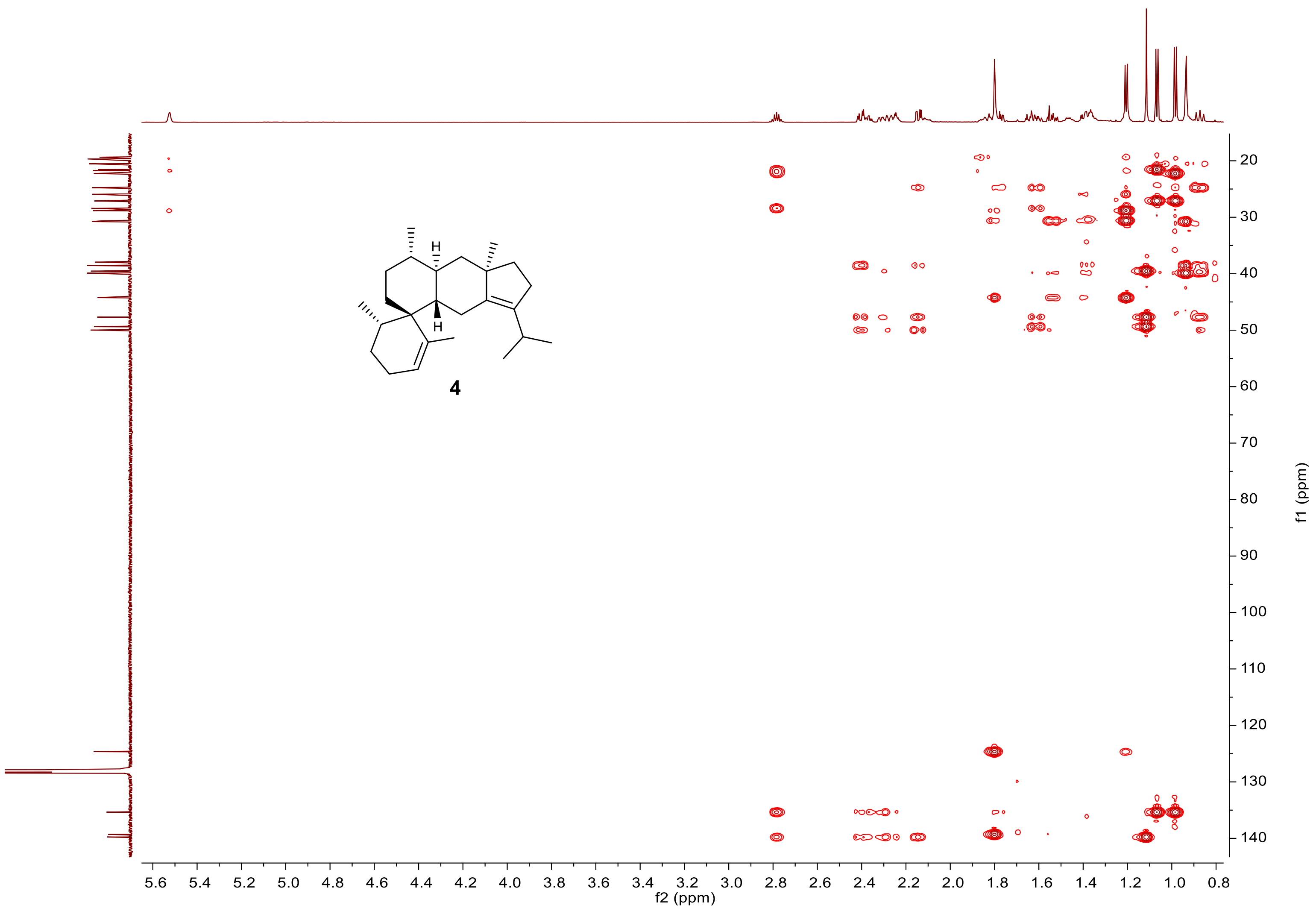


**Figure S44.** HMBC spectrum ($C_6D_6$) of **4**.

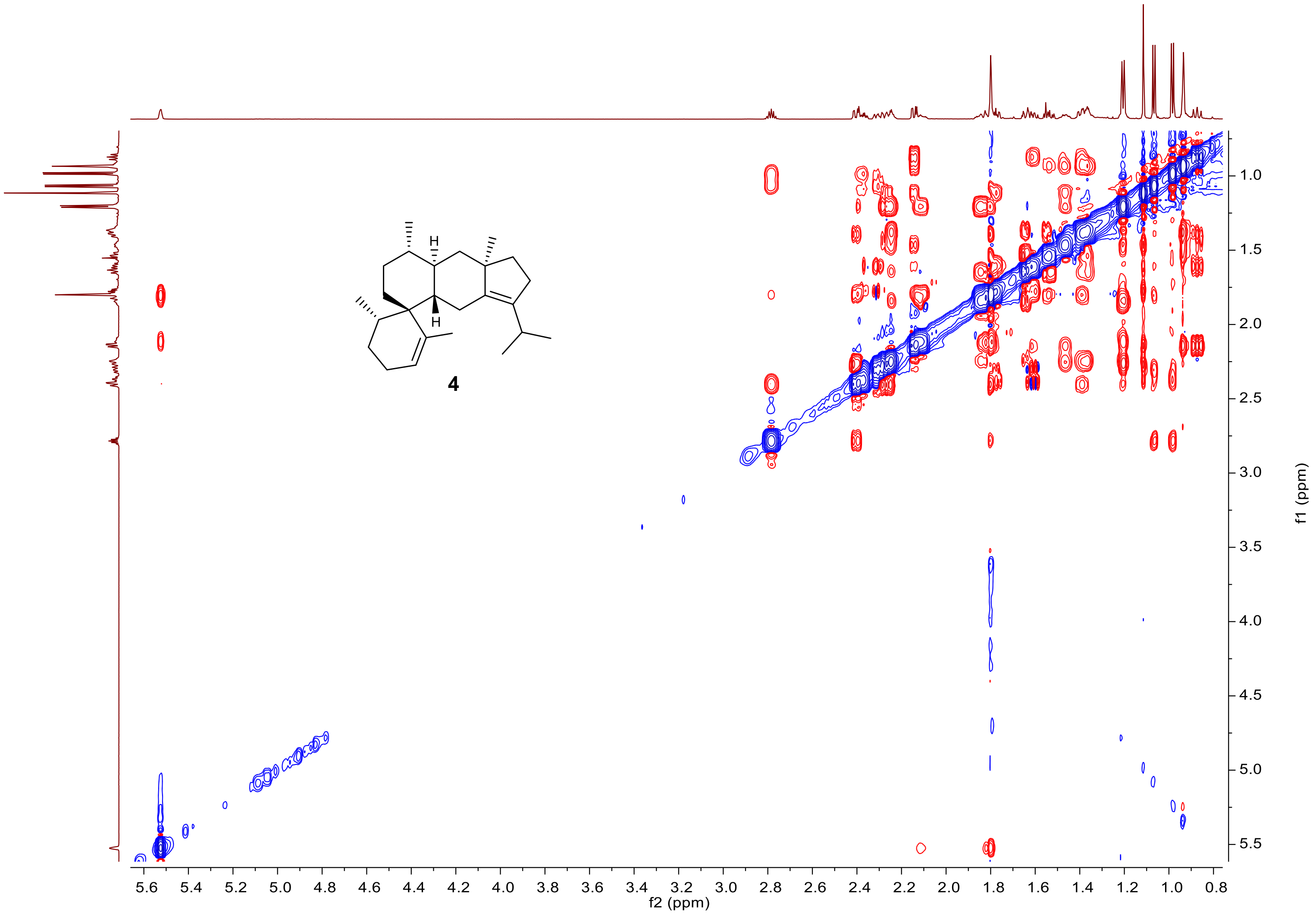


**Figure S45.** NOESY spectrum ($C_6D_6$) of **4**.

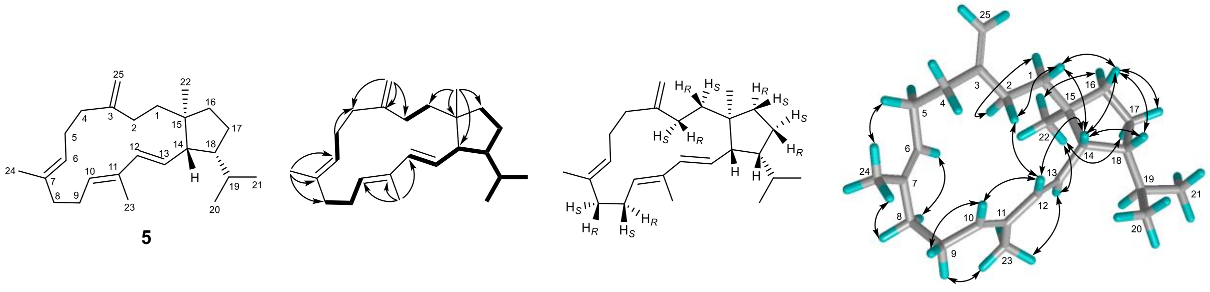

**Figure S46.** Structure elucidation of **5**. Bold: $^{1}H,^{1}H$-COSY, single headed arrows: key HMBC, and double headed arrows: NOESY correlations. Carbon numbering follows GFPP numbering to indicate the origin of each carbon. Diastereotopic hydrogens are labelled $H_R$ (*pro-R*) and $H_S$ (*pro-S*).

**Table S9.** NMR data of sesterviolene E (**5**) in $C_6D_6$ recorded at 298 K.

| C[a] | type | $^{1}H$[b] | $^{13}C$[b] |
|---|---|---|---|
| 1 | $CH_2$ | 1.54 (m, $H_R$)<br>1.47 (m, $H_S$) | 40.20 |
| 2 | $CH_2$ | 2.14 (m, $H_R$)<br>1.82 (m, $H_S$) | 30.78 |
| 3 | $C_q$ | – | 150.44 |
| 4 | $CH_2$ | 2.10 (m, 2H) | 37.72 |
| 5 | $CH_2$ | 2.10 (m, 2H) | 25.69 |
| 6 | CH | 5.08 (m) | 126.60 |
| 7 | $C_q$ | – | 133.10 |
| 8 | $CH_2$ | 2.07 (m, $H_R$)<br>1.99 (m, $H_S$) | 39.55 |
| 9 | $CH_2$ | 2.31 (m, $H_R$)<br>1.99 (m, $H_S$) | 25.11 |
| 10 | CH | 5.13 (dd, $J$ = 12.0, 3.9) | 131.39 |
| 11 | $C_q$ | – | 133.62 |
| 12 | CH | 6.05 (d, $J$ = 15.4) | 138.05 |
| 13 | CH | 5.58 (dd, $J$ = 15.4, 10.8) | 126.61 |
| 14 | CH | 2.36 (t, $J$ = 10.9) | 56.31 |
| 15 | $C_q$ | – | 46.31 |
| 16 | $CH_2$ | 1.36 (dd, $J$ = 11.6, 6.6, $H_R$)<br>1.31 (dd, $J$ = 12.6, 6.9, $H_S$) | 40.93 |
| 17 | $CH_2$ | 1.70 (m, $H_R$)<br>1.50 (m, $H_S$) | 28.94 |
| 18 | CH | 1.79 (m) | 49.90 |
| 19 | CH | 1.66 (m) | 31.06 |
| 20/21 | $CH_3$ | 1.03 (d, $J$ = 6.3) | 24.25 |
| 21/20 | $CH_3$ | 0.90 (d, $J$ = 6.5) | 21.84 |
| 22 | $CH_3$ | 0.80 (s) | 20.68 |
| 23 | $CH_3$ | 1.68 (t, $J$ = 1.2) | 12.51 |
| 24 | $CH_3$ | 1.40 (d, $J$ = 1.2) | 15.17 |
| 25 | $CH_2$ | 4.91 (m, 2H) | 108.25 |

[a] Carbon numbering as shown in Figure S46. [b] Chemical shifts $\delta$ in ppm, multiplicity: s = singlet, d = doublet, t = triplet, m = multiplet, coupling constants $J$ are given in Hertz.

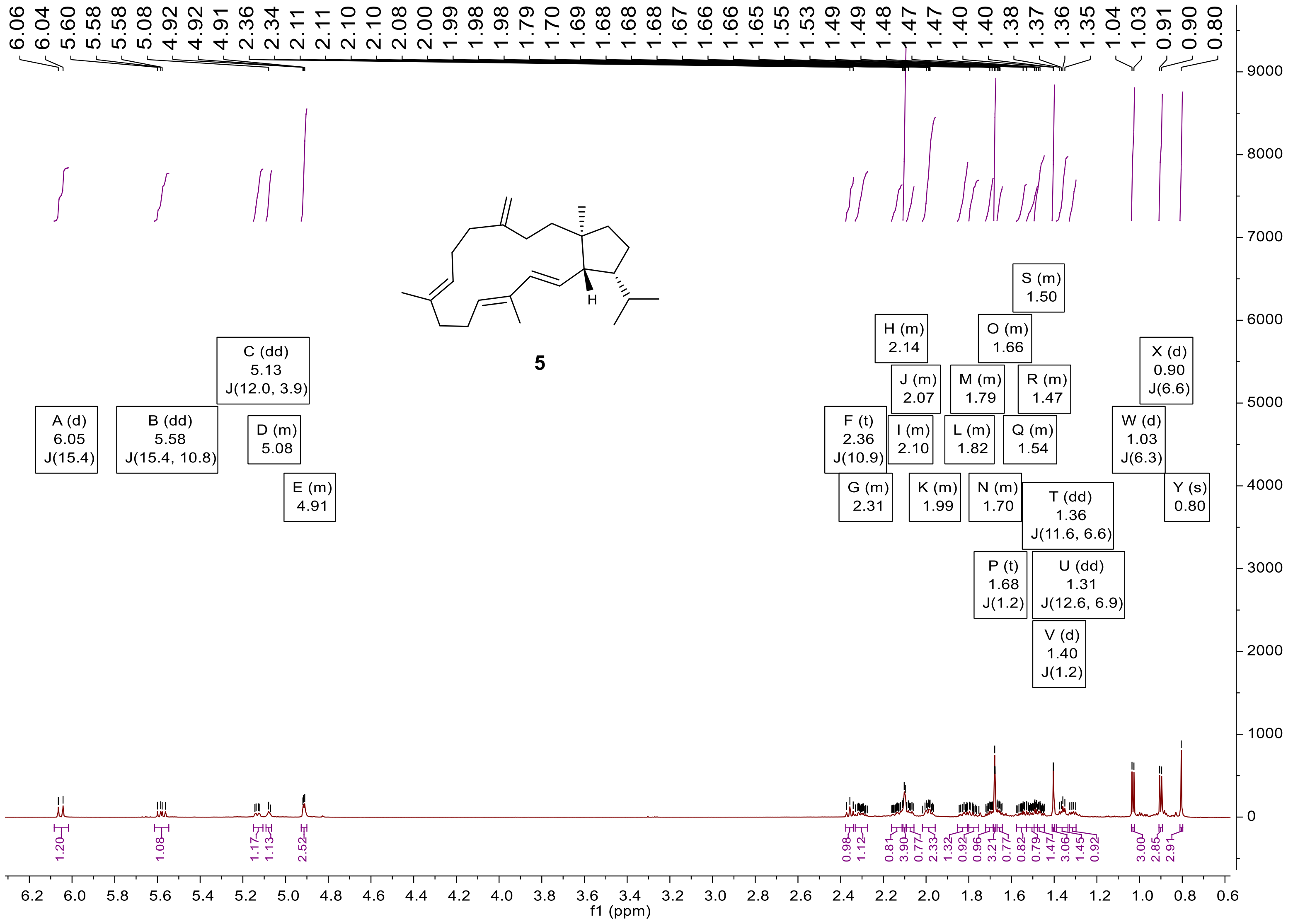


**Figure S47.** $^{1}$H-NMR spectrum of **5** (700 MHz, $C_6D_6$).

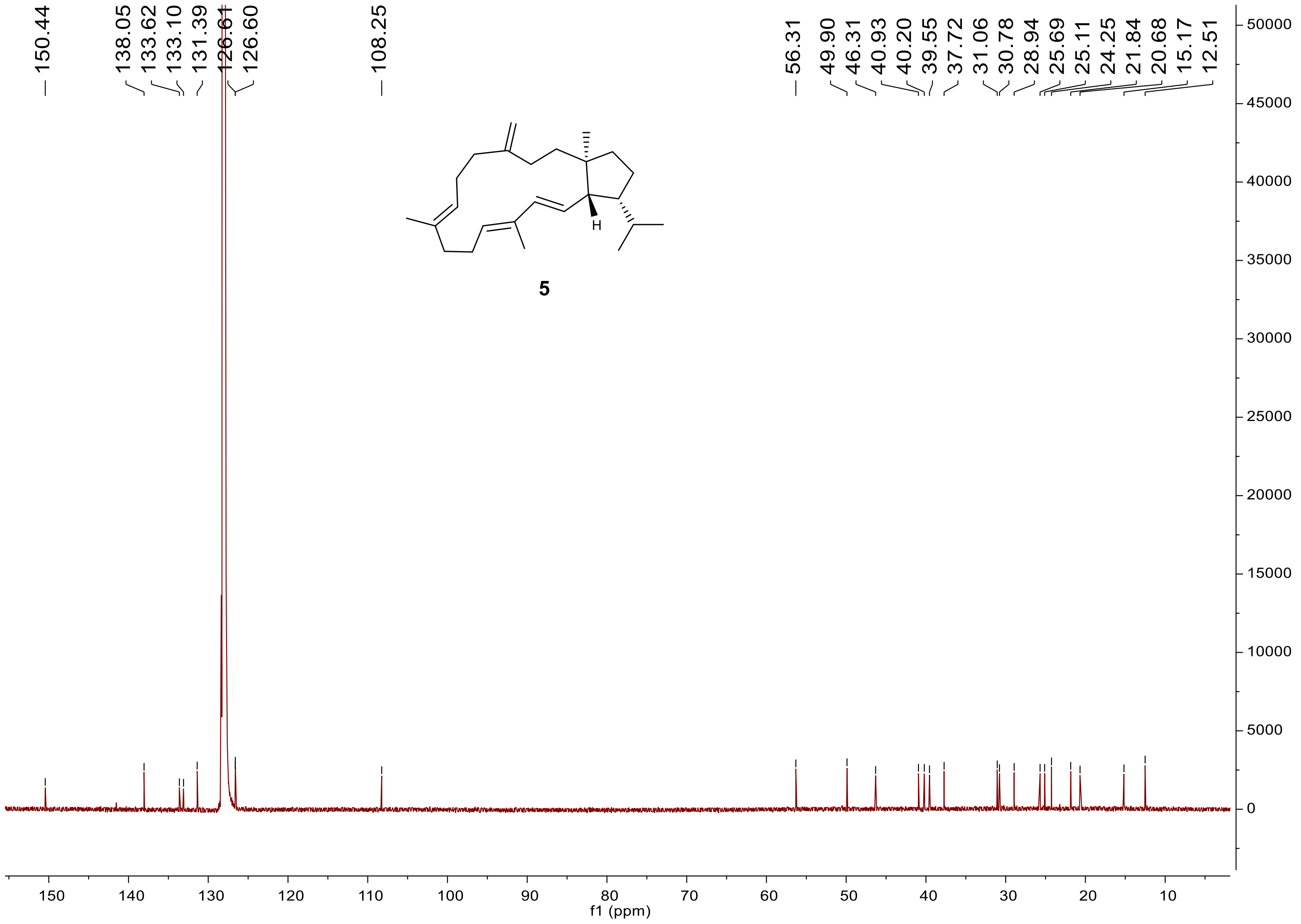


**Figure S48.** $^{13}$C-NMR spectrum of **5** (176 MHz, $C_6D_6$).

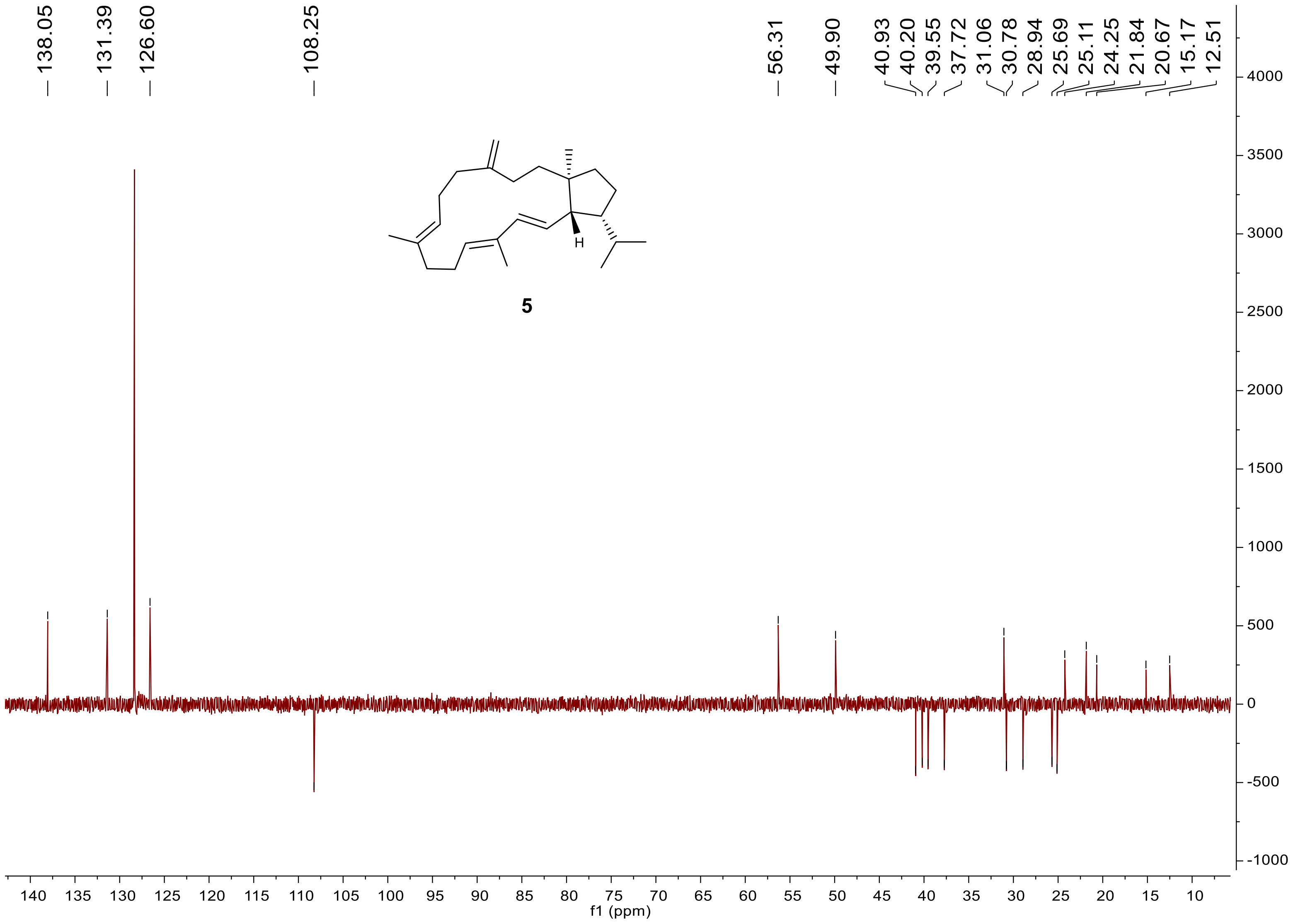


**Figure S49.** $^{13}$C-DEPT135 spectrum of **5** (176 MHz, $C_6D_6$).

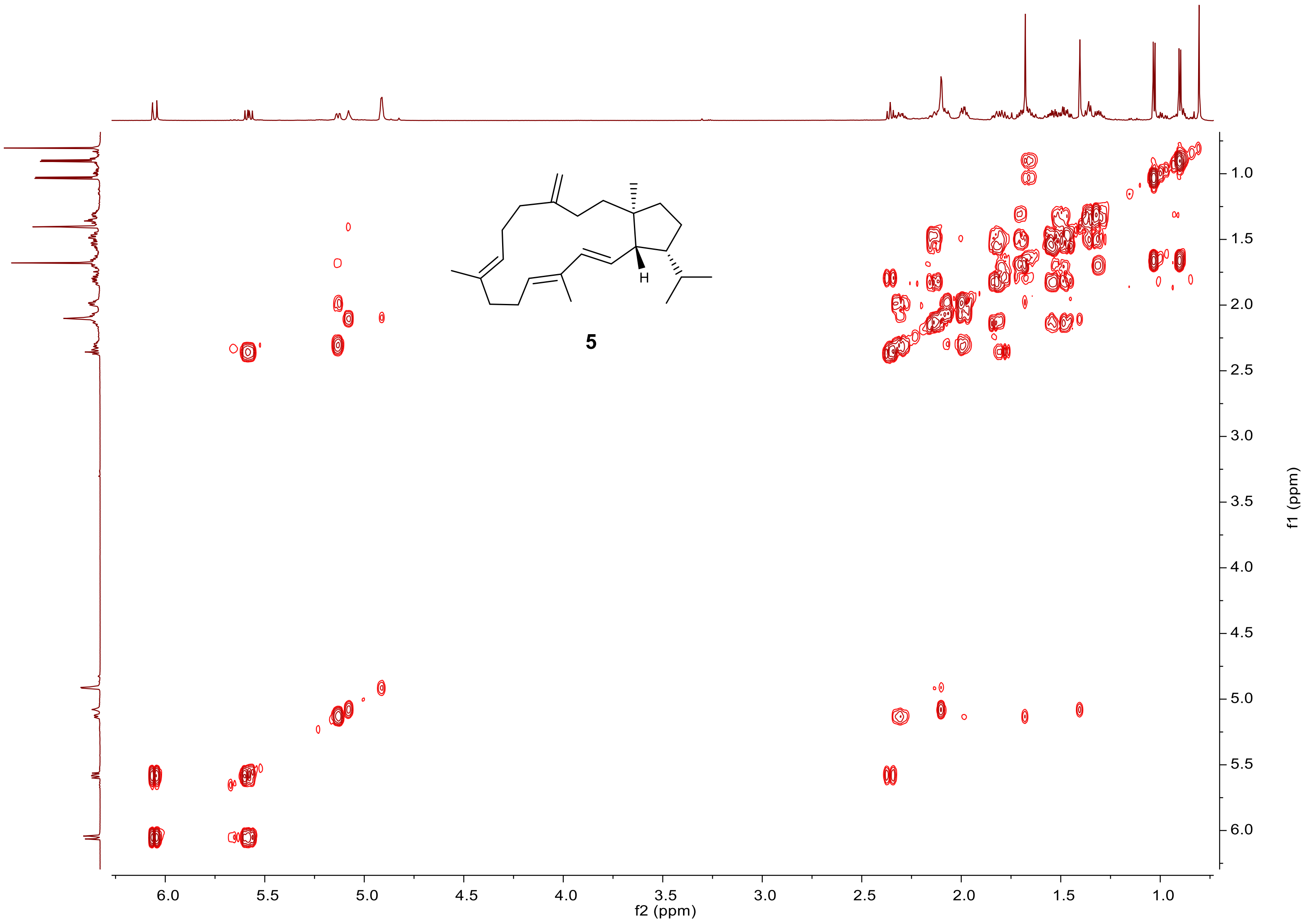


**Figure S50.** $^1H$-$^1H$-COSY spectrum ($C_6D_6$) of **5**.

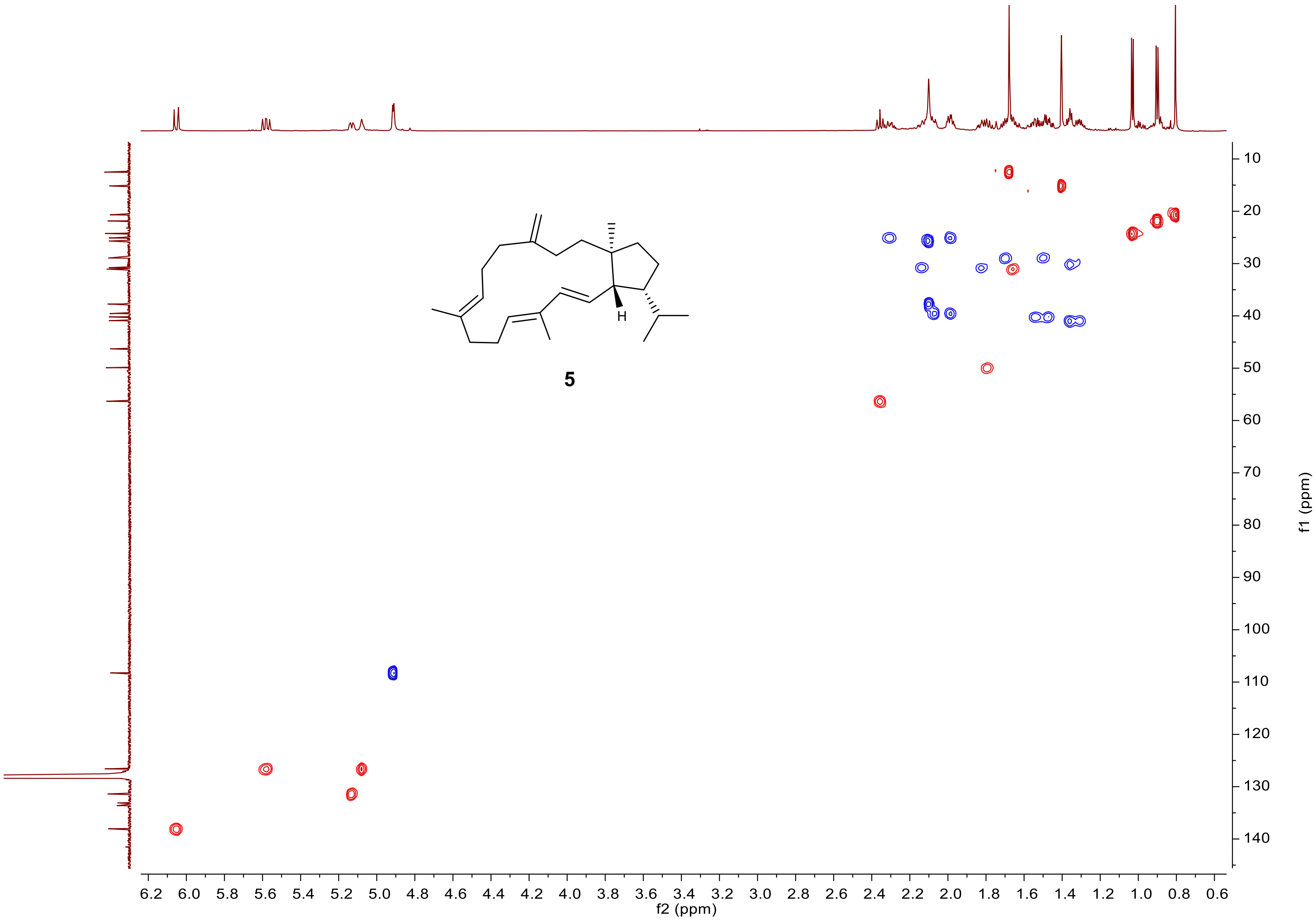


**Figure S51.** HSQC spectrum ($C_6D_6$) of **5**.

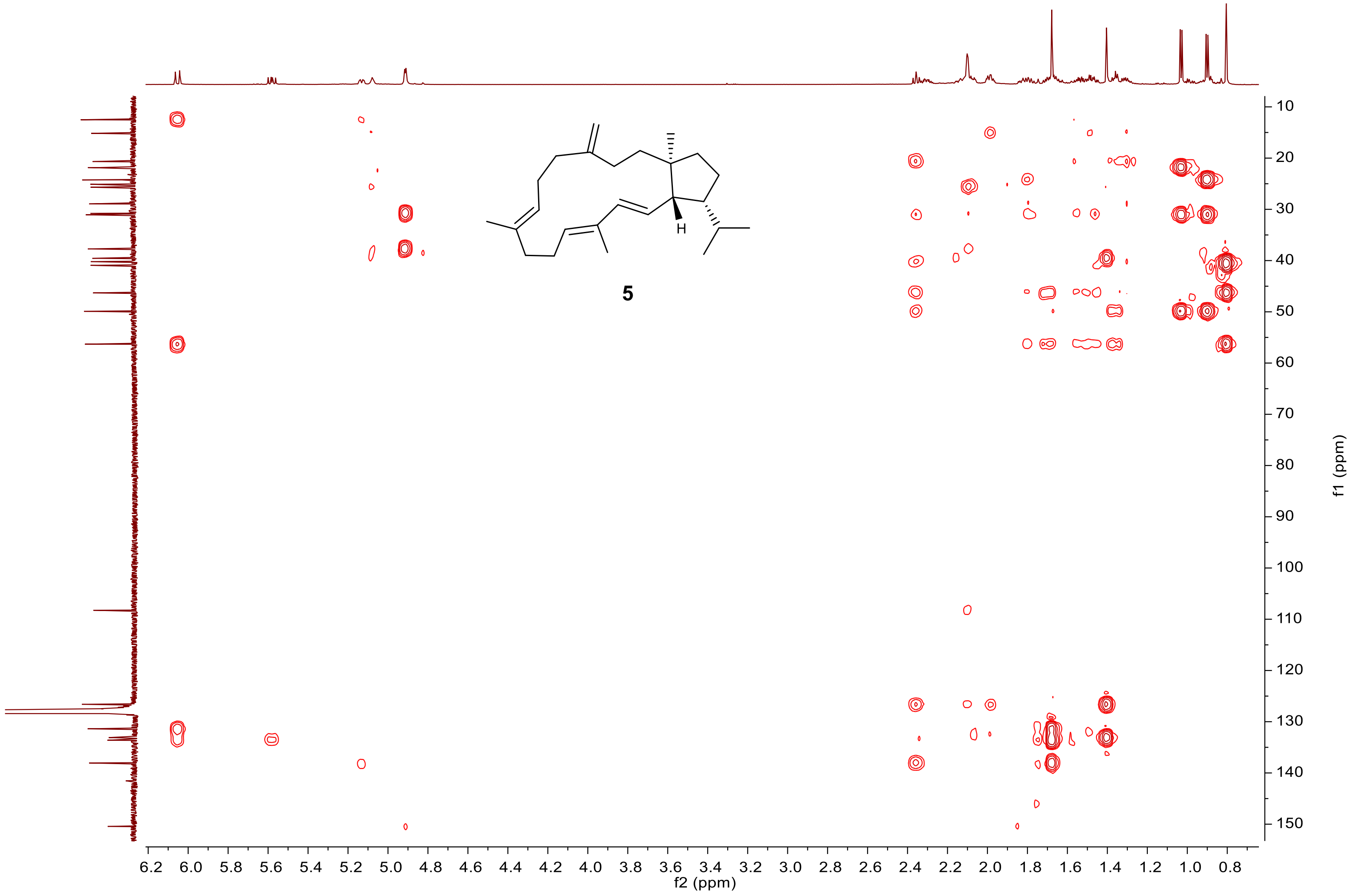


**Figure S52.** HMBC spectrum ($C_6D_6$) of **5**.

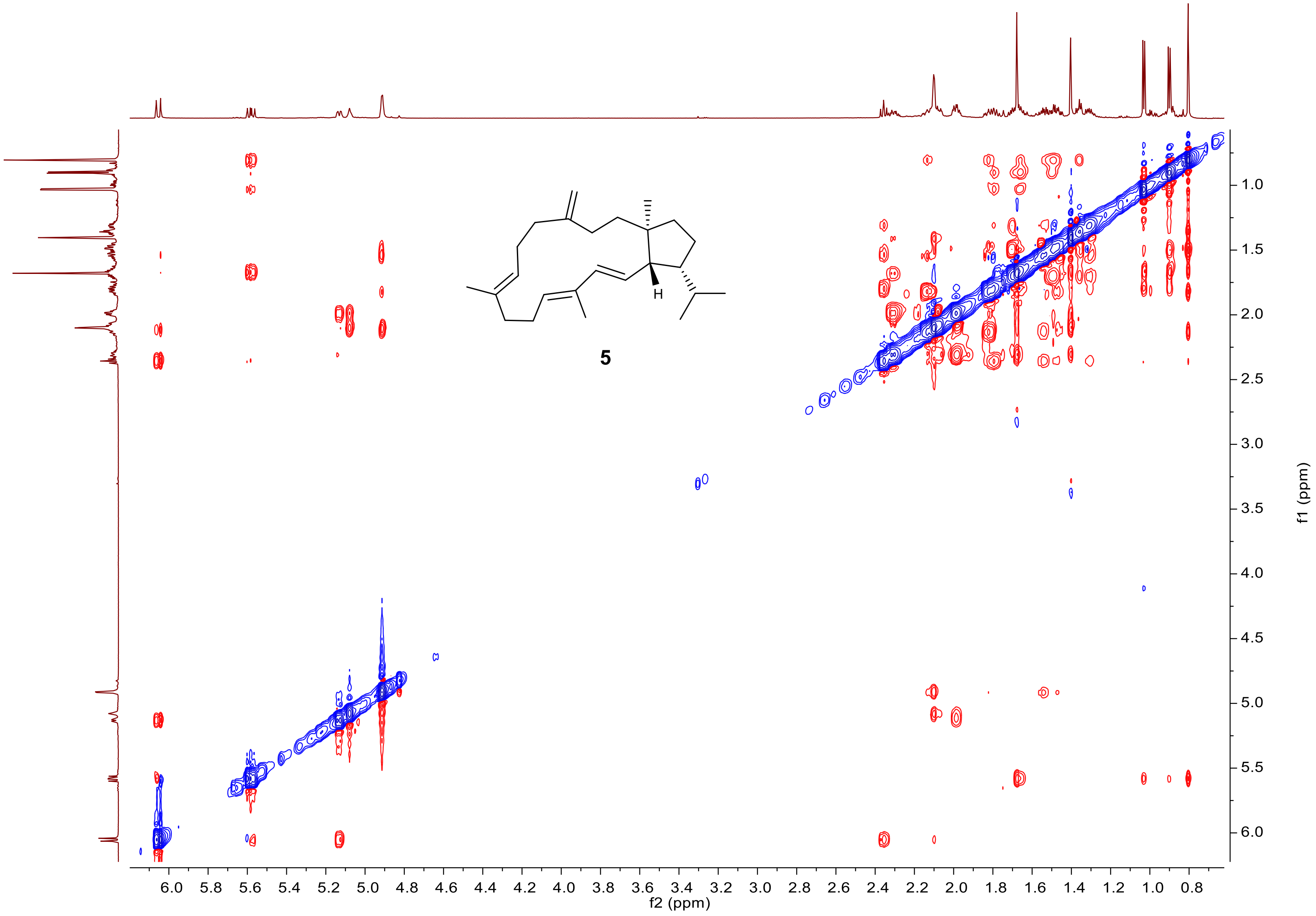


**Figure S53.** NOESY spectrum ($C_6D_6$) of **5**.

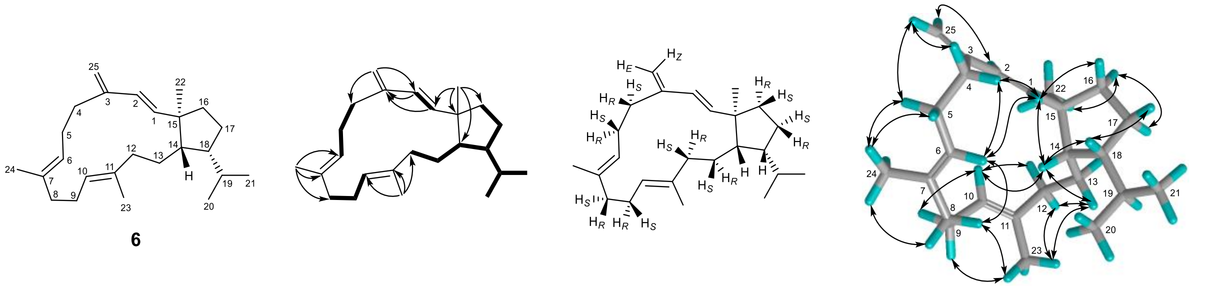

6

**Figure S54.** Structure elucidation of **6**. Bold: $^{1}$H,$^{1}$H-COSY, single headed arrows: key HMBC, and double headed arrows: NOESY correlations. Carbon numbering follows GFPP numbering to indicate the origin of each carbon. Diastereotopic hydrogens are labelled $H_R$ (*pro-R*) and $H_S$ (*pro-S*).

**Table S10.** NMR data of sesterviolene F (**6**) in $C_6D_6$ recorded at 298 K.

| C[a] | type | $^{1}$H[b] | $^{13}$C[b] |
|---|---|---|---|
| 1 | CH | 5.96 (d, *J* = 16.1) | 143.99 |
| 2 | CH | 6.13 (d, *J* = 16.1) | 126.86 |
| 3 | $C_q$ | – | 148.22 |
| 4 | $CH_2$ | 2.44 (ddd, *J* = 13.4, 9.7, 4.1, $H_S$) | 32.53 |
| | | 2.30 (dddd, *J* = 13.2, 6.6, 3.8, 1.1, $H_R$) | |
| 5 | $CH_2$ | 2.37 (m, $H_R$) | 29.14 |
| | | 2.19 (m, $H_S$) | |
| 6 | CH | 5.32 (m) | 126.78 |
| 7 | $C_q$ | – | 133.94 |
| 8 | $CH_2$ | 2.13 (m, $H_S$) | 39.72 |
| | | 2.09 (m, $H_R$) | |
| 9 | $CH_2$ | 2.23 (m, $H_S$) | 24.92 |
| | | 2.09 (m, $H_R$) | |
| 10 | CH | 5.20 (m) | 127.56 |
| 11 | $C_q$ | – | 132.93 |
| 12 | $CH_2$ | 2.22 (m, $H_S$) | 38.53 |
| | | 2.03 (m, $H_R$) | |
| 13 | $CH_2$ | 1.41 (ddt, *J* = 15.3, 12.6, 2.9, $H_S$) | 21.18 |
| | | 1.20 (m, $H_R$) | |
| 14 | CH | 1.96 (ddd, *J* = 10.3, 7.5, 2.7) | 44.98 |
| 15 | $C_q$ | – | 47.57 |
| 16 | $CH_2$ | 1.74 (dt, *J* = 13.0, 8.5, $H_S$) | 38.05 |
| | | 1.46 (ddd, *J* = 12.6, 9.5, 2.8, $H_R$) | |
| 17 | $CH_2$ | 1.67 (m, $H_S$) | 28.29 |
| | | 1.24 (m, $H_R$) | |
| 18 | CH | 1.71 (m) | 50.97 |
| 19 | CH | 1.50 (m) | 28.94 |
| 20/21 | $CH_3$ | 0.83 (d, *J* = 6.5) | 22.83 |
| 2120 | $CH_3$ | 0.87 (d, *J* = 6.6) | 22.00 |
| 22 | $CH_3$ | 1.14 (s) | 24.20 |
| 23 | $CH_3$ | 1.60 (s) | 16.26 |
| 24 | $CH_3$ | 1.51 (d, *J* = 1.3) | 15.33 |
| 25 | $CH_2$ | 5.07 (d, *J* = 2.2, $H_Z$) | 113.01 |
| | | 4.93 (m, $H_E$) | |

[a] Carbon numbering as shown in Figure S54. [b] Chemical shifts $\delta$ in ppm, multiplicity: s = singlet, d = doublet, t = triplet, m = multiplet, coupling constants *J* are given in Hertz.

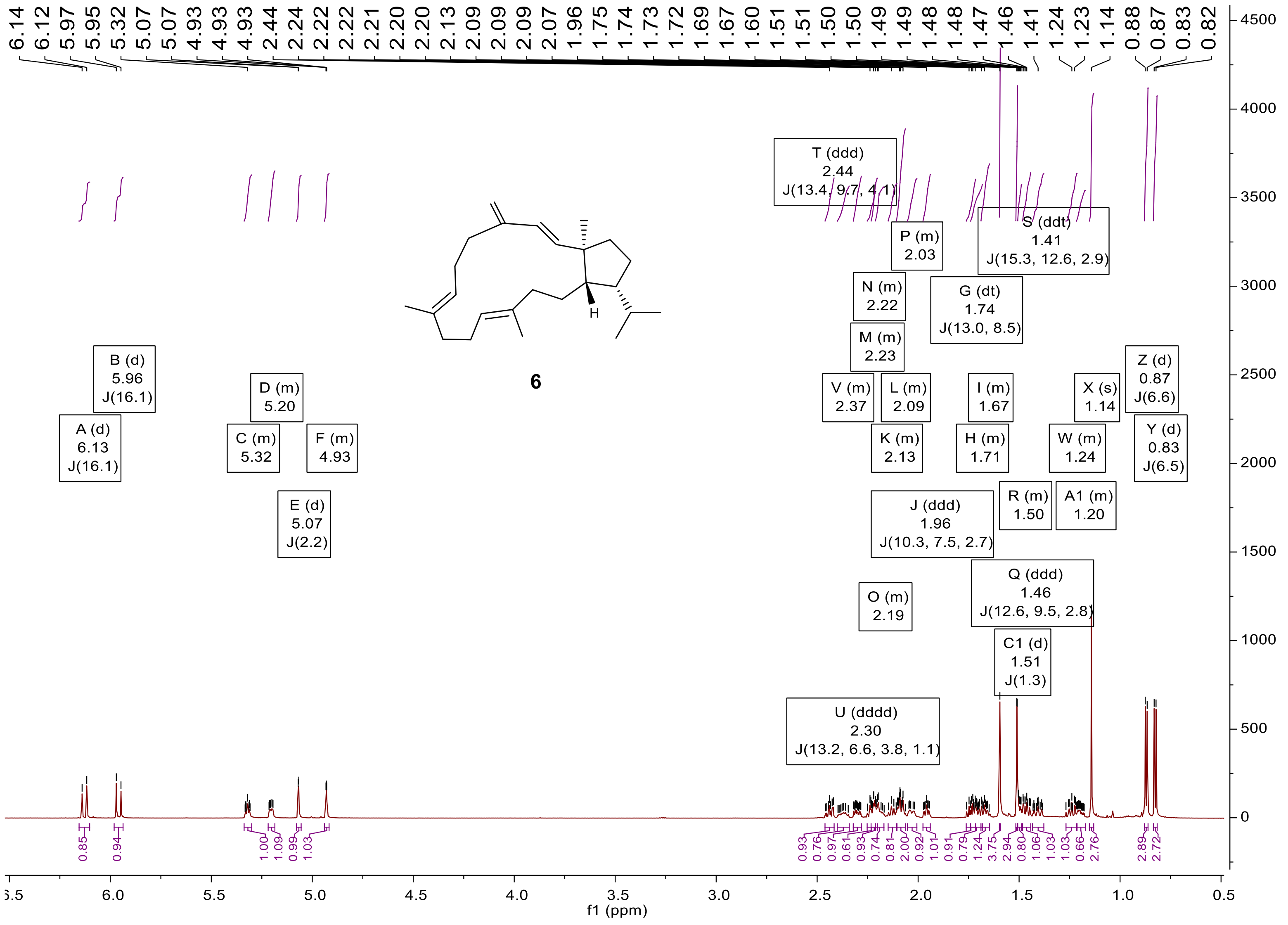


**Figure S55.** $^{1}$H-NMR spectrum of **6** (700 MHz, $C_6D_6$).

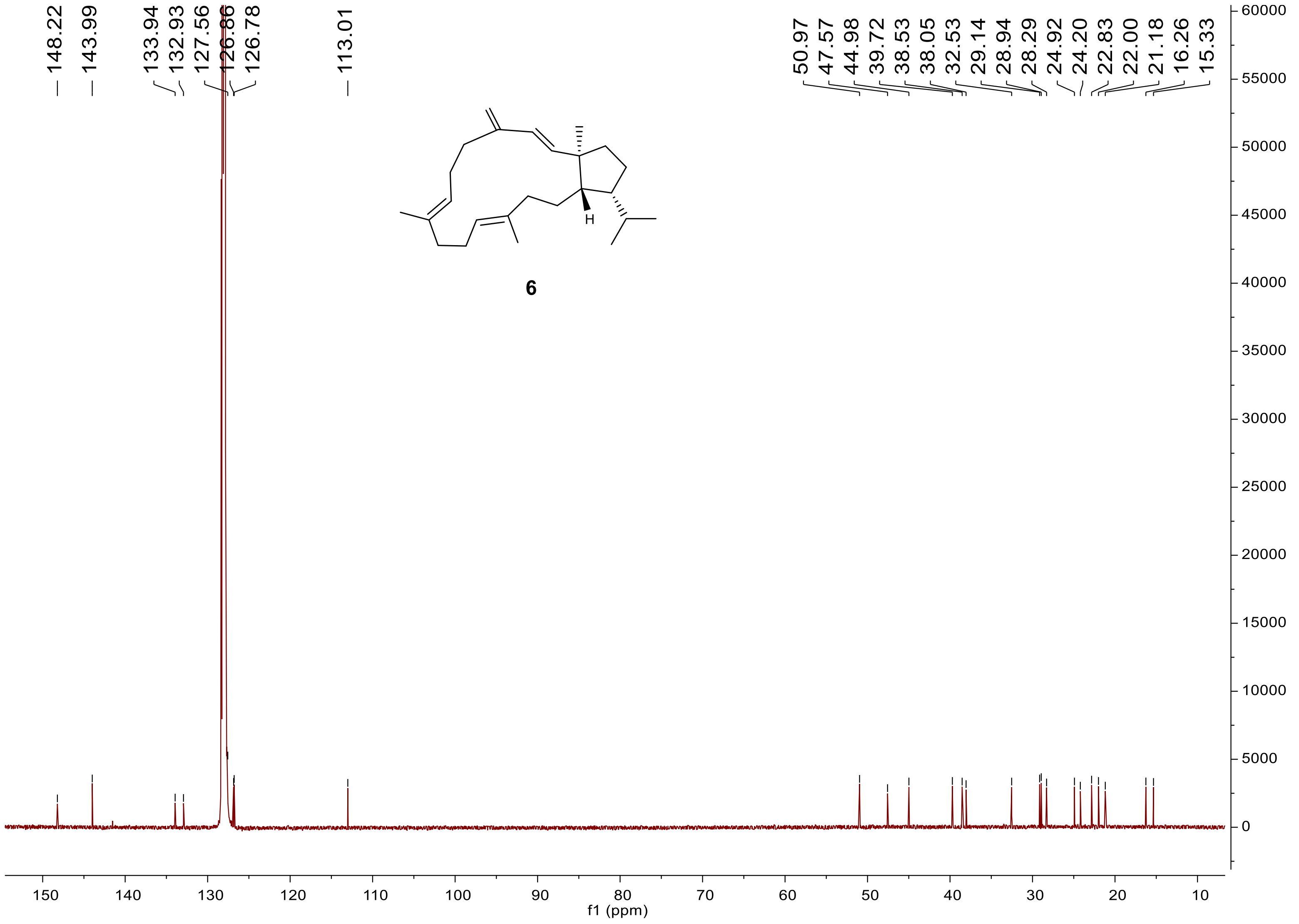


**Figure S56.** $^{13}$C-NMR spectrum of **6** (176 MHz, $C_6D_6$).

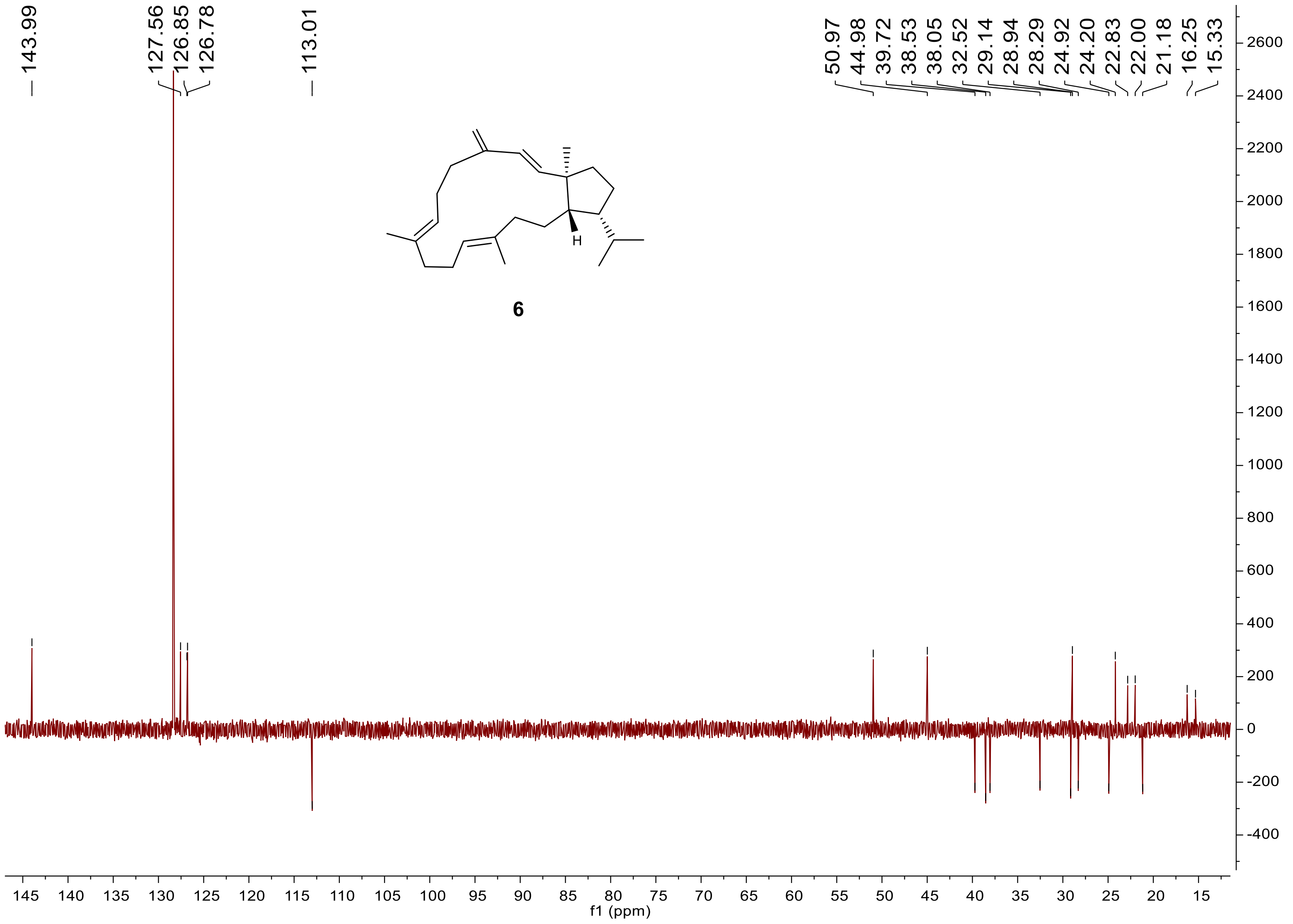


**Figure S57.** $^{13}$C-DEPT135 spectrum of **6** (176 MHz, $C_6D_6$).

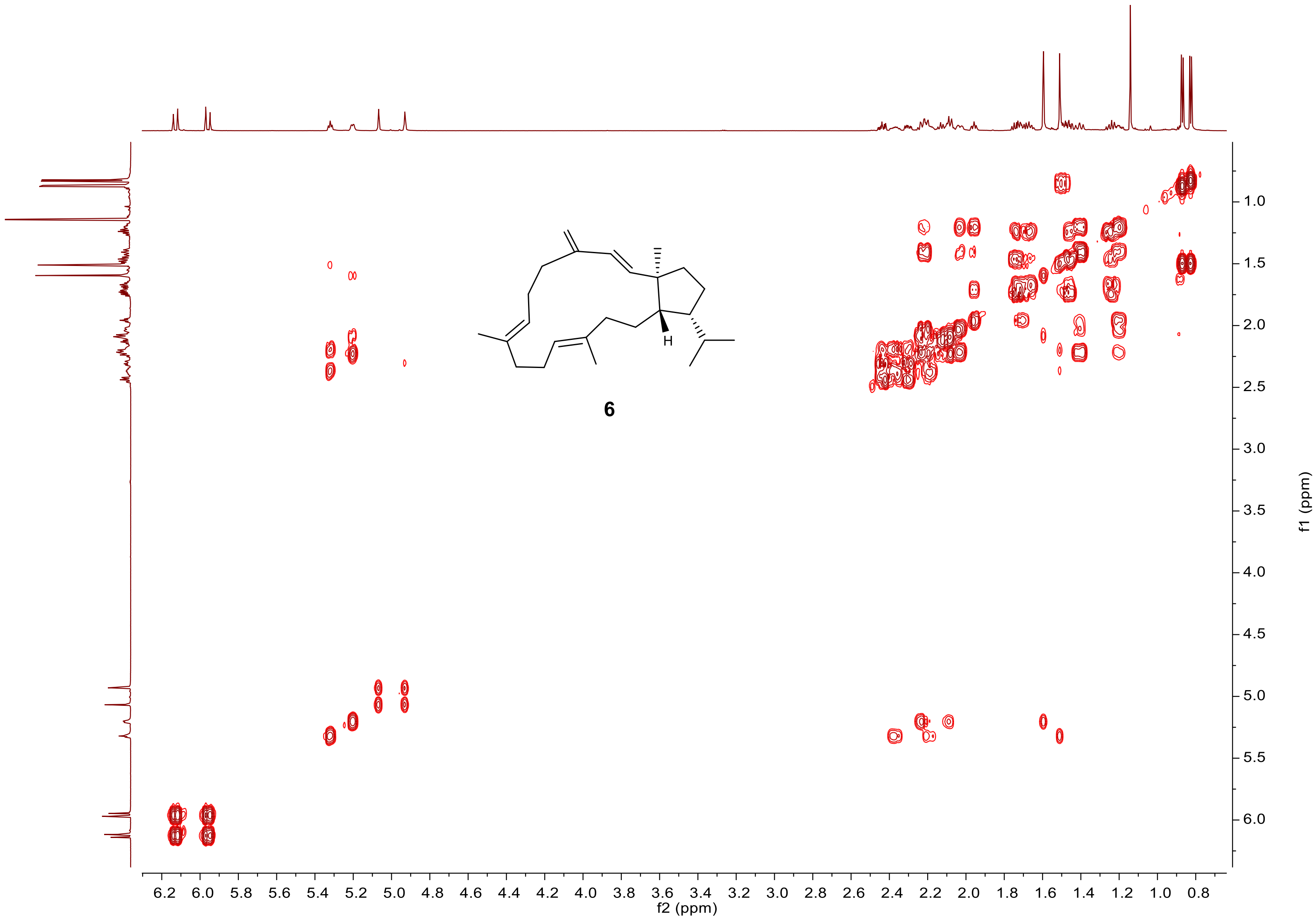


**Figure S58.** $^1H$-$^1H$-COSY spectrum ($C_6D_6$) of **6**.

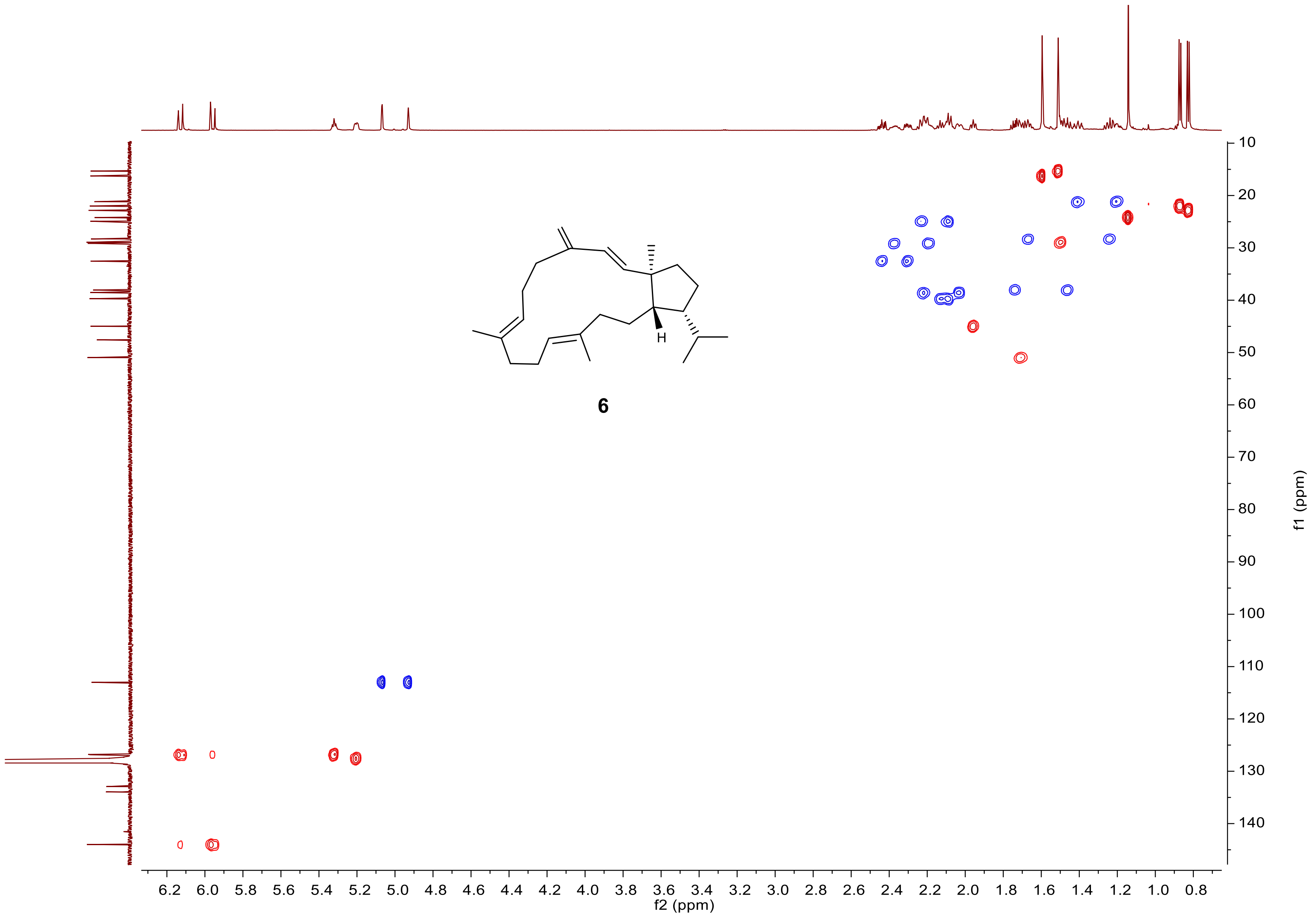


**Figure S59.** HSQC spectrum ($C_6D_6$) of **6**.

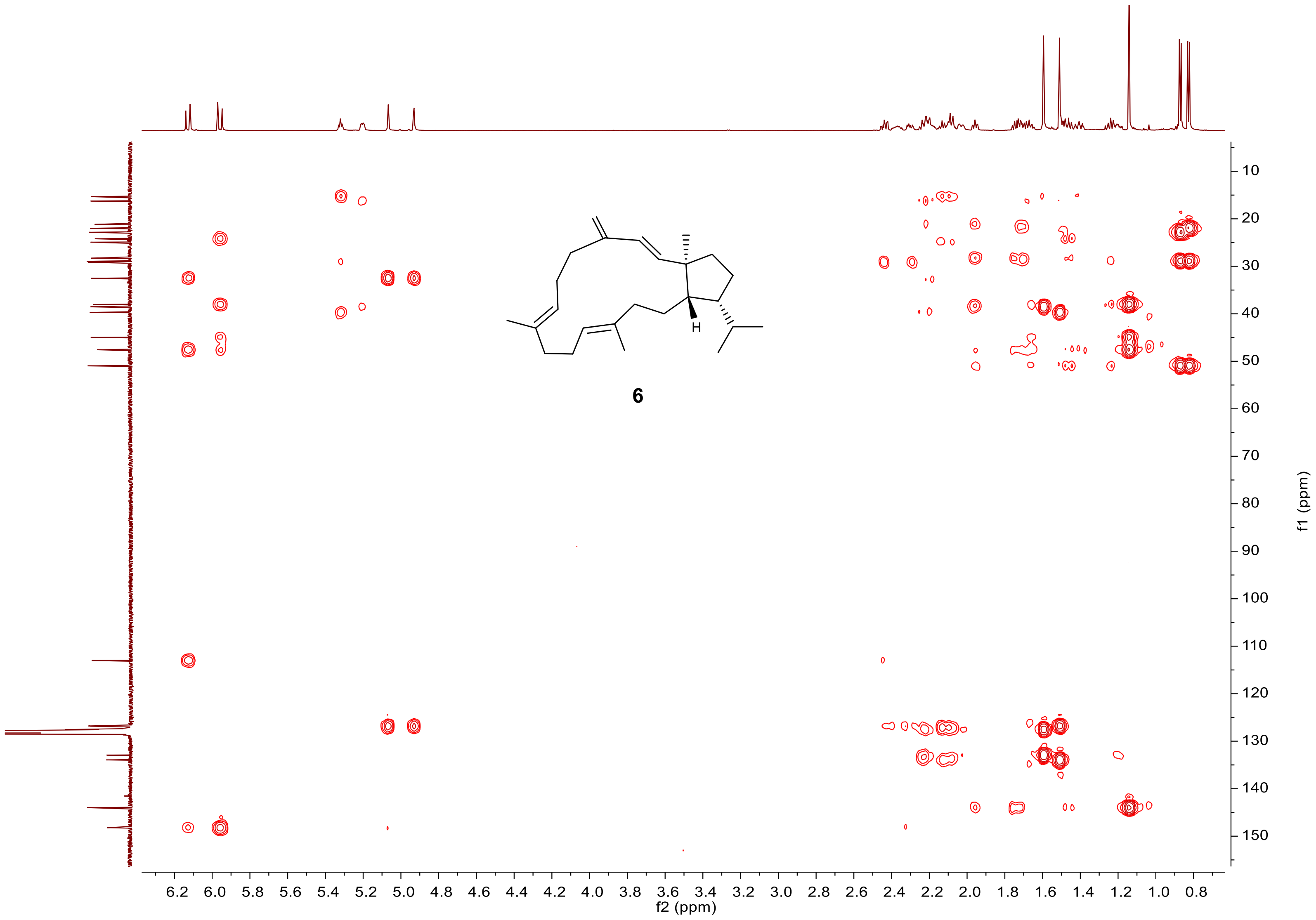


**Figure S60.** HMBC spectrum ($C_6D_6$) of **6**.

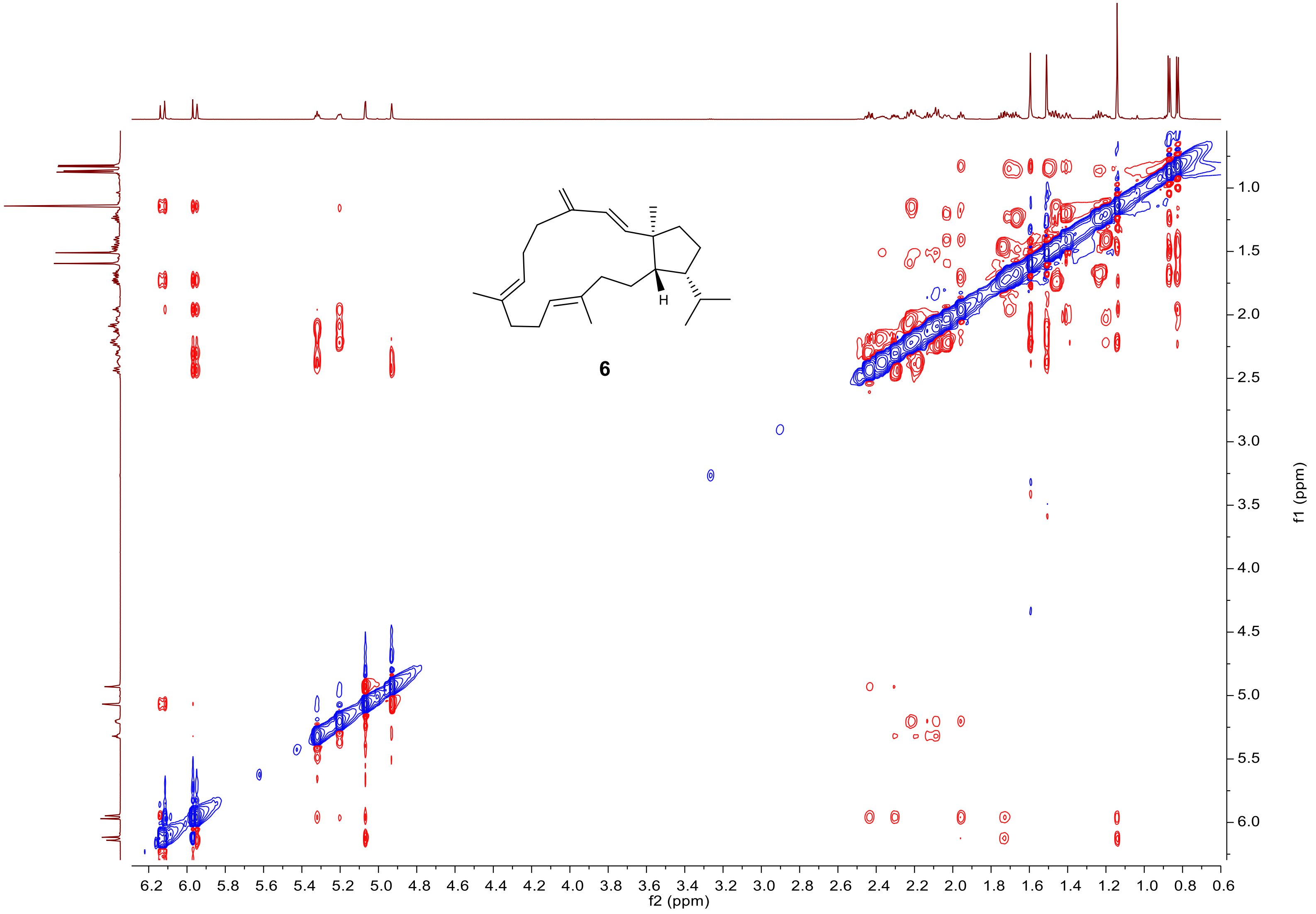


**Figure S61.** NOESY spectrum ($C_6D_6$) of **6**.

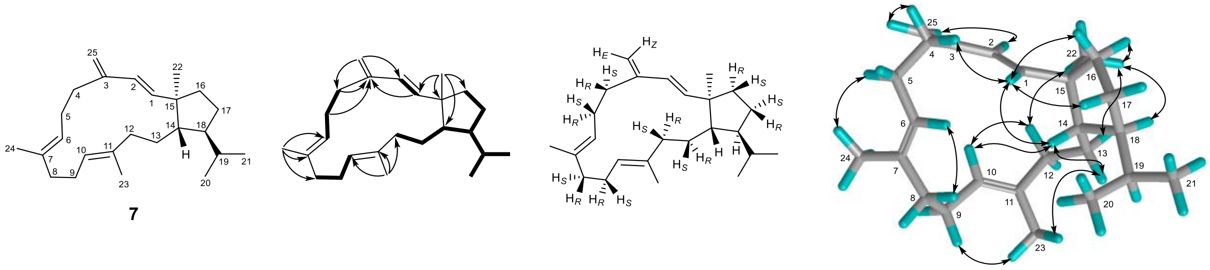


**Figure S62.** Structure elucidation of **7**. Bold: $^{1}H,^{1}H$-COSY, single headed arrows: key HMBC, and double headed arrows: NOESY correlations. Carbon numbering follows GFPP numbering to indicate the origin of each carbon. Diastereotopic hydrogens are labelled $H_R$ (*pro-R*) and $H_S$ (*pro-S*).

**Table S11.** NMR data of sesterviolene G (**7**) in $C_6D_6$ recorded at 298 K.

| C[a] | type | $^{1}H$[b] | $^{13}C$[b] |
|---|---|---|---|
| 1 | CH | 5.94 (d, *J* = 16.1) | 141.74 |
| 2 | CH | 6.17 (d, *J* = 16.1) | 128.49 |
| 3 | $C_q$ | – | 147.31 |
| 4 | $CH_2$ | 2.38 (m, $H_R$) | 31.25 |
| | | 2.29 (m, $H_S$) | |
| 5 | $CH_2$ | 2.30 (m, 2H) | 28.46 |
| 6 | CH | 5.36 (m) | 125.65 |
| 7 | $C_q$ | – | 133.67 |
| 8 | $CH_2$ | 2.07 (m, 2H) | 39.42 |
| 9 | $CH_2$ | 2.14 (m, 2H) | 24.54 |
| 10 | CH | 5.26 (tq, *J* = 6.6, 1.2) | 124.68 |
| 11 | $C_q$ | – | 133.32 |
| 12 | $CH_2$ | 2.25 (m, $H_S$) | 37.33 |
| | | 1.99 (m, $H_R$) | |
| 13 | $CH_2$ | 1.50 (m, $H_R$) | 28.03 |
| | | 1.42 (m, $H_S$) | |
| 14 | CH | 1.64 (m) | 47.70 |
| 15 | $C_q$ | – | 47.10 |
| 16 | $CH_2$ | 1.59 (m, $H_S$) | 41.71 |
| | | 1.43 (m, $H_R$) | |
| 17 | $CH_2$ | 1.56 (m, $H_R$) | 23.92 |
| | | 1.38 (m, $H_S$) | |
| 18 | CH | 1.53 (m) | 51.45 |
| 19 | CH | 1.70 (m) | 30.09 |
| 20/21 | $CH_3$ | 0.82 (d, *J* = 6.8) | 16.67 |
| 21/20 | $CH_3$ | 0.92 (d, *J* = 6.8) | 22.87 |
| 22 | $CH_3$ | 1.00 (s) | 19.63 |
| 23 | $CH_3$ | 1.57 (d, *J* = 1.2) | 16.47 |
| 24 | $CH_3$ | 1.54 (d, *J* = 1.3) | 15.62 |
| 25 | $CH_2$ | 5.14 (d, *J* = 1.9, $H_Z$) | 113.37 |
| | | 4.99 (d, *J* = 1.9, $H_E$) | |

[a] Carbon numbering as shown in Figure S62. [b] Chemical shifts $\delta$ in ppm, multiplicity: s = singlet, d = doublet, t = triplet, q = quartet, m = multiplet, coupling constants *J* are given in Hertz.

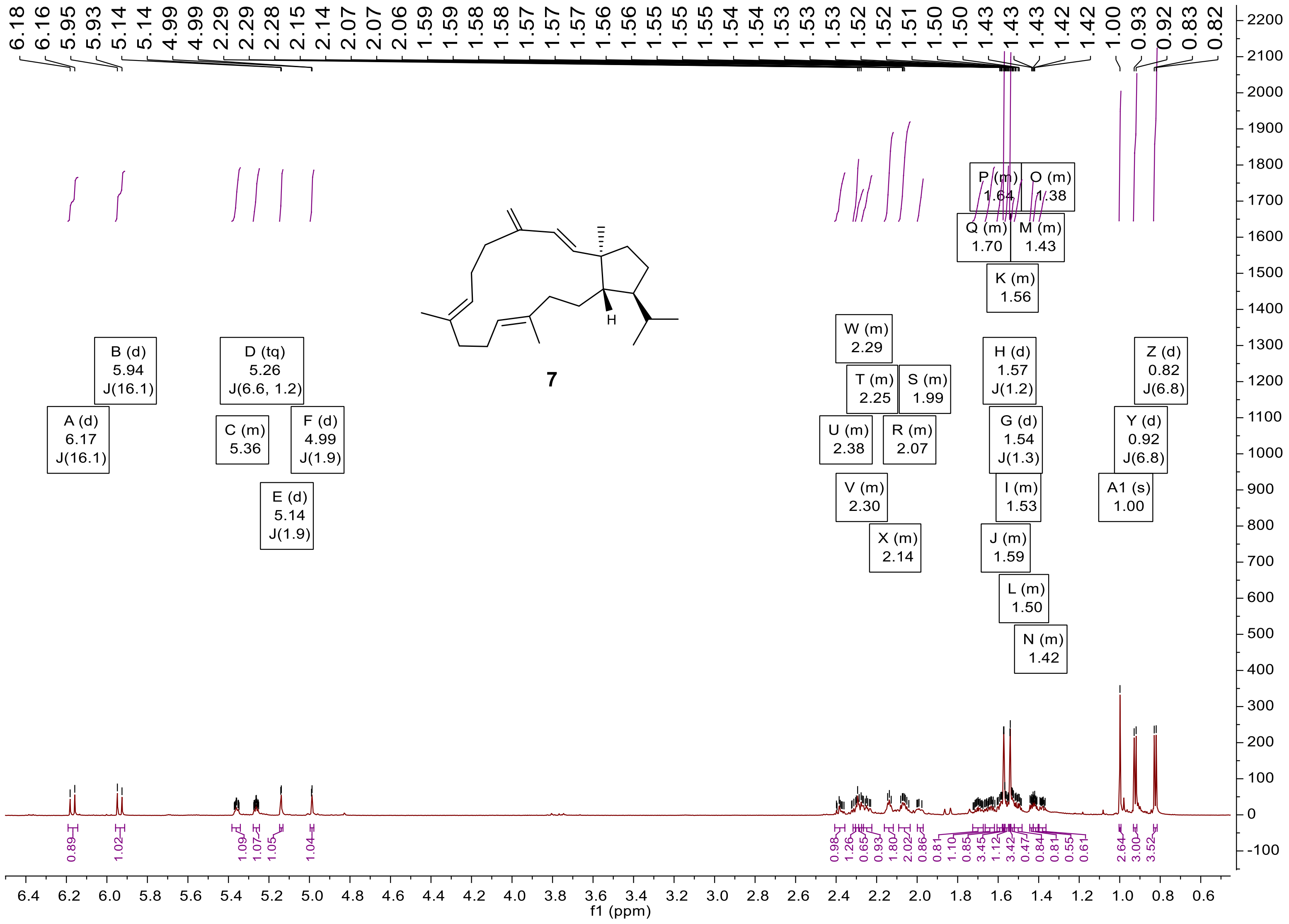

**Figure S63.** $^{1}H$-NMR spectrum of **7** (700 MHz, $C_6D_6$).

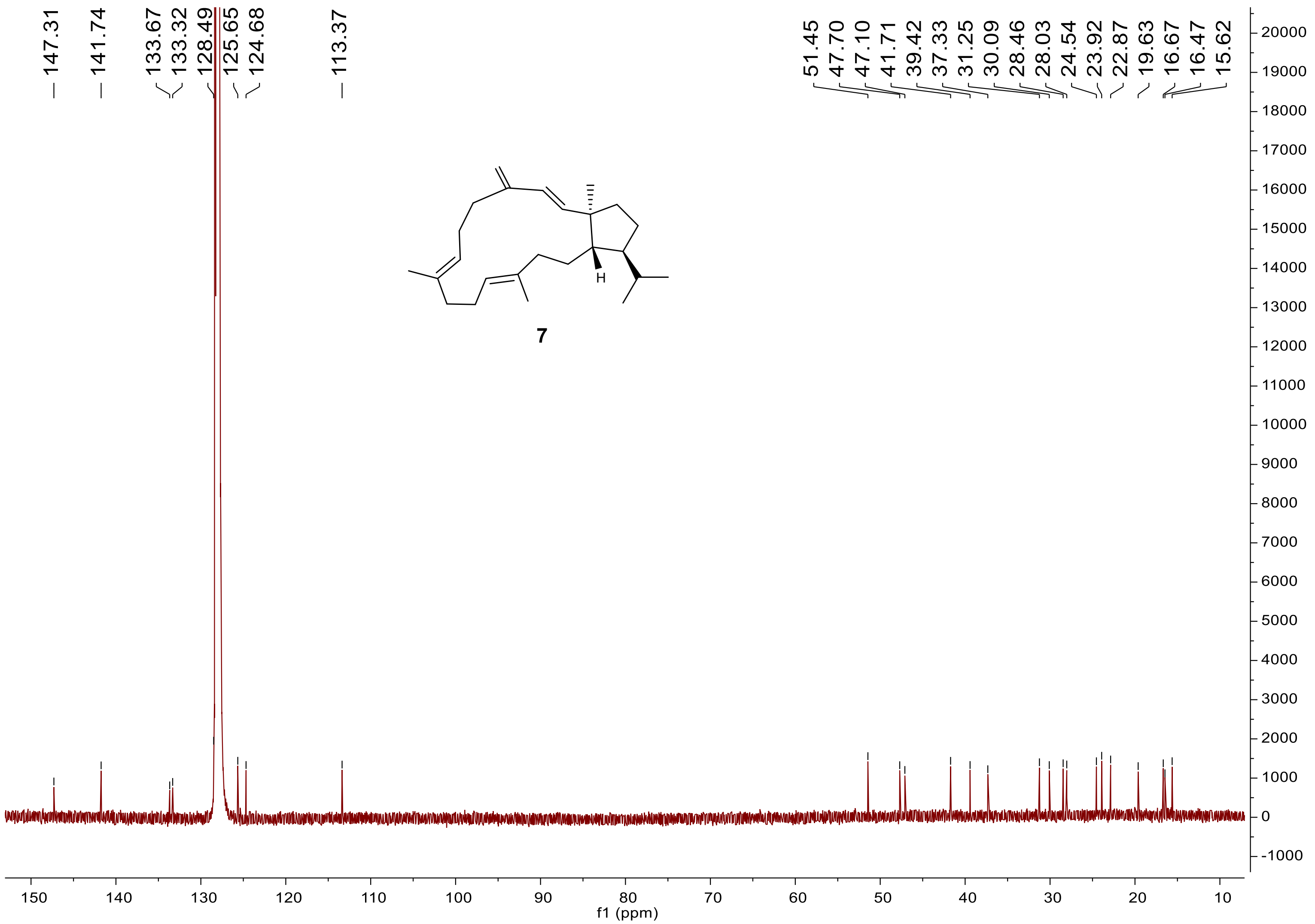


**Figure S64.** $^{13}$C-NMR spectrum of **7** (176 MHz, $C_6D_6$).

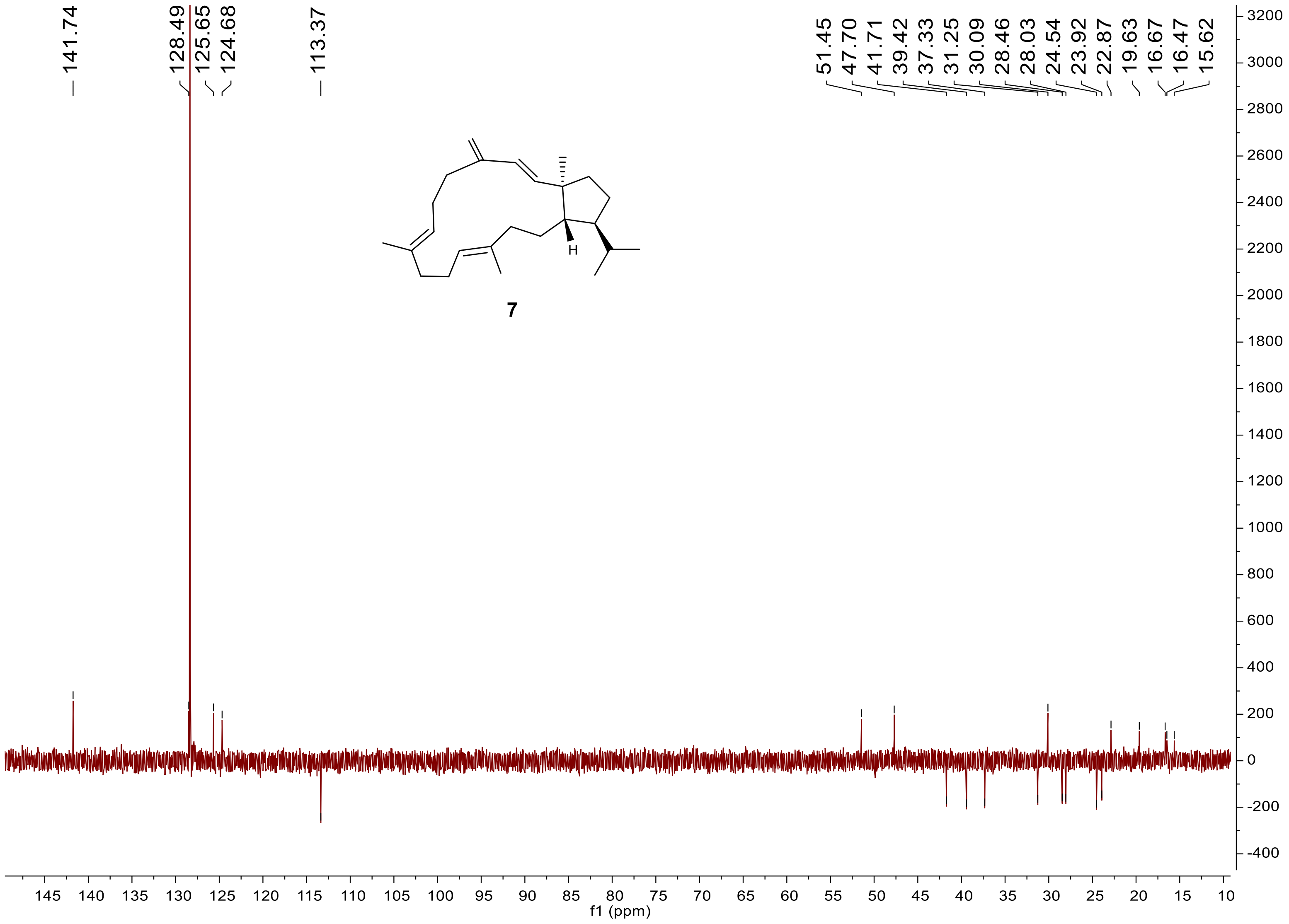


**Figure S65.** $^{13}$C-DEPT135 spectrum of **7** (176 MHz, $C_6D_6$).

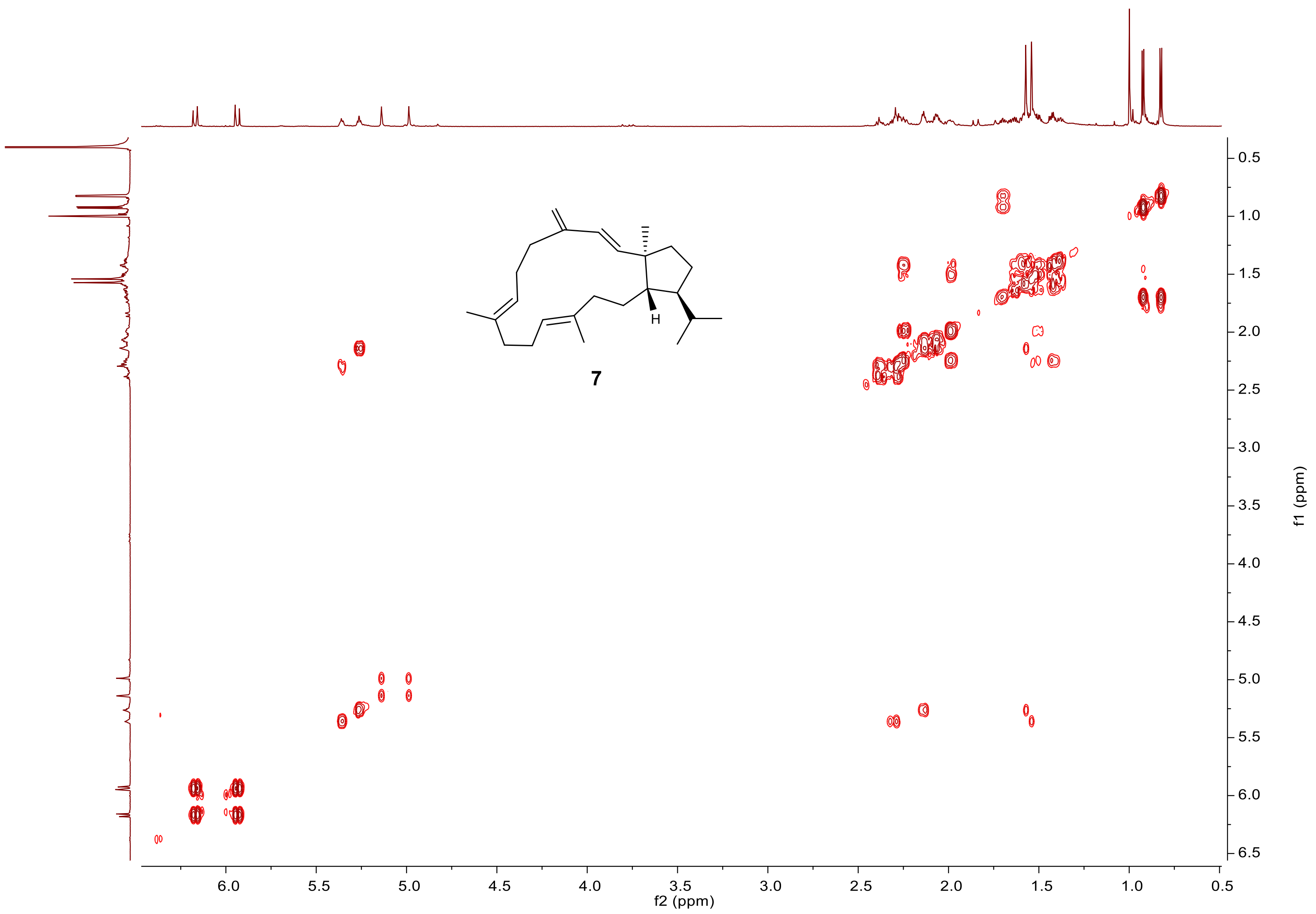


**Figure S66.** $^{1}$H-$^{1}$H-COSY spectrum ($C_6D_6$) of **7**.

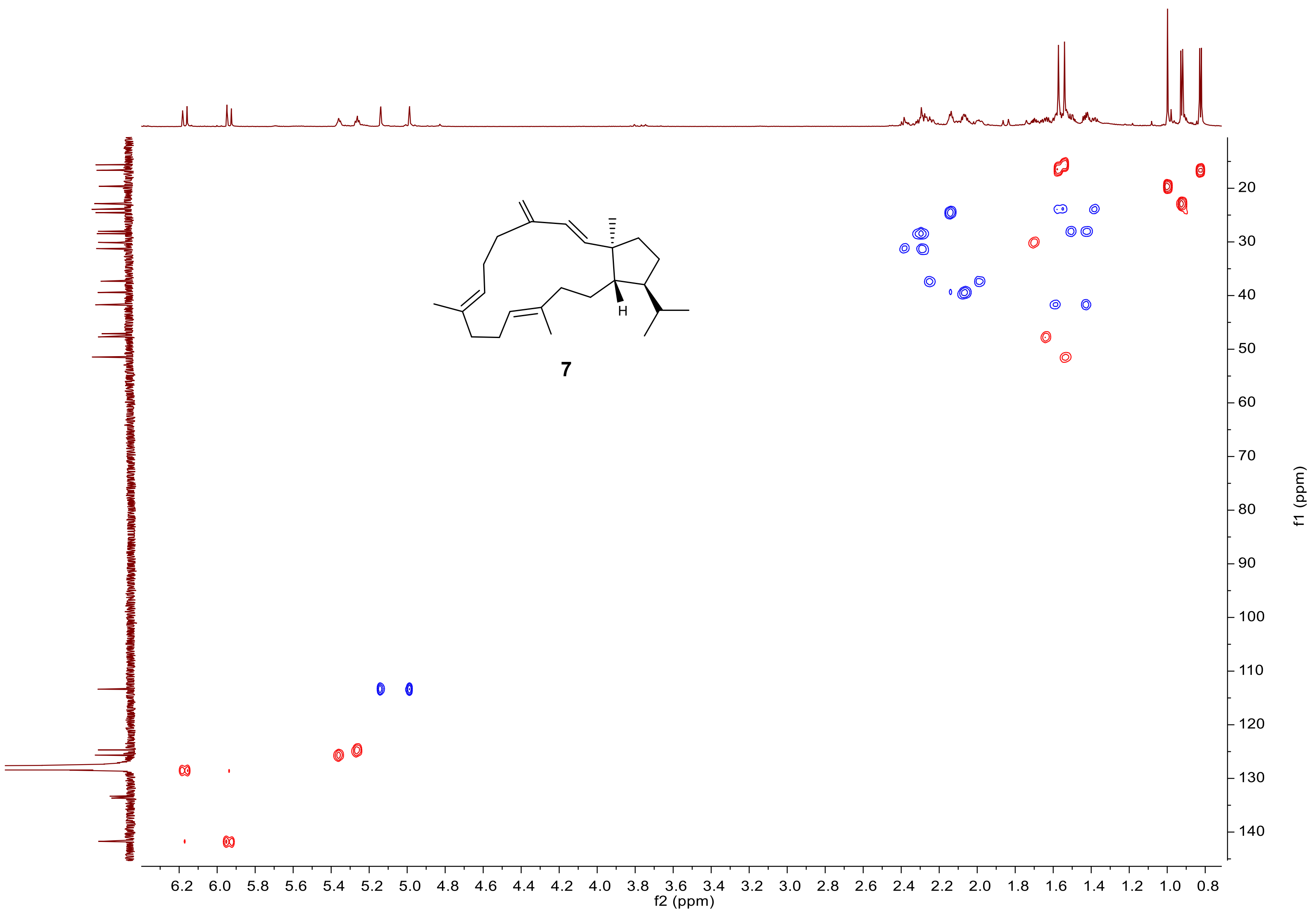


**Figure S67.** HSQC spectrum ($C_6D_6$) of **7**.

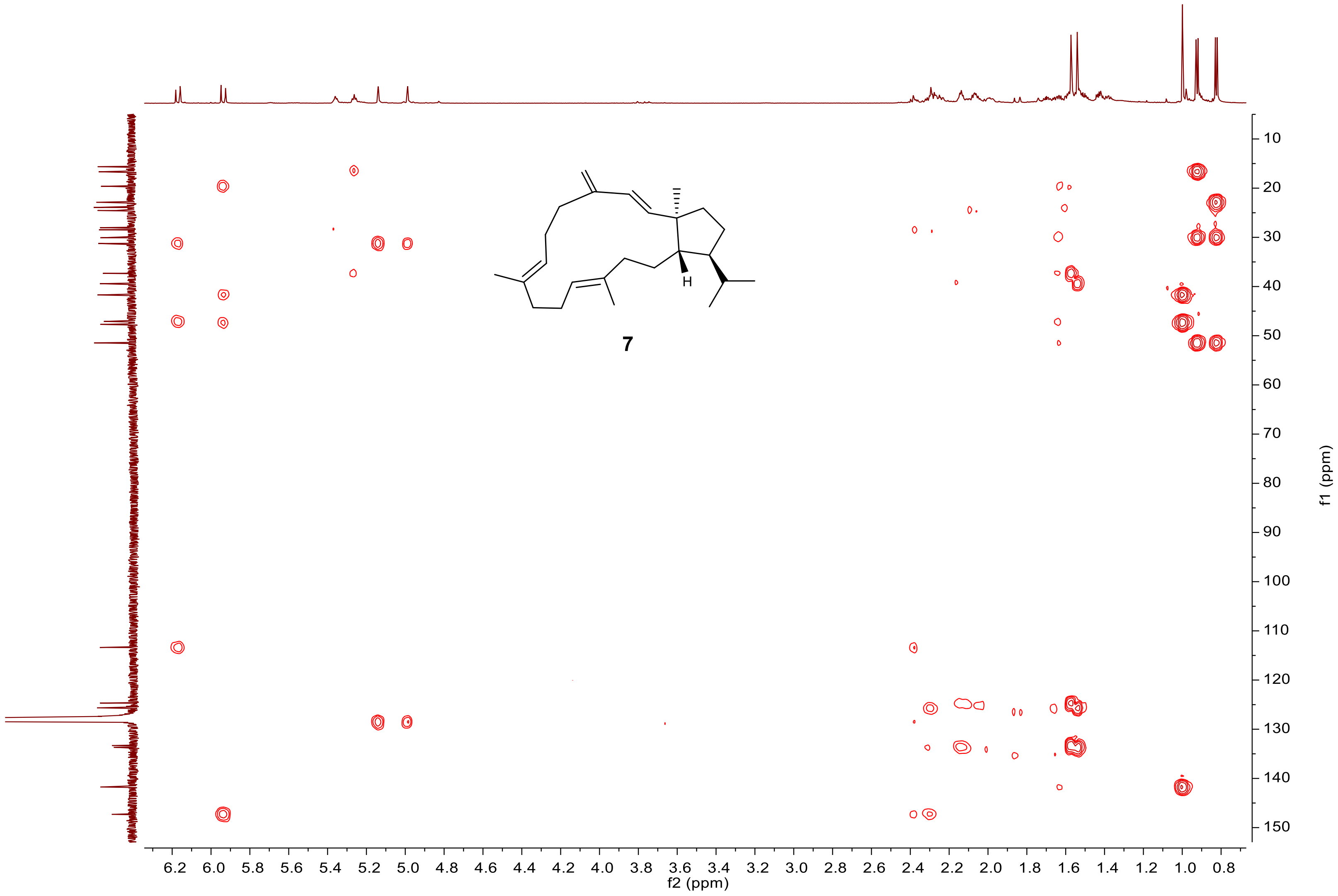


**Figure S68.** HMBC spectrum ($C_6D_6$) of **7**.

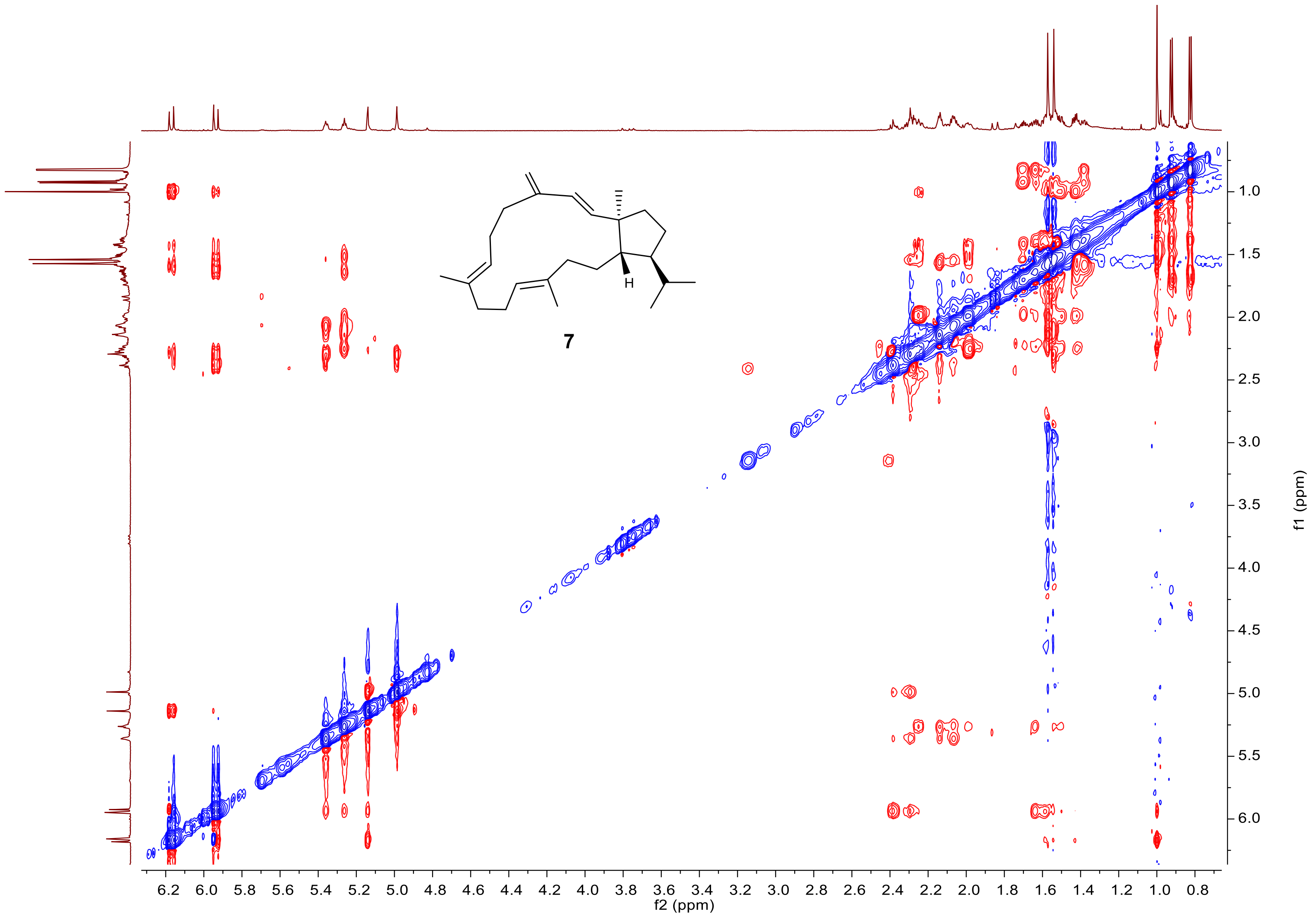


**Figure S69.** NOESY spectrum ($C_6D_6$) of **7**.

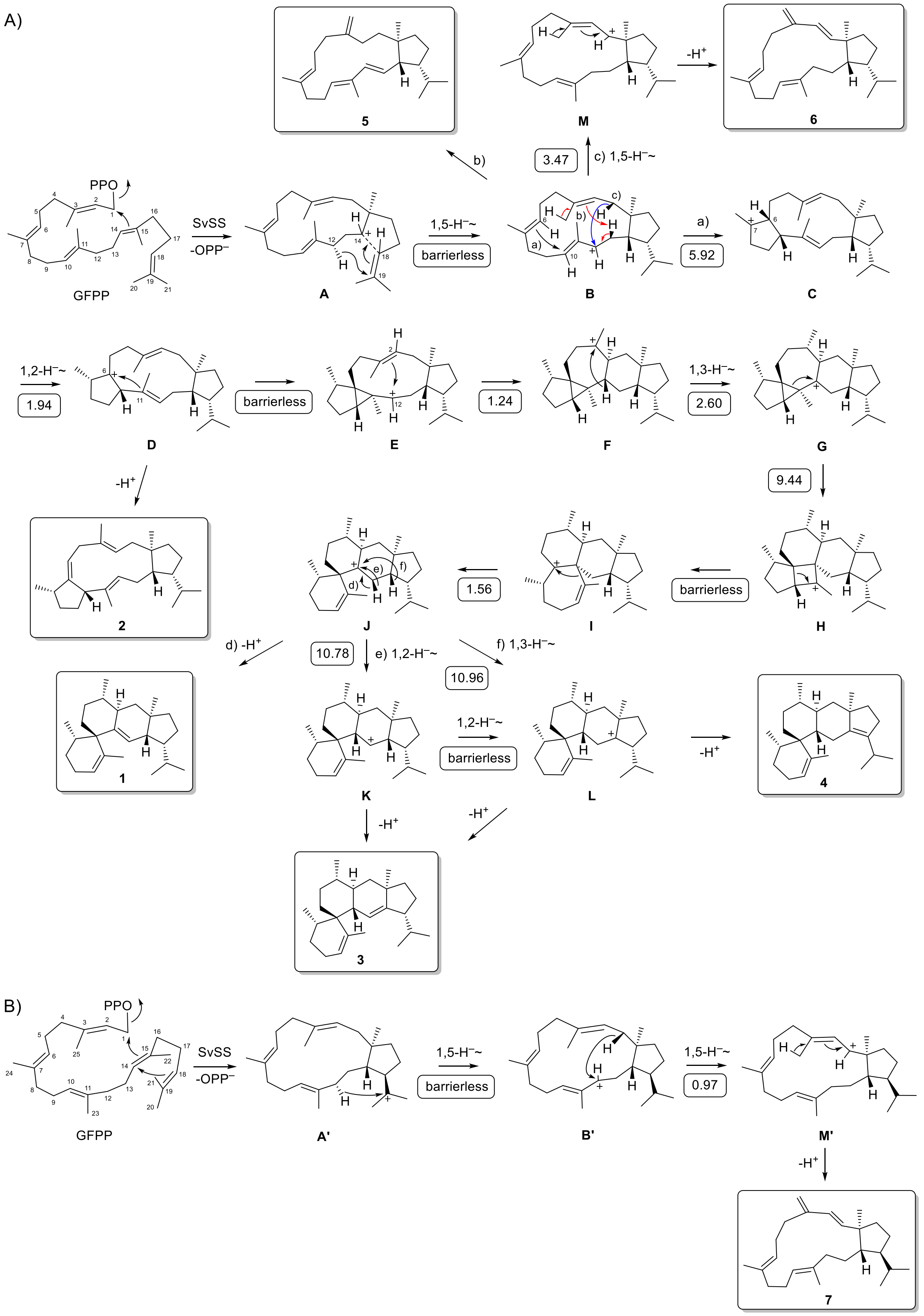


**Scheme S4.** Cyclisation mechanism from GFPP to A) **1 – 6** and B) **7** by SvSS. Numbers in boxes are transition state energies in kcal/mol.

## Computational methods

All computed structures were geometry optimised without restrictions and were characterised as minima or as transition state structures by frequency analyses, also providing Gibbs corrections, using the B97D3/6-31g(d,p) method with the density fitting approximation for s- and p-functions, including Grimme's empirical D3-dispersion correction[47] in Gaussian 16.[48] For improved single point energies, the mPW1PW91 functional was applied with the 6-311+G(d,p) basis set without density fitting and the ultra-fine integration grid, as this method was shown to be very reliable for examining carbocation cyclisation and rearrangement reactions.[1,49-52] The Gibbs corrections include an entropic quasi-harmonic treatment with a frequency cut-off value of 100.0 wavenumbers, according to according to Grimme, using a mixture of RRHO and free-rotor vibrational entropies.[53,54] Computational data are summarised in Table S12 and visualised in Figure S70.

**Table S12.** Results of DFT calculations.

| Structure | Single Point Energy with Gibbs Correction (Hartree) | Relative to A (A') (kcal/mol) |
|---|---|---|
| A | –977.0444998 | 0.00 |
| A-TS | –977.0468954 | –1.50 |
| B | –977.0684684 | –15.04 |
| B-TS1 | –977.0590279 | –9.12 |
| C | –977.0726126 | –17.64 |
| C-TS | –977.0695143 | –15.70 |
| D | –977.0776392 | –20.80 |
| D-TS | –977.0795849 | –22.02 |
| E | –977.0814772 | –23.20 |
| E-TS | –977.0794922 | –21.96 |
| F | –977.0861063 | –26.11 |
| F-TS | –977.0819583 | –23.51 |
| G | –977.1097983 | –40.98 |
| G-TS | –977.0947641 | –31.54 |
| H | –977.1004530 | –35.11 |
| H-TS | –977.1004468 | –35.11 |
| I | –977.1073293 | –39.43 |
| I-TS | –977.1098147 | –40.99 |
| J | –977.1116527 | –42.14 |
| J-TS1 | –977.094480 | –31.36 |
| J-TS2 | –977.094194 | –31.18 |
| K | –977.095188 | –31.80 |
| K-TS | –977.098412 | –33.86 |
| L | –977.117873 | –46.04 |
| B-TS2 | –977.0629340 | –11.57 |
| M | –977.0719920 | –17.25 |
| A' | –977.0591200 | 0.00 |
| A'-TS | –977.0602150 | –0.69 |
| B' | –977.0719230 | –8.03 |
| B'-TS | –977.0706500 | –7.24 |
| M' | –977.0790800 | –12.53 |

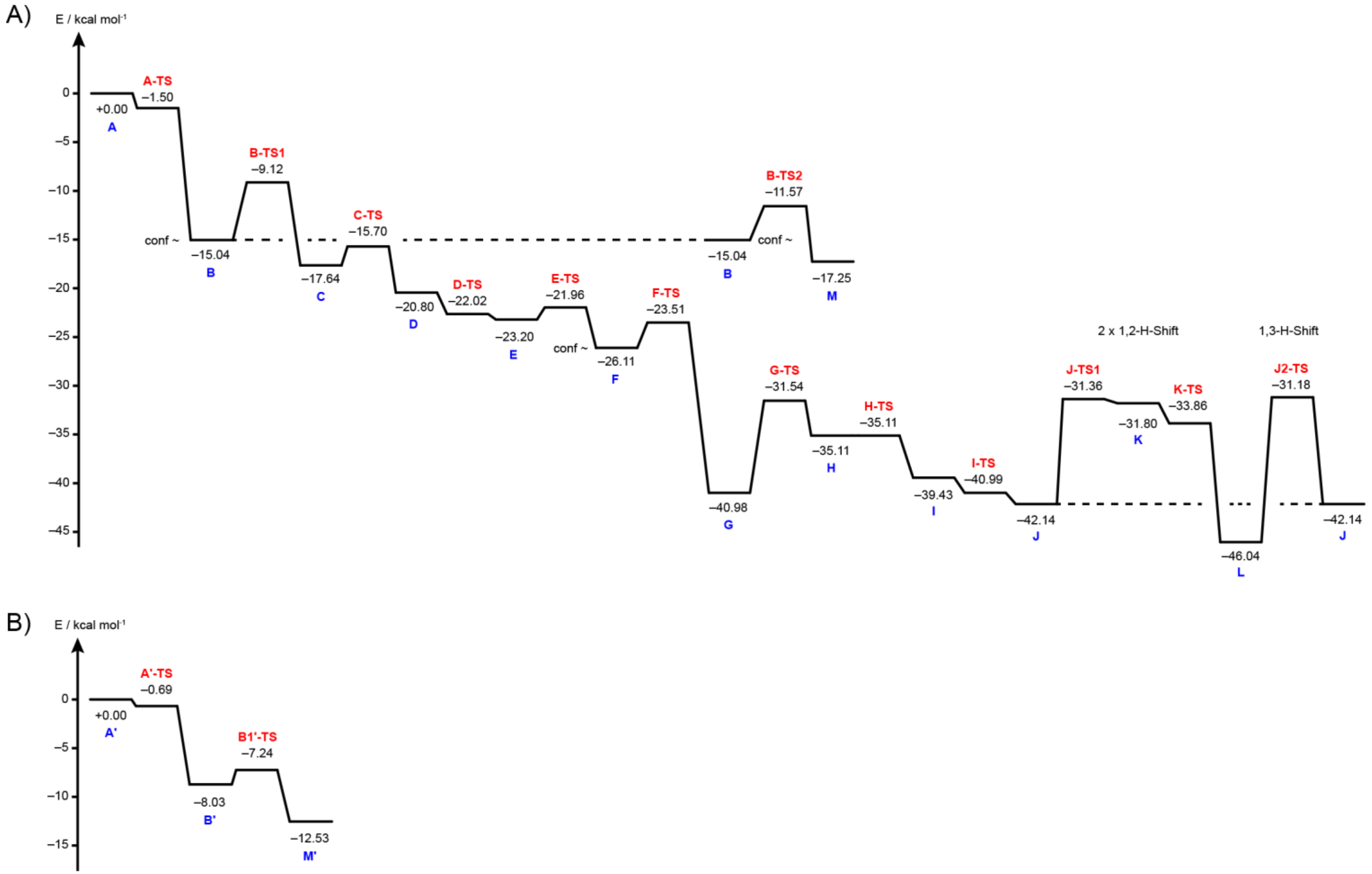


**Figure S70.** Energy profile for the cyclisation mechanism of A) compounds **1** – **6** and B) **7** by SvSS as shown in Scheme 1 of main text based on data from DFT calculations (Table S12). The dashed lines connect identical structures

**Incubation experiment with (2-$^2$H)GPP and (4-$^{13}$C)IPP**

In order to investigate the final hydride shifts in the biosynthesis of **3** and **4**, a large scale isotopic labelling experiment was performed (small scales as for the other labelling experiments failed to give the required NMR signals) with (2-$^2$H)GPP[43] (10 mg, in 12.5 mL 25 mM $NH_4HCO_3$) and (4-$^{13}$C)IPP[13] (15 mg, in 12.5 mL 25 mM $NH_4HCO_3$), incubation buffer (150 mL), preparations of purified enzymes GFPPS and SvSS (each 12.5 mL from 3 L expression cultures) and a solution of β-CD (13.3 mL, 160 mM β-CD, 0.2% SDS, 10% glycerol, 10 mM $MgCl_2$, pH = 8.2). After incubation at 28 °C overnight, the products were extracted with *n*-hexane (3 x 200 mL), the combined extracts were dried with $MgSO_4$ and concentrated in vacuo. The residue was dissolved in $C_6D_6$ (500 µL) and analysed by NMR and GC/MS without further purification.

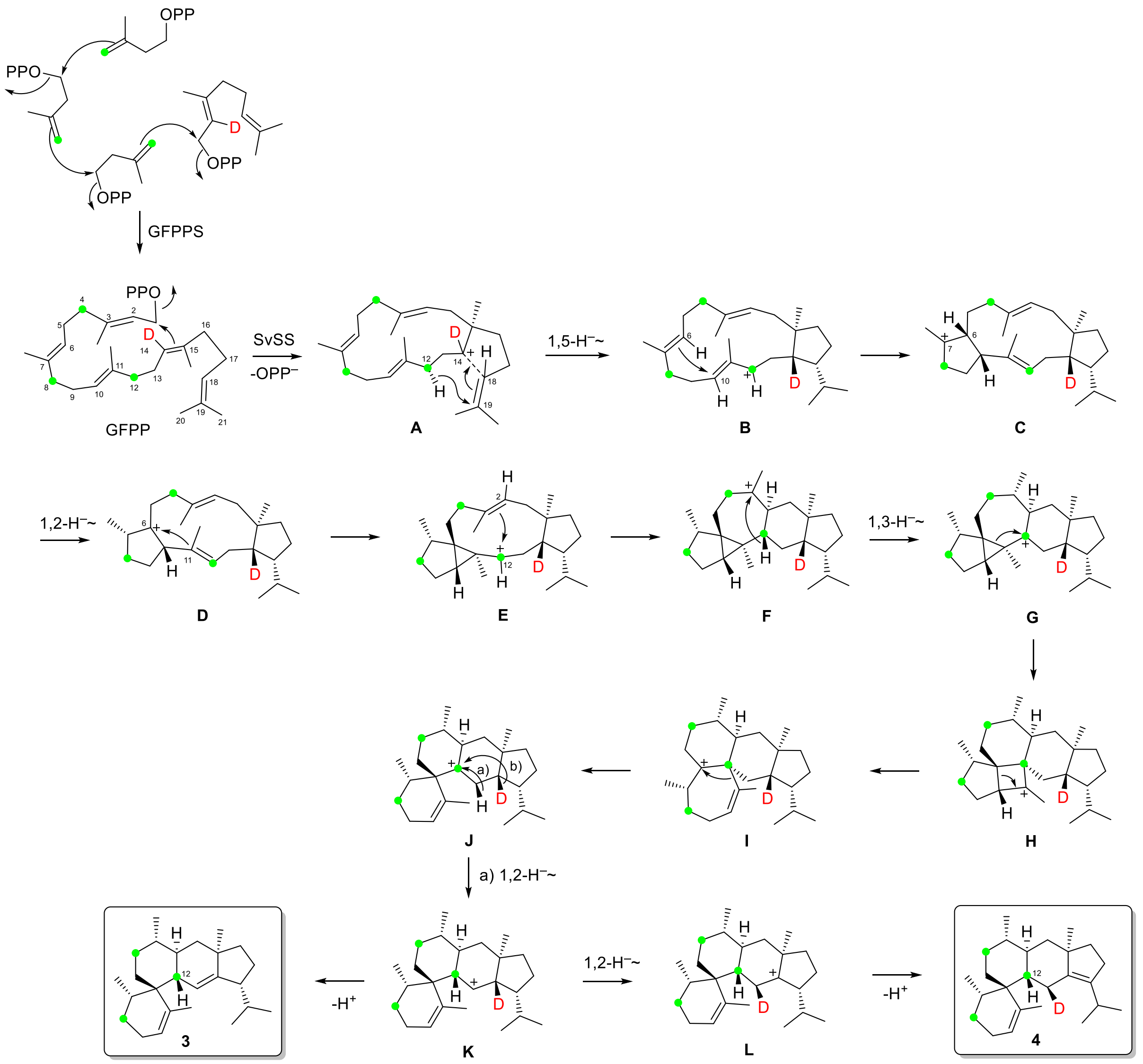


**Scheme S5.** Biosynthesis of labelled **3** and **4** from (2-$^2$H)GPP and (4-$^{13}$C)IPP with GFPPS and SvSS.

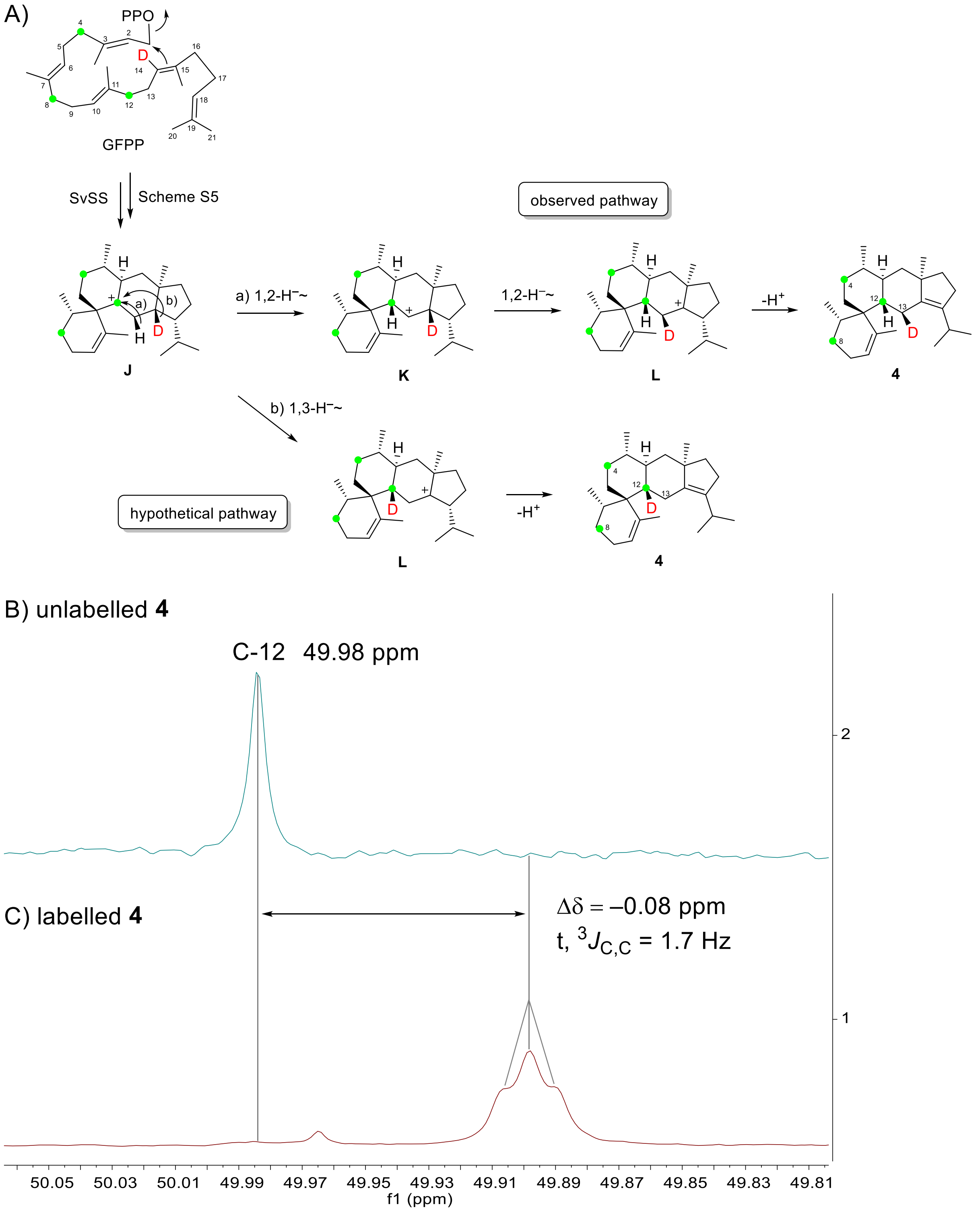


**Figure S71. Final hydride shifts in the biosynthesis of 4.** A) The formation of the precursor of **4**, cation **L**, from intermediate J can either proceed through two sequential 1,2-hydride shifts or a single 1,3-hydride shift. B) $^{13}$C-NMR signal for C12 of unlabelled **4**. C) $^{13}$C-NMR signal for C-12 of labelled **4** obtained from (2-$^{2}$H)GPP and (4-$^{13}$C)IPP with GFPPS and SvSS. The triplet shape of C-12 is due to the $^{3}J_{C,C}$ couplings of C-12 with C-4 and C-8. The deshielding of C-12 (–0.08 ppm) indicates that the vicinal carbon is deuterated which supports the proposed two sequential 1,2-hydride shifts.

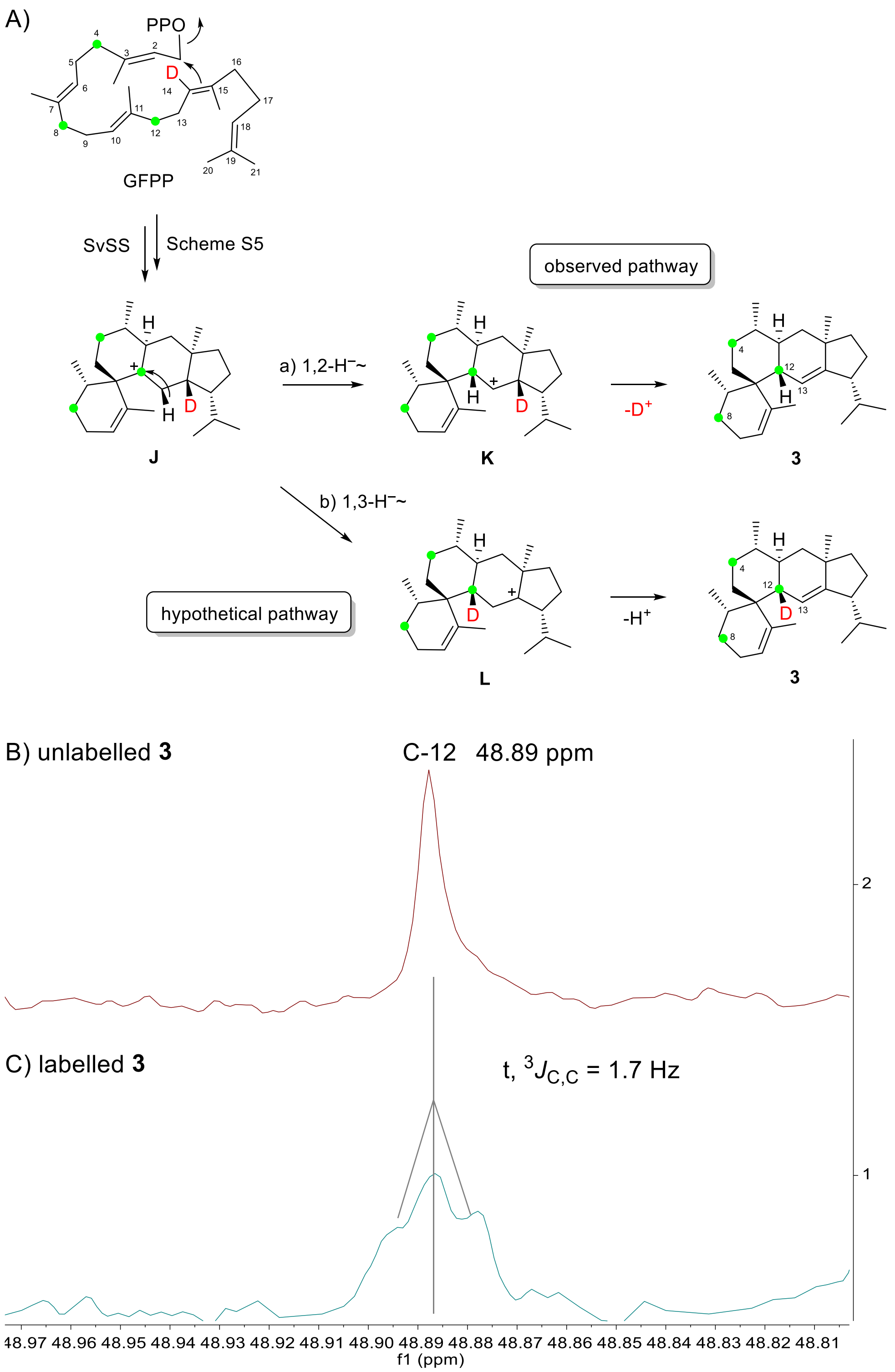


**Figure S72. Final hydride shift in the biosynthesis of 3.** A) The precursor of **3** could be cation **K**, arising through a 1,2-hydride shift from **J**, or cation **L**, formed through a 1,3-hydride shift from **J**. B) $^{13}$C-NMR signal for C-12 of unlabelled **3**. C) $^{13}$C-NMR signal for C-12 of labelled **3** obtained from (2-$^{2}$H)GPP and (4-$^{13}$C)IPP with GFPPS and SvSS. The triplet shape of C12 is due to the $^{3}J_{C,C}$ couplings of C-12 with C-4 and C-8. The absence of a triplet coupling of C-12 with deuterium (a $^{1}J_{C,D}$ coupling would account for a coupling constant of about 20 Hz) supports the proposed 1,2-hydride shift. Moreover, mass spectrometric analysis of **3** indicated the loss of deuterium in its formation.

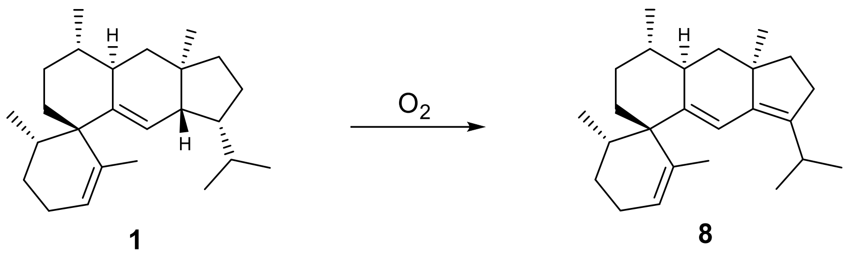

**Scheme S6.** Oxidation of **1** by air.

**Oxidation of 1 by air**
Compound **1** (4.0 mg, 11.7 µmol, 1.0 eq.) was dissolved in a 25 mL round-bottomed flask in $CH_2Cl_2$ (1.0 mL). After 24 hours of exposure to air, the dried sample was used directly for purification. Purification by column chromatography on silica gel [100% *n*-hexane] yielded 14,18-dehydrosesterviolene A (**8**, 2.2 mg, 6.5 µmol, 56%) as a colourless oil.

**14,18-Dehydrosesterviolene A (8).** TLC (100% *n*-hexane): $R_f$ = 0.6. GC (HP5-MS): *I* = 2317. MS (EI, 70 eV): *m*/*z* (%) = 338 (100), 323 (38), 310 (1), 295 (51), 281 (35), 267 (6), 253 (57), 239 (7), 225 (8), 213 (16), 197 (14), 185 (16), 173 (13), 159 (19), 143 (10), 131 (11), 119 (8), 105 (14), 91 (13), 79 (8), 67 (6), 55 (11), 43 (11). IR (diamond ATR): ṽ = 2949 (s), 2924 (s), 2857 (m), 1666 (w), 1456 (m), 1375 (w), 1345 (w), 1326 (w), 1303 (w), 1257 (w), 1196 (w), 1109 (w), 1094 (w), 1017 (w), 961 (w), 891 (w), 811 (w), 734 (w), 706 (w), 665 (w), 580 (w), 543 (w), 517 (w), 466 (w), 438 (w) $cm^{-1}$. HR-MS (Q-TOF, 70 eV): calc. $[C_{25}H_{38}]^{+\cdot}$ *m*/*z* = 338.2968; found: *m*/*z* = 338.2965. Optical rotary power: $[\alpha]_D^{20}$ = +128.6 (*c* 0.22, $CH_2Cl_2$). NMR data are given in Table S13.

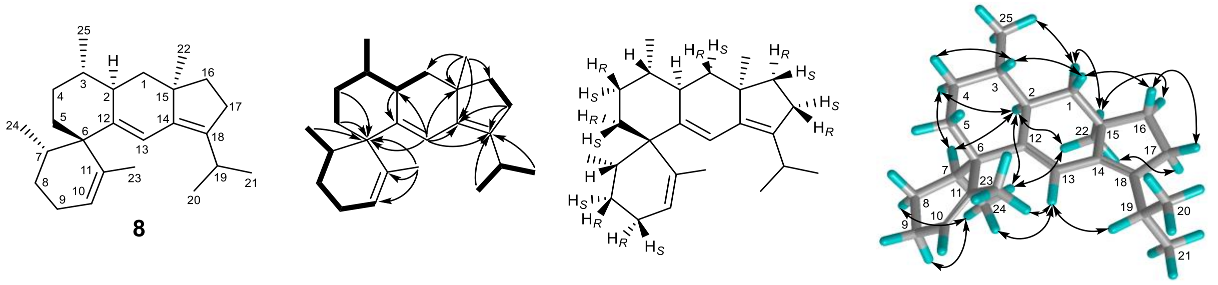

**Figure S73.** Structure elucidation of **8**. Bold: $^{1}$H,$^{1}$H-COSY, single headed arrows: key HMBC, and double headed arrows: NOESY correlations. Carbon numbering follows GFPP numbering to indicate the origin of each carbon. Diastereotopic hydrogens are labelled $H_R$ (*pro-R*) and $H_S$ (*pro-S*).

**Table S13.** NMR data of 14,18-dehydrosesterviolene A (**8**) in $C_6D_6$ recorded at 298 K.

| C[a] | type | $^{1}$H[b] | $^{13}$C[b] |
|---|---|---|---|
| 1 | $CH_2$ | 2.09 (dd, *J* = 12.4, 5.1, $H_S$)<br>1.18 (t, *J* = 11.7, $H_R$) | 43.84 |
| 2 | CH | 1.80 (m) | 40.92 |
| 3 | CH | 1.09 (m) | 38.84 |
| 4 | $CH_2$ | 1.40 (dq, *J* = 13.4, 3.7, $H_R$)<br>1.34 (m, $H_S$) | 31.46 |
| 5 | $CH_2$ | 1.72 (m, 2H) | 34.57 |
| 6 | $C_q$ | – | 49.31 |
| 7 | CH | 2.07 (m) | 31.24 |
| 8 | $CH_2$ | 1.78 (dd, *J* = 11.8, 7.0, $H_R$)<br>1.35 (dd, *J* = 11.7, 5.7, $H_S$) | 25.93 |
| 9 | $CH_2$ | 2.11 (m, $H_R$)<br>1.78 (m, $H_S$) | 21.43 |
| 10 | CH | 5.62 (m) | 125.64 |
| 11 | $C_q$ | – | 138.57 |
| 12 | $C_q$ | – | 140.06 |
| 13 | CH | 6.16 (d, *J* = 2.4) | 119.88 |
| 14 | $C_q$ | – | 137.66 |
| 15 | $C_q$ | – | 44.82 |
| 16 | $CH_2$ | 1.78 (m, $H_R$)<br>1.56 (td, *J* = 11.1, 8.8, $H_S$) | 40.24 |
| 17 | $CH_2$ | 2.48 (m, $H_S$)<br>2.26 (dd, *J* = 16.3, 8.8, $H_R$) | 28.92 |
| 18 | $C_q$ | – | 139.76 |
| 19 | CH | 2.91 (m) | 27.08 |
| 20 | $CH_3$ | 0.96 (d, *J* = 6.9) | 21.42 |
| 21 | $CH_3$ | 1.06 (d, *J* = 7.0) | 22.22 |
| 22 | $CH_3$ | 1.06 (s) | 23.47 |
| 23 | $CH_3$ | 1.86 (m) | 20.79 |
| 24 | $CH_3$ | 0.89 (d, *J* = 7.1) | 17.29 |
| 25 | $CH_3$ | 0.92 (d, *J* = 6.4) | 20.90 |

[a] Carbon numbering as shown in Figure S73. [b] Chemical shifts δ in ppm, multiplicity: s = singlet, d = doublet, t = triplet, q = quartet, m = multiplet, coupling constants *J* are given in Hertz.

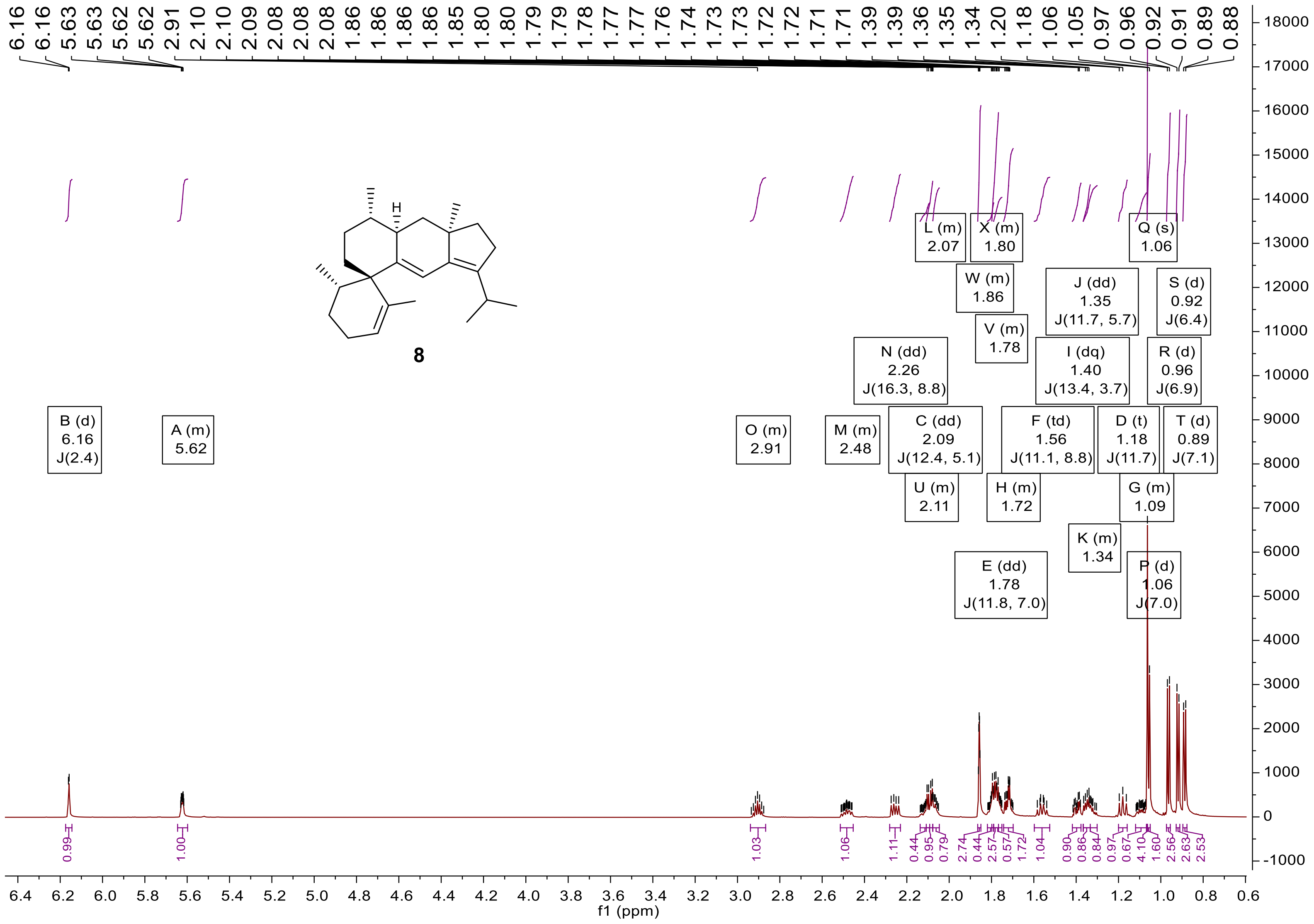

**Figure S74.** $^1$H-NMR spectrum of **8** (700 MHz, $C_6D_6$).

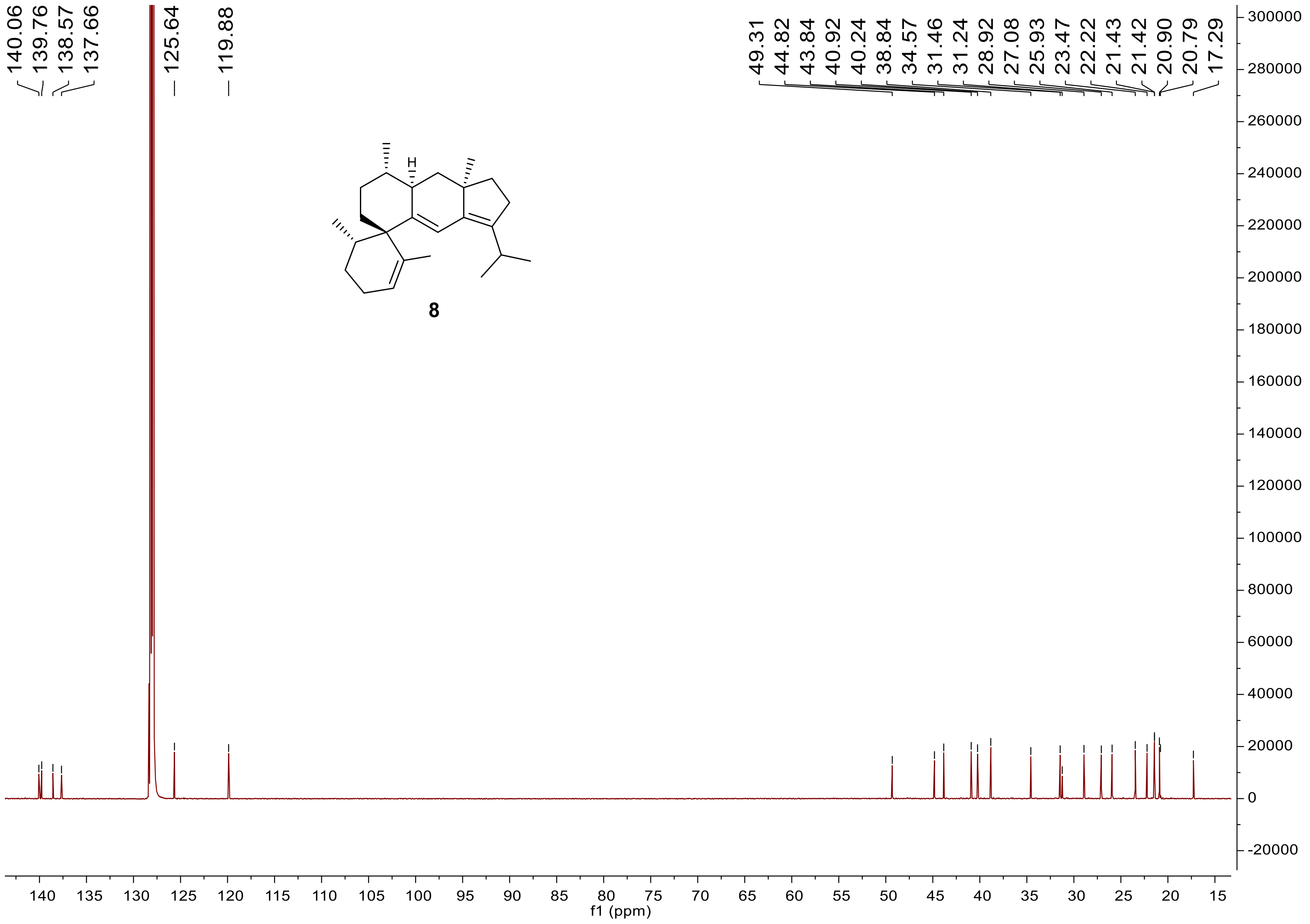


**Figure S75.** $^{13}$C-NMR spectrum of **8** (176 MHz, $C_6D_6$).

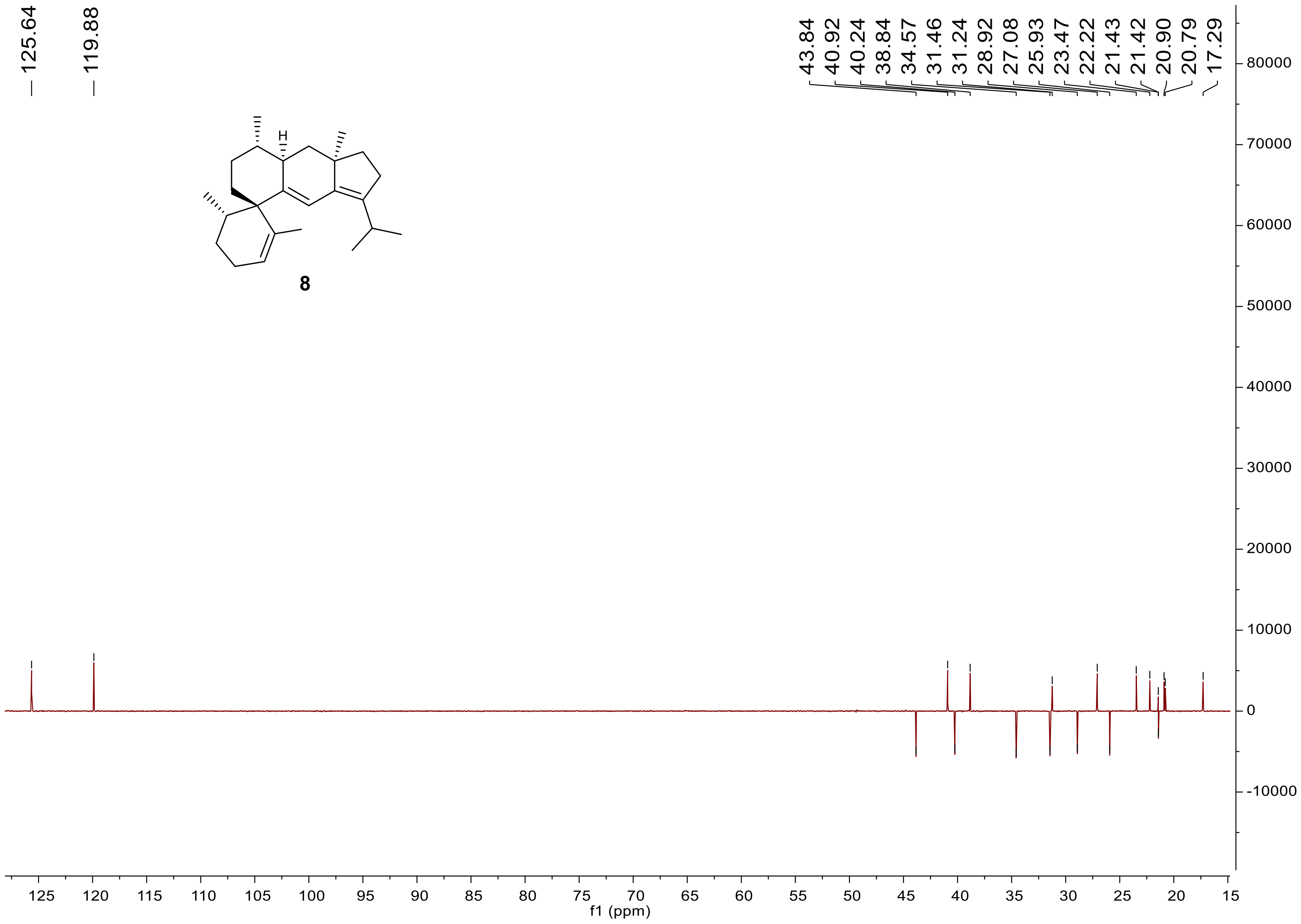


**Figure S76.** $^{13}$C-DEPT135 spectrum of **8** (176 MHz, $C_6D_6$).

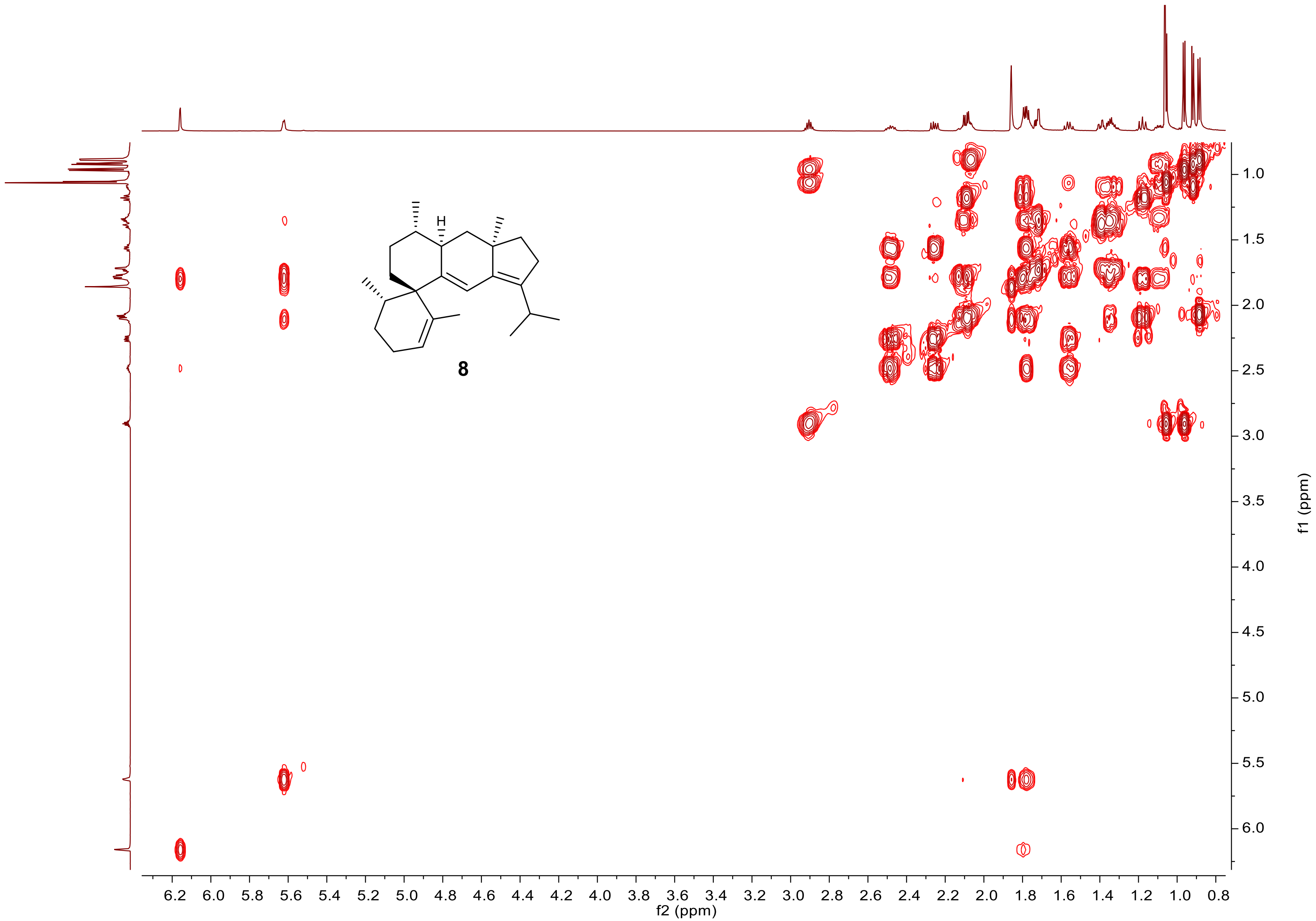


**Figure S77.** $^{1}H$-$^{1}H$-COSY spectrum ($C_6D_6$) of **8**.

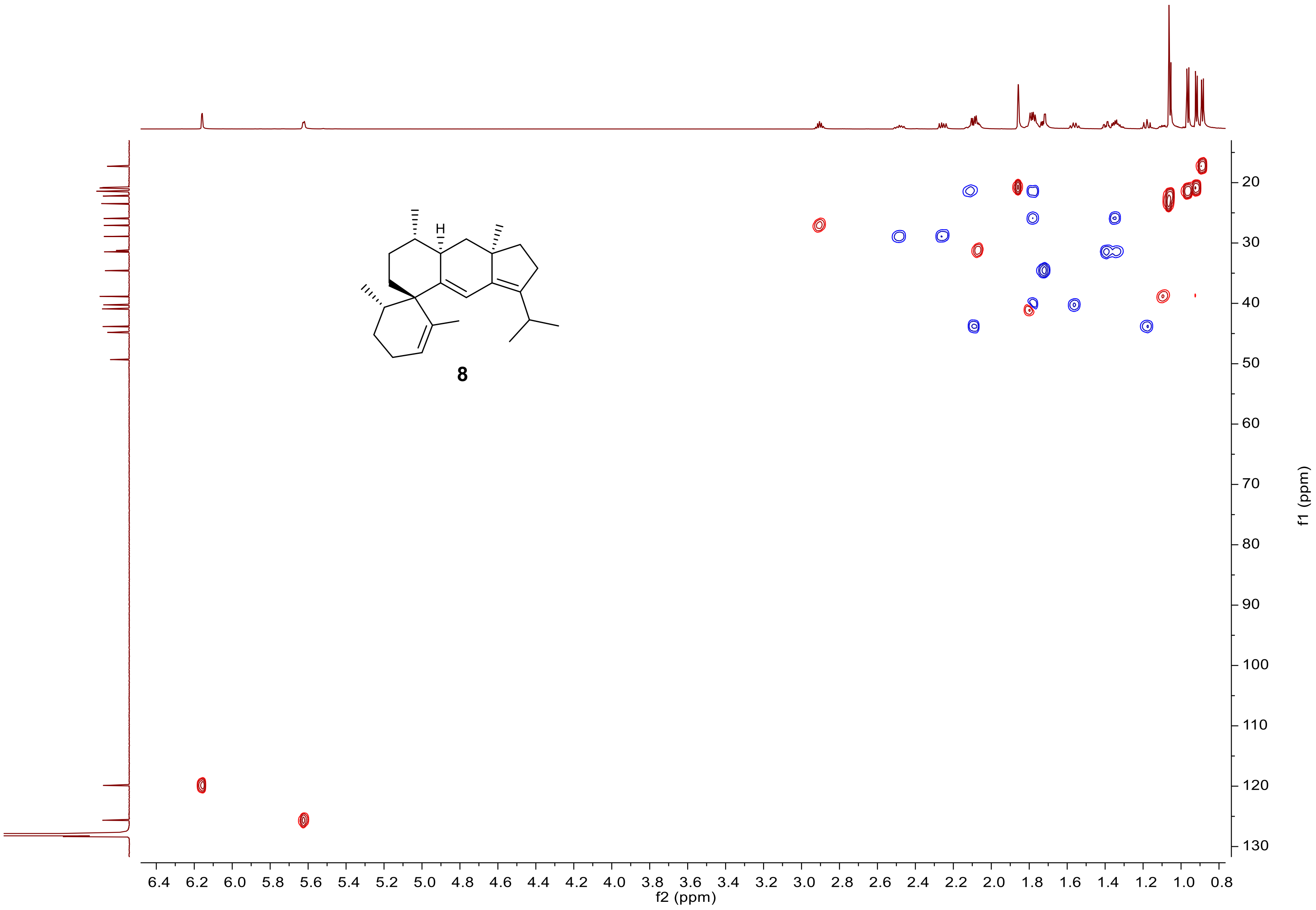


**Figure S78.** HSQC spectrum ($C_6D_6$) of **8**.

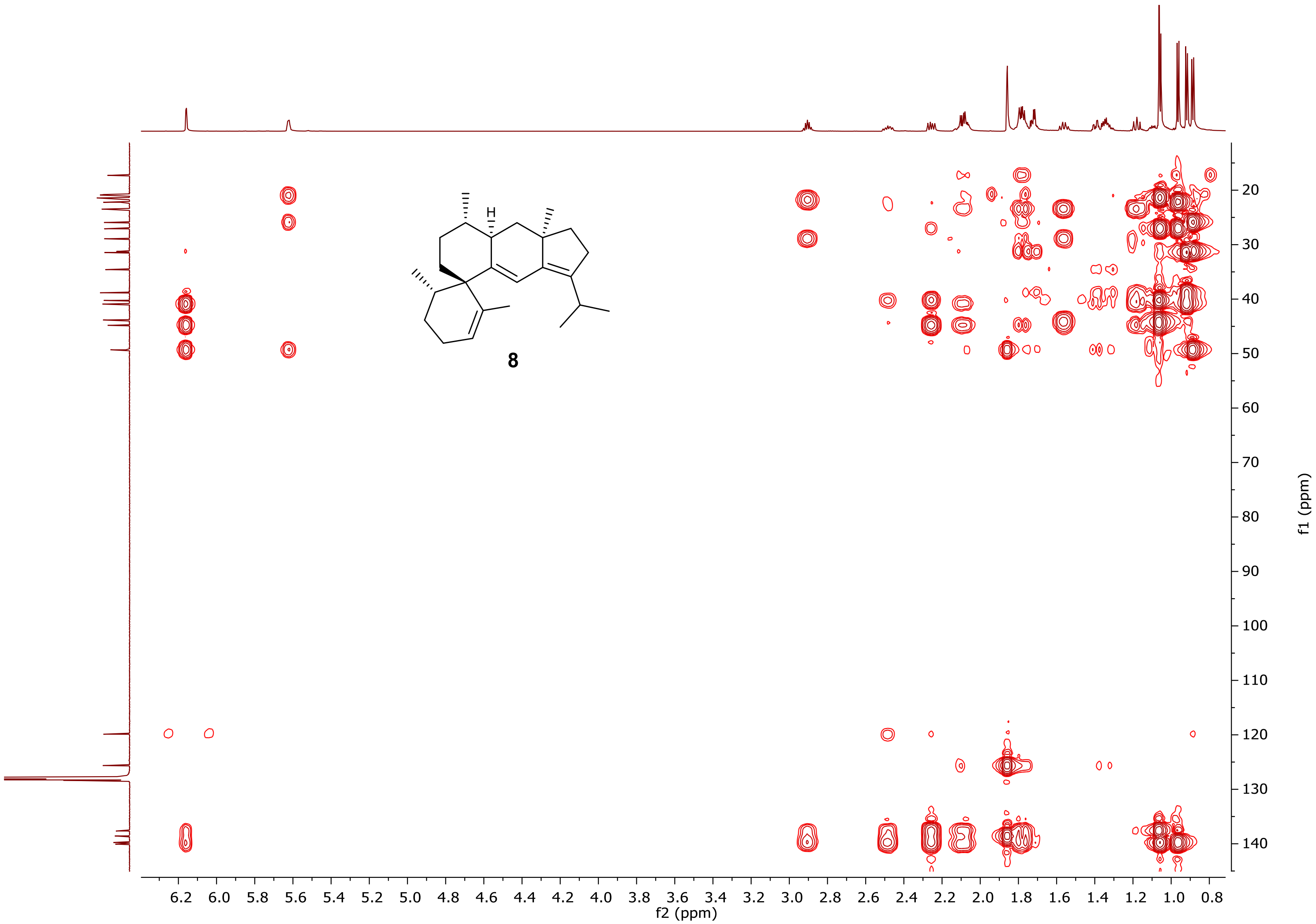


**Figure S79.** HMBC spectrum ($C_6D_6$) of **8**.

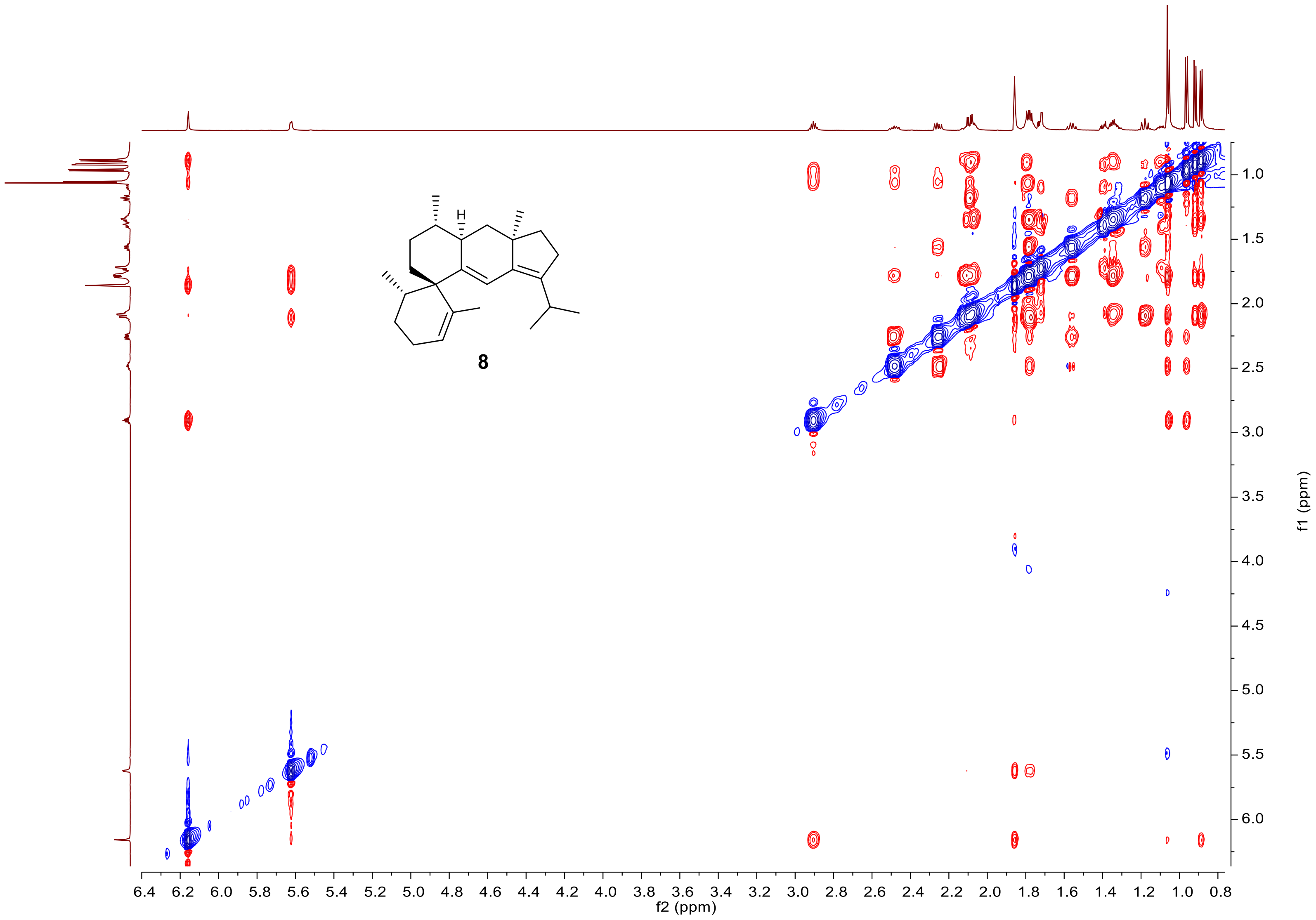


**Figure S80.** NOESY spectrum ($C_6D_6$) of **8**.

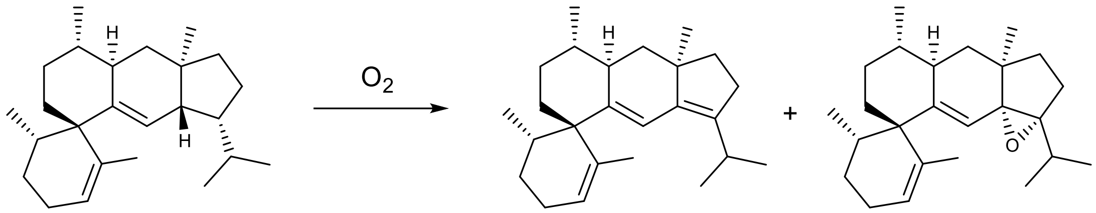


1 8 9

**Scheme S7.** Oxidation of **1** by air.

**Oxidation of 1 by air**

Compound **1** (4.0 mg, 11.7 µmol, 1.0 eq.) was dissolved in a 25 mL round-bottomed flask in $CH_2Cl_2$ (1.0 mL). After 72 hours of exposure to air, the dried sample was used directly for purification. Purification by column chromatography on silica gel [petroether/ethyl acetate (10:1)] yielded 14,18-epoxysesterviolene A (**9**, 0.6 mg, 1.7 µmol, 14%) as a colourless oil.

**Sesterviolene epoxide A (9).** TLC [petroether/ethyl acetate (5:1)]: $R_f$ = 0.8. IR (diamond ATR): $\tilde{v}$ = 2957 (s), 2925 (s), 2856 (m), 1730 (w), 1673 (w), 1455 (m), 1376 (w), 1260 (m), 1194 (w), 1093 (m), 1081 (m), 1022 (s), 969 (w), 892 (w), 853 (w), 801 (s), 687 (w), 587 (w), 507 (w), 472 (w) $cm^{-1}$. HR-MS (Q-TOF, 70 eV): calc. $[C_{25}H_{39}O]^+$ *m*/*z* = 355.2995; found: *m*/*z* =. 355.2992. Optical rotary power: $[\alpha]_D^{20}$ = +51.7 (*c* 0.06, $CH_2Cl_2$). NMR data are given in Table S14.

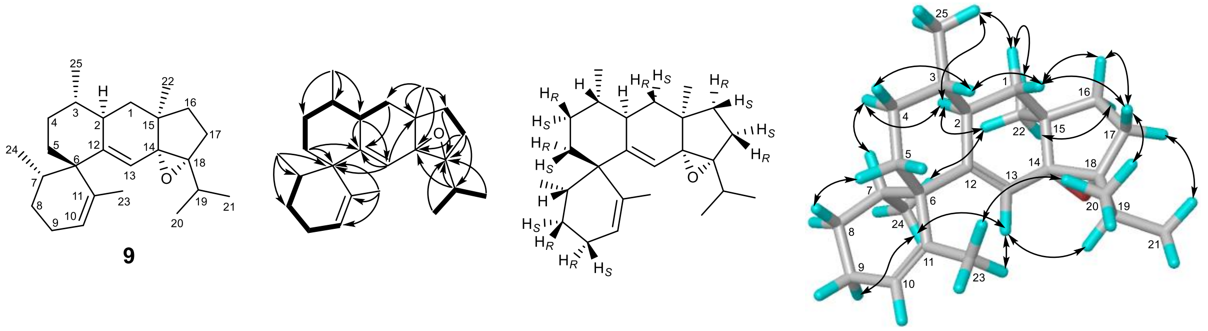

**Figure S81.** Structure elucidation of **9**. Bold: $^1H,^1H$-COSY, single headed arrows: key HMBC, and double headed arrows: NOESY correlations. Carbon numbering follows GFPP numbering to indicate the origin of each carbon. Diastereotopic hydrogens are labelled $H_R$ (*pro-R*) and $H_S$ (*pro-S*).

**Table S14.** NMR data of sesterviolene epoxide A (**9**) in $C_6D_6$ recorded at 298 K.

| C[a] | type | $^1H$[b] | $^{13}C$[b] |
|---|---|---|---|
| 1 | $CH_2$ | 1.68 (m, $H_S$)<br>1.15 (m, $H_R$) | 40.62 |
| 2 | CH | 1.70 (m) | 40.34 |
| 3 | CH | 0.96 (m) | 39.10 |
| 4 | $CH_2$ | 1.35 (m, $H_R$)<br>1.26 (m, $H_S$) | 31.42 |
| 5 | $CH_2$ | 1.62 (m, 2H) | 34.31 |
| 6 | $C_q$ | – | 49.14 |
| 7 | CH | 1.96 (m) | 30.59 |
| 8 | $CH_2$ | 1.70 (m, $H_R$)<br>1.32 (m, $H_S$) | 25.88 |
| 9 | $CH_2$ | 2.04 (m, $H_R$)<br>1.73 (m, $H_S$) | 21.37 |
| 10 | CH | 5.49 (m) | 126.05 |
| 11 | $C_q$ | – | 137.53 |
| 12 | $C_q$ | – | 144.85 |
| 13 | CH | 5.26 (d, $J$ = 2.0) | 122.61 |
| 14 | $C_q$ | – | 74.17 |
| 15 | $C_q$ | – | 40.16 |
| 16 | $CH_2$ | 1.49 (m, $H_R$)<br>1.24 (m, $H_S$) | 35.04 |
| 17 | $CH_2$ | 1.77 (m, $H_S$)<br>1.64 (m, $H_R$) | 22.85 |
| 18 | $C_q$ | – | 77.07 |
| 19 | CH | 1.80 (m) | 31.00 |
| 20 | $CH_3$ | 0.93 (d, $J$ = 7.0) | 18.99 |
| 21 | $CH_3$ | 1.11 (d, $J$ = 6.8) | 18.93 |
| 22 | $CH_3$ | 1.19 (s) | 21.05 |
| 23 | $CH_3$ | 1.66 (m) | 20.42 |
| 24 | $CH_3$ | 0.91 (d, $J$ = 7.0) | 17.13 |
| 25 | $CH_3$ | 0.85 (d, $J$ = 6.4) | 20.28 |

[a] Carbon numbering as shown in Figure S81. [b] Chemical shifts δ in ppm, multiplicity: s = singlet, d = doublet, m = multiplet, coupling constants *J* are given in Hertz.

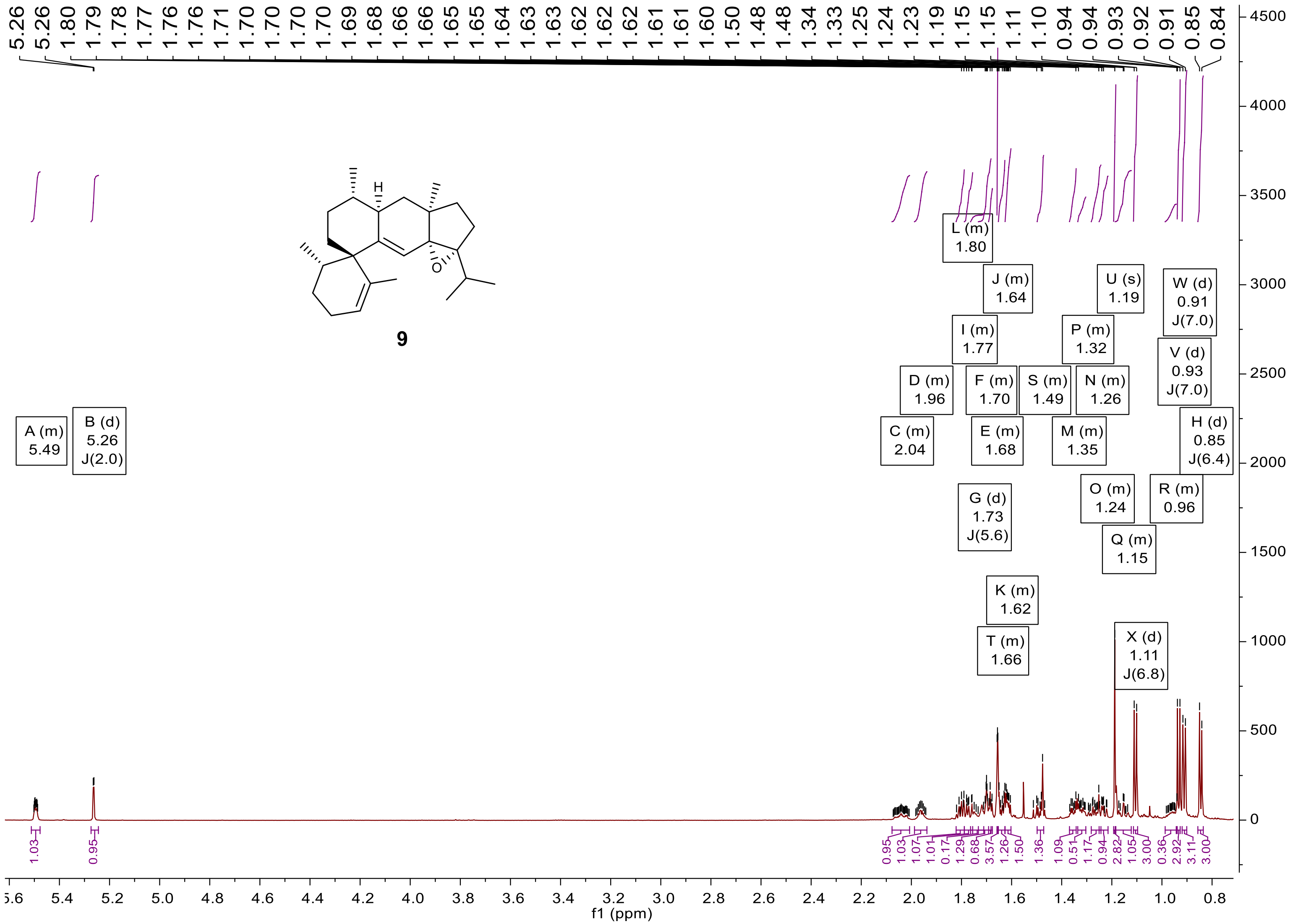

**Figure S82.** $^{1}$H-NMR spectrum of **9** (700 MHz, $C_6D_6$).

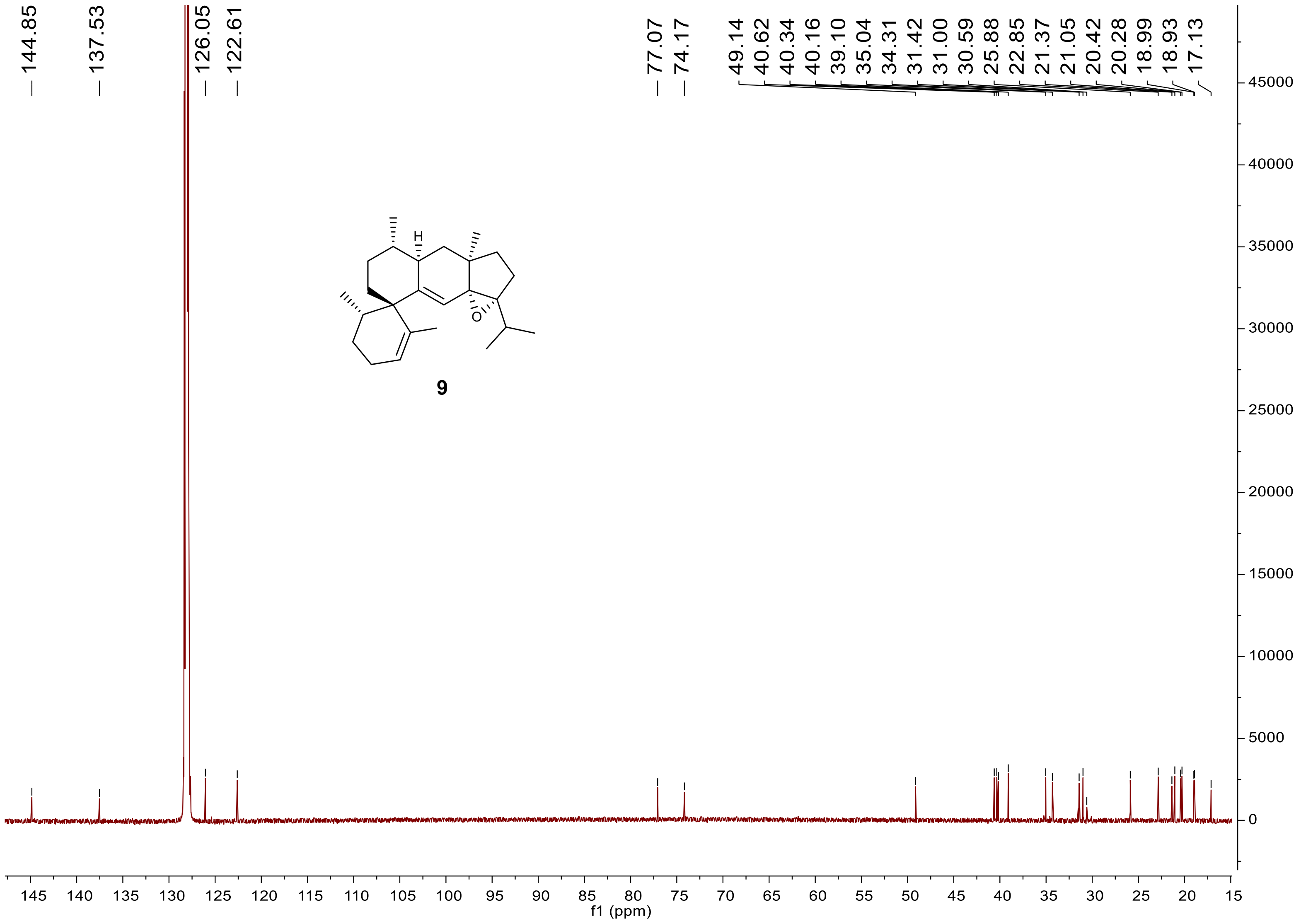


**Figure S83.** $^{13}$C-NMR spectrum of **9** (176 MHz, $C_6D_6$).

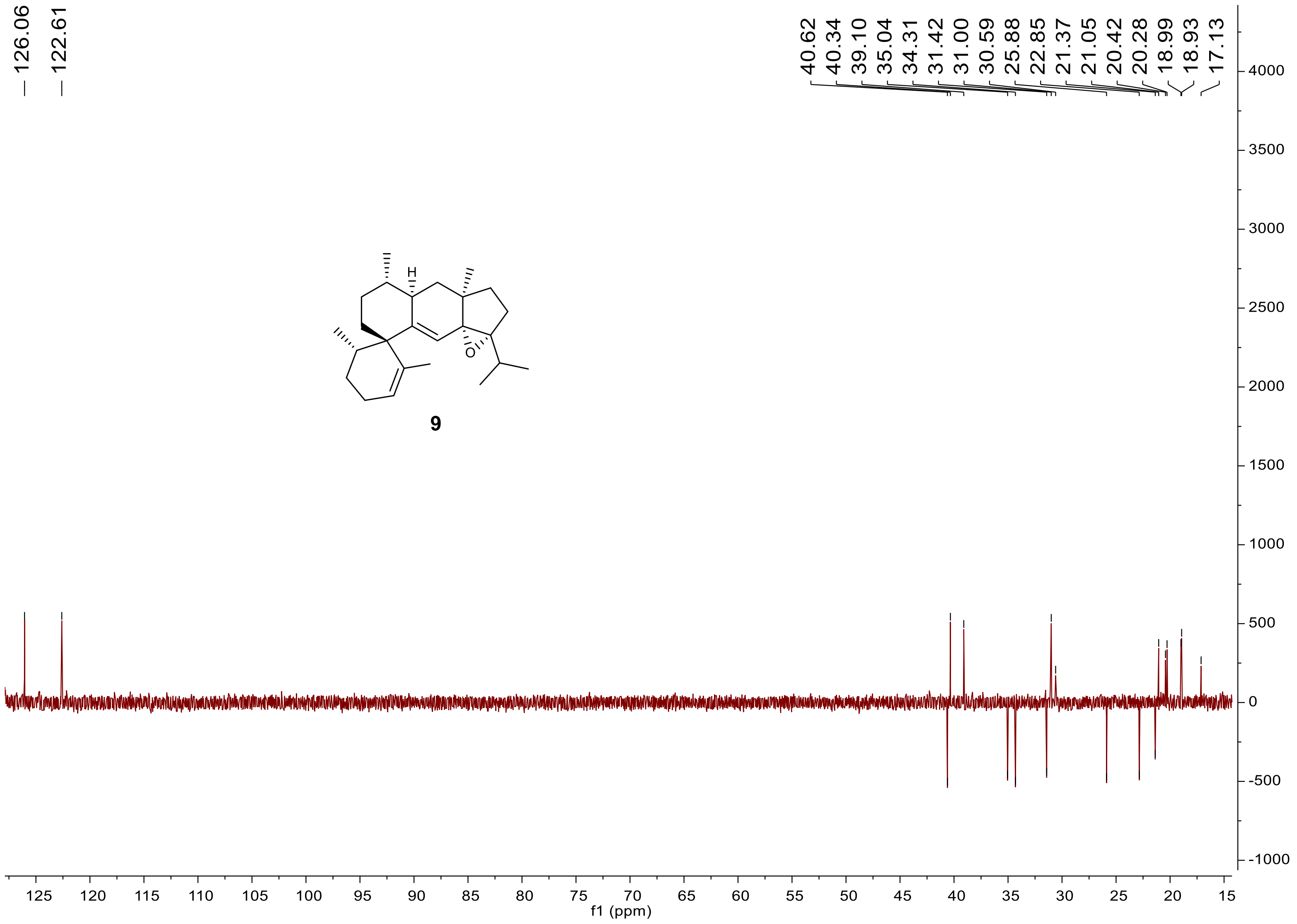


**Figure S84.** $^{13}$C-DEPT135 spectrum of **9** (176 MHz, $C_6D_6$).

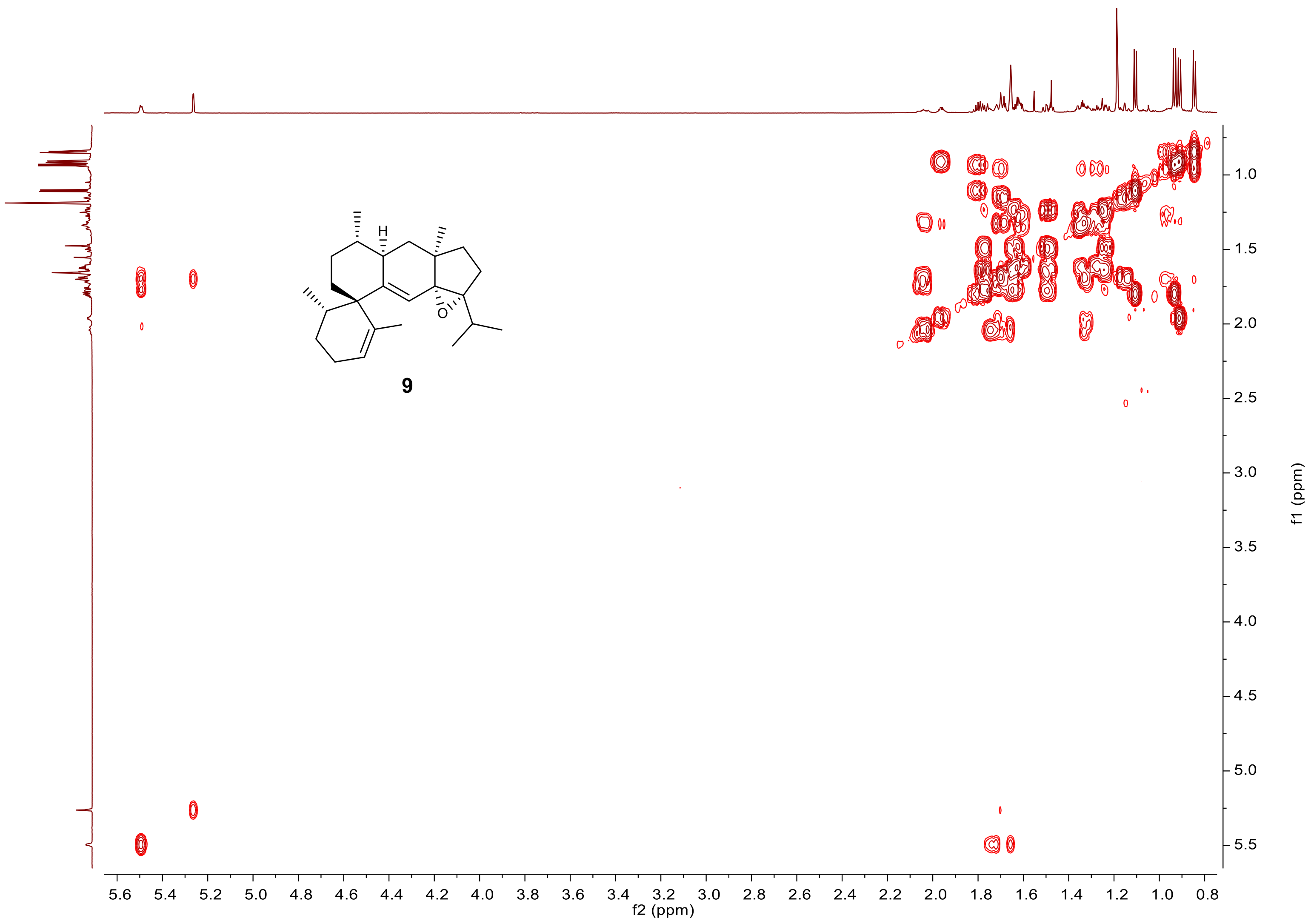


**Figure S85.** $^{1}H$-$^{1}H$-COSY spectrum ($C_6D_6$) of **9**.

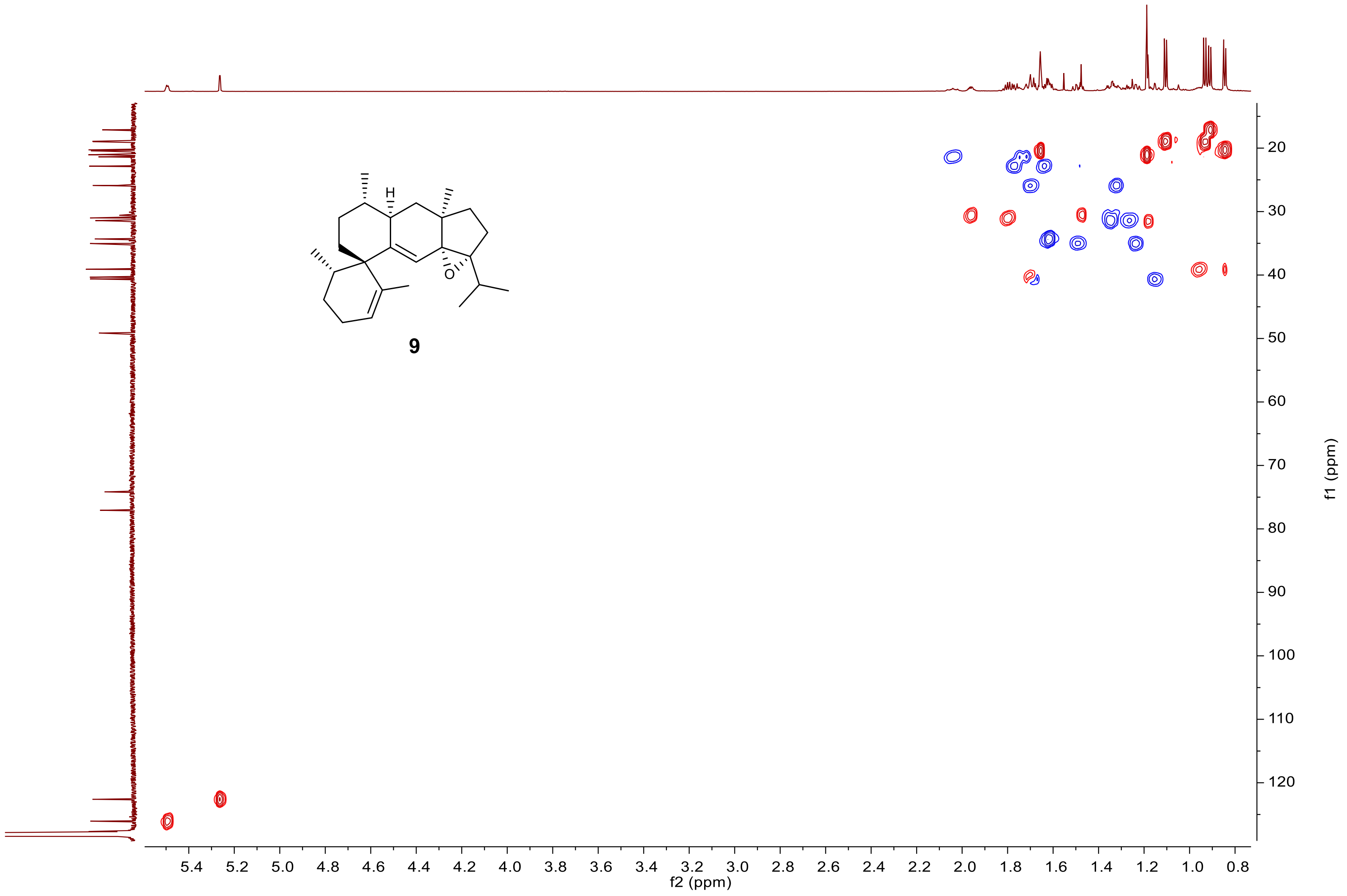


**Figure S86.** HSQC spectrum ($C_6D_6$) of **9**.

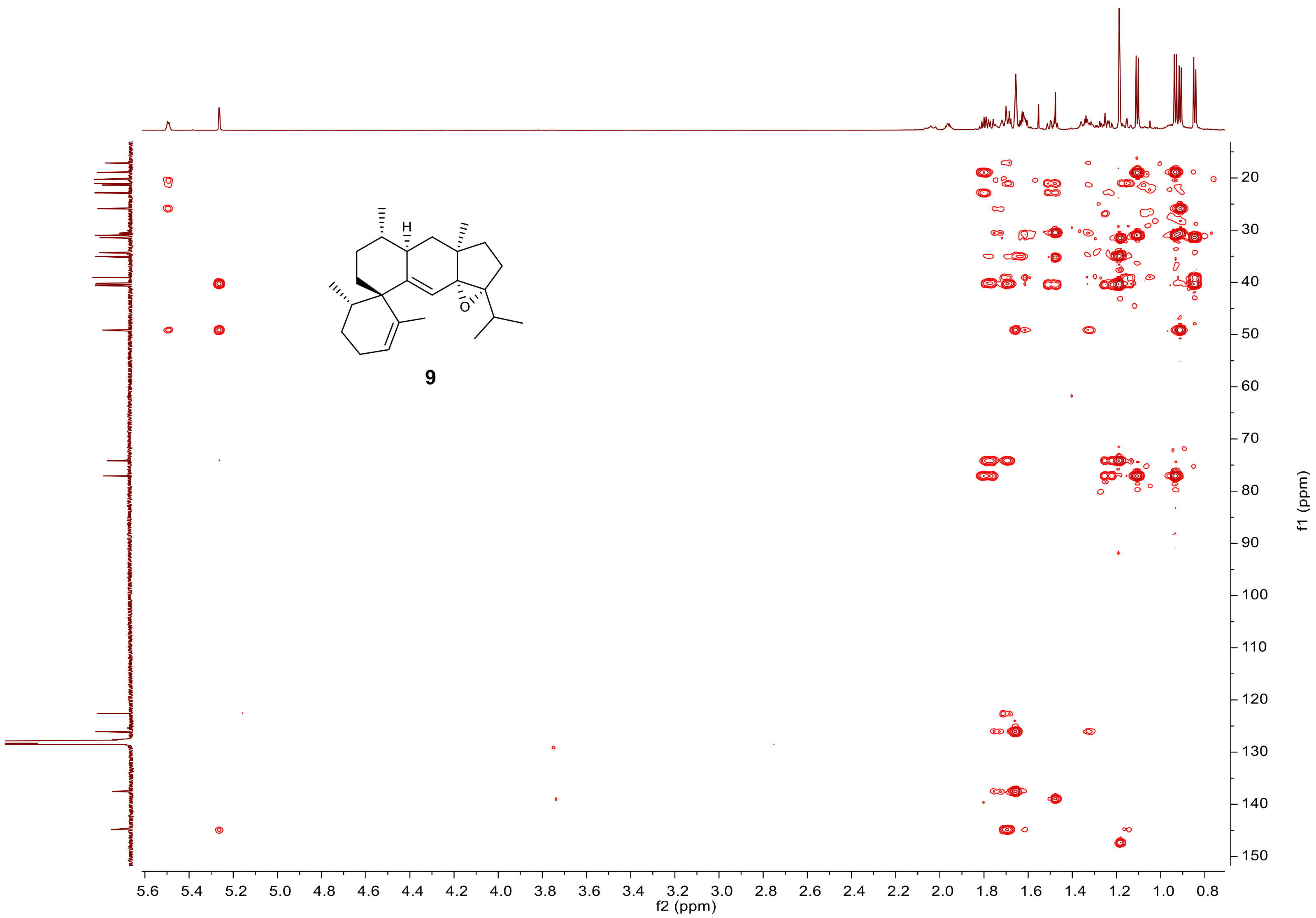


**Figure S87.** HMBC spectrum ($C_6D_6$) of **9**.

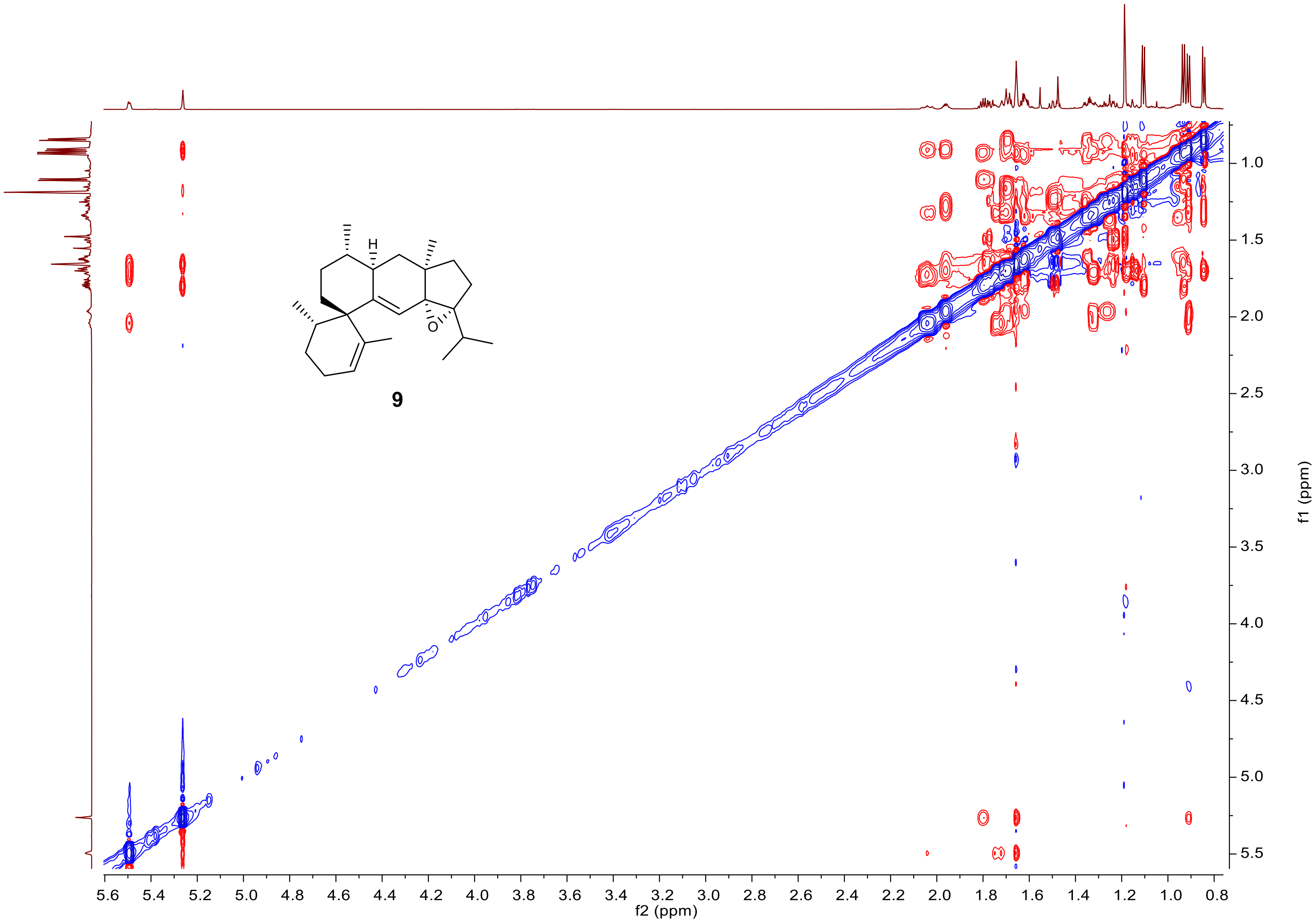


**Figure S88.** NOESY spectrum ($C_6D_6$) of **9**.

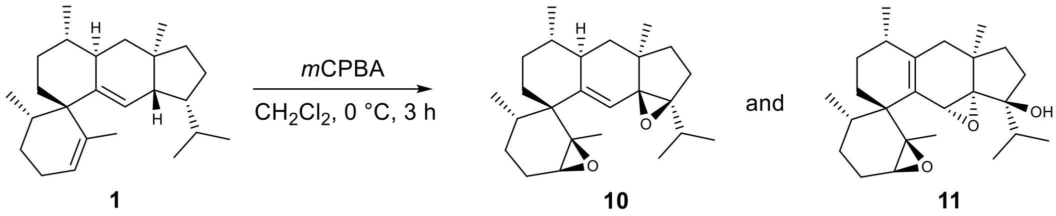

**Scheme S8.** Derivatisation of **1** with *meta*-chloroperoxybenzoic acid (*m*CPBA).

**Derivatisation of 1 with *m*CPBA**

Compound **1** (5.0 mg, 14.7 μmol, 1.0 eq.) was dissolved in dry $CH_2Cl_2$ (1.0 mL). After cooling to 0 °C, *meta*-chloroperoxybenzoic acid (*m*CPBA, 10.0 mg, 57.9 μmol, 3.9 eq.) was added and the reaction mixture was stirred at 0 °C for 3 h. The reaction was quenched by addition of $H_2O$ (1.0 mL). The mixture was extracted with $CH_2Cl_2$ (3 x 10 mL). The combined organic layers were washed with sat. NaCl (5 mL), dried over $MgSO_4$, and concentrated under reduced pressure. Purification by column chromatography on silica gel [petroether/ethyl acetate (5:1)] yielded the bis-epoxides **10** (0.9 mg, 2.4 μmol, 16%) and **11** (0.5 mg, 1.3 μmol, 9%) as colourless oils.

**Sesterviolene epoxide B (10).** TLC [petroether/ethyl acetate (5:1)]: $R_f$ = 0.6. IR (diamond ATR): ṽ = 2955 (s), 2929 (s), 2868 (m), 1773 (w), 1734 (w), 1686 (w), 1619 (w), 1567 (w), 1463 (m), 1376 (m), 1345 (w), 1310 (w), 1216 (w), 1107 (w), 1066 (w), 1016 (w), 975 (w), 918 (w), 898 (w), 876 (w), 864 (w), 831 (w), 734 (w), 544 (m), 488 (w) $cm^{-1}$. HR-MS (Q-TOF, 70 eV): *m*/*z* = 371.2943 (calc. for $[C_{25}H_{39}O_2]^+$ 371.2945). Optical rotary power: $[\alpha]_D^{20}$ = –2.4 (*c* 0.09, $CH_2Cl_2$). NMR data are given in Table S15.

**Sesterviolene epoxide C (11).** TLC [petroether/ethyl acetate (5:1)]: $R_f$ = 0.5. IR (diamond ATR): ṽ = 3491 (w), 2957 (s), 2932 (s), 2873 (m), 1730 (w), 1462 (m), 1380 (m), 1345 (w), 1309 (w), 1260 (w), 1156 (w), 1103 (w), 1072 (w), 1011 (w), 949 (w), 907 (w), 802 (w), 735 (w), 714 (w), 545 (m) $cm^{-1}$. HR-MS (Q-TOF, 70 eV): *m*/*z* = 369.2786 (calc. for $[C_{25}H_{39}O_3 - H_2O]^+$ 369.2788). Optical rotary power: $[\alpha]_D^{20}$ = –124.4 (*c* 0.05, $CH_2Cl_2$). NMR data are given in Table S16.

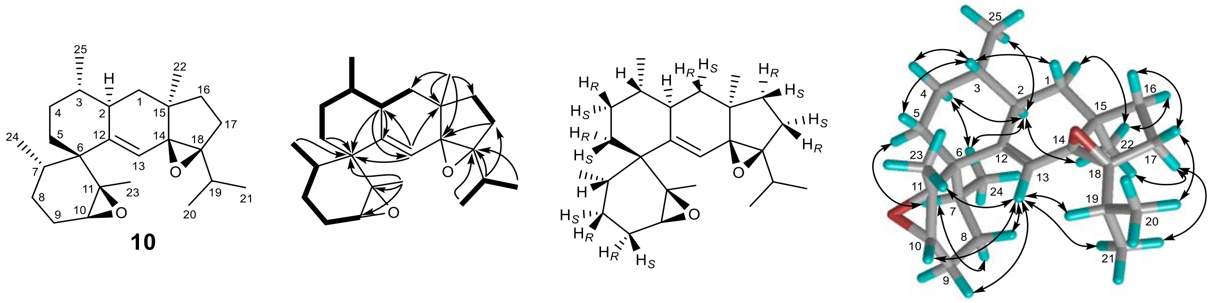


**Figure S89.** Structure elucidation of **10**. Bold: $^{1}$H,$^{1}$H-COSY, single headed arrows: key HMBC, and double headed arrows: NOESY correlations. Carbon numbering follows GFPP numbering to indicate the origin of each carbon. Diastereotopic hydrogens are labelled $H_R$ (*pro-R*) and $H_S$ (*pro-S*).

**Table S15.** NMR data of sesterviolene epoxide B (**10**) in $C_6D_6$ recorded at 298 K.

| C[a] | type | $^{1}$H[b] | $^{13}$C[b] |
|---|---|---|---|
| 1 | $CH_2$ | 1.83 (dd, *J* = 11.9, 5.8, $H_S$) | 37.27 |
| | | 1.50 (dd, *J* = 11.9, 10.5, $H_R$) | |
| 2 | CH | 1.78 (m) | 43.93 |
| 3 | CH | 1.18 (m) | 36.72 |
| 4 | $CH_2$ | 1.57 (m, $H_R$) | 34.86 |
| | | 1.32 (m, $H_S$) | |
| 5 | $CH_2$ | 2.21 (ddd, *J* = 14.9, 13.2, 6.3, $H_S$) | 33.76 |
| | | 1.76 (m, $H_R$) | |
| 6 | $C_q$ | – | 46.12 |
| 7 | CH | 1.99 (m) | 38.69 |
| 8 | $CH_2$ | 1.43 (m, $H_S$) | 24.84 |
| | | 0.90 (m, $H_R$) | |
| 9 | $CH_2$ | 1.89 (m, $H_S$) | 23.38 |
| | | 1.67 (dddd, *J* = 15.6, 8.2, 4.7, 1.0, $H_R$) | |
| 10 | CH | 2.81 (br d, *J* = 4.6) | 61.36 |
| 11 | $C_q$ | – | 64.40 |
| 12 | $C_q$ | – | 147.60 |
| 13 | CH | 5.38 (d, *J* = 2.3) | 122.06 |
| 14 | $C_q$ | – | 71.74 |
| 15 | $C_q$ | – | 38.98 |
| 16 | $CH_2$ | 1.43 (m, $H_S$) | 32.18 |
| | | 1.11 (m, $H_R$) | |
| 17 | $CH_2$ | 1.78 (m, $H_R$) | 24.70 |
| | | 1.61 (m, $H_S$) | |
| 18 | $C_q$ | – | 75.45 |
| 19 | CH | 1.93 (m) | 29.34 |
| 20 | $CH_3$ | 1.10 (d, *J* = 6.9) | 18.75 |
| 21 | $CH_3$ | 0.93 (d, *J* = 7.1) | 19.27 |
| 22 | $CH_3$ | 0.84 (s) | 21.09 |
| 23 | $CH_3$ | 1.38 (s) | 21.23 |
| 24 | $CH_3$ | 0.81 (d, *J* = 7.2) | 20.45 |
| 25 | $CH_3$ | 0.83 (d, *J* = 7.2) | 20.55 |

[a] Carbon numbering as shown in Figure S89. [b] Chemical shifts δ in ppm, multiplicity: s = singlet, d = doublet, m = multiplet, br = broad, coupling constants *J* are given in Hertz.

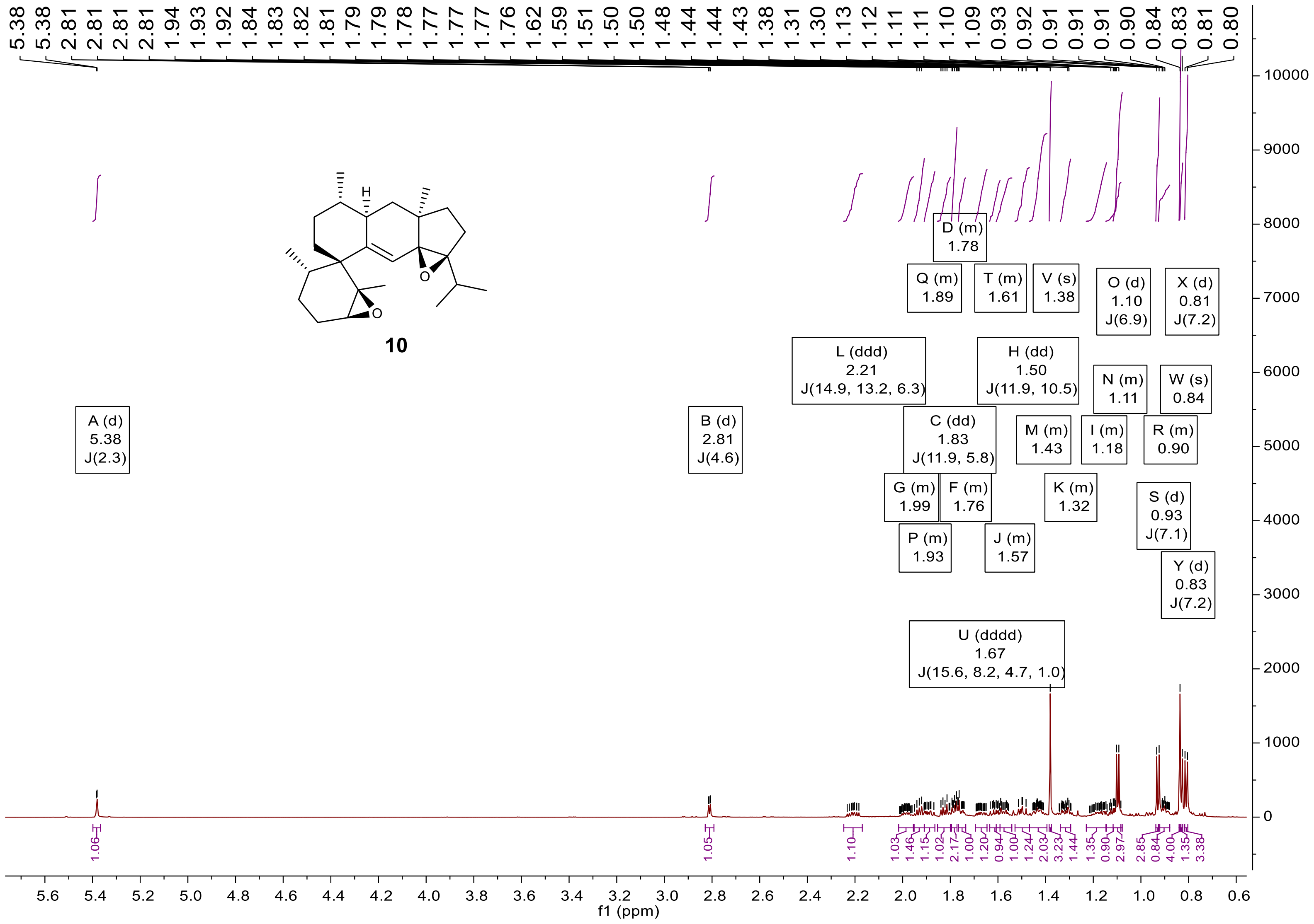

**Figure S90.** $^{1}$H-NMR spectrum of **10** (700 MHz, $C_6D_6$).

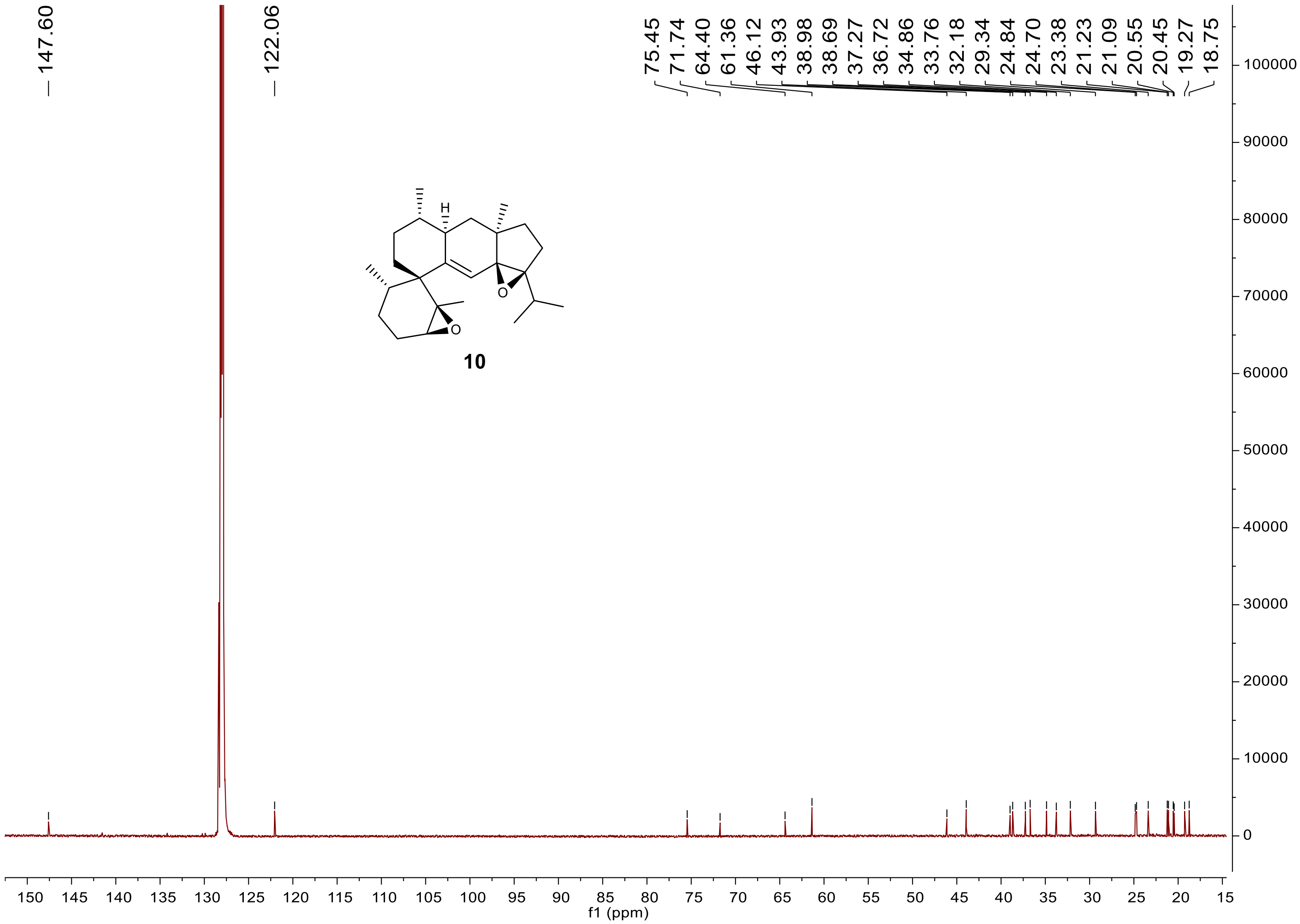


**Figure S91.** $^{13}$C-NMR spectrum of **10** (176 MHz, $C_6D_6$).

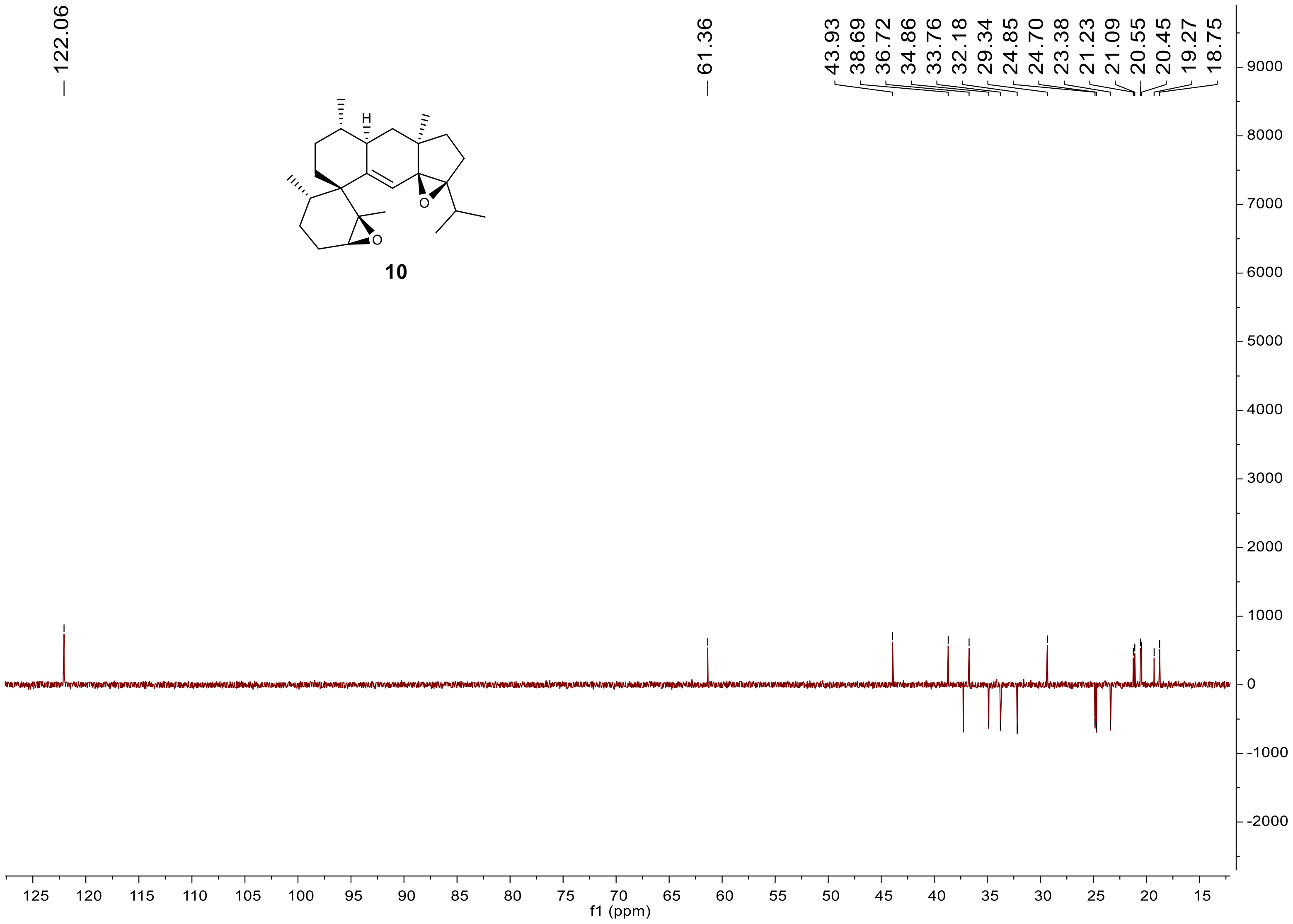


**Figure S92.** $^{13}$C-DEPT135 spectrum of **10** (176 MHz, $C_6D_6$).

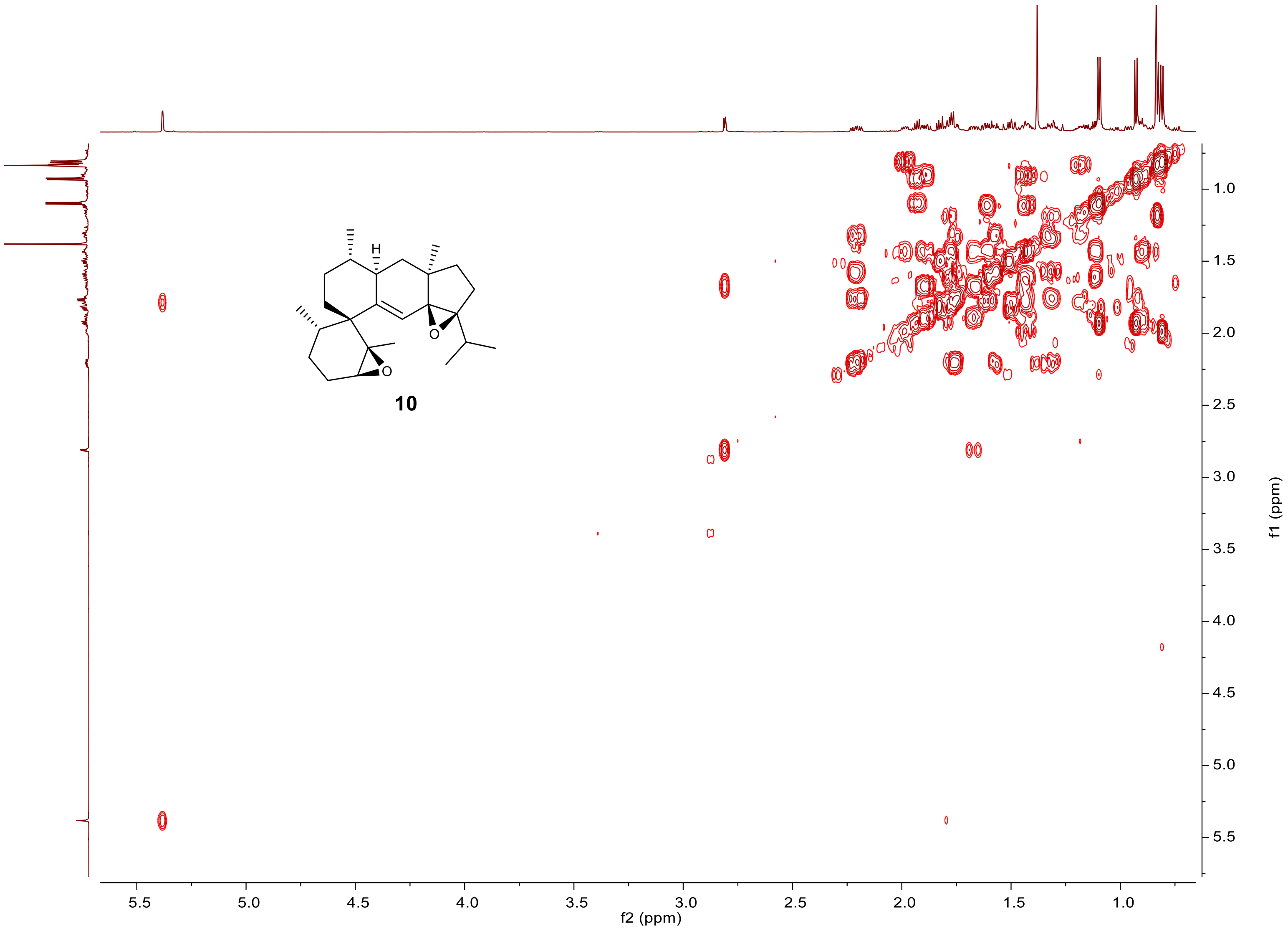


**Figure S93.** $^1$H-$^1$H-COSY spectrum ($C_6D_6$) of **10**.

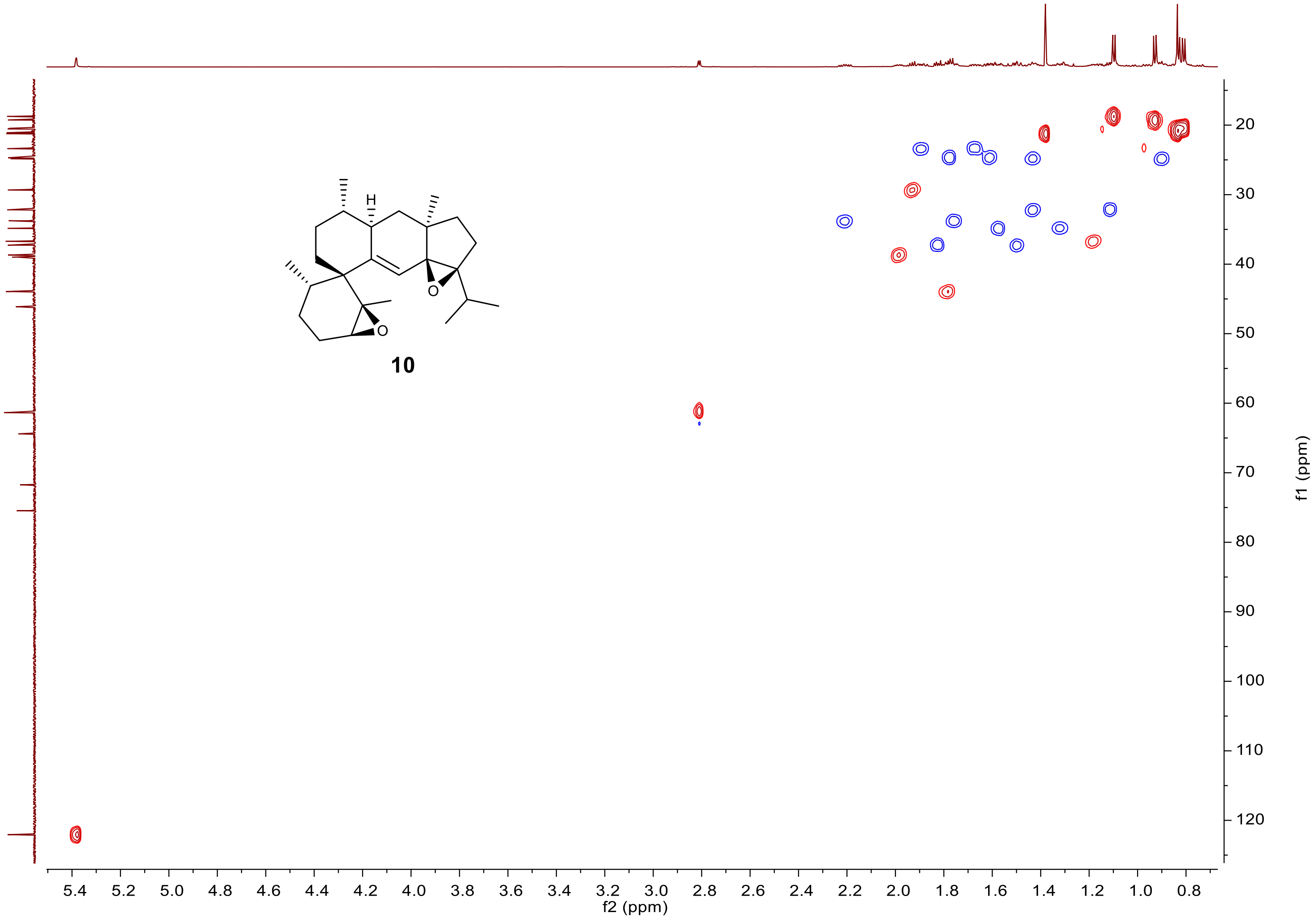


**Figure S94.** HSQC spectrum ($C_6D_6$) of **10**.

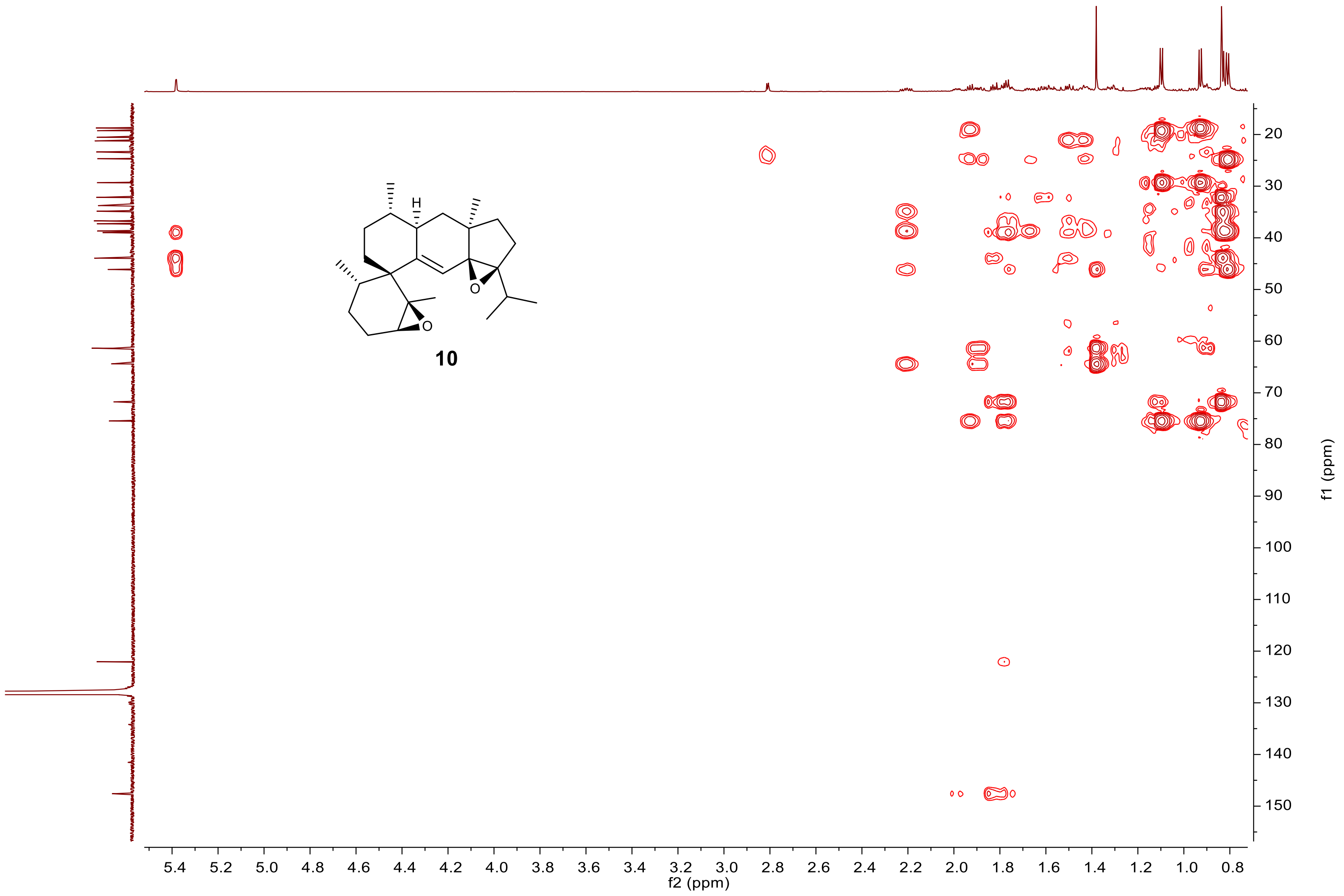


**Figure S95.** HMBC spectrum ($C_6D_6$) of **10**.

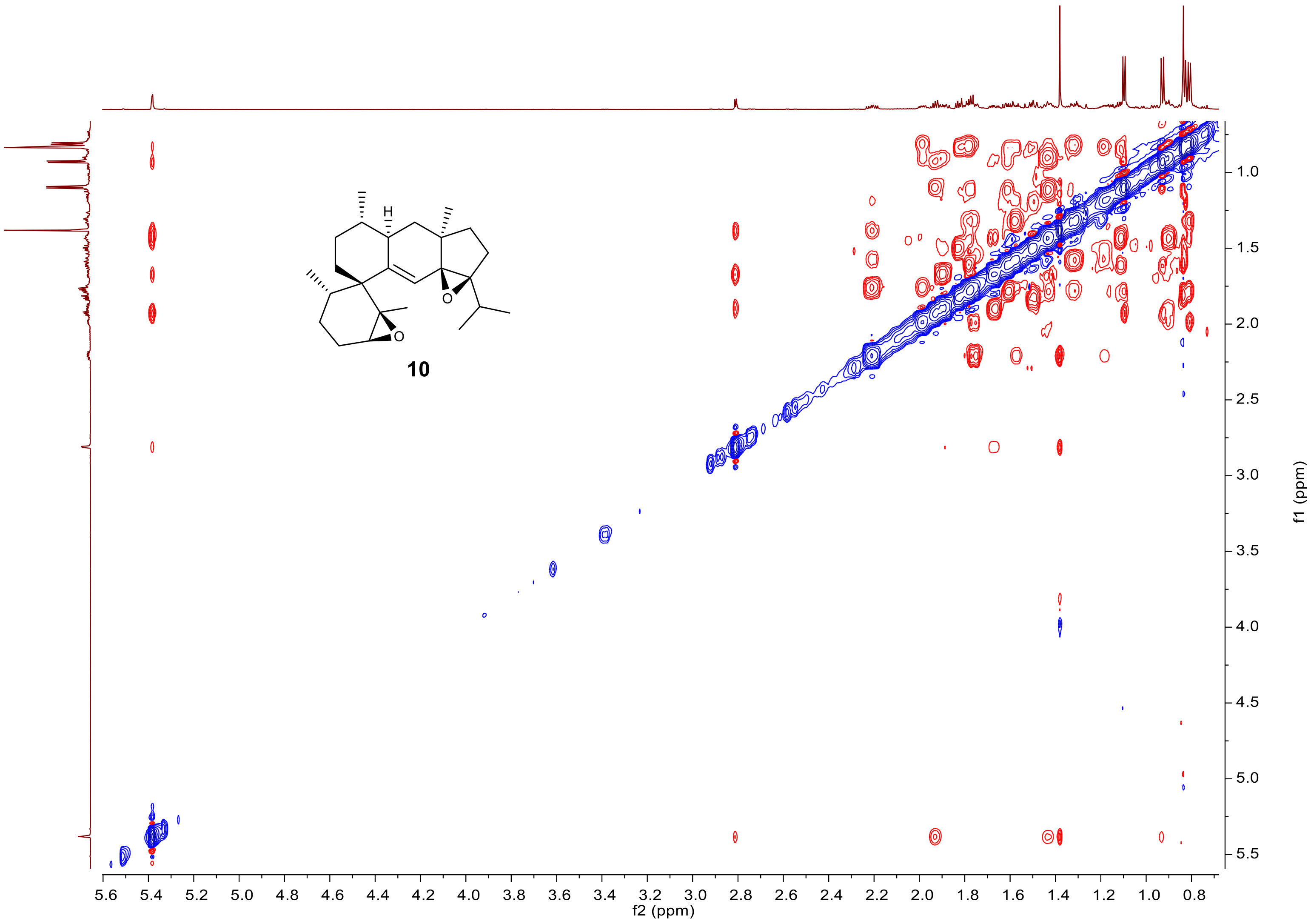


**Figure S96.** NOESY spectrum ($C_6D_6$) of **10**.

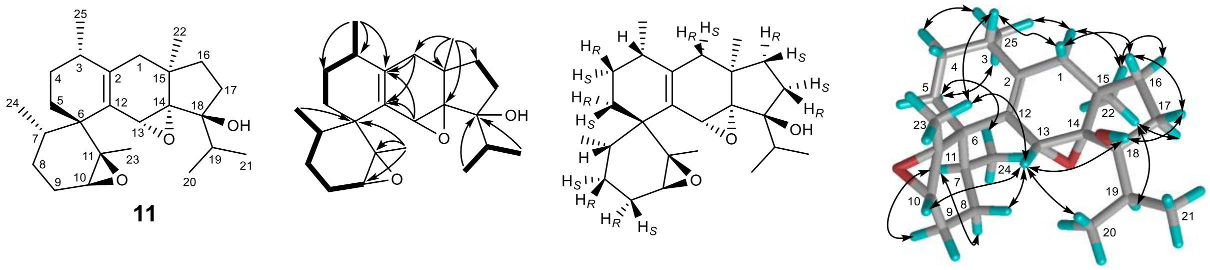


**Figure S97.** Structure elucidation of **11**. Bold: $^1$H,$^1$H-COSY, single headed arrows: key HMBC, and double headed arrows: NOESY correlations. Carbon numbering follows GFPP numbering to indicate the origin of each carbon. Diastereotopic hydrogens are labelled $H_R$ (*pro-R*) and $H_S$ (*pro-S*).

**Table S16.** NMR data of sesterviolene epoxide C (**11**) in $C_6D_6$ recorded at 298 K.

| C[a] | type | $^1$H[b] | $^{13}$C[b] |
|---|---|---|---|
| 1 | $CH_2$ | 2.27 (dt, *J* = 16.0, 1.2, $H_R$)<br>1.56 (d, *J* = 16.0, $H_S$) | 42.07 |
| 2 | $C_q$ | – | 144.79 |
| 3 | CH | 1.82 (m) | 33.60 |
| 4 | $CH_2$ | 1.76 (m, $H_R$)<br>1.30 (m, $H_S$) | 28.42 |
| 5 | $CH_2$ | 1.87 (m, $H_S$)<br>1.69 (m, $H_R$) | 22.95 |
| 6 | $C_q$ | – | 44.31 |
| 7 | CH | 1.72 (m) | 35.72 |
| 8 | $CH_2$ | 1.30 (m, $H_S$)<br>1.22 (m, $H_R$) | 27.31 |
| 9 | $CH_2$ | 2.09 (m, $H_S$)<br>1.69 (m, $H_R$) | 28.75 |
| 10 | CH | 2.78 (br d, *J* = 4.7) | 63.45 |
| 11 | $C_q$ | – | 63.68 |
| 12 | $C_q$ | – | 127.56 |
| 13 | CH | 3.87 (s) | 57.31 |
| 14 | $C_q$ | – | 78.22 |
| 15 | $C_q$ | – | 39.71 |
| 16 | $CH_2$ | 1.71 (m, $H_S$)<br>1.39 (m, $H_R$) | 35.33 |
| 17 | $CH_2$ | 2.02 (m, $H_R$)<br>1.97 (m, $H_S$) | 38.04 |
| 18 | $C_q$ | – | 79.15 |
| 19 | CH | 1.79 (m) | 37.53 |
| 20 | $CH_3$ | 1.11 (d, *J* = 6.6) | 18.22 |
| 21 | $CH_3$ | 1.14 (d, *J* = 6.8) | 18.64 |
| 22 | $CH_3$ | 0.89 (s) | 21.69 |
| 23 | $CH_3$ | 1.47 (s) | 22.48 |
| 24 | $CH_3$ | 0.77 (d, *J* = 6.9) | 19.30 |
| 25 | $CH_3$ | 0.87 (d, *J* = 7.2) | 17.50 |
| 18-OH | OH | 3.19 (s) | |

[a] Carbon numbering as shown in Figure S97. [b] Chemical shifts δ in ppm, multiplicity: s = singlet, d = doublet, t = triplet, m = multiplet, br = broad, coupling constants *J* are given in Hertz.

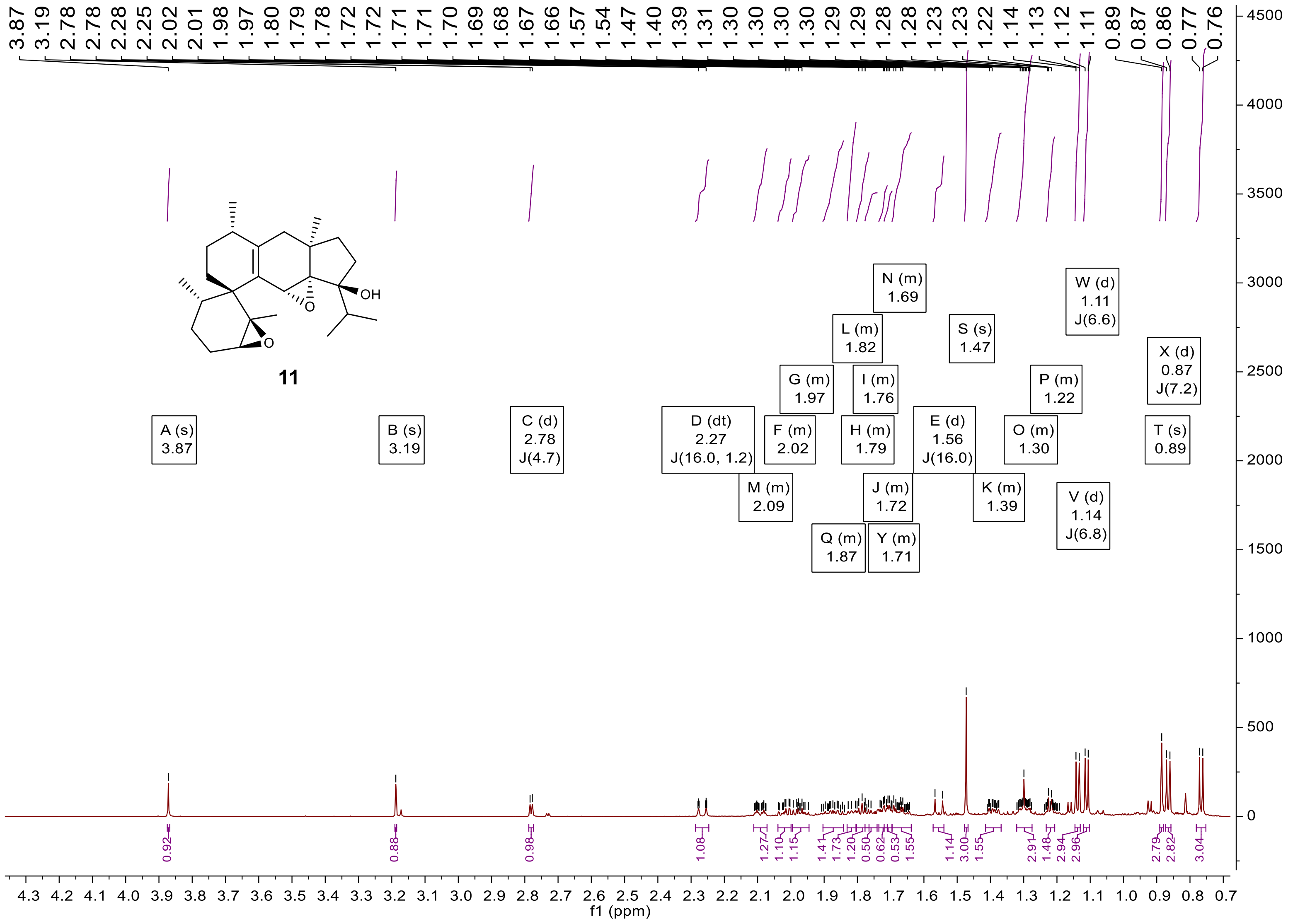


**Figure S98.** $^{1}$H-NMR spectrum of **11** (700 MHz, $C_6D_6$).

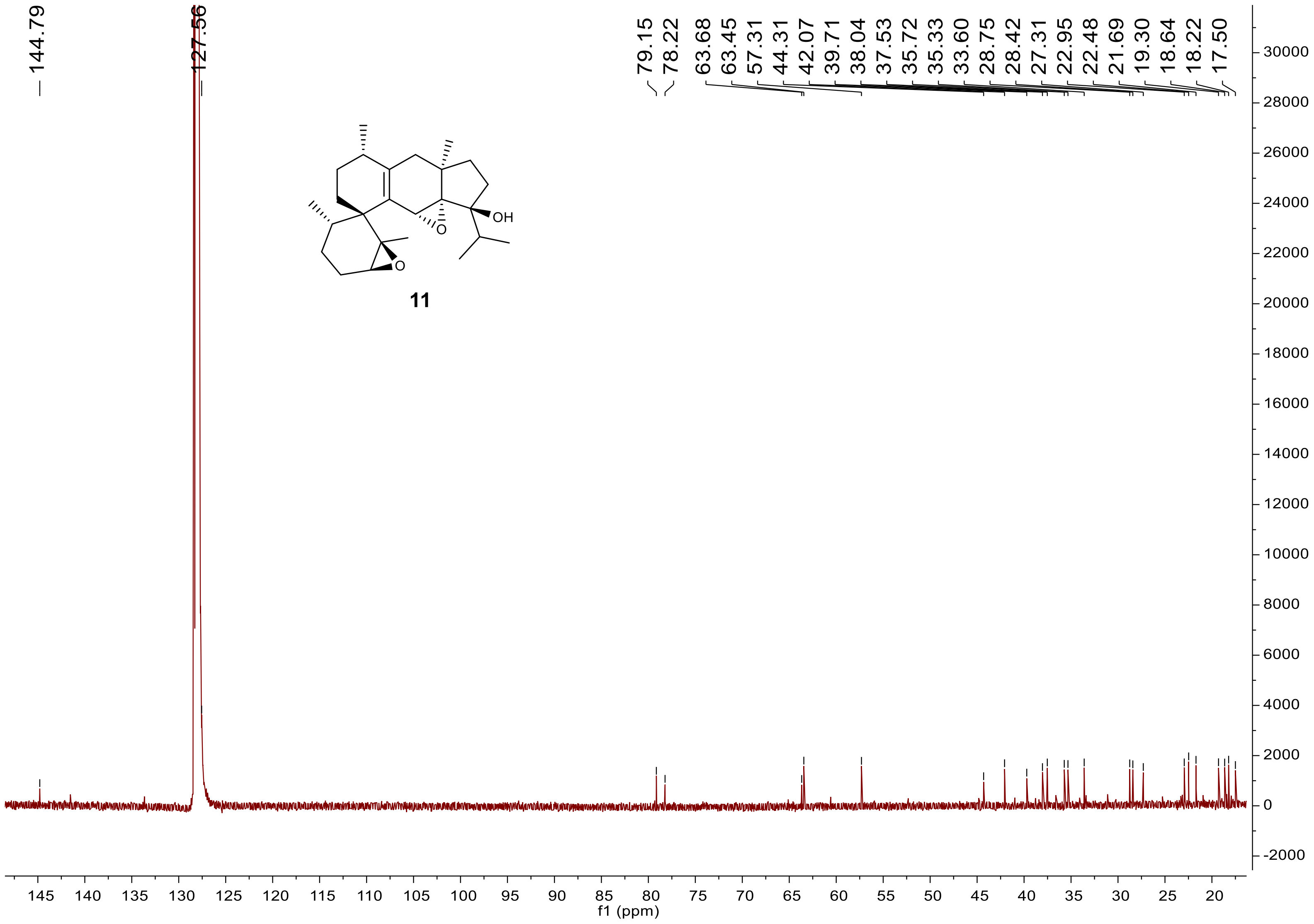


**Figure S99.** $^{13}$C-NMR spectrum of **11** (176 MHz, $C_6D_6$).

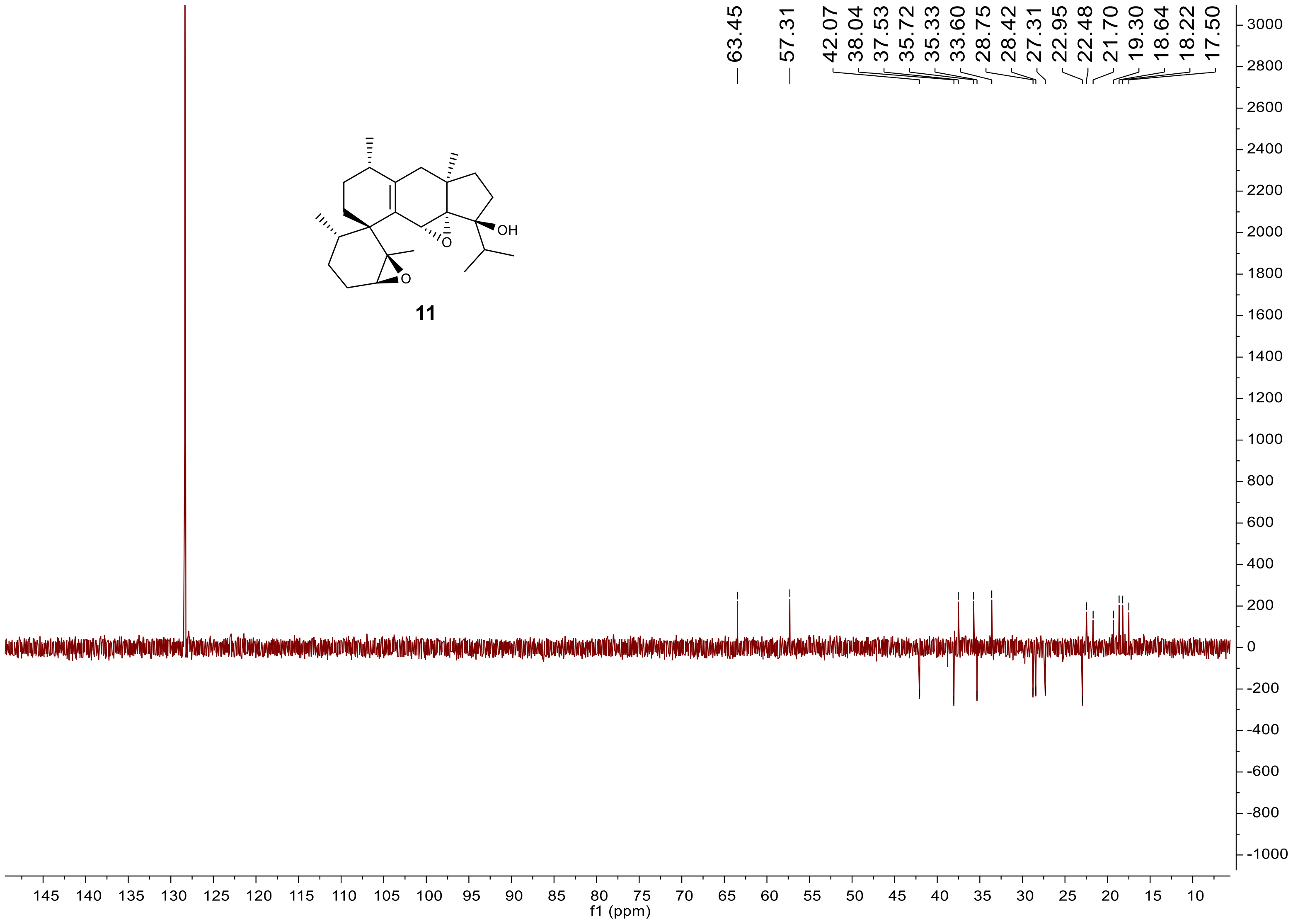


**Figure S100.** $^{13}C$-DEPT135 spectrum of **11** (176 MHz, $C_6D_6$).

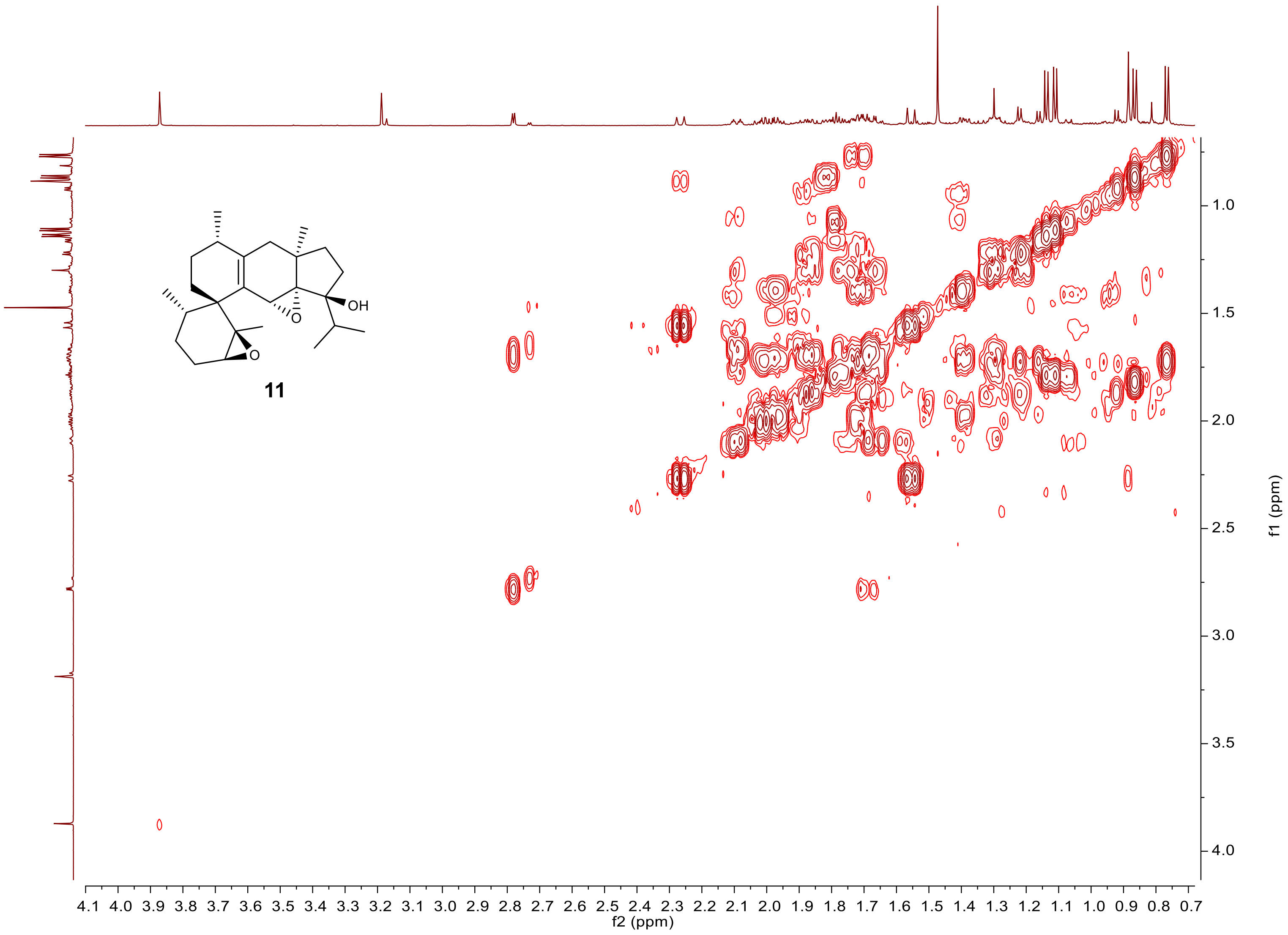


**Figure S101.** $^{1}$H-$^{1}$H-COSY spectrum ($C_6D_6$) of **11**.

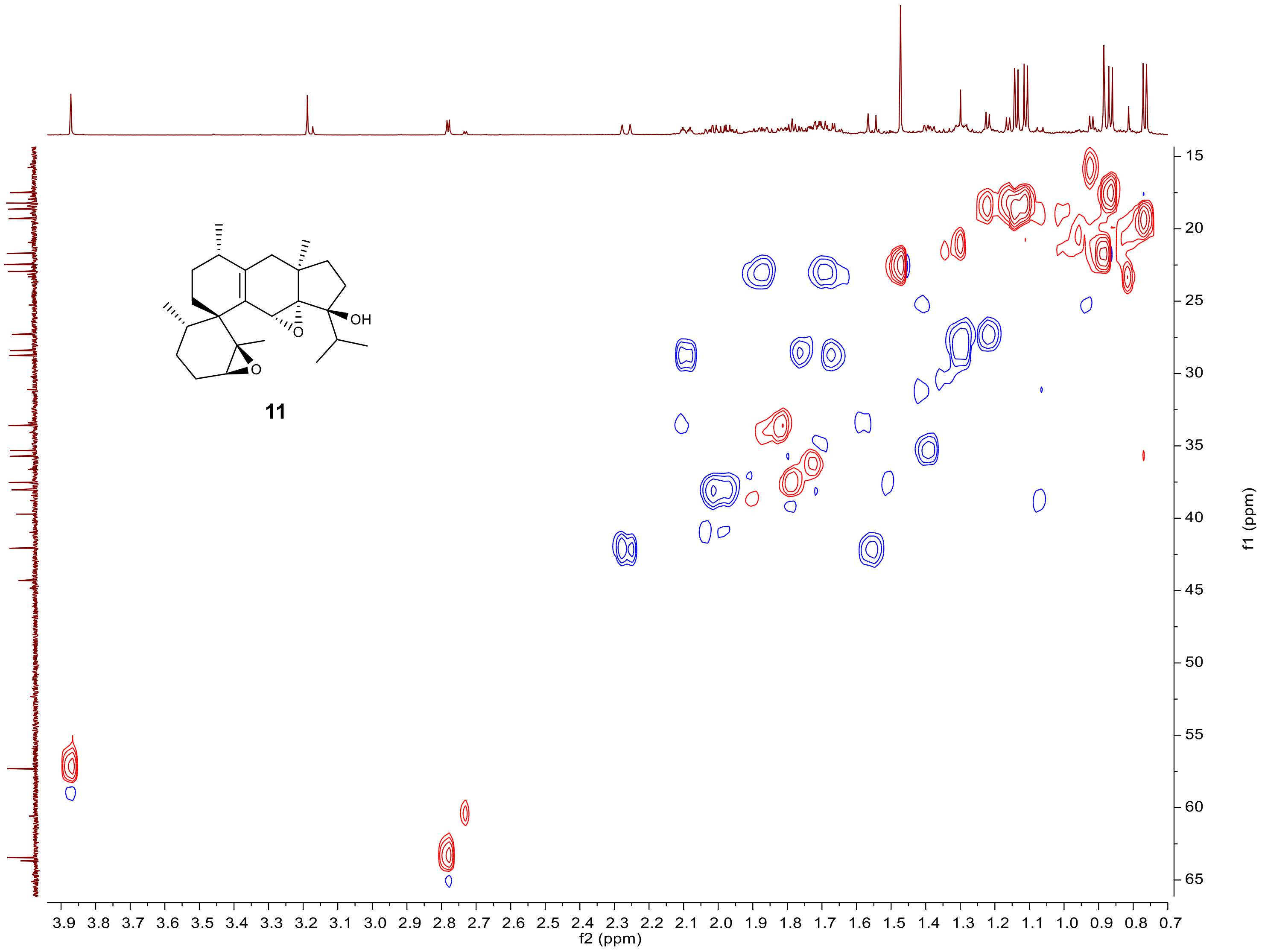


**Figure S102.** HSQC spectrum ($C_6D_6$) of **11**.

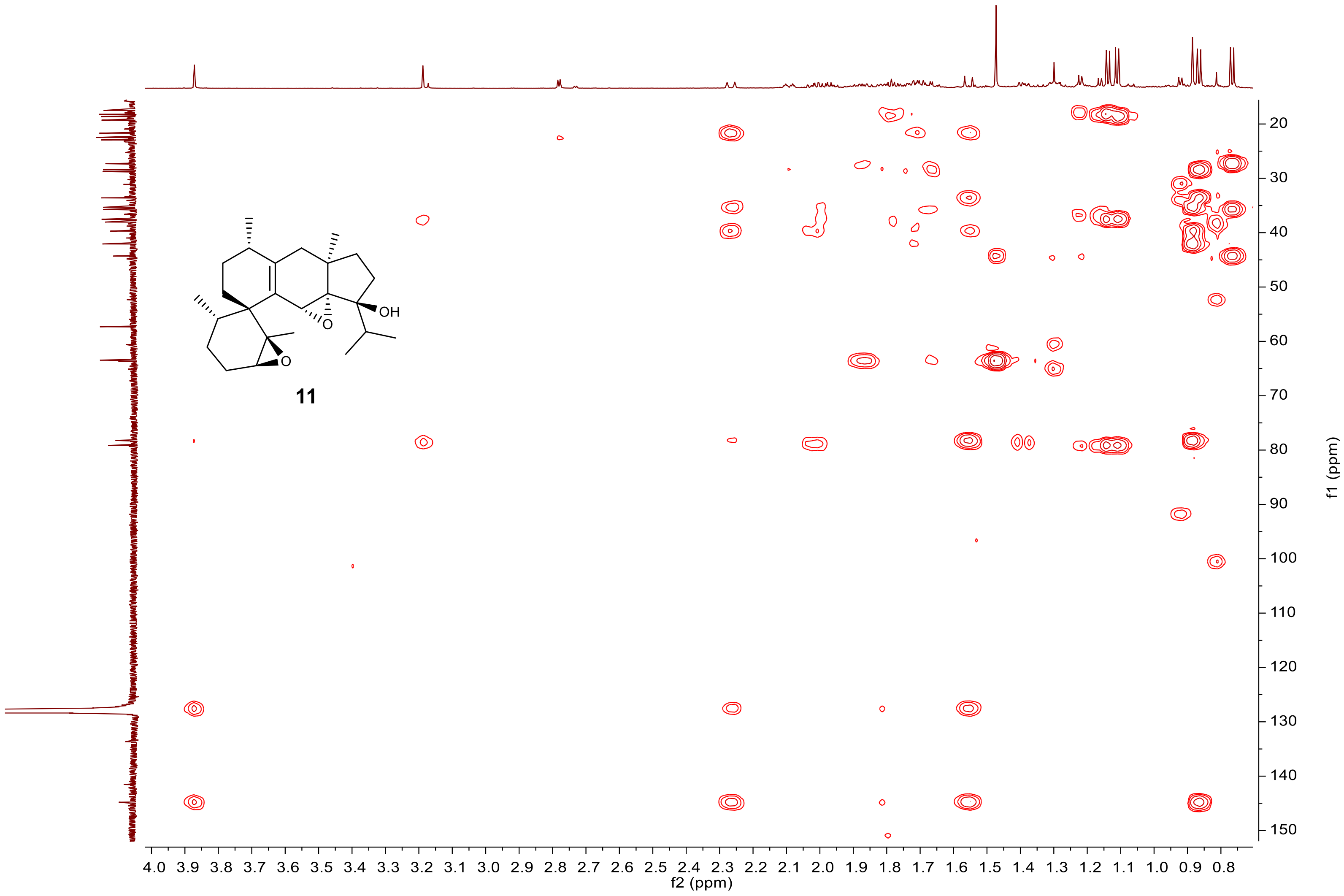


**Figure S103.** HMBC spectrum ($C_6D_6$) of **11**.

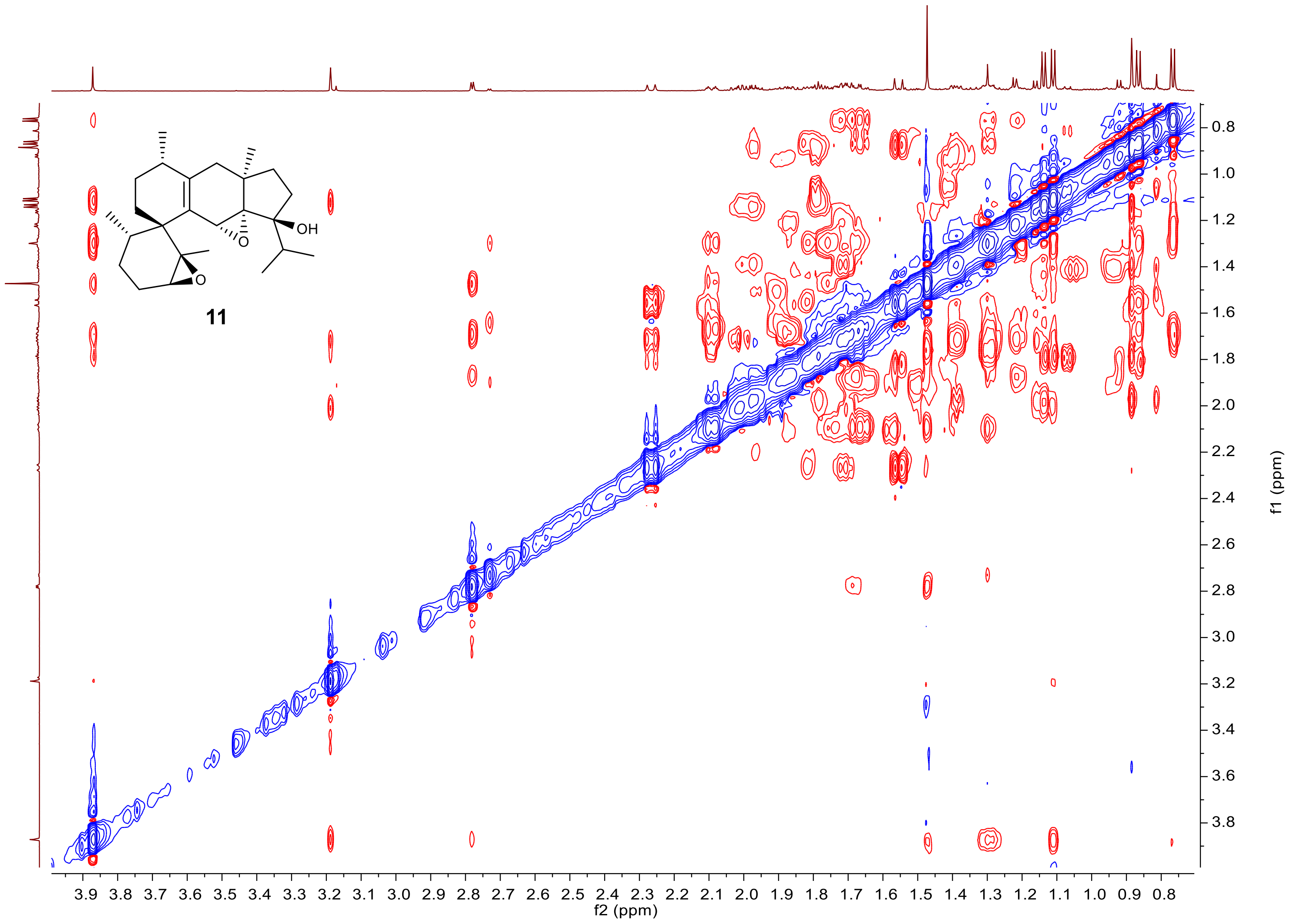


**Figure S104.** NOESY spectrum ($C_6D_6$) of **11**.

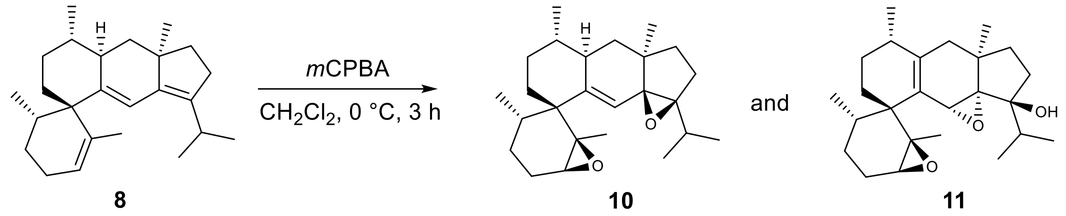

**Scheme S9.** Derivatisation of **8** with *meta*-chloroperoxybenzoic acid (*m*CPBA).

**Derivatisation of 8 with *m*CPBA**

Compound **8** (2.2 mg, 6.5 μmol, 1.0 eq.) was dissolved in dry $CH_2Cl_2$ (1.0 mL). After cooling to 0 °C, *meta*-chloroperoxybenzoic acid (*m*CPBA, 4.4 mg, 25.5 μmol, 3.9 eq.) was added and the reaction mixture was stirred at 0 °C for 3 h. The reaction was quenched by the addition of $H_2O$ (1.0 mL). The mixture was extracted with $CH_2Cl_2$ (3 x 10 mL). The combined organic layers were washed with sat. NaCl (5 mL), dried over $MgSO_4$, and concentrated under reduced pressure. Purification by column chromatography on silica gel [petroether/ethyl acetate (5:1)] yielded the bis-epoxides **10** (0.4 mg, 1.1 μmol, 17%) and **11** (0.1 mg, 0.26 μmol, 4.0%) as colourless oils. The structures of these two molecules were confirmed by comparing their $^1$H-NMR spectra with those of compounds **10** and **11** obtained from the treatment of **1** with *m*CPBA (Figures S105 and S106).

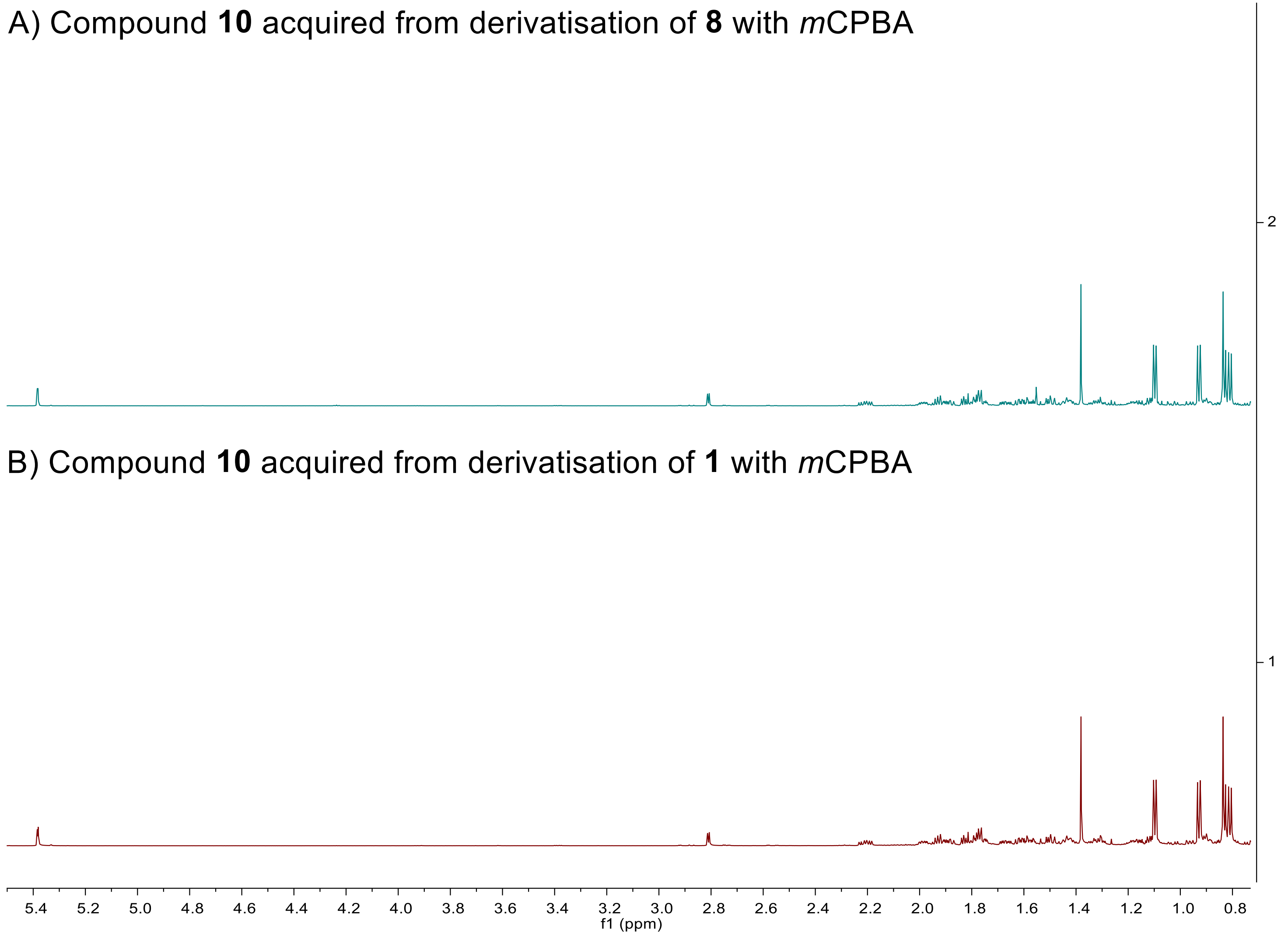


**Figure S105.** $^{1}$H-NMR spectra (700 MHz, $C_6D_6$) of **10** acquired from derivatisation A) of **8**, and B) of **1** with *m*CPBA.

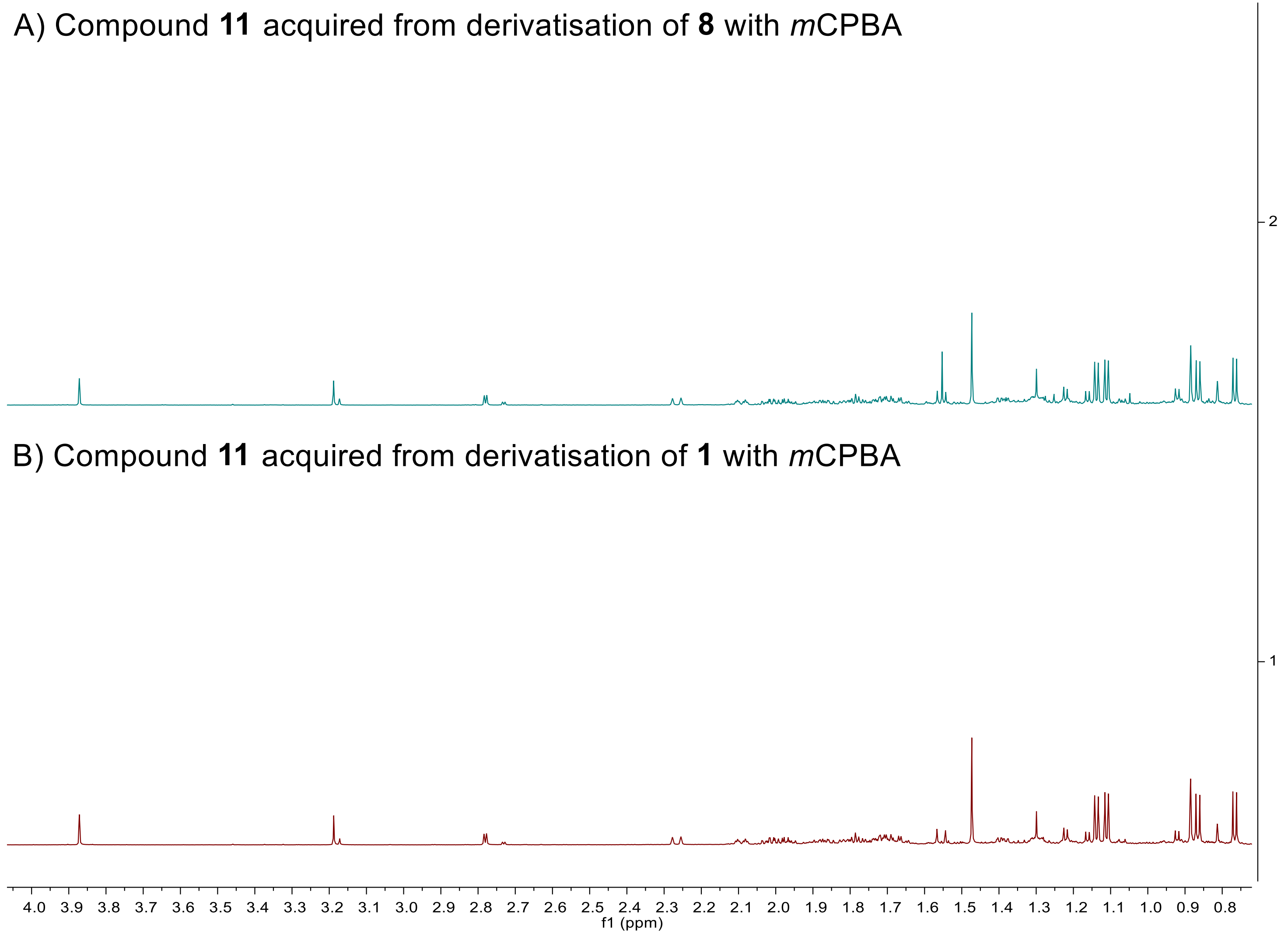


**Figure S106.** $^{1}H$-NMR spectra (700 MHz, $C_6D_6$) of **11** acquired from derivatisation A) of **8**, and B) of **1** with *m*CPBA.

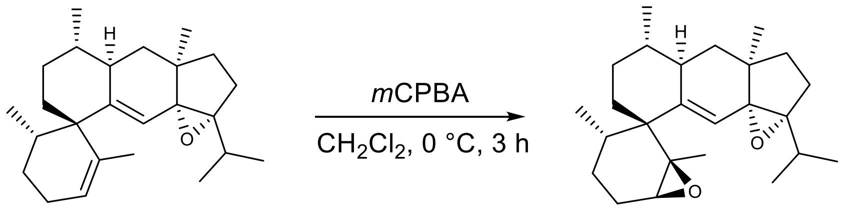


**9** **12**

**Scheme S10.** Derivatisation of **9** with *meta*-chloroperoxybenzoic acid (*m*CPBA).

**Derivatisation of 9 with *m*CPBA**

Compound **9** (0.6 mg, 1.7 μmol, 1.0 eq.) was dissolved in dry $CH_2Cl_2$ (1.0 mL). After cooling to 0 °C, *meta*-chloroperoxybenzoic acid (*m*CPBA, 1.2 mg, 6.8 μmol, 4.0 eq.) was added and the reaction mixture was stirred at 0 °C for 3 h. The reaction was quenched by the addition of $H_2O$ (1.0 mL). The mixture was extracted with $CH_2Cl_2$ (3 x 5 mL). The combined organic layers were washed with sat. NaCl (5 mL), dried over $MgSO_4$, and concentrated under reduced pressure. Purification by column chromatography on silica gel [petroether/ethyl acetate (5:1)] yielded bis-epoxide **12** (0.4 mg, 1.1 μmol, 65%) as colourless oil.

**Sesterviolene epoxide D (12).** TLC [petroether/ethyl acetate (5:1)]: $R_f$ = 0.6. IR (diamond ATR): ṽ = 2958 (s), 2928 (s), 2868 (m), 1772 (w), 1731 (w), 1682 (w), 1464 (m), 1375 (w), 1260 (m), 1216 (w), 1087 (s), 1017 (s), 913 (w), 868 (w), 797 (s), 735 (w), 696 (w), 527 (w) $cm^{-1}$. HR-MS (Q-TOF, 70 eV): *m*/*z* = 371.2942 (calc. for $[C_{25}H_{39}O_2]^+$ 371.2945). Optical rotary power: $[\alpha]_D^{20}$ = –46.0 (*c* 0.04, $CH_2Cl_2$). NMR data are given in Table S17.

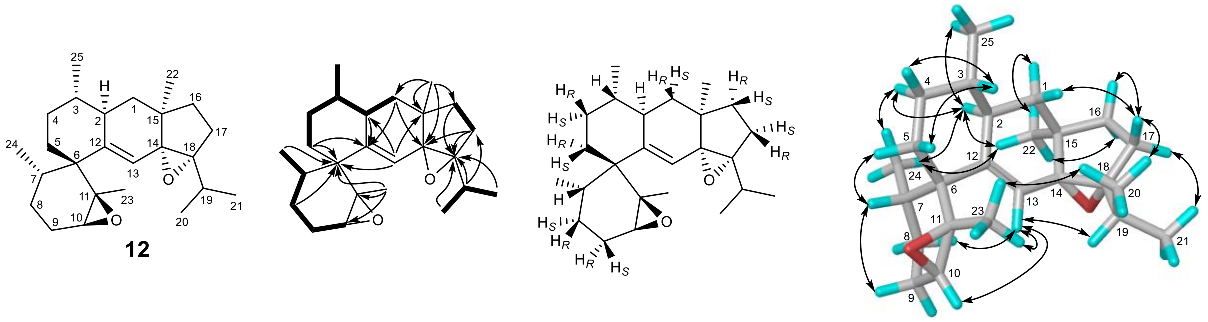

**Figure S107.** Structure elucidation of **12**. Bold: $^{1}$H,$^{1}$H-COSY, single headed arrows: key HMBC, and double headed arrows: NOESY correlations. Carbon numbering follows GFPP numbering to indicate the origin of each carbon. Diastereotopic hydrogens are labelled $H_R$ (*pro-R*) and $H_S$ (*pro-S*).

**Table S17.** NMR data of sesterviolene epoxide D (**12**) in $C_6D_6$ recorded at 298 K.

| C[a] | type | $^{1}$H[b] | $^{13}$C[b] |
|---|---|---|---|
| 1 | $CH_2$ | 1.61 (m, $H_S$) | 40.28 |
| | | 1.12 (m, $H_R$) | |
| 2 | CH | 1.84 (m) | 42.90 |
| 3 | CH | 1.00 (m) | 37.32 |
| 4 | $CH_2$ | 1.59 (m, $H_R$) | 34.72 |
| | | 1.34 (m, $H_S$) | |
| 5 | $CH_2$ | 2.15 (ddd, *J* = 14.9, 13.3, 6.3, $H_S$) | 33.75 |
| | | 1.78 (m, $H_R$) | |
| 6 | $C_q$ | – | 46.02 |
| 7 | CH | 1.95 (m) | 38.57 |
| 8 | $CH_2$ | 1.53 (m, $H_S$) | 24.95 |
| | | 0.85 (m, $H_R$) | |
| 9 | $CH_2$ | 1.77 (m, $H_S$) | 23.10 |
| | | 1.52 (m, $H_R$) | |
| 10 | CH | 2.73 (dd, *J* = 4.2, 1.1) | 61.66 |
| 11 | $C_q$ | – | 63.97 |
| 12 | $C_q$ | – | 144.92 |
| 13 | CH | 5.33 (d, *J* = 2.2) | 124.87 |
| 14 | $C_q$ | – | 74.53 |
| 15 | $C_q$ | – | 39.84 |
| 16 | $CH_2$ | 1.49 (m, $H_R$) | 34.48 |
| | | 1.24 (m, $H_S$) | |
| 17 | $CH_2$ | 1.77 (m, $H_S$) | 22.80 |
| | | 1.63 (m, $H_R$) | |
| 18 | $C_q$ | – | 77.27 |
| 19 | CH | 1.82 (m) | 30.90 |
| 20 | $CH_3$ | 0.90 (d, *J* = 7.0) | 19.33 |
| 21 | $CH_3$ | 1.13 (d, *J* = 6.9) | 18.92 |
| 22 | $CH_3$ | 1.15 (s) | 20.40 |
| 23 | $CH_3$ | 1.31 (s) | 21.51 |
| 24 | $CH_3$ | 0.85 (d, *J* = 7.2) | 20.73 |
| 25 | $CH_3$ | 0.81 (d, *J* = 6.5) | 20.10 |

[a] Carbon numbering as shown in Figure S107. [b] Chemical shifts δ in ppm, multiplicity: s = singlet, d = doublet, m = multiplet, coupling constants *J* are given in Hertz.

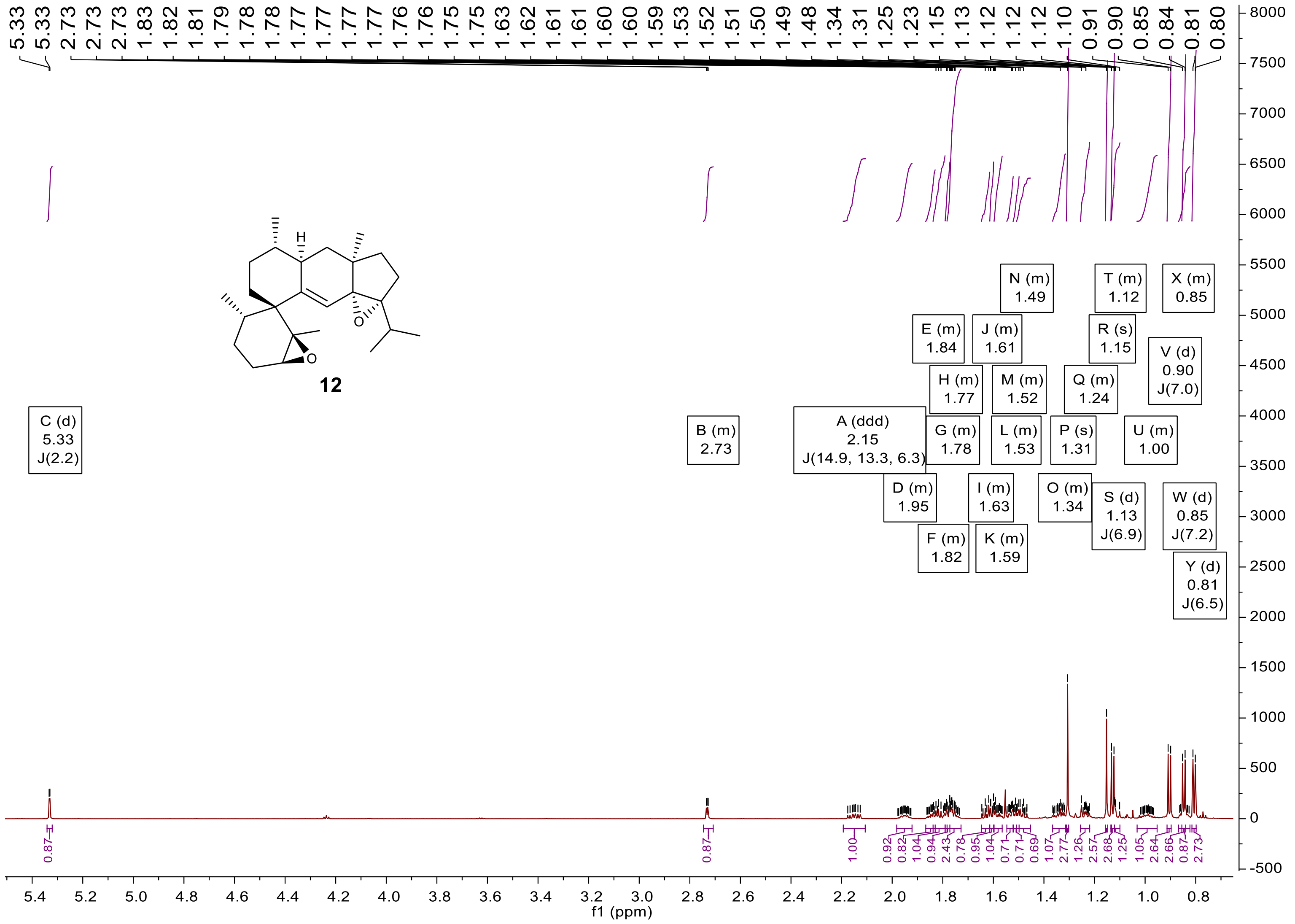


**Figure S108.** $^{1}$H-NMR spectrum of **12** (700 MHz, $C_6D_6$).

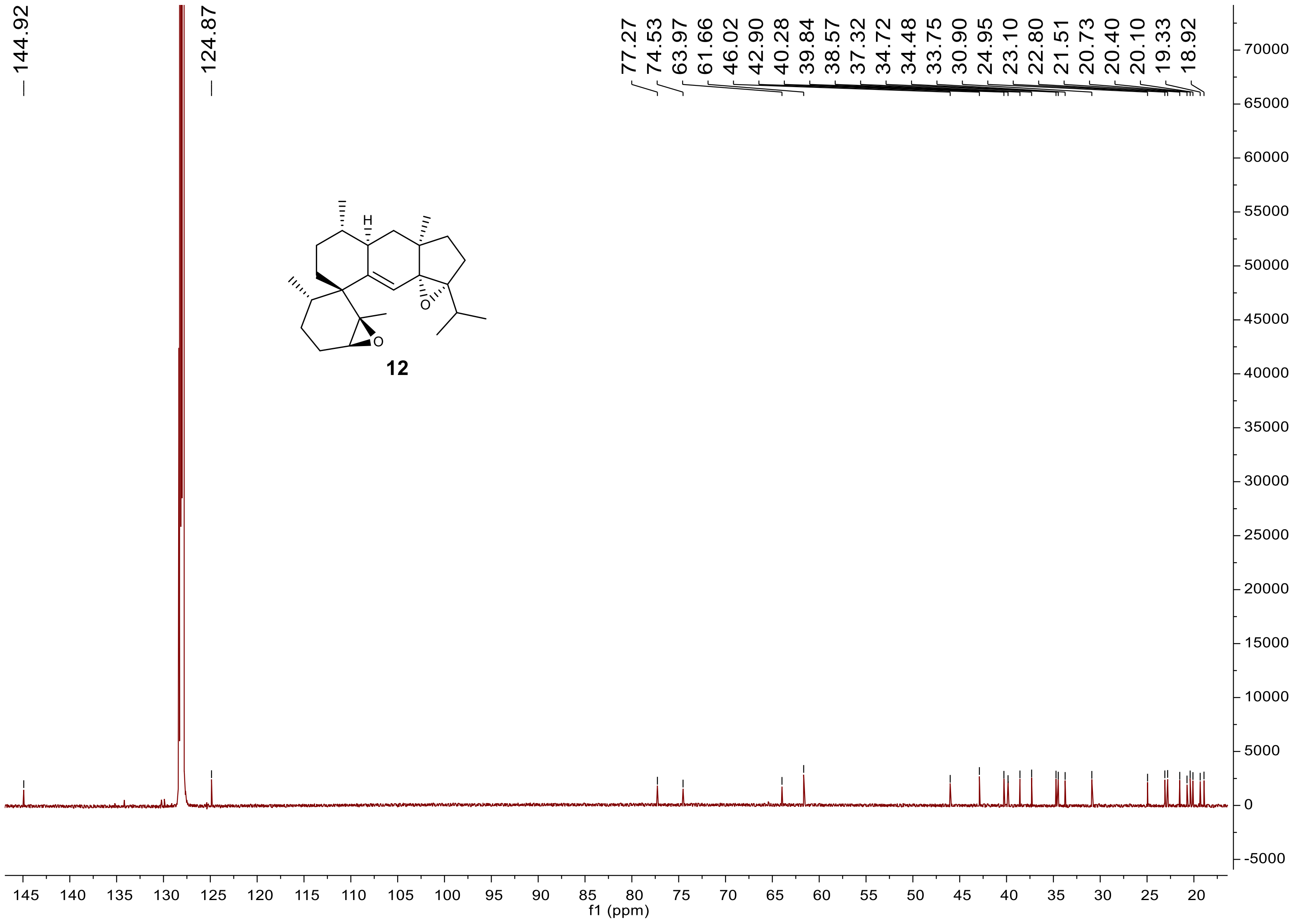


**Figure S109.** $^{13}$C-NMR spectrum of **12** (176 MHz, $C_6D_6$).

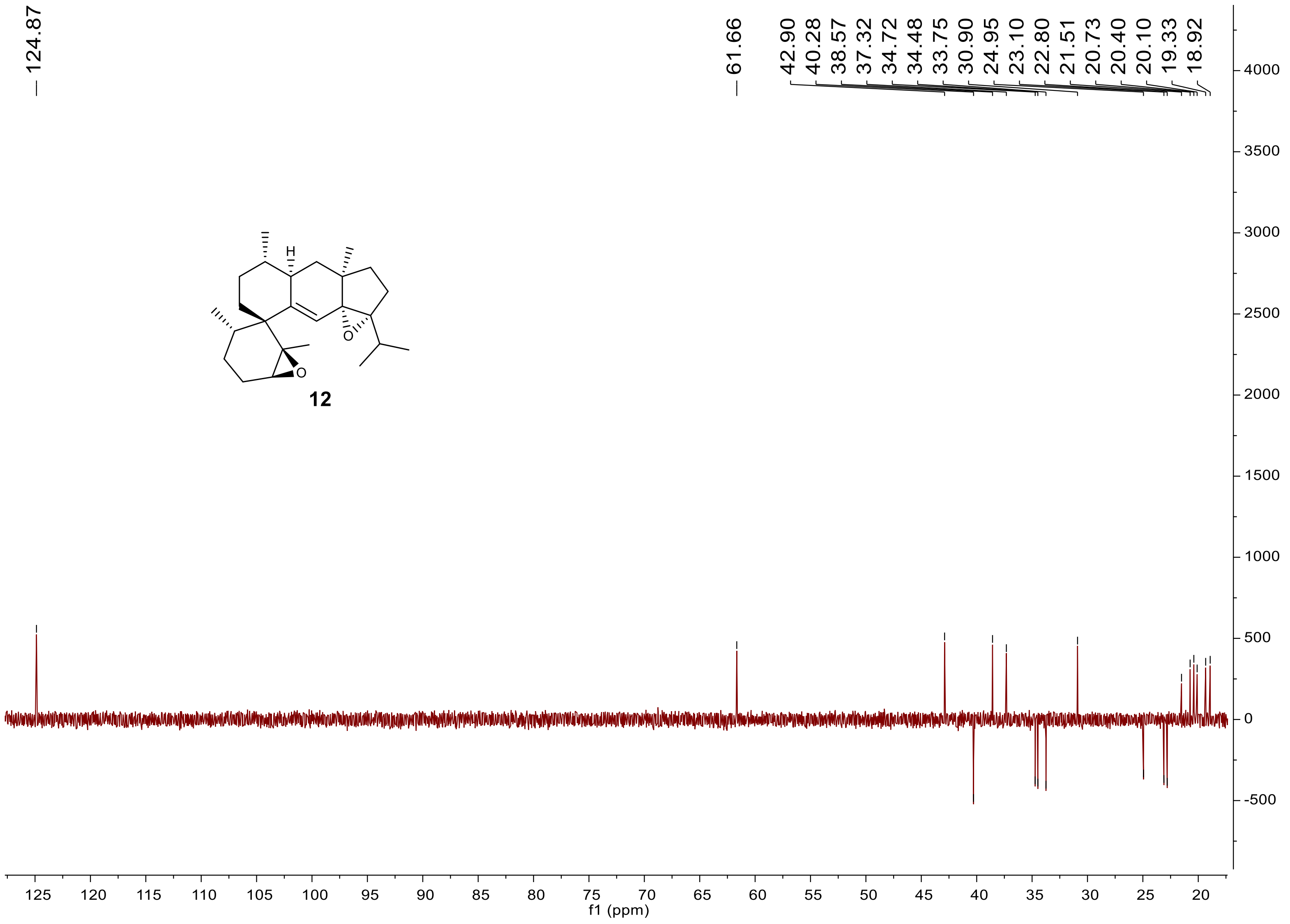


**Figure S110.** $^{13}$C-DEPT135 spectrum of **12** (176 MHz, $C_6D_6$).

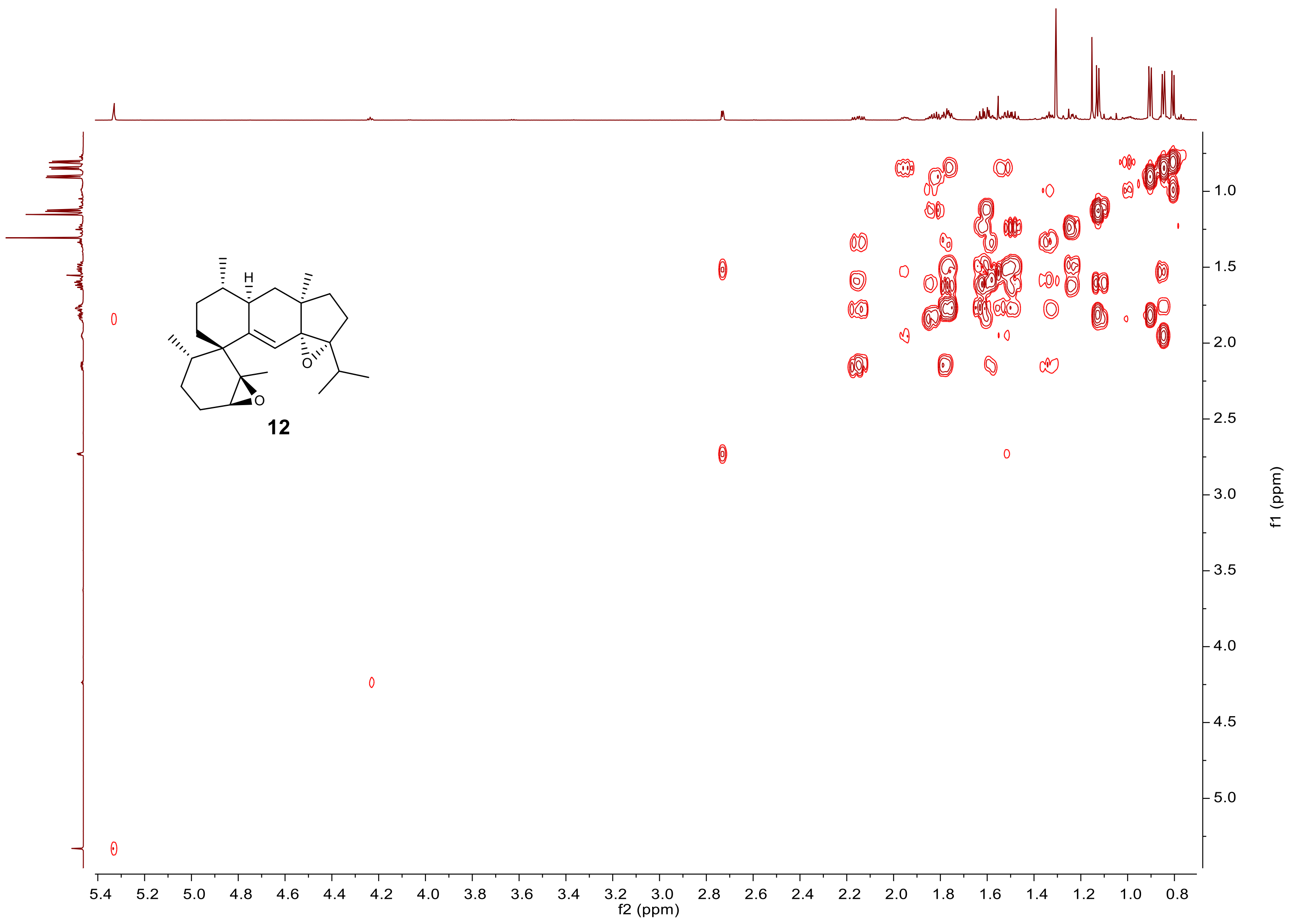


**Figure S111.** $^{1}H$-$^{1}H$-COSY spectrum ($C_6D_6$) of **12**.

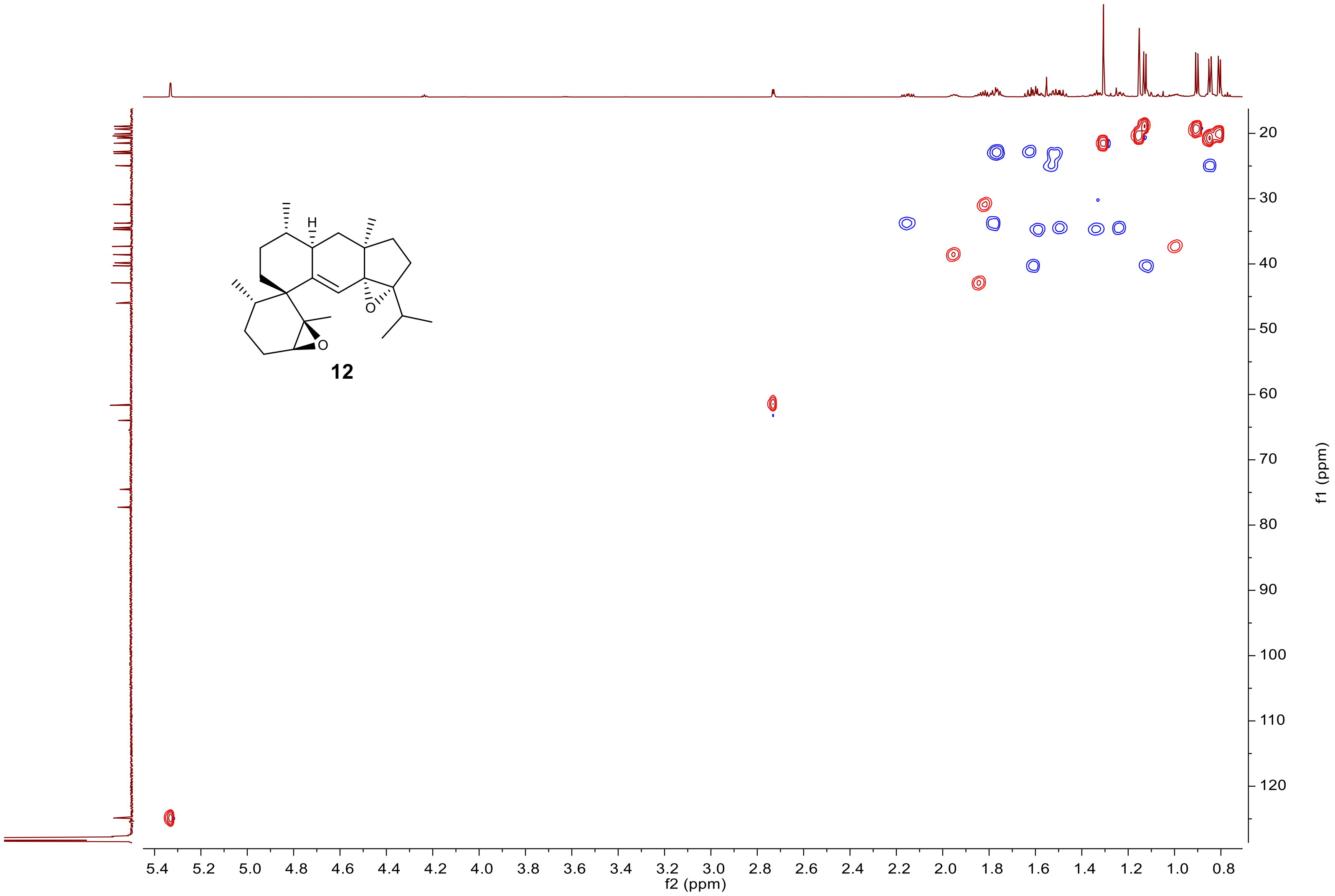


**Figure S112.** HSQC spectrum ($C_6D_6$) of **12**.

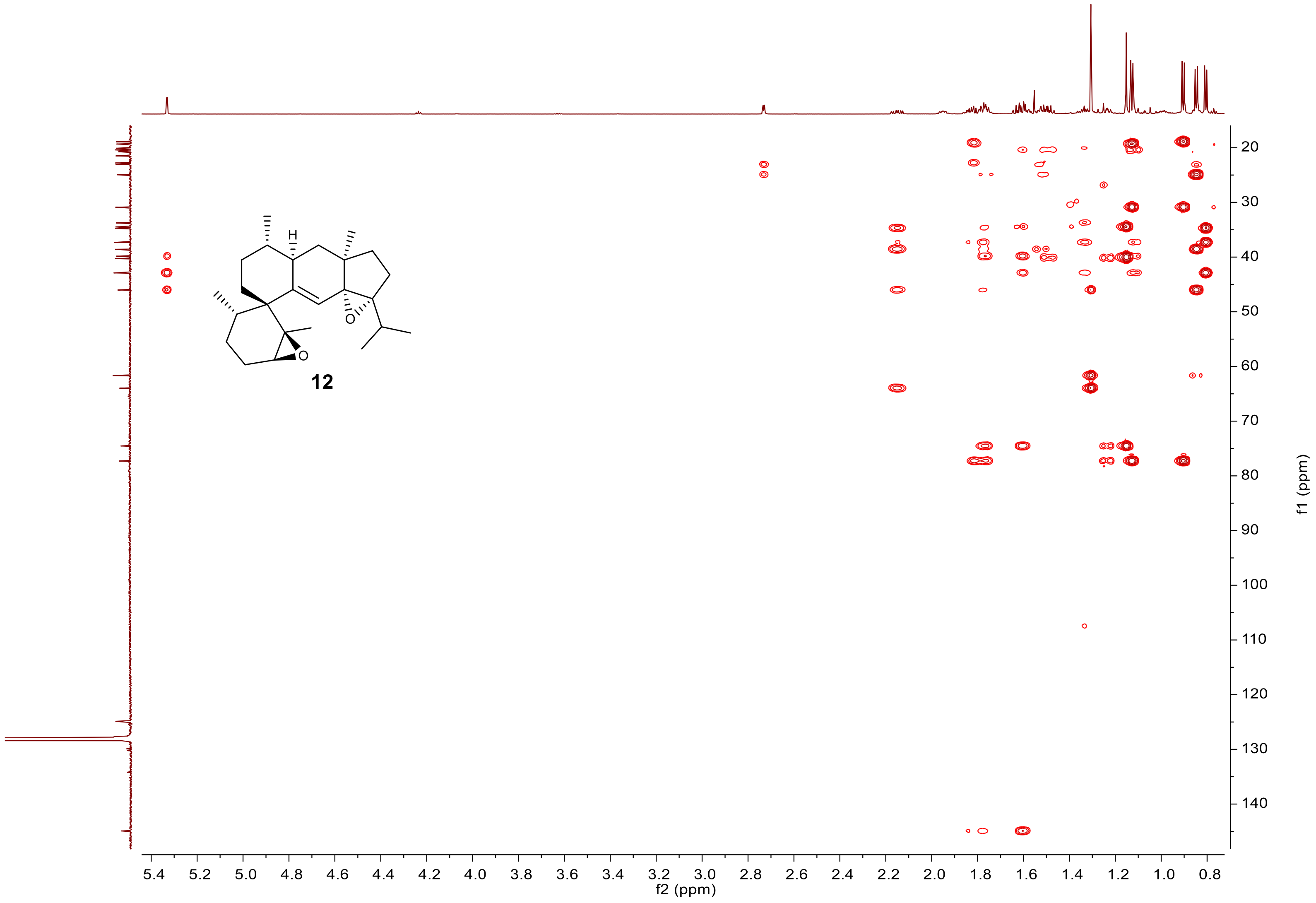


**Figure S113.** HMBC spectrum ($C_6D_6$) of **12**.

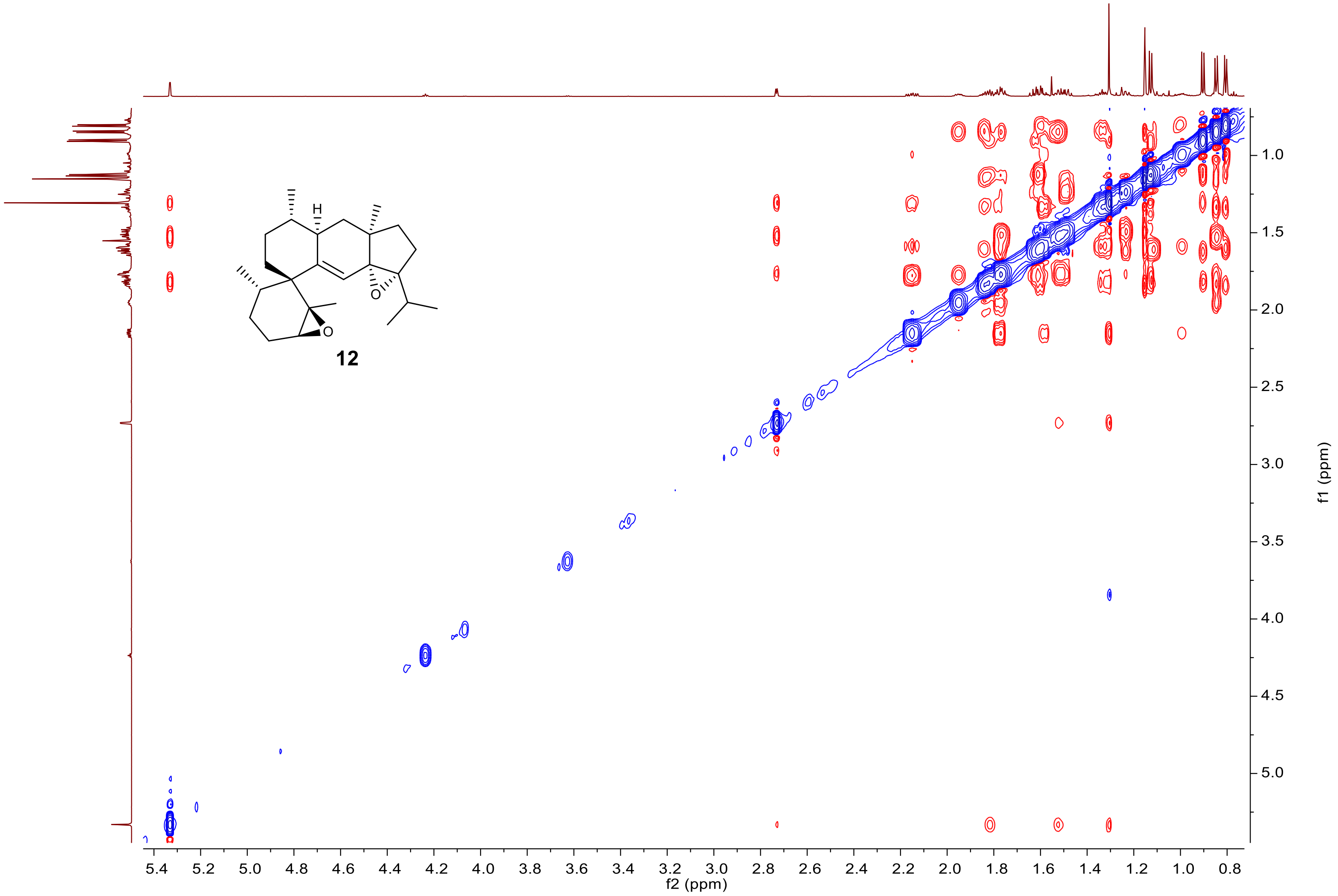


**Figure S114.** NOESY spectrum ($C_6D_6$) of **12**.

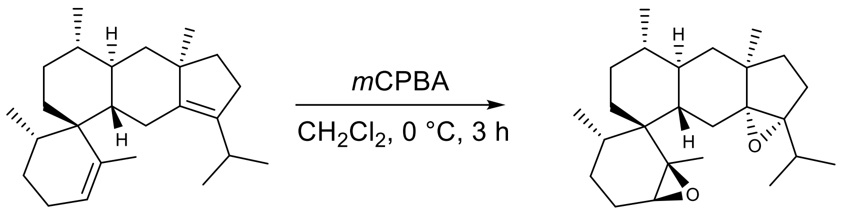


**Scheme S11.** Derivatisation of **4** with *meta*-chloroperoxybenzoic acid (*m*CPBA).

**Derivatisation of 4 with *m*CPBA**
Compound **4** (0.6 mg, 1.8 µmol, 1.0 eq.) was dissolved in dry $CH_2Cl_2$ (1.0 mL). After cooling to 0 °C, *meta*-chloroperoxybenzoic acid (*m*CPBA, 1.2 mg, 7.0 µmol, 3.9 eq.) was added and the reaction mixture was stirred at 0 °C for 3 h. The reaction was quenched by the addition of $H_2O$ (1.0 mL). The mixture was extracted with $CH_2Cl_2$ (3 x 5 mL). The combined organic layers were washed with sat. NaCl (5 mL), dried over $MgSO_4$, and concentrated under reduced pressure. Purification by column chromatography on silica gel [petroether/ethyl acetate (5:1)] yielded bis-epoxide **13** (0.4 mg, 1.1 µmol, 61%) as colourless oil.

**Sesterviolene epoxide E (13).** TLC [petroether/ethyl acetate (5:1)]: $R_f$ = 0.6. IR (diamond ATR): ṽ = 2958 (s), 2926 (s), 2869 (m), 1797 (w), 1772 (w), 1729 (w), 1676 (w), 1458 (m), 1376 (w), 1260 (m), 1215 (w), 1092 (s), 1017 (s), 969 (w), 910 (w), 863 (w), 799 (s), 734 (w), 699 (w), 606 (w), 500 (w) $cm^{-1}$. HR-MS (Q-TOF, 70 eV): *m*/*z* = 373.3097 (calc. for $[C_{25}H_{41}O_2]^+$ 373.3101). Optical rotary power: $[\alpha]_D^{20}$ = +22.5 (*c* 0.04, $CH_2Cl_2$). NMR data are given in Table S18.

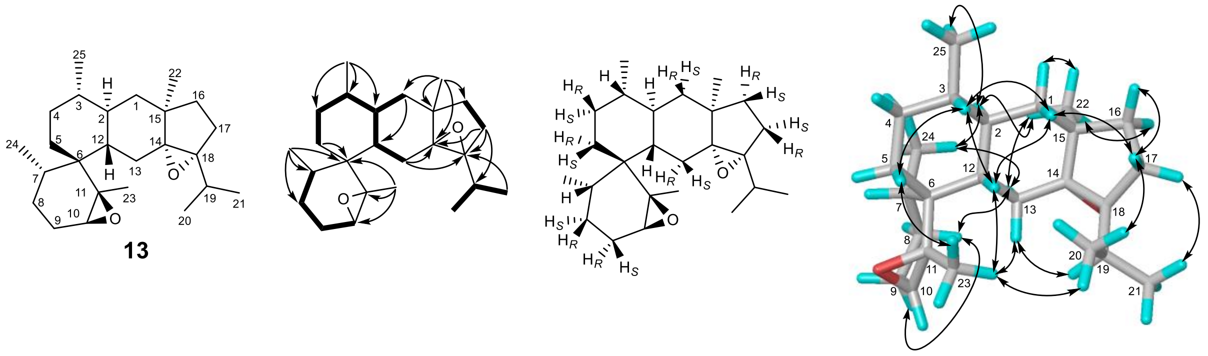

**Figure S115.** Structure elucidation of **13**. Bold: $^{1}H,^{1}H$-COSY, single headed arrows: key HMBC, and double headed arrows: NOESY correlations. Carbon numbering follows GFPP numbering to indicate the origin of each carbon. Diastereotopic hydrogens are labelled $H_R$ (*pro-R*) and $H_S$ (*pro-S*).

**Table S18.** NMR data of sesterviolene epoxide E (**13**) in $C_6D_6$ recorded at 298 K.

| C[a] | type | $^{1}H$[b] | $^{13}C$[b] |
|---|---|---|---|
| 1 | $CH_2$ | 1.66 (dd, *J* = 12.9, 3.6, $H_S$)<br>0.61 (dd, *J* = 12.9, 11.4, $H_R$) | 42.80 |
| 2 | CH | 1.62 (m) | 38.49 |
| 3 | CH | 0.80 (m) | 38.24 |
| 4 | $CH_2$ | 1.47 (m, 2H) | 32.74 |
| 5 | $CH_2$ | 1.87 (ddd, *J* = 14.4, 4.3, 2.8, $H_R$)<br>1.79 (m, $H_S$) | 36.47 |
| 6 | $C_q$ | – | 41.66 |
| 7 | CH | 1.81 (m) | 36.59 |
| 8 | $CH_2$ | 1.33 (m, $H_S$)<br>1.10 (m, $H_R$) | 29.51 |
| 9 | $CH_2$ | 1.72 (m, $H_S$)<br>1.59 (m, $H_R$) | 23.13 |
| 10 | CH | 2.75 (d, *J* = 4.5) | 61.38 |
| 11 | $C_q$ | – | 63.39 |
| 12 | CH | 1.39 (m) | 48.47 |
| 13 | $CH_2$ | 2.29 (t, *J* = 13.6, $H_R$)<br>1.52 (dd, *J* = 13.6, 3.7, $H_S$) | 27.28 |
| 14 | $C_q$ | – | 75.79 |
| 15 | $C_q$ | – | 41.59 |
| 16 | $CH_2$ | 1.41 (m, $H_R$)<br>1.12 (m, $H_S$) | 34.30 |
| 17 | $CH_2$ | 1.73 (m, $H_S$)<br>1.56 (m, $H_R$) | 22.42 |
| 18 | $C_q$ | – | 75.60 |
| 19 | CH | 1.76 (m) | 29.06 |
| 20 | $CH_3$ | 0.93 (d, *J* = 6.9) | 20.35 |
| 21 | $CH_3$ | 1.16 (d, *J* = 6.5) | 19.12 |
| 22 | $CH_3$ | 1.15 (s) | 20.70 |
| 23 | $CH_3$ | 1.26 (s) | 21.21 |
| 24 | $CH_3$ | 1.16 (d, *J* = 7.5) | 23.20 |
| 25 | $CH_3$ | 0.86 (d, *J* = 6.4) | 20.48 |

[a] Carbon numbering as shown in Figure S115. [b] Chemical shifts $\delta$ in ppm, multiplicity: s = singlet, d = doublet, t = triplet, m = multiplet, coupling constants *J* are given in Hertz.

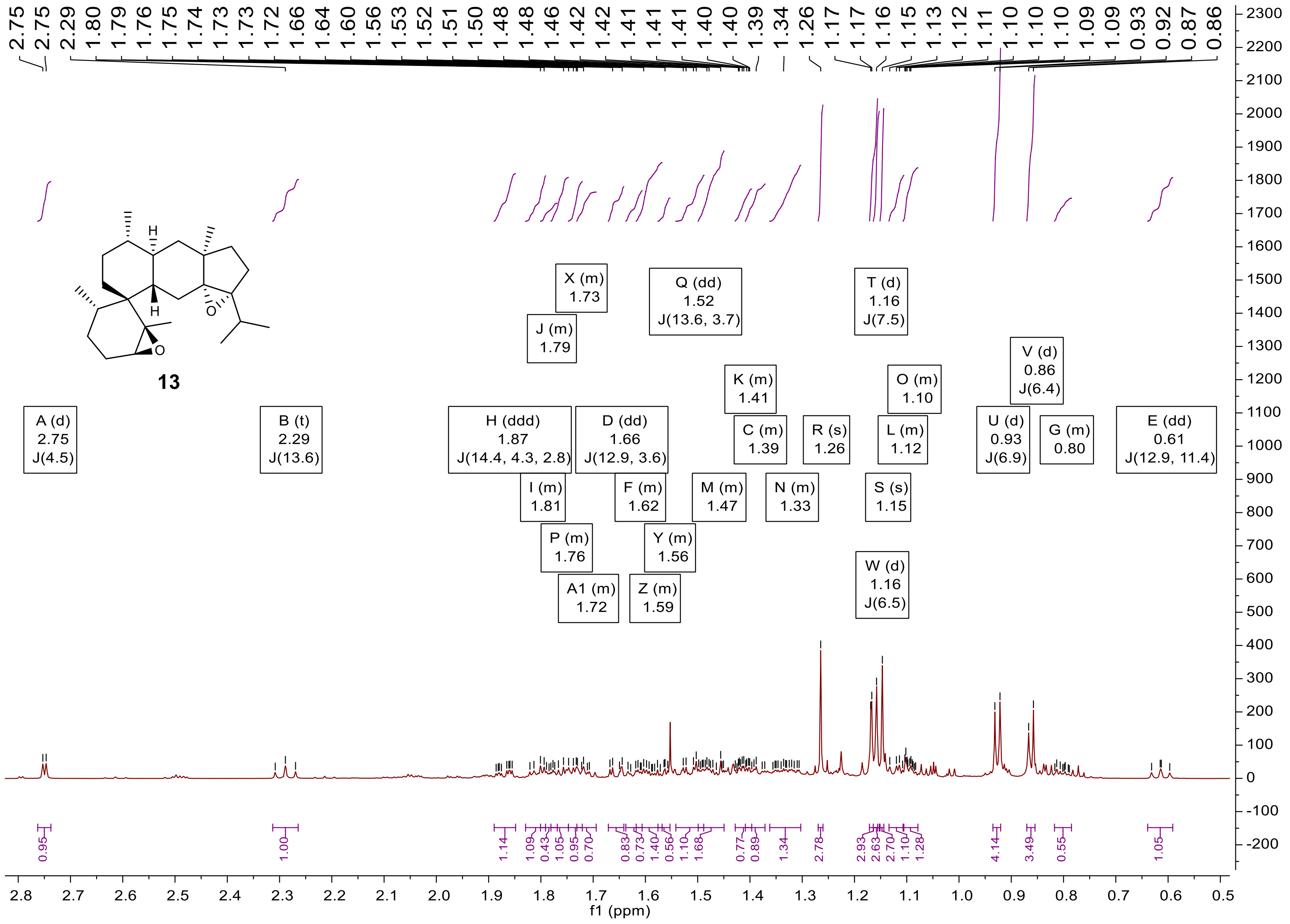

**Figure S116.** $^{1}$H-NMR spectrum of **13** (700 MHz, $C_6D_6$).

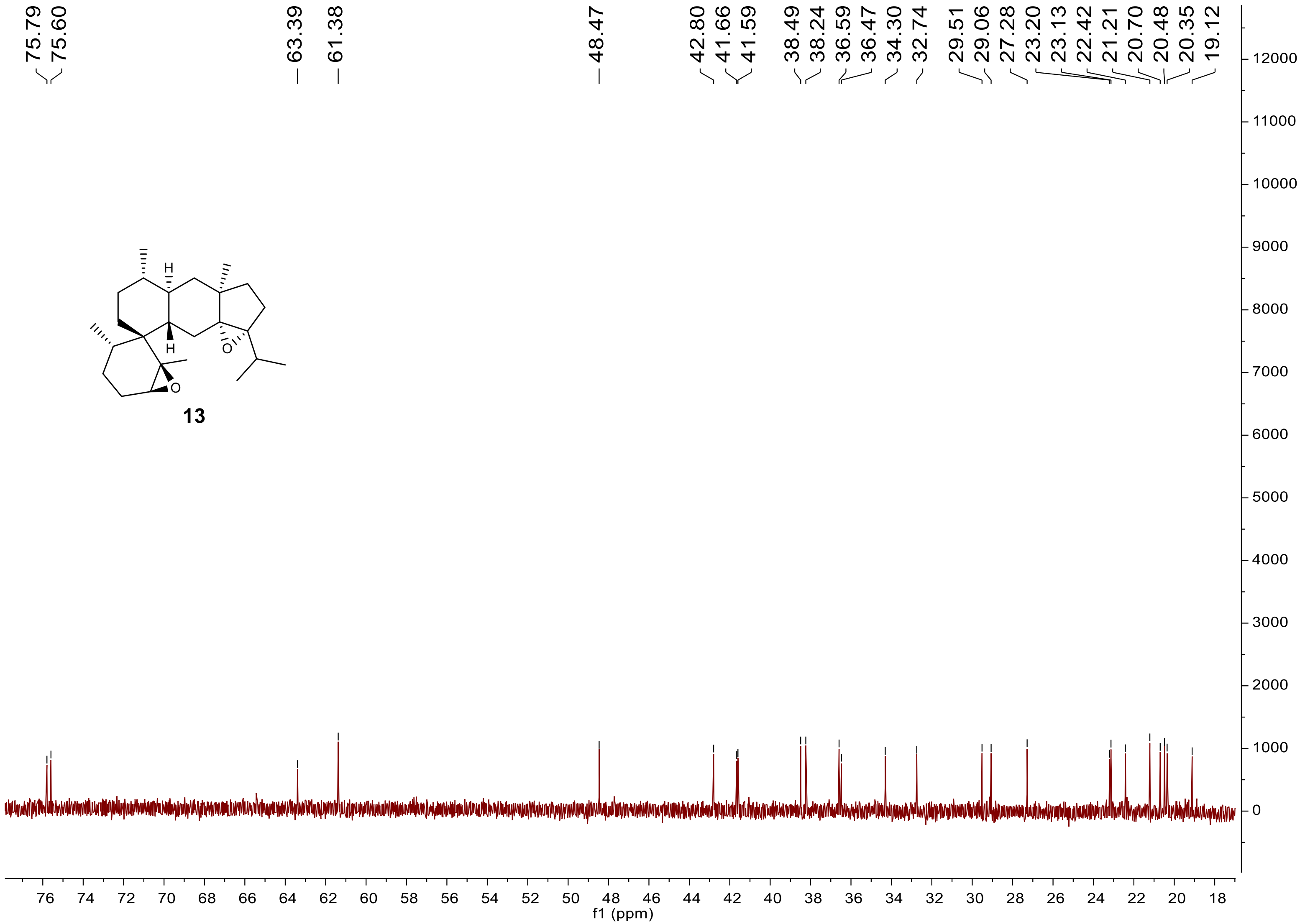


**Figure S117.** $^{13}$C-NMR spectrum of **13** (176 MHz, $C_6D_6$).

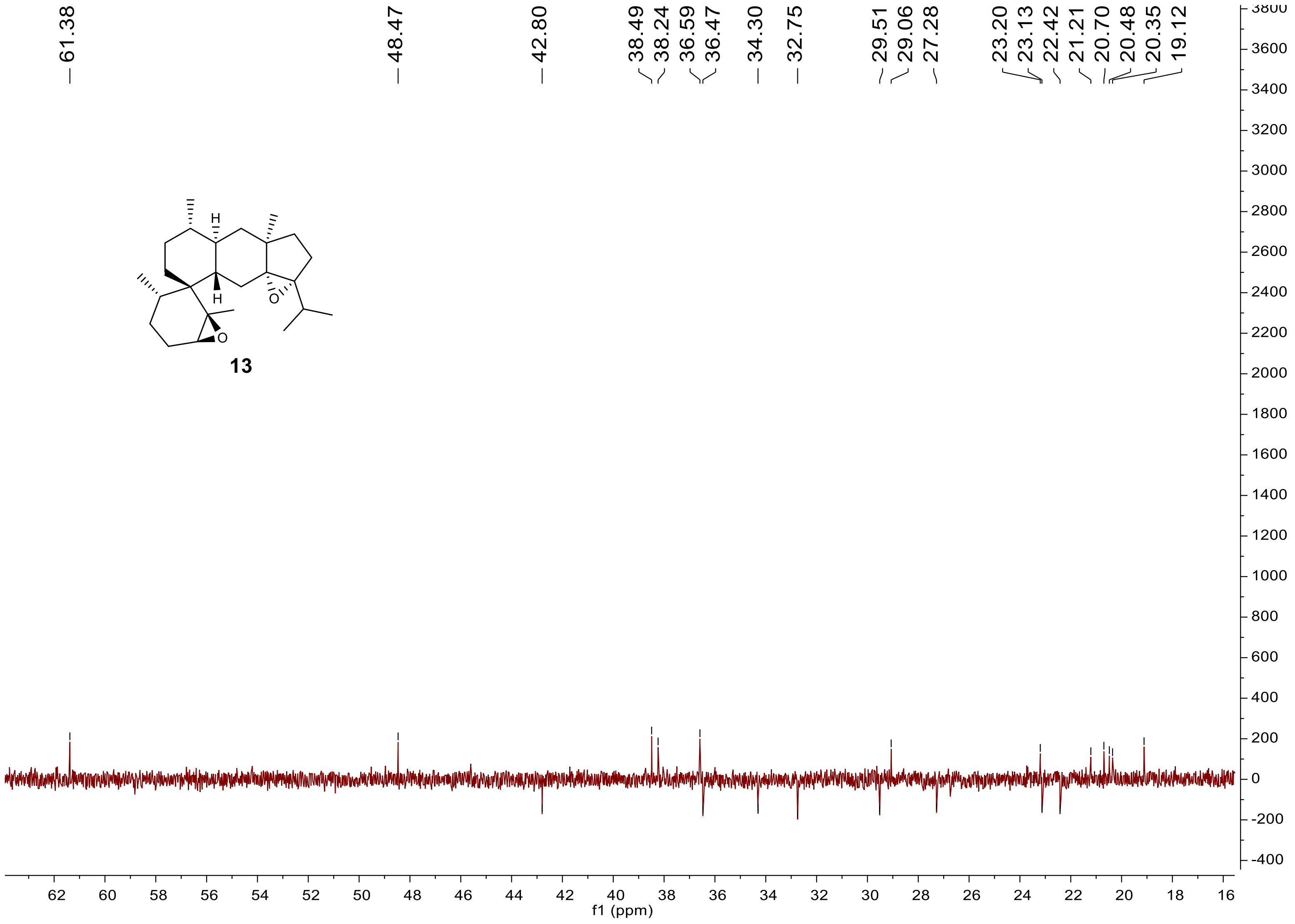


**Figure S118.** $^{13}$C-DEPT135 spectrum of **13** (176 MHz, $C_6D_6$).

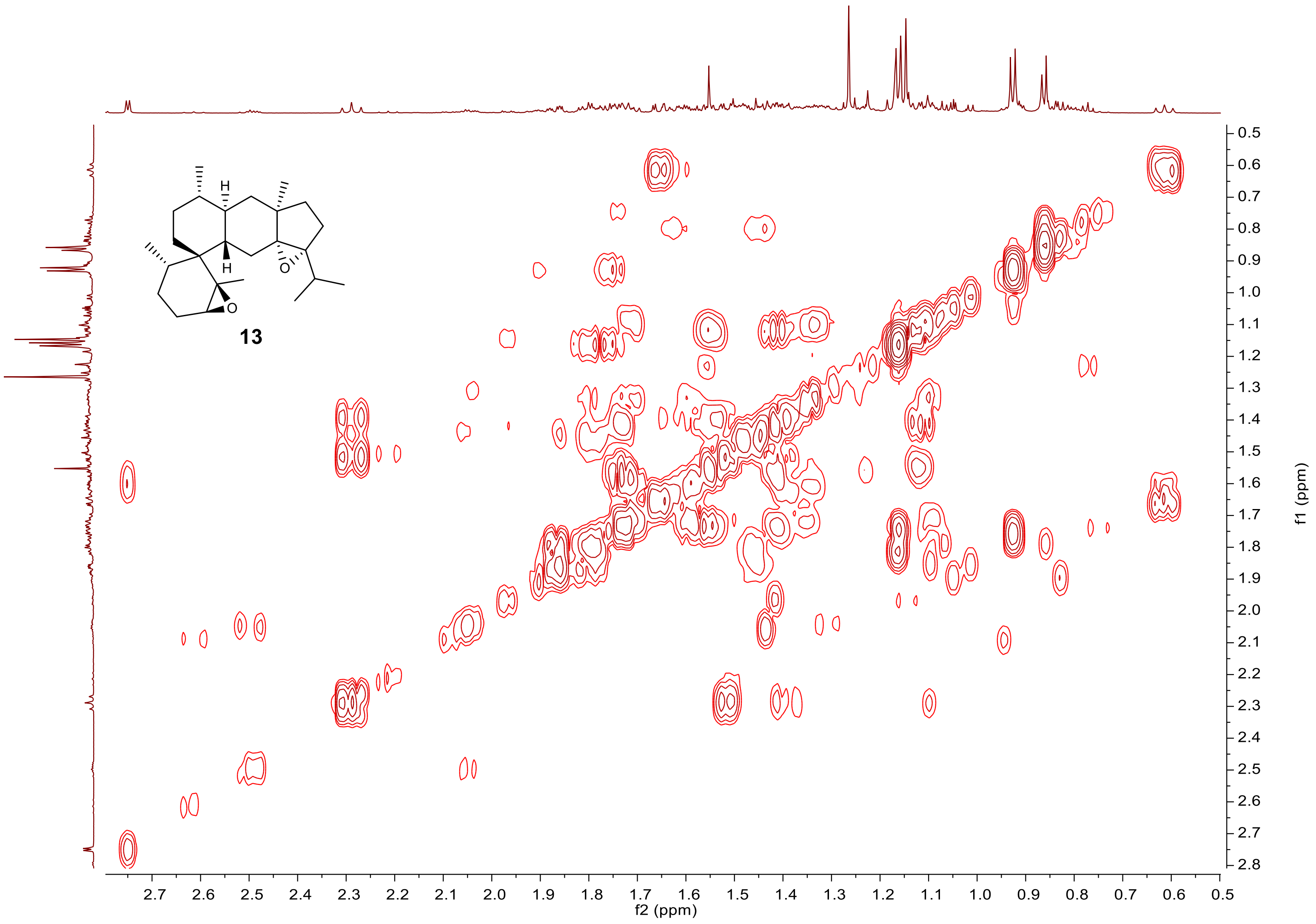


**Figure S119.** $^{1}H$-$^{1}H$-COSY spectrum ($C_6D_6$) of **13**.

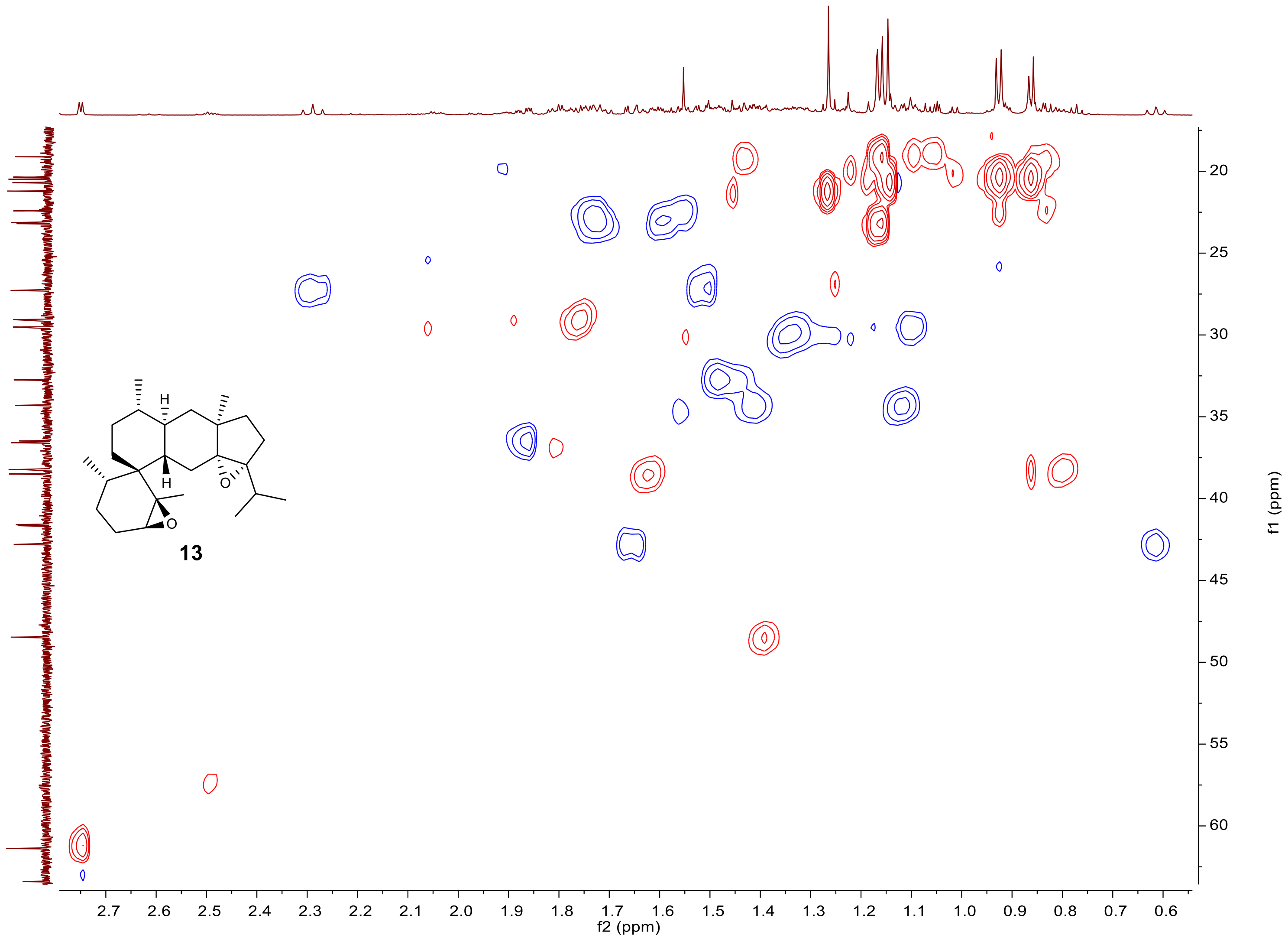


**Figure S120.** HSQC spectrum ($C_6D_6$) of **13**.

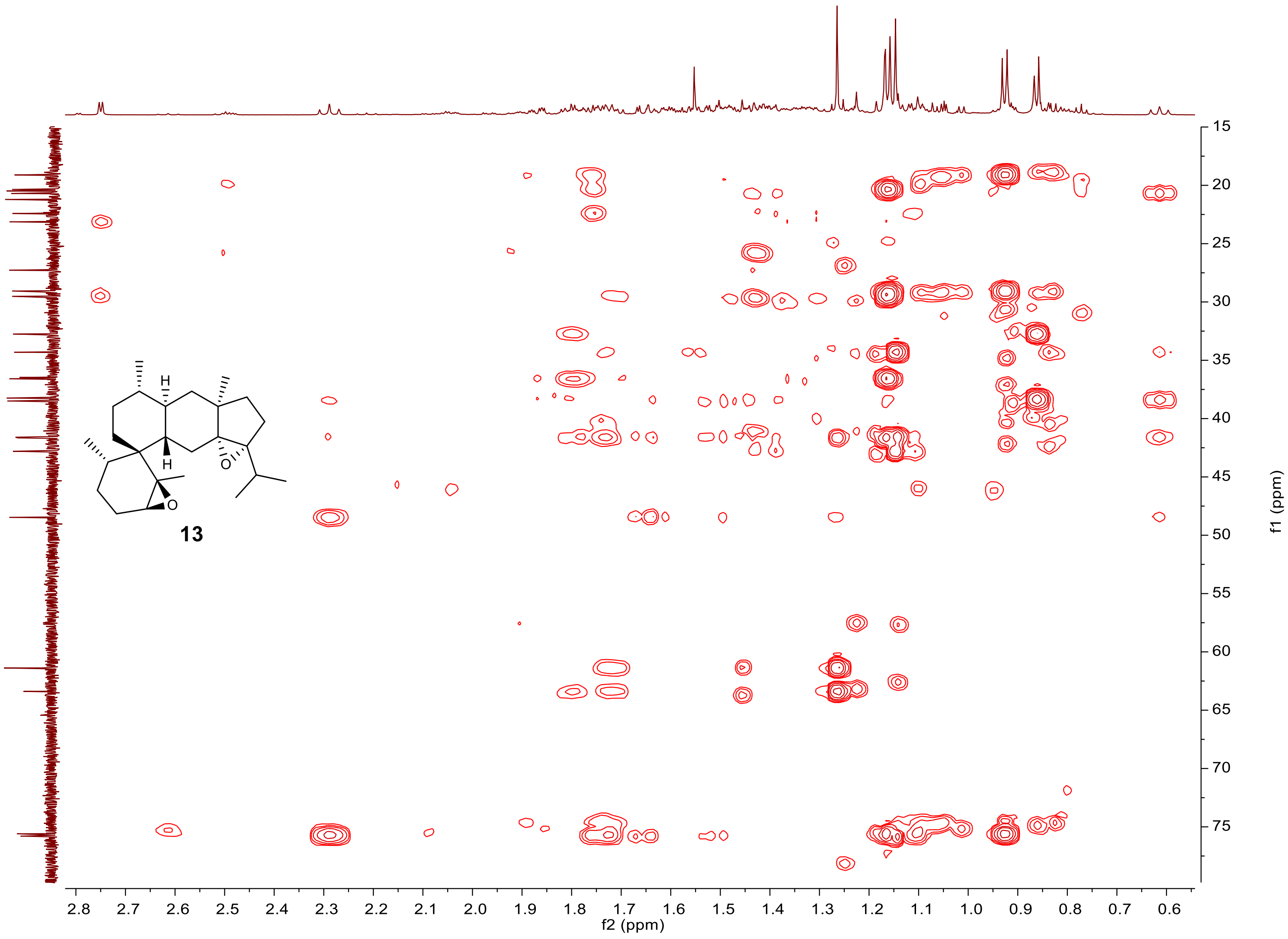


**Figure S121.** HMBC spectrum ($C_6D_6$) of **13**.

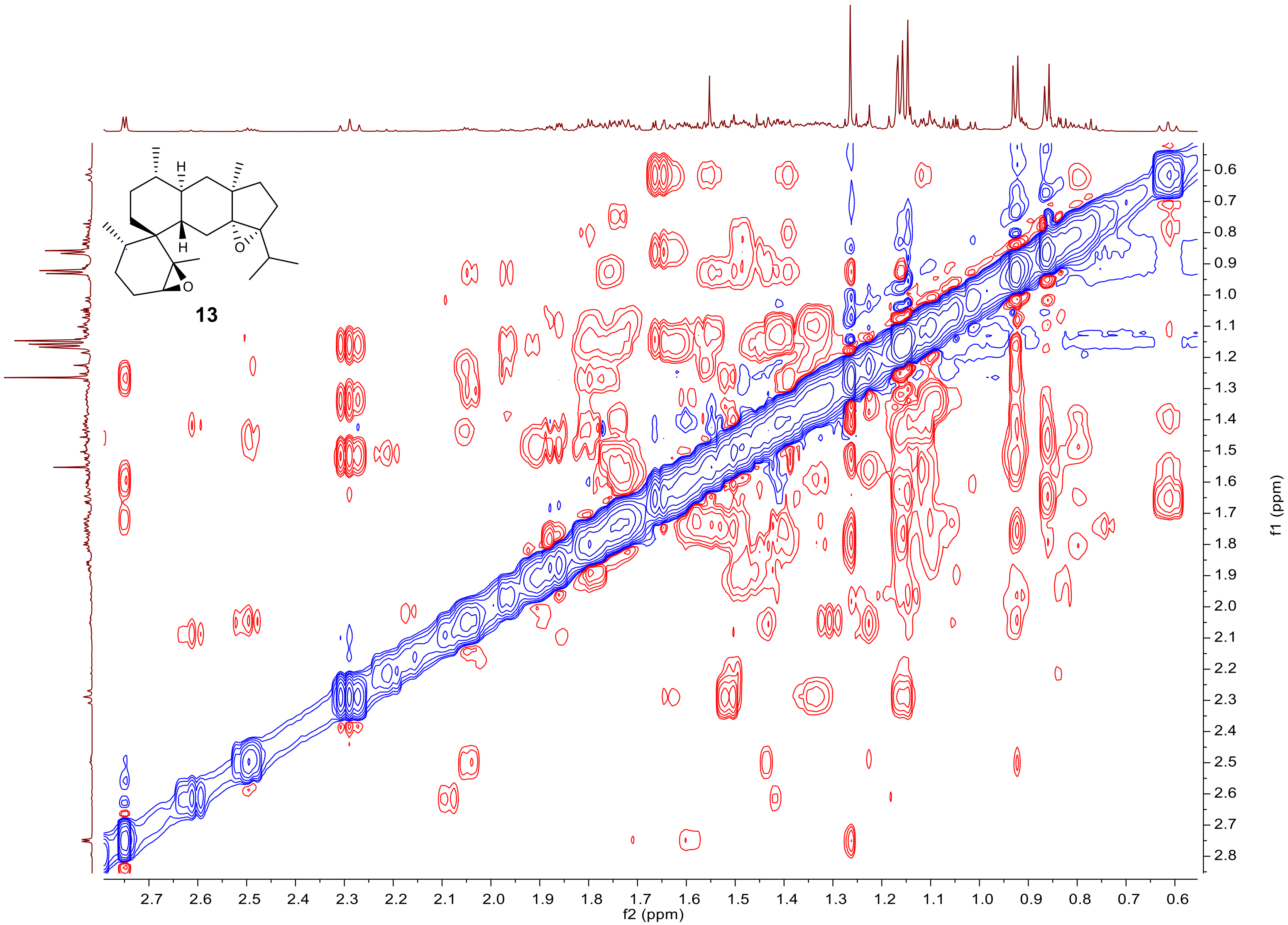


**Figure S122.** NOESY spectrum ($C_6D_6$) of **13**.

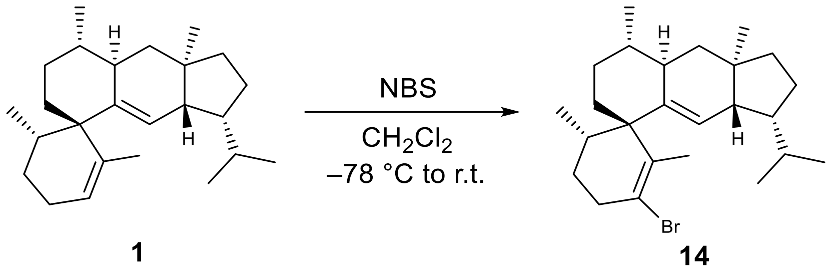

**Scheme S12.** Derivatisation of **1** with *N*-bromosuccinimide (NBS).

**Derivatisation of 1 with NBS**

*N*-Bromosuccinimide (NBS, 3.2 mg, 18 μmol, 1.0 eq.) was added in one portion to a stirred solution of **1** (6.0 mg, 18 μmol, 1.0 eq.) in dry $CH_2Cl_2$ (2 mL) at –78 °C. After stirring for 10 min at –78 °C, the reaction was allowed to warm to room temperature and stirring was continued for 2 h. Another portion of NBS (1.0 eq.) was added and the reaction was further stirred at room temperature for 20 min, before saturated $NH_4HCl$ solution (4 mL) was added. The mixture was extracted with $Et_2O$ (3 x 10 mL). The combined organic layers were washed with sat. NaCl (5 mL), dried over $MgSO_4$, and concentrated under reduced pressure. Purification by column chromatography on silica gel [100% *n*-hexane] yielded 10-bromosesterviolene A (**14**, 0.8 mg, 1.9 μmol, 11%) as a colourless oil.

**10-Bromosesterviolene A (14).** TLC [100% *n*-hexane]: $R_f$ = 0.7. GC (HP5-MS): *I* = 2751. MS (EI, 70 eV): *m*/*z* (%) = 420 (77), 418 (79), 405 (8), 403 (9), 377 (34), 375 (37), 339 (63), 323 (6), 297 (8), 295 (11), 283 (9), 281 (19), 255 (11), 253 (12), 239 (10), 229 (13), 215 (38), 213 (41), 207 (33), 203 (58), 187 (36), 175 (81), 173 (45), 161 (28), 159 (37), 155 (22), 145 (31), 143 (30), 133 (73), 119 (57), 107 (84), 105 (68), 95 (34), 93 (49), 91 (66), 81 (30), 79 (36), 77 (23), 69 (33), 67 (22), 55 (63), 43 (100), 41 (72). IR (diamond ATR): ṽ = 2953 (s), 2924 (s), 2856 (s), 1729 (m), 1684 (w), 1643 (w), 1601 (w), 1461 (m), 1376 (w), 1260 (s), 1089 (s), 1074 (s), 1018 (s), 963 (w), 866 (w), 848 (w), 799 (s), 742 (w), 702 (w), 668 (w), 615 (w) $cm^{-1}$. HR-MS (Q-TOF, 70 eV): calc. $[C_{25}H_{39}Br]^{+\cdot}$ *m*/*z* = 418.2230; found: *m*/*z* = 418.2232. Optical rotary power: $[\alpha]_D^{20}$ = +67.5 (*c* 0.08, $CH_2Cl_2$). NMR data are given in Table S19.

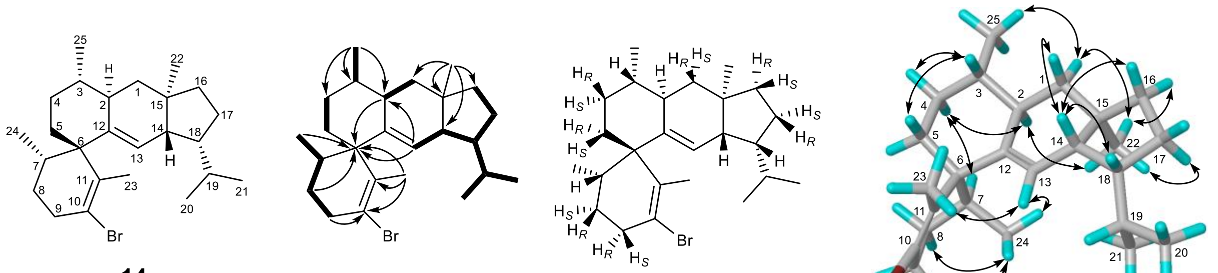

**14**

**Figure S123.** Structure elucidation of **14**. Bold: $^{1}H,^{1}H$-COSY, single headed arrows: key HMBC, and double headed arrows: NOESY correlations. Carbon numbering follows GFPP numbering to indicate the origin of each carbon. Diastereotopic hydrogens are labelled $H_R$ (*pro-R*) and $H_S$ (*pro-S*).

**Table S19.** NMR data of 10-bromosesterviolene A (**14**) in $C_6D_6$ recorded at 298 K.

| C[a] | type | $^{1}H$[b] | $^{13}C$[b] |
|---|---|---|---|
| 1 | $CH_2$ | 1.94 (dd, *J* = 12.2, 6.1, $H_S$)<br>0.93 (t, *J* = 11.6, $H_R$) | 45.54 |
| 2 | CH | 1.57 (m) | 41.10 |
| 3 | CH | 1.01 (m) | 39.13 |
| 4 | $CH_2$ | 1.32 (dq, *J* = 13.3, 3.6, $H_R$)<br>1.15 (m, $H_S$) | 31.77 |
| 5 | $CH_2$ | 1.58 (m, $H_S$)<br>1.52 (dt, *J* = 13.9, 3.5, $H_R$) | 34.57 |
| 6 | $C_q$ | – | 53.59 |
| 7 | CH | 1.91 (m) | 30.77 |
| 8 | $CH_2$ | 1.72 (m, $H_R$)<br>1.18 (m, $H_S$) | 27.74 |
| 9 | $CH_2$ | 2.66 (m, $H_R$)<br>2.37 (m, $H_S$) | 33.21 |
| 10 | $C_q$ | – | 122.90 |
| 11 | $C_q$ | – | 137.57 |
| 12 | $C_q$ | – | 137.27 |
| 13 | CH | 5.66 (t, *J* = 2.3) | 127.00 |
| 14 | CH | 2.23 (ddd, *J* = 9.5, 4.2, 2.2) | 51.00 |
| 15 | $C_q$ | – | 41.04 |
| 16 | $CH_2$ | 1.47 (dd, *J* = 11.4, 7.9, $H_R$)<br>1.09 (m, $H_S$) | 39.99 |
| 17 | $CH_2$ | 1.84 (dtd, *J* = 13.5, 9.2, 1.1, $H_R$)<br>1.59 (m, $H_S$) | 28.50 |
| 18 | CH | 1.72 (m) | 46.42 |
| 19 | CH | 1.60 (m) | 32.41 |
| 20/21 | $CH_3$ | 1.01 (d, *J* = 6.5) | 24.09 |
| 21/20 | $CH_3$ | 0.82 (d, *J* = 6.6) | 22.42 |
| 22 | $CH_3$ | 0.86 (s) | 20.68 |
| 23 | $CH_3$ | 1.97 (t, *J* = 1.9) | 23.55 |
| 24 | $CH_3$ | 0.89 (d, *J* = 7.0) | 17.28 |
| 25 | $CH_3$ | 0.84 (d, *J* = 6.4) | 20.25 |

[a] Carbon numbering as shown in Figure S123. [b] Chemical shifts δ in ppm, multiplicity: s = singlet, d = doublet, t = triplet, q = quartet, m = multiplet, coupling constants *J* are given in Hertz.

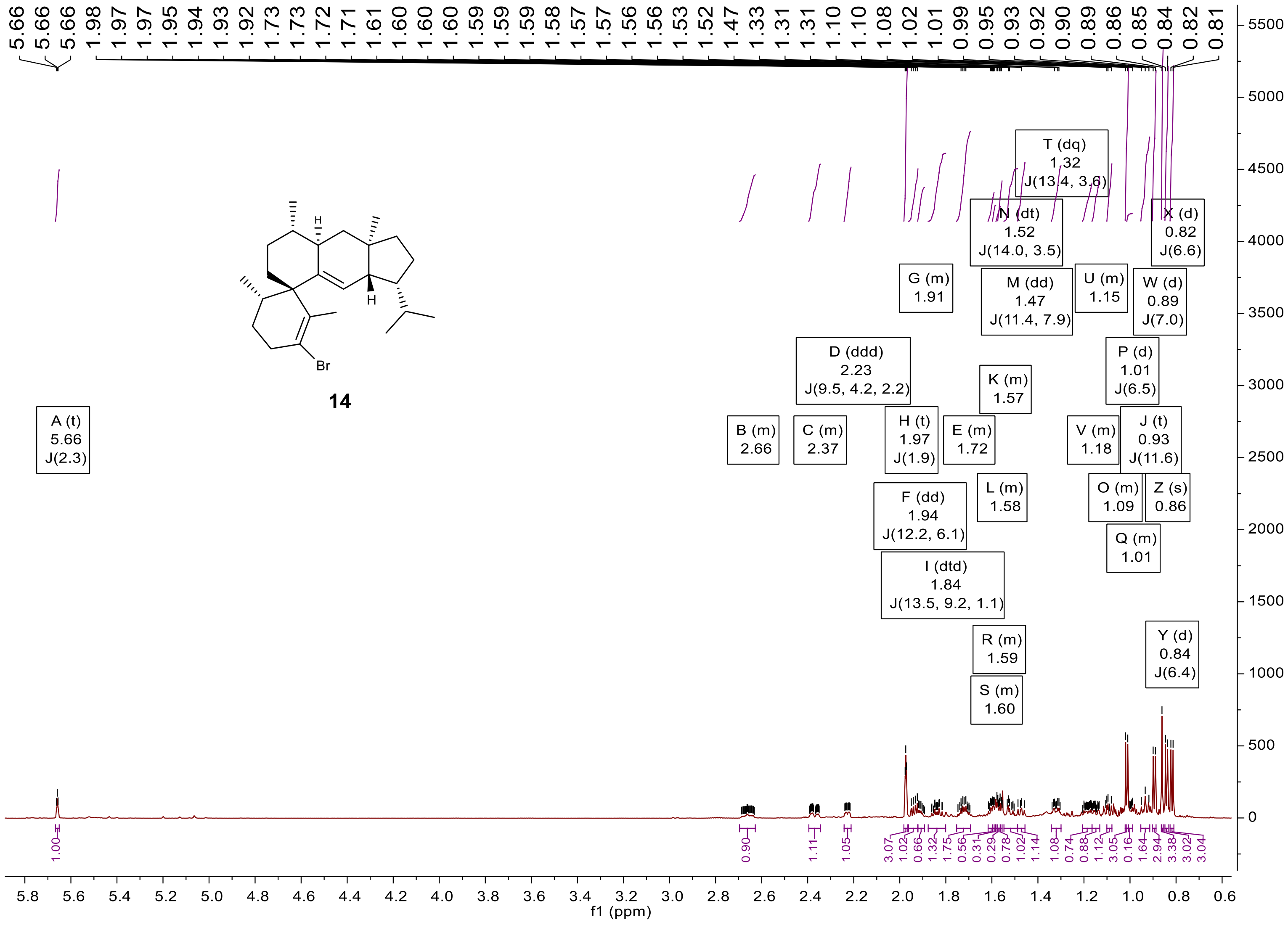

**Figure S124.** $^1$H-NMR spectrum of **14** (700 MHz, $C_6D_6$).

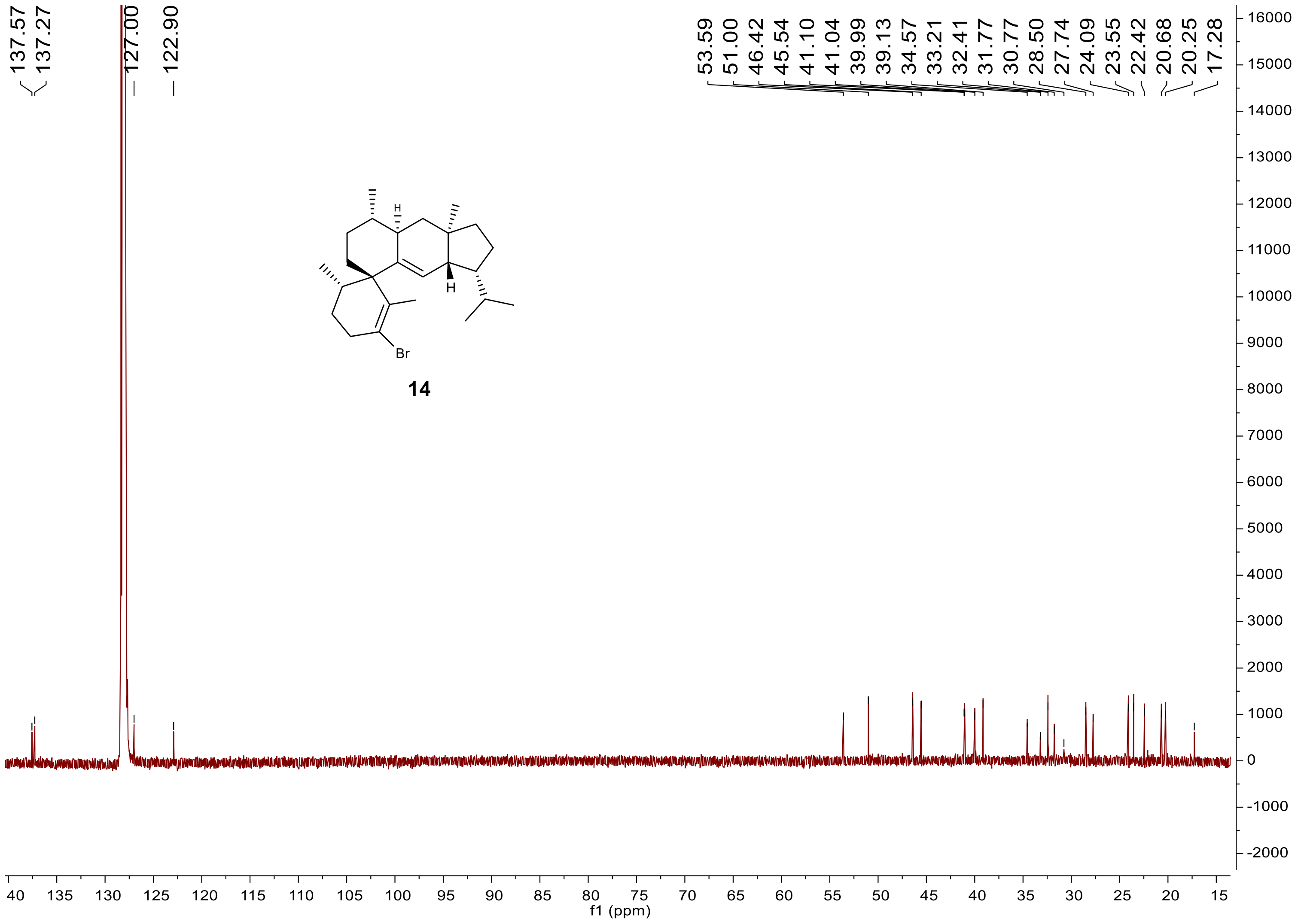


**Figure S125.** $^{13}C$-NMR spectrum of **14** (176 MHz, $C_6D_6$).

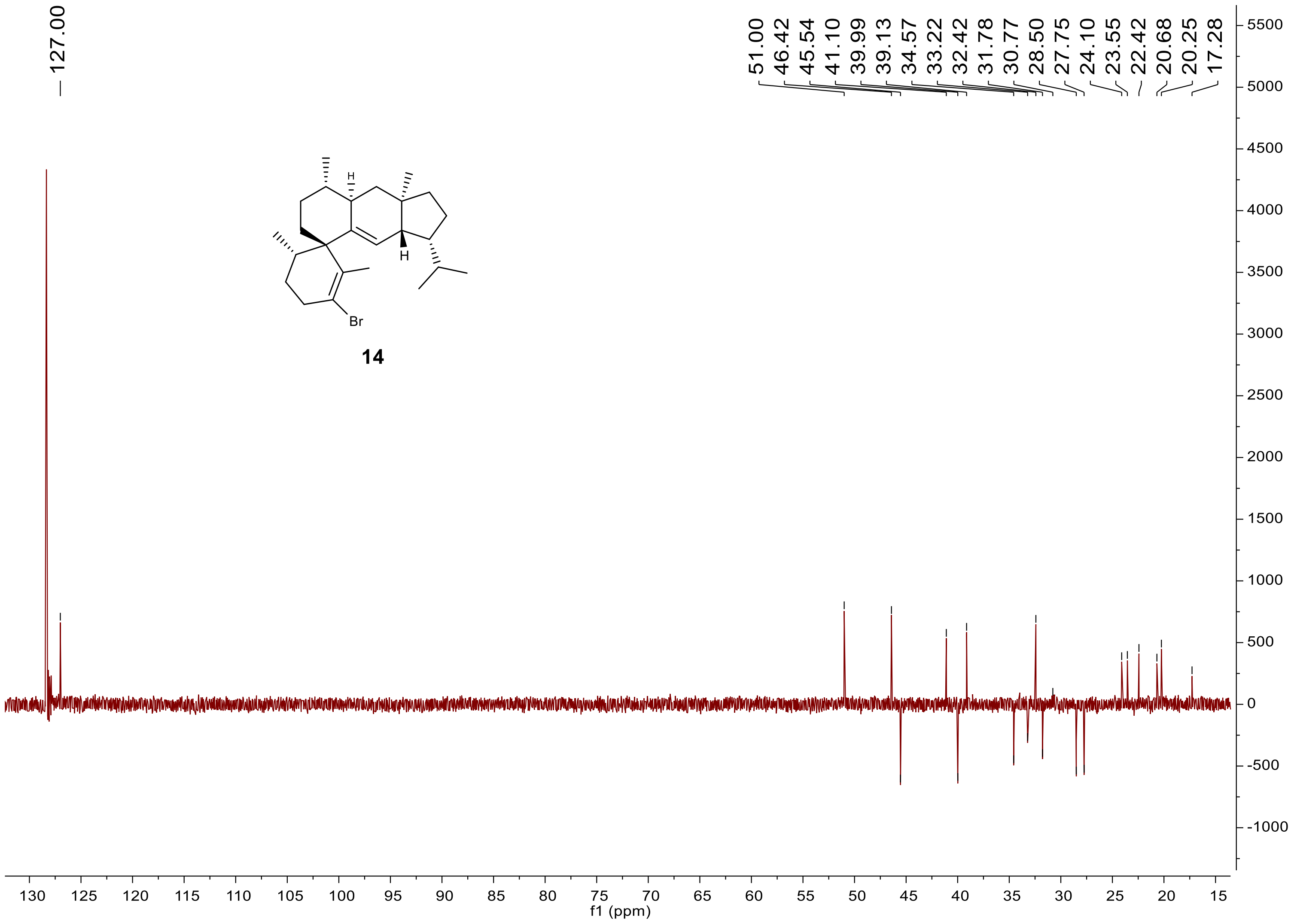


**Figure S126.** $^{13}$C-DEPT135 spectrum of **14** (176 MHz, $C_6D_6$).

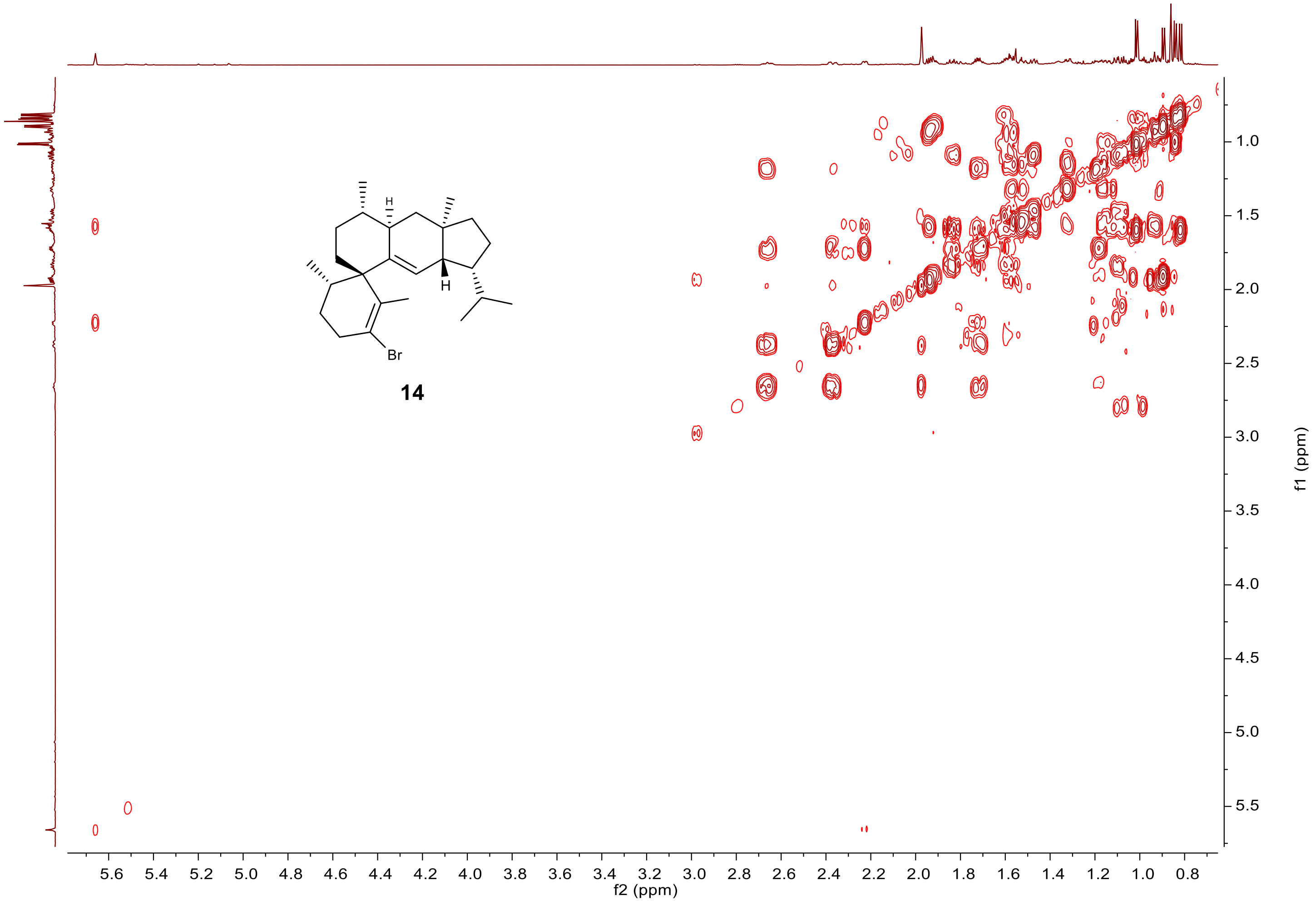


**Figure S127.** $^1$H-$^1$H-COSY spectrum ($C_6D_6$) of **14**.

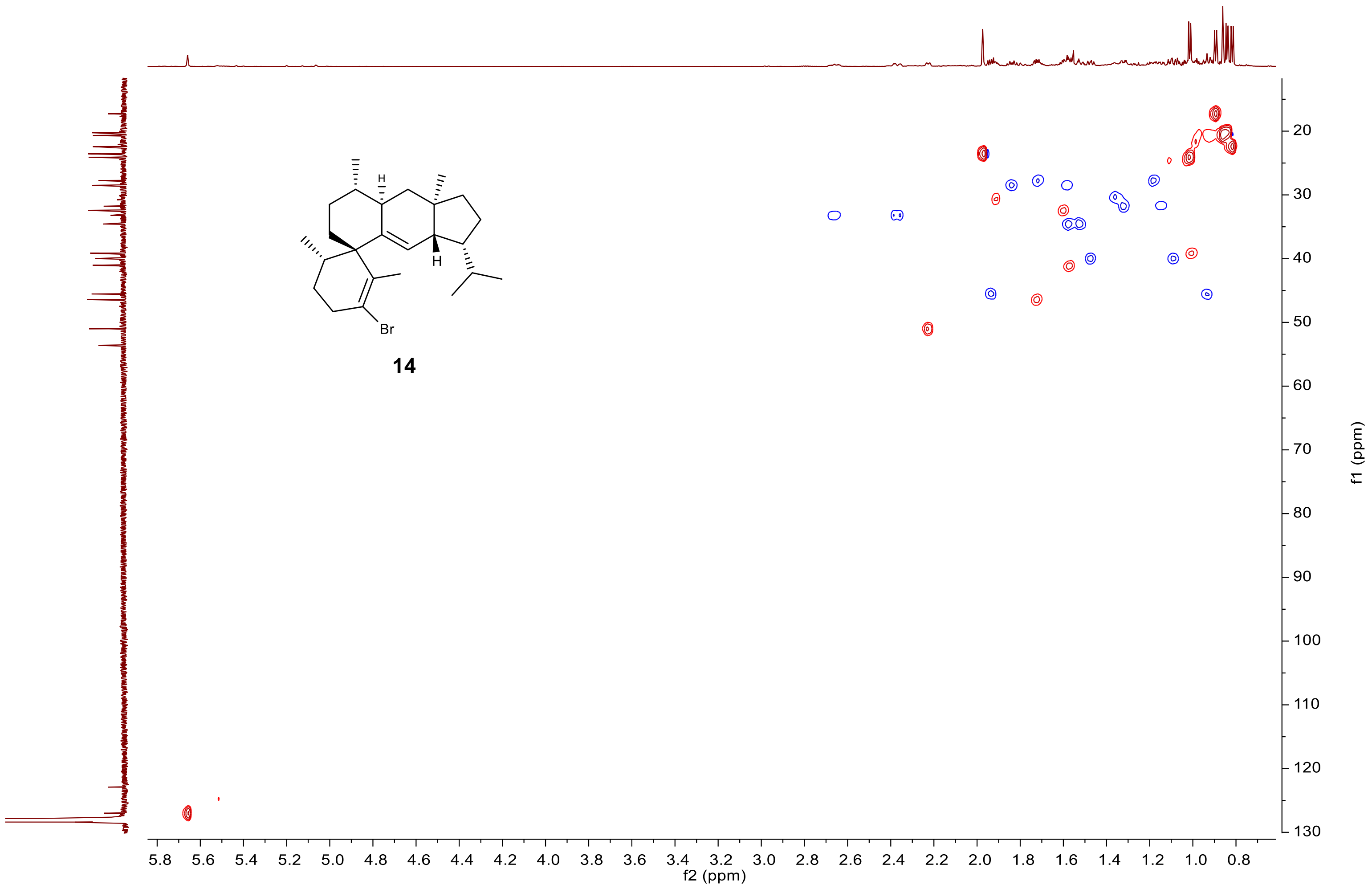


**Figure S128.** HSQC spectrum ($C_6D_6$) of **14**.

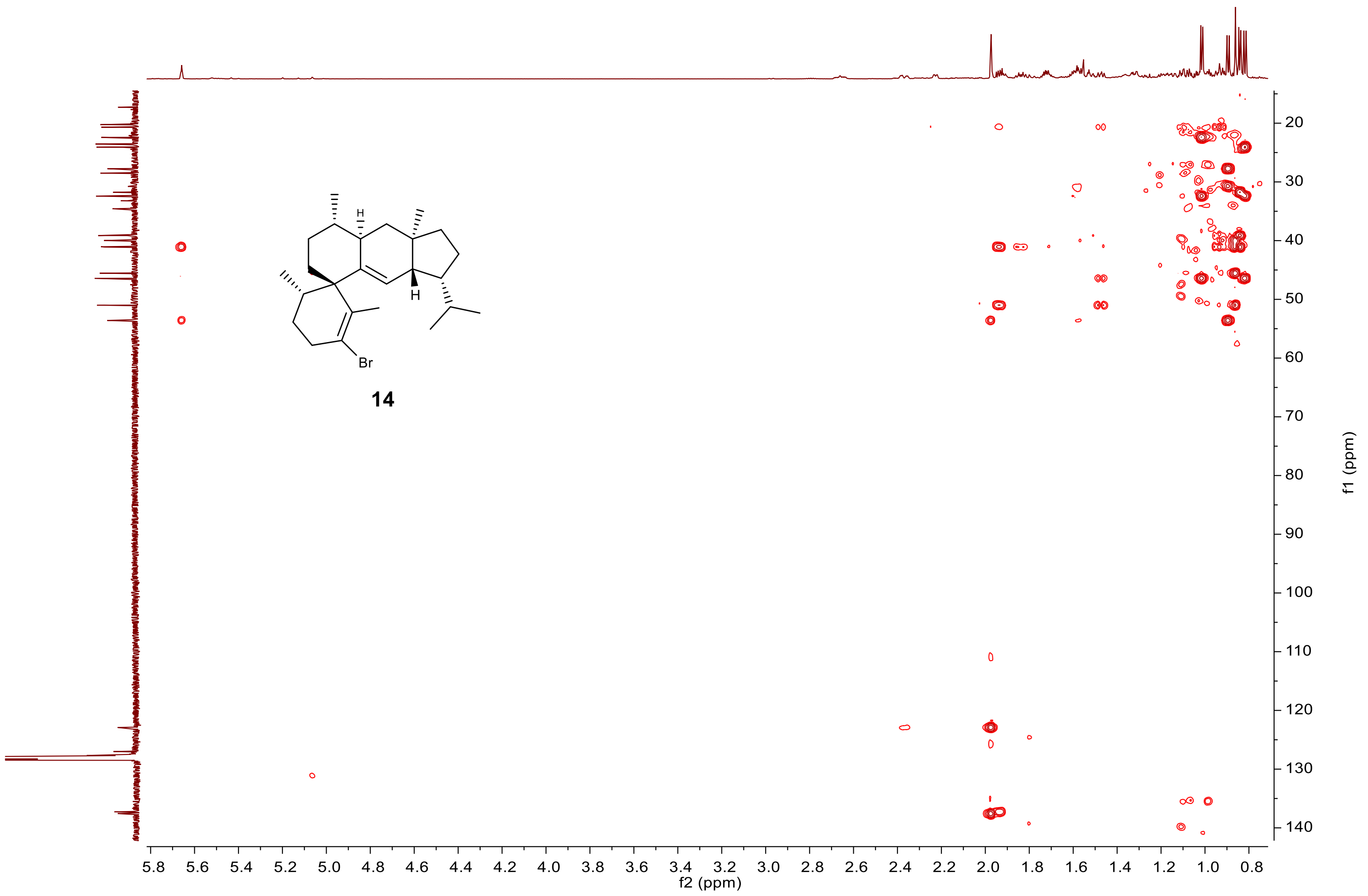


**Figure S129.** HMBC spectrum ($C_6D_6$) of **14**.

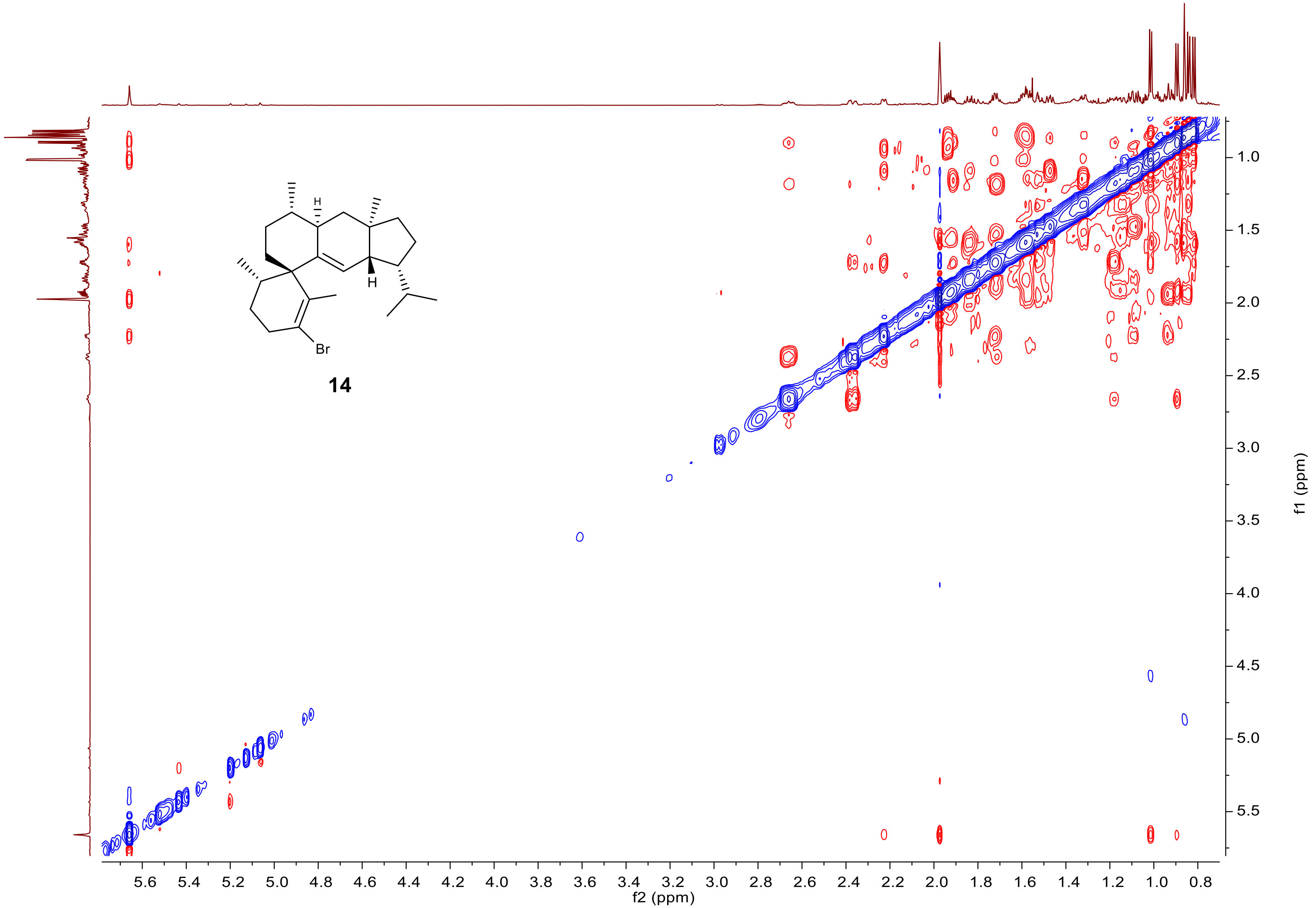


**Figure S130.** NOESY spectrum ($C_6D_6$) of **14**.

## References


[1] L. Lauterbach, B. Goldfuss, J. S. Dickschat, *Angew. Chem. Int. Ed.* **2020**, *59*, 11943.
[2] Y. Yamada, T. Kuzuyama, M. Komatsu, K. Shin-ya, S. Omura, D. E. Cane, H. Ikeda, *Proc. Natl. Acad. Sci. USA* **2015**, *112*, 857.
[3] C. Nakano, S. Horinouchi, Y. Ohnishi, *J. Biol. Chem.* **2011**, *286*, 27980.
[4] L. Lauterbach, J. S. Dickschat, *Org. Biomol. Chem.* **2020**, *18*, 4547.
[5] J. Rinkel, L. Lauterbach, P. Rabe, J. S. Dickschat, *Angew. Chem. Int. Ed.* **2018**, *57*, 3238.
[6] P. Rabe, J. Rinkel, T. A. Klapschinski, L. Barra, J. S. Dickschat, *Org. Biomol. Chem.* **2016**, *14*, 158.
[7] S.-Y. Kim, P. Zhao, M. Igarashi, R. Sawa, T. Tomita, M. Nishiyama, T. Kuzuyama, *Chem. Biol.* **2009**, *16*, 736.
[8] J. Rinkel, P. Rabe, P. Garbeva, J. S. Dickschat, *Angew. Chem. Int. Ed.* **2016**, *55*, 13593.
[9] G. Li, Y.-W. Guo, J. S. Dickschat, *Angew. Chem. Int. Ed.* **2021**, *60*, 1488.
[10] P. Rabe, J. S. Dickschat, *Angew. Chem. Int. Ed.* **2013**, *52*, 1810.
[11] J. Rinkel, S. T. Steiner, J. S. Dickschat, *Angew. Chem. Int. Ed.* **2019**, *58*, 9230.
[12] L. Lauterbach, J. Rinkel, J. S. Dickschat, *Angew. Chem. Int. Ed.* **2018**, *57*, 8280.
[13] P. Rabe, J. Rinkel, E. Dolja, T. Schmitz, B. Nubbemeyer, T. H. Luu, J. S. Dickschat, *Angew. Chem. Int. Ed.* **2017**, *56*, 2776.
[14] P. Baer, P. Rabe, K. Fischer, C. A. Citron, T. A. Klapschinski, M. Groll, J. S. Dickschat, *Angew. Chem. Int. Ed.* **2014**, *53*, 7652.
[15] Y. Hu, W. K. W. Chou, R. Hopson, D. E. Cane, *Chem. Biol.* **2011**, *18*, 32.
[16] P. Rabe, L. Barra, J. Rinkel, R. Riclea, C. A. Citron, T. A. Klapschinski, A. Janusko, J. S. Dickschat, *Angew. Chem. Int. Ed.* **2015**, *54*, 13448.
[17] A. Hou, J. S. Dickschat, *Angew. Chem. Int. Ed.* **2020**, *59*, 19961.
[18] Z. Li, Y. Jiang, X. Zhang, Y. Chang, S. Li, X. Zhang, S. Zheng, C. Geng, P. Men, L. Ma, Y. Yang, Z. Gao, Y.-J. Tang, S. Li, *ACS Catal.* **2020**, *10*, 5846.
[19] C. Nakano, F. Kudo, T. Eguchi, Y. Ohnishi, *ChemBioChem* **2011**, *12*, 2271.
[20] W. K. W. Chou, I. Fanizza, T. Uchiyama, M. Komatsu, H. Ikeda, D. E. Cane, *J. Am. Chem. Soc.* **2010**, *132*, 8850.
[21] D. E. Cane, J. K. Sohng, C. R. Lamberson, S. M. Rudnicki, Z. Wu, M. D. Lloyd, J. S. Oliver, B. R. Hubbard, *Biochemistry* **1994**, *33*, 5846.
[22] P. Rabe, M. Samborskyy, P. F. Leadlay, J. S. Dickschat, *Org. Biomol. Chem.* **2017**, *15*, 2353.
[23] T. A. Klapschinski, P. Rabe, J. S. Dickschat, *Angew. Chem. Int. Ed.* **2016**, *55*, 10141.
[24] J. Rinkel, J. S. Dickschat, *ChemBioChem* **2020**, *21*, 807.
[25] J. Rinkel, J. S. Dickschat, *Org. Lett.* **2019**, *21*, 9442.
[26] X. Lin, R. Hopson, D. E. Cane, *J. Am. Chem. Soc*. **2006**, *128*, 6022.
[27] S. A. Agger, F. Lopez-Gallego, T. R. Hoye, C. Schmidt-Dannert, *J. Bacteriol.* **2008**, *190*, 6084.
[28] J. S. Dickschat, J. Rinkel, P. Rabe, A. Beyraghdar Kashkooli, H. J. Bouwmeester, *Beilstein J. Org. Chem.* **2017**, *13*, 1770.
[29] J. Rinkel, J. S. Dickschat, *Org. Lett.* **2019**, *21*, 2426.
[30] J. S. Dickschat, K. A. K. Pahirulzaman, P. Rabe, T. A. Klapschinski, *ChemBioChem* **2014**, *15*, 810.
[31] A. Schifrin, T. T. B. Ly, N. Günnewich, J. Zapp, V. Thiel, S. Schulz, F. Hannemann, Y. Khatri, R. Bernhardt, *ChemBioChem* **2015**, *16*, 337.
[32] C. Nakano, T. Tezuka, S. Horinouchi, Y. Ohnishi, *J. Antibiot.* **2012**, *65*, 551.
[33] J. Rinkel, L. Lauterbach, J. S. Dickschat, *Angew. Chem.* **2017**, *129*, 16603.
[34] H. Xu, J. Rinkel, J. S. Dickschat, *Org. Chem. Front.* **2021**, *8*, 1177.
[35] P. Rabe, K. A. K. Pahirulzaman, J. S. Dickschat, *Angew. Chem. Int. Ed.* **2015**, *54*, 6041.
[36] A. Hou, B. Goldfuss, J. S. Dickschat, *Angew. Chem. Int. Ed.* **2021**, *60*, 20781.

[37] G. R. Fulmer, A. J. M. Miller, N. H. Sherden, H. E. Gottlieb, A. Nudelman, B. M. Stoltz, J. E. Bercaw and K. I. Goldberg, *Organometallics*, **2010**, *29*, 2176.
[38] R. D. Giets and R. H. Schiestl, *Nat. Protoc.* **2007**, *2*, 31.
[39] M. M. Bradford, *Anal. Biochem.* **1976**, *72*, 248.
[40] J. Rinkel, L. Lauterbach, J. S. Dickschat, *Angew. Chem. Int. Ed.* **2019**, *58*, 452.
[41] Z. Quan, J. S. Dickschat, *Org. Biomol. Chem*. **2020**, *18*, 6072.
[42] H. Li, J. S. Dickschat, *Org. Chem. Front*. **2022**, *9*, 795.
[43] G. Bian, J. Rinkel, Z. Wang, L. Lauterbach, A. Hou, Y. Yuan, Z. Deng, T. Liu, J. S. Dickschat, *Angew. Chem. Int. Ed.* **2018**, *57*, 15887.
[44] T. Mitsuhashi, J. Rinkel, M. Okada, I. Abe, J. S. Dickschat, *Chem. Eur. J.* **2017**, *23*, 10053.
[45] J. Rinkel, P. Rabe, L. zur Horst, J. S. Dickschat, *Beilstein J. Org. Chem.* **2016**, *12*, 2317.
[46] R. Higuchi, B. Krummel, R. Saiki, *Nucleic Acids Res.* **1988**, *16*, 7351.
[47] S. Grimme, S. Ehrlich, L. Goerigk, *J. Comp. Chem.* **2011**, *32*, 1456.
[48] Gaussian 16, Revision B.01, M. J. Frisch, G. W. Trucks, H. B. Schlegel, G. E. Scuseria, M. A. Robb, J. R. Cheeseman, G. Scalmani, V. Barone, G. A. Petersson, H. Nakatsuji, X. Li, M. Caricato, A. V. Marenich, J. Bloino, B. G. Janesko, R. Gomperts, B. Mennucci, H. P. Hratchian, J. V. Ortiz, A. F. Izmaylov, J. L. Sonnenberg, D. Williams-Young, F. Ding, F. Lipparini, F. Egidi, J. Goings, B. Peng, A. Petrone, T. Henderson, D. Ranasinghe, V. G. Zakrzewski, J. Gao, N. Rega, G. Zheng, W. Liang, M. Hada, M. Ehara, K. Toyota, R. Fukuda, J. Hasegawa, M. Ishida, T. Nakajima, Y. Honda, O. Kitao, H. Nakai, T. Vreven, K. Throssell, J. A. Montgomery, Jr., J. E. Peralta, F. Ogliaro, M. J. Bearpark, J. J. Heyd, E. N. Brothers, K. N. Kudin, V. N. Staroverov, T. A. Keith, R. Kobayashi, J. Normand, K. Raghavachari, A. P. Rendell, J. C. Burant, S. S. Iyengar, J. Tomasi, M. Cossi, J. M. Millam, M. Klene, C. Adamo, R. Cammi, J. W. Ochterski, R. L. Martin, K. Morokuma, O. Farkas, J. B. Foresman, D. J. Fox, Gaussian, Inc., Wallingford CT, **2016**.
[49] Y. J. Hong, D. J. Tantillo, *J. Org. Chem.* **2018**, *83*, 3780.
[50] C. Adamo, V. Barone, *J. Chem. Phys.* **1998**, *108*, 664.
[51] S. P. T. Matsuda, W. K. Wilson, Q. Xiong, *Org. Biomol. Chem.* **2006**, *4*, 530.
[52] H. Xu, B. Goldfuss, J. S. Dickschat, *Chem. Eur. J.* **2021**, *27*, 9758.
[53] S. Grimme, *Chem. Eur. J.* **2012**, *18*, 9955.
[54] GoodVibes v3.0.1, G. Luchini, J. V. Alegre-Requena, Y. Guan, I. Funes-Ardoiz, R. S. Paton, **2019**.
